# Synthesis of Nucleoside Derivatives by Biomimetic Ester Migration

**DOI:** 10.1002/cbic.202500395

**Published:** 2025-06-19

**Authors:** Nathalie J. Kurrle, Christoph J. B. Seifert, Nathalie Hampel, Tamara Rauch, Michael Thoma, Luca V. Parziale, Marian S. R. Ebeling, Dino Berthold, Oliver Trapp

**Affiliations:** ^1^ Department of Chemistry Ludwig‐Maximilians‐University Munich Butenandtstr. 5–13 81377 Munich Germany; ^2^ Max‐Planck‐Institute for Astronomy Königstuhl 17 69117 Heidelberg Germany

**Keywords:** aminoacyl shifts, biomimetic reaction, nucleosid, ribose, triflate

## Abstract

Modified nucleosides play important roles as agents in medicinal chemistry due to their anti‐inflammatory, antiviral, and antiproliferative properties, as well as in biochemical processes like protein biosynthesis. Aminoacylated nucleosides in tRNA represent the central transfer unit of amino acids in the biosynthesis of peptides. Consequently, their synthesis in a prebiotic context is of great significance for further elucidations regarding the origin of life. To verify the formation of these structures in complex mixtures of regio‐ and stereoisomers, reference structures and their synthesis are of fundamental importance. However, state‐of‐the‐art methodologies for the synthesis of monomeric tRNA nucleoside derivatives frequently result in the production of regioisomeric mixtures or encounter challenges related to isomerization. In this context, a concise and comprehensive approach for the chemical synthesis of nucleosidic amino acid esters is presented. The three‐step reaction sequence exploits the phenomenon of 2′‐3′‐transaminoacylation in nucleosides providing the desired compounds in high yields. This biomimetic approach is further expanded to the activation of hydroxy groups by application of sulfonic acid esters. This has the potential to facilitate extensive modification via substitution or cross‐coupling reactions, enabling the stereo‐ and regio‐controlled transformation of nucleosides into valuable target molecules or precursors in medicinal chemistry.

## Graphical abstract

In tRNA aminoacylated nucleosides represent the central transfer unit of amino acids. An occurring 2′‐3′‐transaminoacylation is biomimetically exploited to successfully synthesize nucleosidic acid esters. This approach can be further expanded on sulfonic acid esters, which has the potential of a broad range of stereo‐ and regio‐controlled modifications of nucleosides, resulting in precursors or target molecules in medicinal chemistry.

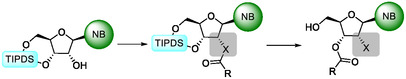

## 1 Introduction

**Scheme 1:**
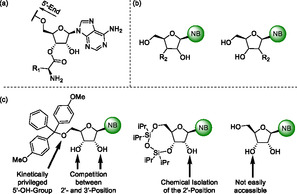
Nucleosides modified by esterification or substitution in 2′‐ or 3′‐position with relevance in either nature or medicinal chemistry. a) tRNA nucleoside equipped with an amino acid. R_1_ = amino acid residue. b) Examples for 2′‐ or 3′‐modified ribonucleosides. –NB is any canonical or noncanonical nucleobase. R_2_ = –SH, –F, –Cl,^[^ ^20^ ^]^ –Br,^[^ ^21^ ^]^ –OMe,^[^ ^13^ ^]^ –NH_2_.^[^ ^22^, ^23^, ^24^ ^]^ c) Synthesis strategies for nucleosides with modified sugar moieties.

It is a well‐established fact that RNA nucleosides are composed of a nucleobase which is linked to a ribose moiety via a *N*‐glycosidic bond. They constitute a part of nucleotides, which, in turn, form oligomeric or polymeric RNAs by linking the nucleosides via phosphoric acid diesters. While the majority of RNA nucleosides consist of canonical ribonucleosides, a significant proportion of those found in nature are also epigenetically modified.^[^ ^1^, ^2^, ^3^ ^]^ However, there are also examples of nucleosides that contain modified sugars.^[^ ^4^, ^5^ ^]^ A prominent example of this is the terminal adenosine on the acceptor stem of tRNAs, which is loaded with amino acids via esterification in the course of protein biosynthesis. A 2′‐3′‐aminoacyl shift is commonly observed in this context.^[^ ^6^ ^]^ An example of such an amino acid ester is illustrated in **Scheme** **1**a with a generic amino acid residue.

The chemical origin of acylated nucleosides (Scheme 1a) in nature remains to be fully elucidated, and the prebiotic syntheses of tRNA derivatives are associated with numerous challenges. The analysis of complex mixtures resulting from reactions containing prebiotically plausible substrates, such as short‐chain aldehydes and nucleobases, often gives rise to several isomers with the same exact mass.^[^ ^7^, ^8^ ^]^ Elucidation of the exact structures is often challenging, due to the presence of a large number of products that vary constitutionally and stereomerically. This necessitates the use of a multitude of reference compounds, even when working with a limited number of initial materials, to ensure precise identification and characterization.

As illustrated in Scheme 1c, a number of chemical syntheses have been developed for the introduction of esters in the 2′‐ or 3′‐position of nucleosides.^[^ ^9^ ^]^ Either a sterically demanding protecting group (e.g., DMT = 4,4′‐dimethoxytrityl) is first installed on the kinetically privileged 5′‐hydroxy group and the nucleoside is then subjected to esterification conditions or the reaction is performed without any protecting group at all.^[^ ^10^ ^]^ Depending on the size of the carboxylic acid, a mixture of 2′‐ and 3′‐substituted nucleosides is obtained. These can be separated by column chromatography, yet in our experience isomerization of isolated compounds back to regioisomeric mixtures can occur both on‐column and in solution. This is a significant issue as it is detrimental to their application as reference compounds for the exact confirmation of the presence of specific compounds in complex mixtures.

While 3′,5′‐protection with cyclic silyl ethers such as TIPDS (1,1,3,3‐tetraisopropyl‐disiloxanes) or DTBS (di‐*tert*‐butylsiloxanes) offers the potential for selective modifications in the 2′‐position, no such facile route is known for the 3′‐position.^[^ ^11^ ^]^ Instead, a sequence of several protection–deprotection steps is required to selectively modify this position or obtained regioisomers have to be separated by chromatography. This is particularly disadvantageous for noncanonical nucleosides with complex prior syntheses as yields are often diminished by an increased number of reaction and purification steps.^[^ ^12^ ^]^

Furthermore, nucleosides containing entirely other functional groups than the 2′‐ or 3′‐hydroxy group represent important target molecules and precursors thereof in medicinal chemistry.^[^ ^13^ ^]^ Generic structures of these compounds are also depicted in Scheme 1b and—among others—including halogenated derivatives such as 3′‐deoxy‐3′‐fluoroadenosine. These unnatural nucleosides are considered to be applied as anti‐inflammatory, antiviral, or antitumor‐proliferation agents or act as valuable precursors in their synthesis.^[^ ^14^, ^15^, ^16^, ^17^, ^18^ ^]^ However, no general synthetic pathway toward modified 2′‐ or 3′‐nucleosides has been established.

In order to surmount these challenges, a concise protection–modification–deprotection sequence is hereby presented, involving an in situ biomimetic ester migration to access both biologically relevant carboxylic esters and activated nucleosides. These can then be transformed into desired target molecules for medicinal or synthetic chemistry. The reaction sequence is composed of a limited number of steps and offers full control over regio‐ and stereochemistry at the 2′‐ and 3′‐position.

**Scheme 2:**
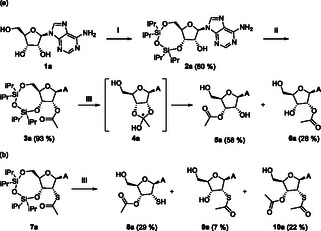
a) Synthesis of 2′‐acetyl‐protected adenosine **6a** from a 3′,5′‐TIPDS protected precursor **3a** resulting in a mixture of 2′‐ and 3′‐esters. **4a** is a proposed intermediate. Conditions: i) TIPDSCl_2_, pyridine; ii) AcOH, DMAP, EDCI, THF; iii) HF·pyridine, THF; A = adenine. b) Migration reaction in thio‐*S*‐esters.

In the course of our studies regarding the synthesis of reference substances for prebiotic investigations, we observed a mixture of acetyl adenosine regioisomers during the silyl deprotection of 3′,5′‐TIPDS‐protected precursor **2a** (**Scheme** **2**a). Extensive NMR studies including 2D correlation experiments revealed the presence of both 2′‐ and 3′‐acylated adenosines **5a** and **6a**. We monitored the course of the deprotection via ^1^H‐NMR analysis to confirm the partial in situ migration from the 2′‐ to the 3′‐position during the reaction. The formation of the 5′‐acylated compound was not observed, presumably due to the opposing spatial orientation and distance of the 2′‐/3′‐ compared to the 5′‐hydroxy group. The ratio of the two regioisomers present shifted in favor of the 3′‐isomer over time in solution. Furthermore, the presence of partially deprotected intermediates was observed and confirmed by HRMS. Due to steric hinderance, it is anticipated that the cyclic silyl ether of **3a** will be cleaved at the 5′‐position first.

DFT calculations demonstrated that the 3′‐derivative **5a** should exhibit a greater stability of 15.8 kJ mol^−1^ in comparison with the 2′‐derivative **6a** in THF. This is equivalent to an equilibrium constant of 0.0171 or a ratio of 1:586 of 2′‐ to 3′‐ester. In accordance with the findings of Sakamoto et al. the shift may occur via a cyclic intermediate,^[^ ^19^ ^]^ which, in our case, introduces an additional asymmetric center in **4a.** This intermediate, depending on the absolute configuration of the ortho di‐ester, is 0.3 and 4.1 kJ mol^−1^ less stable than the 2′‐isomer **6a**, respectively. This result is in good agreement with our findings and suggests that the equilibrium should shift even further with prolonged time. In order to further investigate the acyl shift on a substrate with different electronic and steric properties—and since the focus was also on sulfur‐containing compounds with regard to prebiotic synthesis—a 2′‐thioacetate silyl‐protected adenosine **7a** was synthesized. The subsequent silyl‐deprotection, employing HF·pyridine, resulted in a mixture of regioisomers (**8a**:**9a **= 81:19), thereby demonstrating that the migration is not confined to oxygen‐derived acyl groups (Scheme 2b). Furthermore, a twice‐acylated product on **10a** could be isolated, showing not only an intramolecular but also an intermolecular acyl shift. This can be attributed to the enhanced nucleophilicity of the free thiol in 2′‐position in **8a**.

**Scheme 3:**
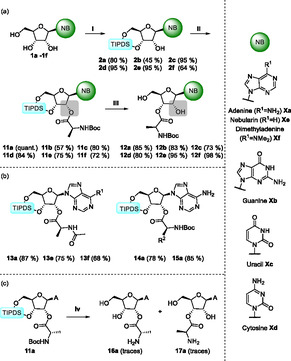
a) Synthesis of protected 2′‐aminoacylated nucleosides followed by 2′‐3′‐aminoacyl migration. NB = canonical nucleobases (A, C, G, U) and adenine derivatives purine and *N* ^6^,*N* ^6^‐dimethyladenine. **Xa** = adenosine, **Xb** = guanosine, **Xc** = cytidin, **Xd** = uridine, **Xe** = nebularine, **Xf** = *N* ^6^,*N* ^6^‐dimethyladenosine. Conditions: i) TIPDSCl_2_, pyridine; ii) Boc‐*N*‐Ala‐OH, DMAP, EDCI, THF; iii) HF·pyridine, THF. b) Synthesized *N*‐acetyl‐protected alanine‐silyl‐adenosine, –nebularin and –*N* ^6^,*N* ^6^‐dimethyladenosine (**13a** R^1^ = –NH_2_, **13e** R^1^ = –H, **13f** R^1^ = –NMe_2_) as well as *N*‐Boc‐valine‐ and phenylalanine‐silyl‐adenosine (**14a** R^2^ = –iPr, **15a** R^2^ = –Bn); c) deprotection of **11a** results in a mixture of **16a** and **17a**. Conditions: iv) either only 1), or 1) followed by 2). 1) HCl, 1,4‐dioxane; 2) HF·pyridine, THF. A = adenine.

In order to apply the biomimetic migration process to a substrate more closely related to tRNA nucleosides, the 2′‐ester of *N*‐Boc‐protected alanine **11a** from adenosine was prepared as a model substrate. The Steglich esterification conditions were employed providing **11a** in high yields (**Scheme** **3**a). Subsequently, a variety of fluoridic and acidic deprotection reagents and conditions for the following silyl ether cleavage were tested. This investigation revealed that the only suitable reagents were indeed HF complexes with nitrogen bases—in detail HF·pyridine and HF·triethylamine (**Table** **1**, entries 7 and 8). These reagents were found to result in conversion rates exceeding 80%, with reaction times of ≈18 h, while the use of alternative reagents often led to either inadequate conversion (entries 4 and 5) or decomposition (entries 1, 2, 3, and 6). In addition to these observations, the ratio of 2′‐ to 3′‐isomers was determined.

**Table 1:** Overview of the screening of tested deprotection reagents.

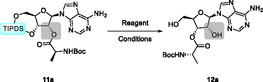				
#	Reagenta)	Result	Total yield	Fraction of 3′‐isomer 12a
1	CsF	Decomposition	–	–
2	HClb)	Partial decomposition	53%c)	42%
3	TBAFd)	Decomposition	–	–
4	NaF	No conversion	–	–
5	NH_4_F	No conversion	–	–
6	TASF	Severe decomposition	Traces	–
7	HF·NEt_3_ e)	Good conversion	82%	>95%
8	HF·Pyridinef)	Good conversion	85%	>95%

a) Exact conditions are provided in the supporting information to this communication.

b) 4 m in 1,4‐dioxane.

c) No Boc‐deprotected product with an intact silyl‐protection group isolated. Yield is given with respect to the sum of the fully and partially deprotected compounds.

d) As solution 1 m in THF or as solid TBAF·H_2_O.

e) NEt_3_·3HF.

f) 70 wt% HF.

In order to extend the scope of our findings, we prepared the 2′‐nucleoside esters of *N*‐Boc‐alanine **11b–d** for the remaining canonical RNA nucleosides according to our previously described route (Scheme 3a). It is notable that all conversions proceeded in high yields; however, the conversion of the guanosine‐derived products was impeded by solubility issues. Under optimized conditions (HF·pyridine), all canonical nucleosides were deprotected—also in high yields—and analyzed with regard to the position of their respective amino acid ester moiety as described above for the acetyl derivative. Nearly quantitative migration from the 2′‐ to the 3′‐position was observed for all canonical nucleosides **12a–d**. Furthermore, the influence of noncanonical nucleobases, TIPDS‐nebularine‐Boc‐alanine **11e** and TIPDS‐*N* ^6^,*N* ^6^‐dimethyladenosine‐Boc‐alanine **11f**, on their capability of performing an in situ migration to the 3′‐position was investigated, resulting in a complete shift to yield **12e** and **12f**. In addition, the ester migration process was examined on derivatives of supplementary *N*‐Boc amino acids, namely, valine **(14a)** and phenylalanine **(15a)** (Scheme 3b). These experiments demonstrated complete migration, and the desired products were obtained in high yields. These findings suggest that the observed migration reaction is independent from nucleobase and amino acid. In order to investigate molecules with even higher prebiotic relevance,^[^ ^10^ ^]^ the behavior of acetyl‐protected alanine–adenosine **13a** and its derivatives nebularine **13e** and *N* ^6^,*N* ^6^‐dimethyladenosine **13f** (Scheme 3b) were elucidated. As was previously observed, a complete migration was once again detected, which demonstrates that the nature of the amino acid protecting group does not have a significant influence on the final distribution of regioisomers. However, the steric demand of the ester appears to exert a substantial influence on the efficiency of the acyl‐shift. This hypothesis was corroborated by DFT calculations, which revealed that for Boc‐protected **12a** and unprotected **16a/17a** the 3′‐compound was 24.7 and 20.3 kJ mol^−1^ more stable than their respective 2′‐isomers.

In order to mimic the biological reaction as closely as possible in our synthesis, it was necessary to demonstrate the migration reaction on both unprotected alanine and adenosine. Starting from **11a**, a Boc‐deprotection was first performed (Table 1, entry 2), resulting in two different products. The major species was identified as a Boc‐deprotected product with a partially hydrolyzed silyl ether still attached, while the minor species was found to be the completely deprotected alanine–adenosine without either protecting group (also see Table 1, entry 2). NMR analysis of the latter revealed only a partial migration of the alanine ester resulting in a regioisomeric mixture (**16a**:**17a** = 58:42). Subsequently, the partially silyl‐protected adenosine‐2′‐alanine was deprotected using HF·pyridine. This process also resulted in the formation of a regioisomeric mixture of the alanine–adenosine, with the majority being 3′‐alanine ester nucleoside (**16a**:**17a** = 17:83). This outcome serves to underscore the pivotal role of the base in facilitating the HF deprotection process, thereby effecting a more rapid shift in equilibrium toward the 3′‐isomer **17a** (Scheme 3c).

In the course of the investigation, a reversal of the order of the deprotection steps of the adenosine derivative **11a** was tested—silyl‐deprotection was initiated first, followed by Boc‐deprotection—which resulted in the 3′‐aminoacetylated product **17a** as the sole product in excellent yield. It was observed that there was no further migration of the ester during the Boc‐deprotection from this point onward. However, it should be noted that all fully deprotected compounds are particularly susceptible to hydrolysis.

**Scheme 4:**
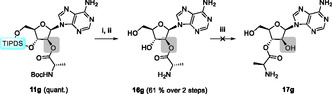
Boc‐alanine‐TIPDS‐vidarabine (**11g**) showing no ester migration upon deprotection to **16g**. Conditions: i) HF·pyridine, THF; ii) HCl, 1,4‐dioxane; iii) DBU, ACN.

As our findings revealed no dependence on any particular nucleobase or amino acid, we then investigated arabinose‐based structures. In a manner analogous to the respective ribose counterpart and commencing from vidarabine, we performed protection and esterification in a manner analogous to the adenosine derivatives—isolated in high overall yields. *N*‐Boc‐protected aminoacylated vidarabine (**11g**) demonstrated no indications of migration during silyl‐deprotection using HF·pyridine or the subsequent Boc‐deprotection, as depicted in **Scheme** **4**.

In conditions that induce analogous acyl shifts, no such migration was observed.^[^ ^19^ ^]^ This phenomenon can be attributed to the spatial configuration of the 2′‐ and 3′‐hydroxy groups in vidarabine, which, in the context of prebiotic chemistry, might have led to an enhanced selection of ribose over arabinose in tRNA nucleosides.

**Scheme 5:**
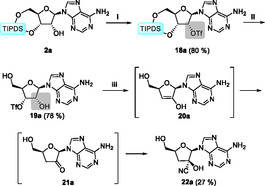
Synthesis and derivatization for determination of the regiochemistry of the sulfonic acid ester **19a** via cyanhydrine **22a**. Conditions: i) Tf_2_O, DMAP, DCM; ii) HF·pyridine, THF; iii) KCN, DMF.

Building on the findings from previous studies on the migration of carboxylic acid esters, the objective was to expand the scope of investigation to include sulfonic acid esters. To this end, the 2′‐hydroxy function of **2a** was converted to the corresponding triflate **18a** using triflic anhydride and DMAP, with a high yield. Following the silyl‐ether cleavage, the determination of the position of the sulfonic ester was hindered by the absence of favorable NMR‐active nuclei and the unreliable visibility of the remaining–OH proton. To address this challenge, we employed a derivatization protocol using cyanide, which ultimately yielded the desired cyanhydrine **22a**. This process is depicted in **Scheme** **5**.

The regiochemistry of compound **22a** could be unambiguously assigned to be 2′‐cyanhydrine by means of NMR spectroscopy. In consideration of the proposed mechanism of its formation, it can be concluded that the sulfonic ester **19a** was the 3′‐isomer, thereby demonstrating that the 2′‐3′‐migration of esters is not limited to carboxylic acids.

In conclusion, a simple protocol has been developed to access 3′‐esterified nucleosides that are both relevant in biochemical systems and valuable precursors in synthetic and medicinal chemistry. The protocol is a three‐step process involving protection, modification, and deprotection, with the ester being transferred from the 2′‐ to the 3′‐position during deprotection via a biomimetic migration reaction. The efficacy of this method was demonstrated through the successful implementation on carboxylic acid esters, as well as sulfonic acid esters, specifically triflates.

**Figure 1:**
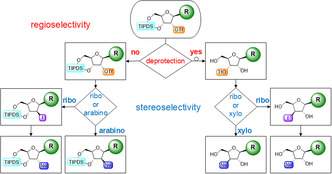
Decision tree for the regioselective and stereoselective modification of nucleosides by triflate migration initiated by deprotection of TIPDS or continuation of synthesis with protective groups. R represents a (modified) canonical nucleobase.

Furthermore, the activation of hydroxyl groups in the form of triflates could allow for regioselective and stereoselective functionalization of the 2′‐ or 3′‐position via substitution or cross‐coupling reactions, as illustrated in **Figure** **1**.

The choice of suitable solvents and nucleophiles for S_N_2 reactions could result in the inversion of the stereocenters. In the event of modification in the 2′‐position being required, the substitution reaction should be performed on the silyl‐protected riboside, resulting in the respective arabino derivative. The implementation of deprotection prior to further modification inevitably results in the migration of the triflate, thereby rendering the 3′‐position accessible for functionalization. This process would yield substituted xylofuranose derivatives. Should retention of the original stereocenters be desired a simple iodide substitution followed by the intended modification can be performed to obtain the ribofuranose derivative. The suggested reaction concept offers full control over regio‐ and stereochemistry of the 2′‐ and 3′‐position, depending on the order of modification and deprotection steps.

## Conflict of Interest

The authors declare no conflict of interest.

## Supporting information

Supplementary Material

## Data Availability

The data that support the findings of this study are available in the supplementary material of this article.
