# Supplementary material for: Synthesis of Nucleoside Derivatives by Biomimetic Ester Migration
Source: Chembiochem. 2025 Jun 19;26(14):e202500395. doi: 10.1002/cbic.202500395 (PMC12278338; doi:10.1002/cbic.202500395)
Supplement: Supplementary file 1 — Supplementary Material [file CBIC-26-e202500395-s001.pdf]

Supporting Information

Synthesis of Nucleoside Derivatives by Biomimetic Ester Migration

Nathalie J. Kurrle‡[a], Christoph J. B. Seifert‡[a], Nathalie Hampel[a], Tamara Rauch[a], Michael Thoma[a], Luca V. Parziale[a], Marian S. R. Ebeling[a], Dino Berthold[b], Oliver Trapp*[a,c]

[a] N. J. Kurrle, C. J. B. Seifert, N. Hampel, T. Rauch, M. Thoma, L. V. Parziale, M. S. R. Ebeling, O. Trapp
Department of Chemistry
Ludwig-Maximilians-University Munich
Butenandtstr. 5–13, 81377 Munich, Germany
E-mail: oliver.trapp@cup.uni-muenchen.de

[b] D. Berthold
Department of Chemistry
Ludwig-Maximilians-University Munich
Butenandtstr. 5–13, 81377 Munich, Germany
E-mail: dino.berthold@cup.uni-muenchen.de

[c] O. Trapp
Max-Planck-Institute for Astronomy
Königstuhl 17, 69117 Heidelberg

‡ Authors contributed equally.

Contents

General Considerations 2

Synthetic Procedures and Characterisation of Isolated Compounds 4

- Adenosine-based Compounds 4
- Vidarabine-based Compounds 31
- Guanosine-based Compounds 41
- Uridine-based Compounds 44
- Cytidine-based Compounds 47
- N^6,N^6-Dimethyladenosine-based Compounds 50
- Nebularine-based Compounds 57

NMR Spectra 65

DFT Calculations 114

Supplemental Bibliography 131

General Considerations

Moisture- and air-sensitive reactions were performed in flame-dried glassware equipped with a PTFE-coated stir bar under an atmosphere of argon or nitrogen.

All **chemicals and solvents** were purchased from commercial sources (ABCR GmbH, THERMO FISHER SCIENTIFIC GmbH, BLD PHARMATECH GmbH, MERCK KGaA, TCI DEUTSCHLAND GmbH) and were stored according to their respective instructions. Dry solvents were obtained from a Solvent Purification System MB SPS-800 (M. BRAUN Inc.). Argon (Ar 5.0) and nitrogen was purchased from AIR LIQUIDE DEUTSCHLAND GmbH. Pure water was dispensed from a VWR Puranity PU 15 UV water purification system.

Flash column chromatography was performed manually using silica (35–70 µm, 60 A) as the stationary phase or using INTERCHIM XS520Plus or 5050 instruments with 15SIHP or 30C18HP columns of appropriate capacity. Thin layer chromatography (TLC) was performed on Polygram SIL G/UV254 (MACHEREY-NAGEL GmbH & Co. KG) silica-coated plates and compounds were made visible by ultraviolet light (254 nm) or by using staining methods (dinitrophenylhydrazine, potassium permanganate, iodine vapor).

Nuclear magnetic resonance (**NMR**) spectra were recorded on a BRUKER Avance III HD (400 MHz), a 600 MHz VARIAN NMR-System or a BRUKER Avance III HD (800 MHz). Chemical shift values (*δ*) are reported in ppm and calibrated using the residual solvent peak. Coupling constants (*J*) are given in Hertz (Hz). Multiplicities are given as follows: s (singlet), d (doublet), t (triplet), q (quartet), p (pentet), m (multiplet), br s (broad singlet) and by combination of these. Assignment of most peaks was accomplished by 2D-NMR spectroscopy ($^{1}H/^{1}H$ COSY, $^{1}H/^{13}C$ HSQC, $^{1}H/^{13}C$ HMBC).

High resolution mass spectra (**HRMS**) were recorded on a THERMO SCIENTIFIC GmbH Orbitrap Q Exactive Plus mass spectrometer. Ionization was achieved by electrospray ionization after direct injection. The sample was dissolved or diluted in acetonitrile, methanol, isopropanol or ultrapure water. Isopropanol/water (80:20) + 0.05 % formic acid was used as the injection solvent. The flow rate was set to 0.05–0.10 mL/min. Ions were analysed in the scan range of *m/z* = 85 – 850 m/z with a resolution of 280000 in positive mode. The following parameters were applied during mass analysis: Sheath gas flow: 2 L/min, auxiliary gas flow: 1 L/min, spray voltage: 4 kV, spray current: 0.80 µA, capillary temperature: 280 °C.

UPLC coupled with **HRMS** was performed on an AGILENT 1260 Infinity II LC system coupled with a downstream AGILENT 6550 iFunnel Q-TOF/MS. A ZORBAX Extend C18 (2.1 x 50 mm, 1.80 µm particle size) column was used. During analysis, its temperature was constantly set to 40 °C. The mobile phase was a gradient made up from water containing 0.05 % formic acid (eluent A) and methanol (eluent B). Gradient: 0 % B was held constant for 0.54 min, then was increased to 40 % B within 2.38 min and held for 0.59 min. Subsequently, eluent B was increased to 100 % within 0.03 min and held for 0.60 min to flush the column. Then, the starting conditions were reconstituted within 0.03 min, and re-equilibration with 0 % B for 6.86 min resulted in a total analysis time of 11.0 min. The liquid flow was set to a constant 0.55 mL/min.

Freeze drying was performed on a CHRIST Alpha 1-2 LDPlus system. Samples were frozen in liquid nitrogen before they were subjected to lyophilisation.

Synthetic Procedures and Characterisation of Isolated Compounds

Adenosine-based Compounds

(6a*R*,8*R*,9*R*,9a*S*)-8-(6-amino-9*H*-purin-9-yl)-2,2,4,4-tetraisopropyltetrahydro-6*H*-furo[3,2-*f*][1,3,5,2,4]trioxadisilocin-9-ol (**2a**)

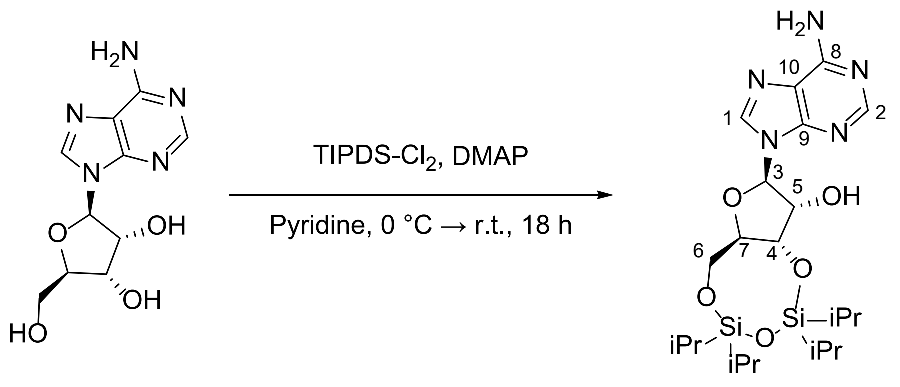

1a **2a**

Adenosine (**1a**) (8.02 g, 30.0 mmol, 1.00 equiv.) and 4-dimethylaminopyridine (DMAP; 1.83 g, 15.0 mmol, 0.50 equiv.) were suspended in dry pyridine (75 mL) and the mixture was cooled to 0 °C. 1,3-Dichloro-1,1,3,3-tetraisopropyldisiloxane (TIPDS-Cl_2; 11.5 mL, 36.0 mmol, 1.20 equiv.) was added dropwise and the mixture was slowly warmed to r.t. overnight. Cold water (20 mL) was added, once TLC indicated full conversion (18 h) and all volatiles were removed under reduced pressure. The residue was dissolved in EtOAc (100 mL) and the solution was washed with sat. aqueous NaCl solution (50 mL). The organic phase was dried over Na_2SO_4 concentrated *in vacuo* and the residue was purified *via* column chromatography (DCM/MeOH 98:2 → DCM/MeOH 94:4) to yield **2a** (12.2 g, 23.9 mmol, 80 %) as a colourless foam.

HR-MS (ESI): m/z calculated for $C_{22}H_{40}N_5NaO_5Si_2^+$: 510.2562; found: 510.2567.

^{1}H-NMR (400 MHz, DMSO): δ [ppm] = 8.21 (s, 1H, H^1), 8.07 (s, 1H, H^2), 5.87 (s, 1H, H^3), 5.64 (s, 1H, H^{OH}), 4.79 (dd, *J* = 8.5, 5.1 Hz, 1H, H^4), 4.52 (s, 1H, H^5), 4.06 (dd, *J* = 12.7, 3.4 Hz, 1H, H^{6a}), 4.00 (dt, *J* = 8.6, 3.0 Hz, 1H, H^7), 3.93 (dd, *J* = 12.7, 2.7 Hz, 1H, H^{6b}), 1.04 (s, 28H, H^{iPr}).

^{13}C{^{1}H}-NMR (101 MHz, DMSO): δ [ppm] = 156.6 (C^8), 153.0 (C^2), 149.1 (C^9), 139.7 (C^1), 119.7 (C^{10}), 89.8 (C^3), 81.2 (C^7), 74.1 (C^5), 70.2 (C^4), 61.2 (C^6), 17.9 – 12.4 (12C, C^{iPr}).

R_f: 0.42 (EtOAc 100 %).

(6a*R*,8*R*,9*R*,9a*R*)-8-(6-amino-9*H*-purin-9-yl)-2,2,4,4-tetraisopropyltetrahydro-6*H*-furo[3,2-*f*][1,3,5,2,4]trioxadisilocin-9-yl acetate (**3a**)

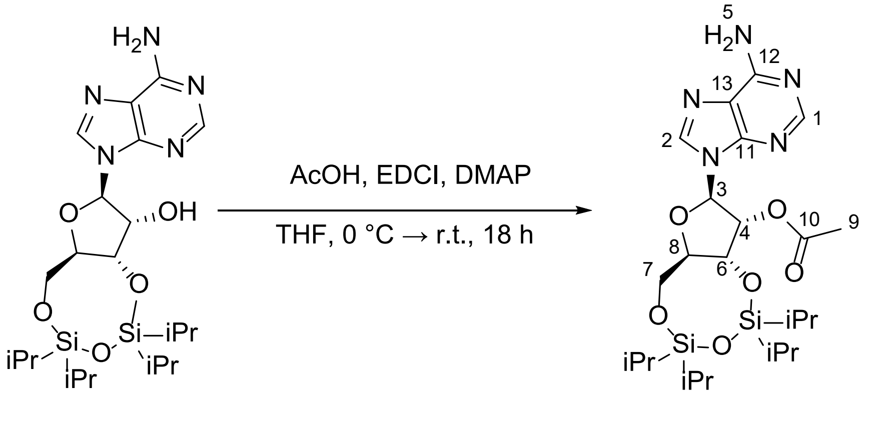

2a **3a**

Acetic acid (57.2 µL, 1.00 mmol, 1.00 equiv.) was dissolved in dry THF and the solution was cooled to 0 °C. EDCI·HCl (211 mg, 1.10 mmol, 1.10 equiv.) was added and the resulting mixture was stirred for 15 min. TIPDS-protected adenosine **2a** (510 mg, 1.00 mmol, 1.00 equiv.) and DMAP (183 mg, 1.50 mmol, 1.50 equiv.) were added and the suspension was stirred for 18 h at r.t. until TLC analysis confirmed full conversion of the starting material. All volatiles were removed under diminished pressure and the residue was purified *via* flash column chromatography (EtOAc 100 %). Ester **3a** (515 mg, 933 µmol, 93 %) was obtained as a white solid.

HR-MS (ESI): m/z calculated for $C_{24}H_{42}N_5O_6Si_2^+$: 552.2668; found: 552.2668.

^{1}H-NMR (400 MHz, CDCl$_3$): δ [ppm] = 8.30 (s, 1H, H^1), 7.98 (s, 1H, H^2), 6.03 (s, 1H, H^3), 5.77 (d, *J* = 5.3 Hz, 1H, H^4), 5.72 (s, 2H, H^5), 5.06 (t, *J* = 7.0 Hz, 1H, H^6), 4.15 (t, *J* = 15.1 Hz, 1H, H^{7a}), 4.03 (d, *J* = 13.4 Hz, 2H, $H^{7b,8}$), 2.17 (s, 3H, H^9), 1.06 (ddd, *J* = 21.6, 12.4, 5.9 Hz, 28H, H^{iPr}).

^{13}C{^{1}H}-NMR (101 MHz, CDCl$_3$): δ [ppm] = 169.5 (C^{10}), 155.6 (C^{11}), 153.3 (C^1), 149.3 (C^{12}), 139.3 (C^2), 120.4 (C^{13}), 87.5 (C^3), 82.2 (C^8), 75.8 (C^4), 69.1 (C^6), 60.7 (C^7), 20.9 (C^9), 17.6 – 12.9 (12C, C^{iPr}).

R$_f$: 0.49 (EtOAc/MeOH 98:2).

(2*R*,3*R*,4*R*,5*R*)-2-(6-amino-9*H*-purin-9-yl)-4-hydroxy-5-(hydroxymethyl)tetrahydrofuran-3-yl acetate (**5a**)

(2*R*,3*S*,4*R*,5*R*)-5-(6-amino-9*H*-purin-9-yl)-4-hydroxy-2-(hydroxymethyl)tetrahydrofuran-3-yl acetate (**6a**)

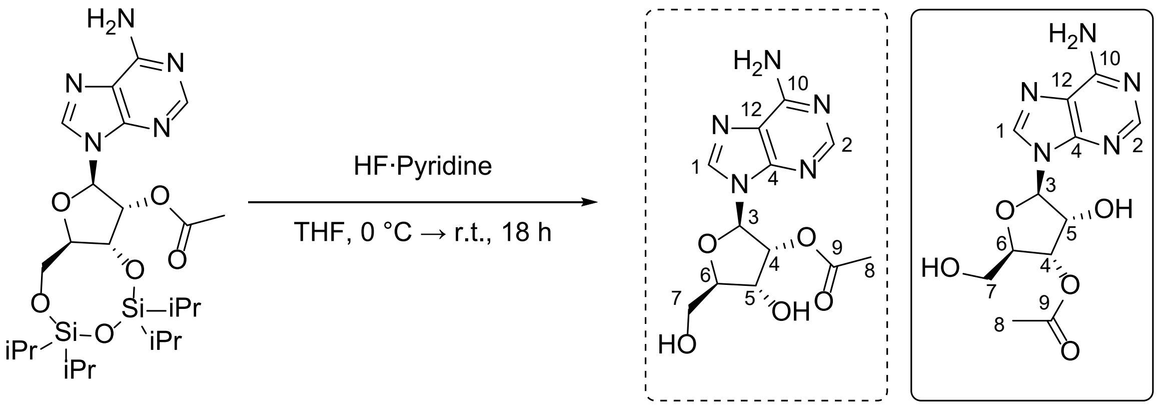

3a **5a** 32 : 68 **6a**

TIPDS-protected Ester **3a** (214 mg, 389 µmol, 1.00 equiv.) was dissolved in dry THF (2.3 mL) in a PTFE vessel. HF·pyridine (70 wt.%; 200 µL, 7.75 mmol, 20.0 equiv.) was added and the mixture was stirred for 18 h until TLC analysis confirmed full conversion. Excess fluoride was destroyed by adding TMS-OMe (3.0 mL) and the reaction was further stirred for 30 min. All volatiles were removed *in vacuo* and the colourless residue was purified by flash column chromatography (EtOAc/MeOH 98:2) to receive a mixture of 2'- and 3'-acetyl adenosine **5a** and **6a** (102 mg, 331 µmol, 85 % sum of the two regioisomers). The ratio of the 2'- to 3'-isomer was 1:2 according to ^{1}H-NMR integrals, however the ratio was subject to shift in solution in favour of the 3'-regioisomer.

HR-MS (ESI): m/z calculated for $C_{12}H_{16}N_5O_5^+$: 310.1146.; found: 310.1145.

R$_f$: 0.08 (No separation between isomers observed; EtOAc/MeOH 98:2).

2'-Isomer **5a**:

^{1}H-NMR (400 MHz, MeOD): δ [ppm] = 8.30 (s, 1H), 8.18 (s, 1H, H^1), 5.97 (d, *J* = 7.2 Hz, 1H, H^2), 5.40 (dd, *J* = 5.4, 1.9 Hz, 1H, H^3), 4.98 (dd, *J* = 7.3, 5.4 Hz, 1H, H^4), 4.28 (q, *J* = 2.3 Hz, 1H, H^5), 4.19 (q, *J* = 2.8 Hz, 1H, H^6), 3.90 (td, *J* = 12.9, 2.4 Hz, 1H, H^{7a}), 3.78 (dt, *J* = 12.6, 2.5 Hz, 1H, ^{7b}), 2.17 (s, 3H, H^8).

$^{13}C\{^1H\}$-NMR (101 MHz, MeOD): δ [ppm] = δ 170.6 (C9), 156.0 (C10), 152.0 (C2), 148.4 (C11), 140.4 (C1), 119.5 (C5), 89.3 (C3), 84.6 (C6), 73.7 (C4), 72.4 (C5), 61.8 (C7), 19.3 (C8).

3'-Isomer **6a**:

^1H-NMR (400 MHz, MeOD): δ [ppm] = 8.33 (s, 1H, H^1), 8.18 (s, 1H, H^2), 6.20 (d, *J* = 6.1 Hz, 1H, H^3), 5.67 (dd, *J* = 6.0, 5.2 Hz, 1H, H^4), 4.64 (dd, *J* = 5.2, 3.4 Hz, 1H, H^5), 4.19 (q, *J* = 2.8 Hz, 1H, H^6), 3.90 (td, *J* = 12.9, 2.4 Hz, 1H, H^{7a}), 3.78 (dt, *J* = 12.6, 2.5 Hz, 1H, H^{7b}), 2.08 (s, 3H, H^8).

$^{13}C\{^1H\}$-NMR (101 MHz, MeOD): δ [ppm] = 170.2 (C9), 156.0 (C10), 152.1 (C2), 148.5 (C11), 140.2 (C1), 119.3 (C5), 87.0 (C3), 86.7 (C6), 75.6 (C4), 69.4 (C5), 61.5 (C7), 19.0 (C8).

NMR Experiment

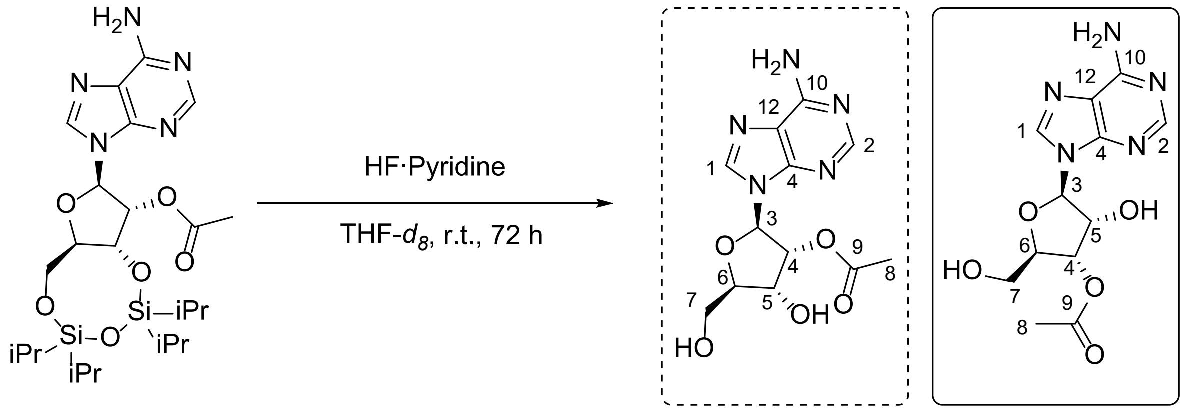

3a **5a** **6a**

Ester **3a** (10.5 mg, 19.0 µmol, 1.00 equiv.) was dissolved in THF-d_8 (0.75 mL) in a NMR tube equipped with a PTFE inlet. HF·pyridine (70 wt.%; 7.90 µL, 381 µmol, 20.0 equiv.) was added and the reaction progress was tracked with repeated ^{1}H-NMR analysis at 20 °C. For the first hour spectra were recorded every 10 min, for the next 2 h every 30 min, for the next 9 h every 1 h and after that every 3 h.

The acetyl protons 8 in **3a**, **5a**, **6a** and any possible intermediates (all at approximately 2.10 ppm) were monitored in order to follow the reaction progress. Several different species were observed this way.

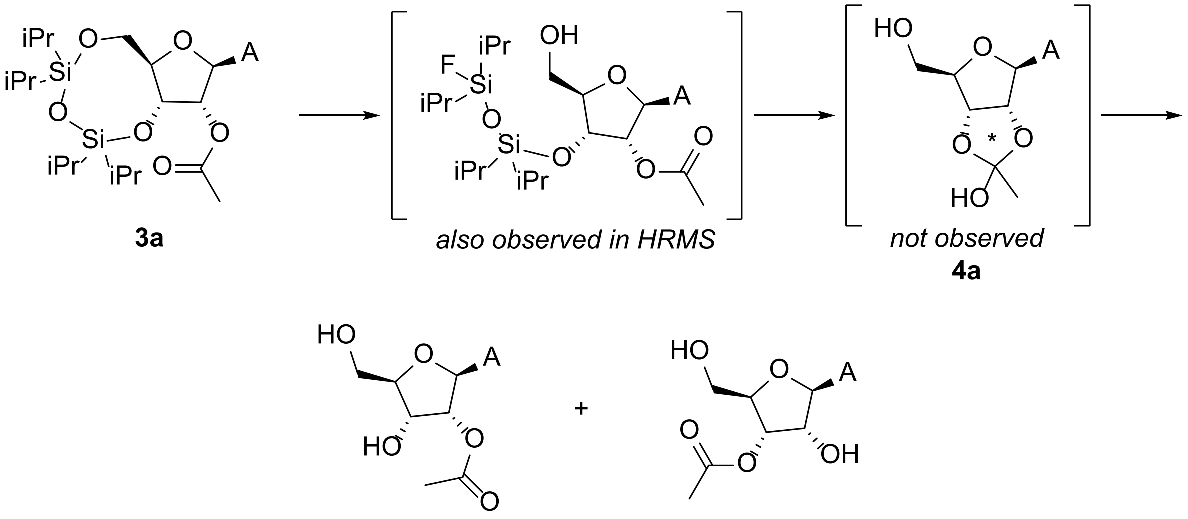

3a *also observed in HRMS* *not observed* **4a**

5a **6a**

The most prominent ones can be seen in **Figure 1** below. Reactant **3a** and products **5a** and **6a** could be unambiguously assigned with reference compounds and *via* HMBC-NMR (correlation illustrated in **Figure 2**). The higher ratio of **5a**:**6a** in **Figure 1**

at 24 h when compared to the aforementioned reactions can be explained by the absence of stirring in the NMR tube, as well as lower reaction temperatures in comparison to the standard reaction due to climate control in the NMR room. With prolonged reaction time the ratio eventually shifts in favour of the 3’-isomer. The proposed intermediate **4a** for similar acyl shift reactions proposed by SAKAMOTO *et. al.* could not be unambiguously identified as such during our experiment.[1] Instead, MS analysis of the sample revealed the presence of the starting material with a partially fluorinated silyl ether still attached (assumed to be represented by the grey curve in **Figure 1**).

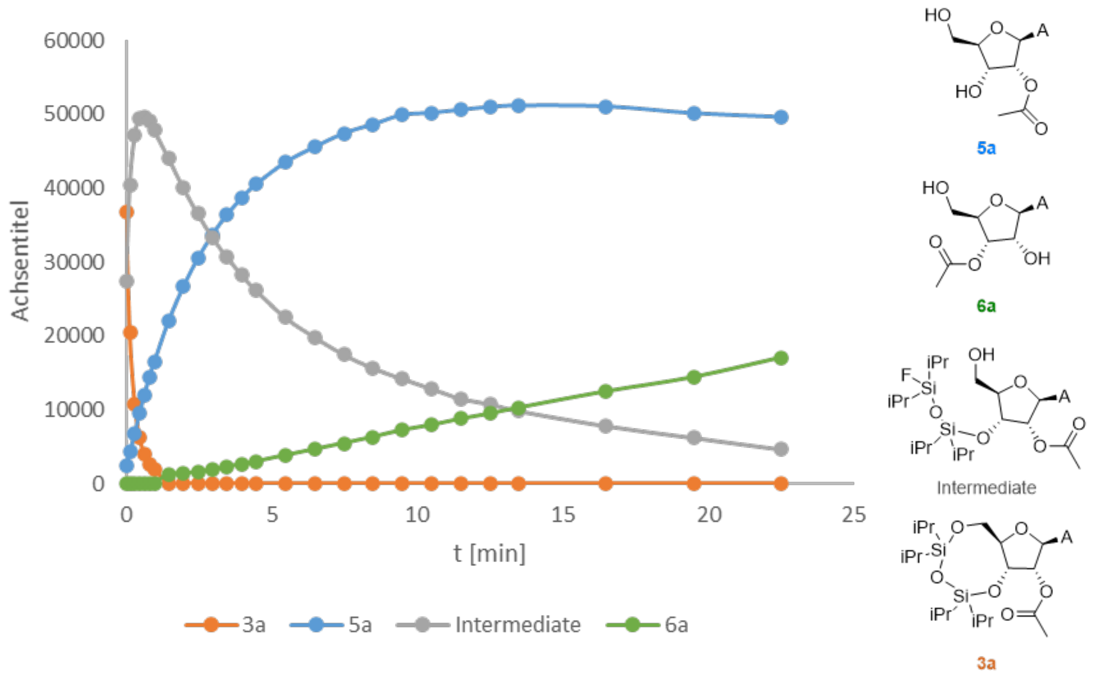

Figure 1. Monitoring of the reaction progress *via* repeated ^{1}H-NMR measurements.

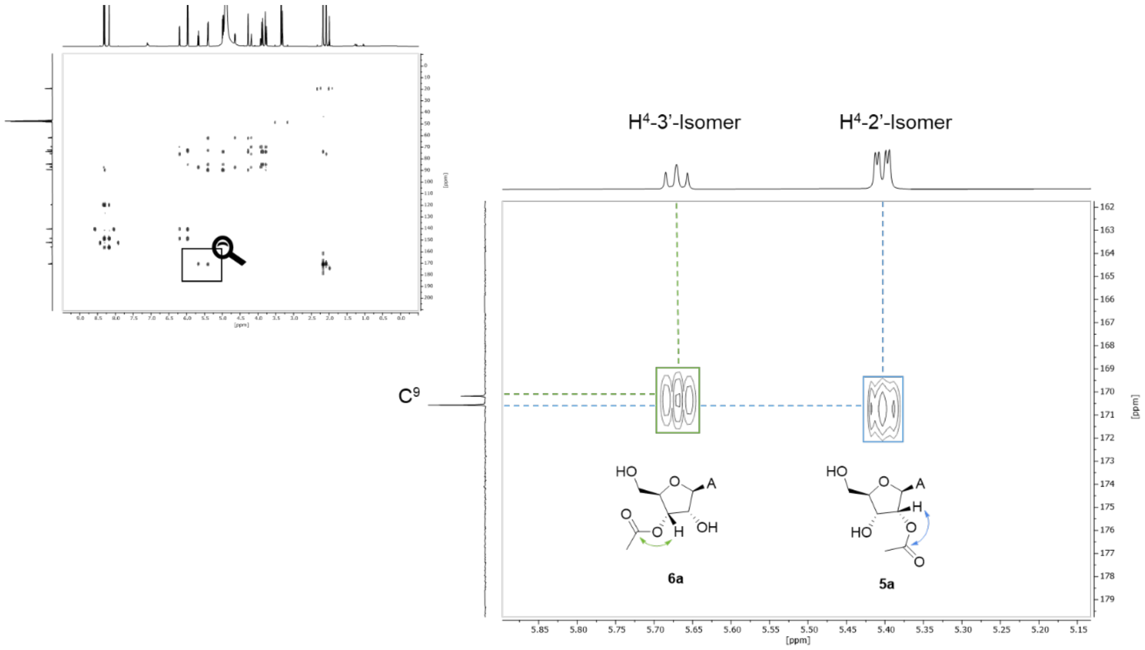

Figure 2. HMBC revealing the position of the acetyl group.

(6a*R*,8*R*,9*R*,9a*R*)-8-(6-amino-9*H*-purin-9-yl)-2,2,4,4-tetraisopropyltetrahydro-6*H*-furo[3,2-*f*][1,3,5,2,4]trioxadisilocin-9-yl (*tert*-butoxycarbonyl)-L-alaninate (**11a**)

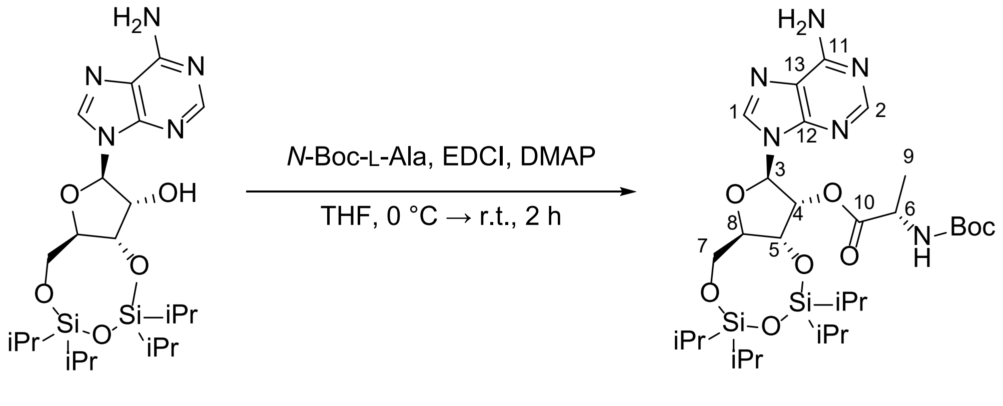

2a **11a**

N-Boc-L-Alanine (223 mg, 1.18 mmol, 1.20 equiv.) was dissolved in dry THF (5 mL). The solution was cooled to 0 °C and EDCI·HCl (282 mg, 1.47 mmol, 1.50 equiv.) was added. After 15 min, **2a** (500 mg, 980 µmol, 1.00 equiv.) and DMAP (240 mg, 1.96 mmol, 2.00 equiv.) were added. The reaction mixture was allowed to warm to r.t. and stirred for 2 h and was subsequently concentrated *in vacuo*. The crude material was purified by column chromatography (cHex/EtOAc 1:1) to give compound **11a** as white foam (668 mg, 980 µmol, 100 %).

HR-MS (ESI): m/z calculated for $C_{30}H_{53}N_6O_8Si_2^+$: 681.3458; found: 681.3466.

^{1}H-NMR (400 MHz, DMSO): δ [ppm] = 8.26 (s, 1H, H^1), 8.04 (s, 1H, H^2), 7.38 – 7.36 (m, 3H, H^{NH2}, H^{NH}), 6.10 (s, 1H, H^3), 5.98 (d, *J* = 6.2 Hz, 1H, H^4), 5.29 – 5.21 (m, 1H, H^5), 4.15 (p, *J* = 7.4 Hz, 1H, H^6), 4.02 (q, *J* = 7.2 Hz, 2H, H^7), 3.97 – 3.87 (m, 1H, H^8), 1.11 – 0.93 (m, 37H, H^{iPr}), 1.33 (d, 3H, H^9).

^{13}C{^{1}H}-NMR (101 MHz, DMSO): δ [ppm] = 172.5 (C^{10}), 156.6 (C^{11}), 155.6 ($C^{Boc\text{-}CO}$), 153.0 (C^2), 149.0 (C^{12}), 140.9 (C^1), 119.7 (C^{13}), 87.0 (C^3), 81.3 (C^8), 78.7 ($C^{Boc\text{-}quart.}$),75.1 (C^4), 69.31 (C^5), 60.2 (C^7), 49.4 (C^6), 28.6 ($C^{Boc\text{-}CH3}$),17.8 – 11.9 (13C, C^9, C^{iPr}).

R_f: 0.57 (EtOAc 100 %).

(2*R*,3*S*,4*R*,5*R*)-5-(6-amino-9*H*-purin-9-yl)-4-hydroxy-2-(hydroxymethyl)tetrahydrofuran-3-yl (*tert*-butoxycarbonyl)-L-alaninate (**12a**)

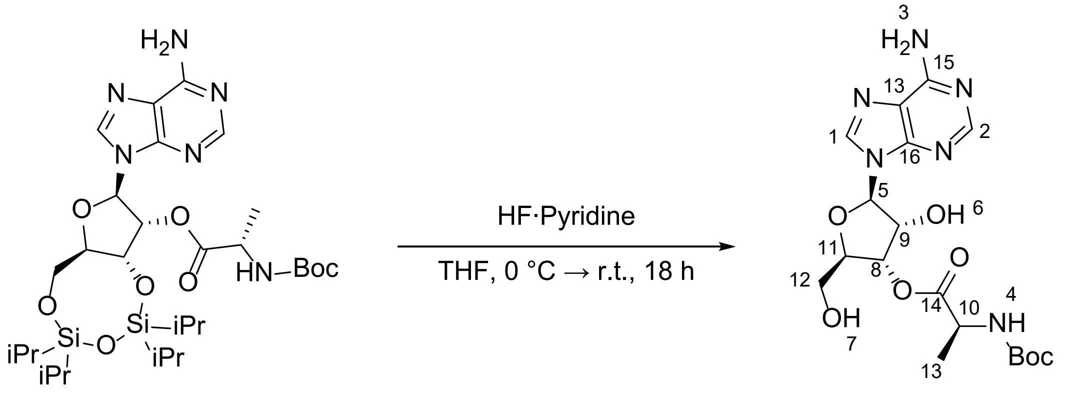

11a **12a**

Compound **11a** (200 mg, 293 µmol, 1.00 equiv.) was dissolved in dry THF (2 mL) and the solution was cooled to 0 °C. HF·pyridine (70 wt.%; 191 µL, 7.34 mmol, 25.0 equiv.) was added dropwise and the mixture was stirred for 18 h at room temperature until TLC confirmed full conversion. Excess fluoride was quenched by adding TMS-OMe (2 mL) and stirring for an additional 30 min. All volatiles were removed *in vacuo* and the residue was purified by flash column chromatography (cHex/EtOAc 1:1 → EtOAc 100 %). Compound **12a** (110 mg, 250 µmol, 85 %) was obtained as a white foam.

HR-MS (ESI): m/z calculated for $C_{18}H_{27}N_6O_7^+$: 439.1936; found: 439.1938.

^{1}H-NMR (400 MHz, DMSO): δ [ppm] = 8.38 (s, 1H, H^1), 8.15 (s, 1H, H^2), 7.41 (s, 2H, H^3), 7.35 (d, *J* = 7.5, 1H, H^4), 5.92 (d, *J* = 7.3, 1H, H^5), 5.76 (d, *J* = 6.5, 1H, H^6), 5.63 (d, *J* = 8.4, 1H, H^7), 5.29 – 5.22 (m, 1H, H^8), 4.89 (q, *J* = 6.2, 1H, H^9), 4.15 (p, *J* = 7.4, 1H, H^{10}), 4.06 (q, *J* = 3.0, 1H, H^{11}), 3.72 – 3.55 (m, 2H, H^{12}), 1.40 (s, 9H, H^{Boc}), 1.32 (d, *J* = 7.4, 3H, H^{13}).

^{13}C{^{1}H}-NMR (101 MHz, DMSO): δ [ppm] = 172.3 (C^{14}), 156.3 (C^{15}), 155.4 ($C^{Boc\text{-}CO}$), 149.2 (C^{16}), 139.7 (C^1), 119.3 (C^{17}), 87.3 (C^5), 78.3 ($C^{Boc\text{-}quart.}$), 73.9 (C^8), 71.9 (C^9), 61.6 (C^{12}), 28.2 ($C^{Boc\text{-}CH3}$).

R_f: 0.44 (EtOAc 100 %).

Silyl Reagent Screening

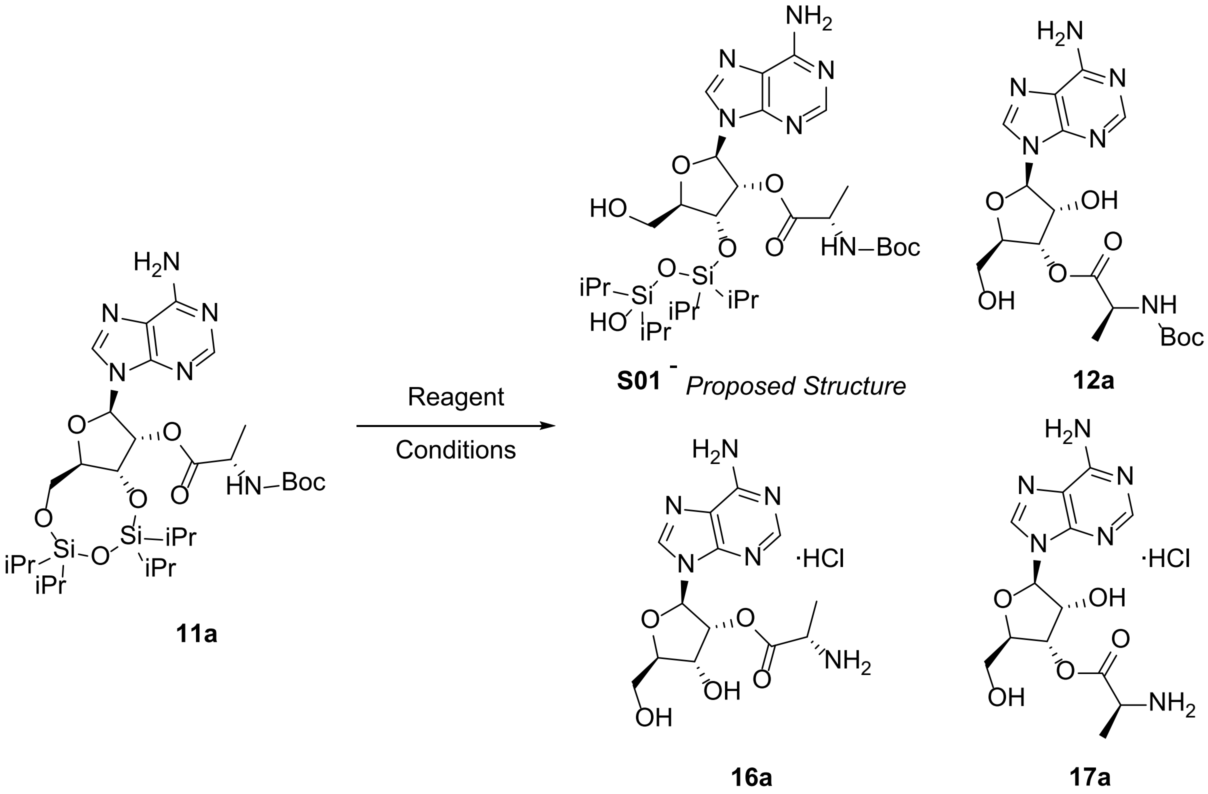

Reagent[1]	Solvent	Equiv.	T [°C]	t [h]	Yield [%]
CsF (s)	NH_3 in MeOH (7 M)	5.00	65	18	Decomposition
HCl (4 M in Et_2O)	1,4-Dioxane	25.0	r.t.	18	3.5 **16a;** 2.5 **17a**; 47 **S01**
3HF·NEt_3	THF	25.0	0 → r.t.	18	82 **12a**
HF·Pyridine (70 wt.% HF)	THF	20.0	0 → r.t.	18	85 **12a**
NaF (s)	THF	25.0	r.t. → 66	48	No Conversion
NH_4F (s)	THF	25.0	r.t. → 66	24	No Conversion
TASF (s) [2]	THF	6.00	0 → r.t.	18	Decomposition
TBAF (1 M, in THF)[3]	THF	1.20	0	0.2	Decomposition
TBAF·H_2O (s)	THF	3.00	0	0.2	Decomposition

[1] All experiments were performed according to modified literature procedures; [2] TASF = Tris(dimethylamino)sulfonium difluorotrimethylsilicate; [3] TBAF = Tetrabutylammonium fluoride.

Compound **11a** (68.3 mg, 0.10 mmol, 1.00 equiv.) was dissolved in a solvent listed in the table above (0.5 mL). A reagent for deprotection (view table for stoichiometry) was added at a suitable temperature (0 °C or r.t.) and reaction progress was monitored by TLC and/or HRMS. When no conversion was observed within 24 h the temperature was increased. The reaction was stopped either after full consumption of the starting material or upon full conversion according to MS analysis. Excess fluoride was quenched with TMS-OMe (0.2 mL) and after 30 min all volatiles were removed *in vacuo*. Purification *via* flash column chromatography yielded **12a**, **16a**, **16b** or **S01** in the yields given in the table above.

For analytical data of the products obtained by successful reactions with HCl or HF·pyridine view the respective pages of the mentioned compounds.

(2*R*,3*S*,4*R*,5*R*)-5-(6-amino-9*H*-purin-9-yl)-4-hydroxy-2-(hydroxymethyl)tetrahydrofuran-3-yl L-alaninate hydrochloride (**17a**)

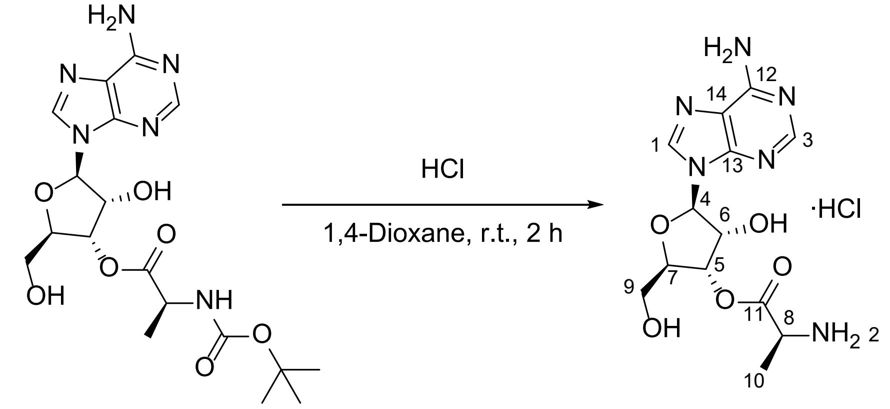

12a **17a**

Ester **12a** (90.0 mg, 205 µmol, 1.00 equiv.) was dissolved in 1,4-dioxane (2 mL) and HCl in 1,4-dioxane (4 M, 1.00 mL) was added dropwise to the solution. The mixture was stirred at r.t. for 2 h until a white suspension had formed. The white residue was filtered and washed with Et_2O. Compound **17a** (53.2 mg, 142 µmol, 69 %) was obtained as a hydrochloride in the form of white foam.

HR-MS (ESI): m/z calculated for $C_{13}H_{19}N_6O_5^+$: 339.1411; found: 339.1413.

^{1}H-NMR (400 MHz, DMSO): δ [ppm] = 8.79 (s, 1H, H^1), 8.76 – 8.70 (m, 3H, H^2), 8.57 (s, 1H, H^3), 6.11 (d, *J* = 6.8 Hz, 1H, H^4), 5.39 (dd, *J* = 5.2, 2.4 Hz, 1H, H^5), 4.87 (dd, *J* = 6.8, 5.2 Hz, 1H, H^6), 4.26 (q, *J* = 3.6 Hz, 1H, H^7), 4.23 – 4.13 (m, 1H, H^8), 3.66 (qd, *J* = 12.1, 3.9 Hz, 2H, H^9), 1.50 (d, *J* = 7.2 Hz, 3H, H^{10}).

^{13}C{^{1}H}-NMR (101 MHz, DMSO): δ [ppm] = 168.9 (C^{11}), 150.5 (C^{12}), 148.6 (C^{13}), 145.5 (C^3), 141.9 (C^1), 118.6 (C^{14}), 86.8 (C^4), 83.3 (C^7), 74.6 (C^5), 73.0 (C^6), 60.9 (C^9), 48.1 (C^8), 15.8 (C^{10}).

(2*R*,3*R*,4*R*,5*R*)-2-(6-amino-9*H*-purin-9-yl)-4-hydroxy-5-(hydroxymethyl)tetrahydrofuran-3-yl L-alaninate (**16a**)

(2*R*,3*R*,4*R*,5*R*)-2-(6-amino-9*H*-purin-9-yl)-4-hydroxy-2-(hydroxymethyl)tetrahydrofuran-3-yl L-alaninate (**17a**)

(2*R*,3*R*,4*R*,5*R*)-2-(6-amino-9*H*-purin-9-yl)-4-((3-hydroxy-1,1,3,3-tetraisopropyldisiloxaneyl)oxy)-5-(hydroxymethyl)tetrahydrofuran-3-yl L-alaninate hydrochloride (**S01**)

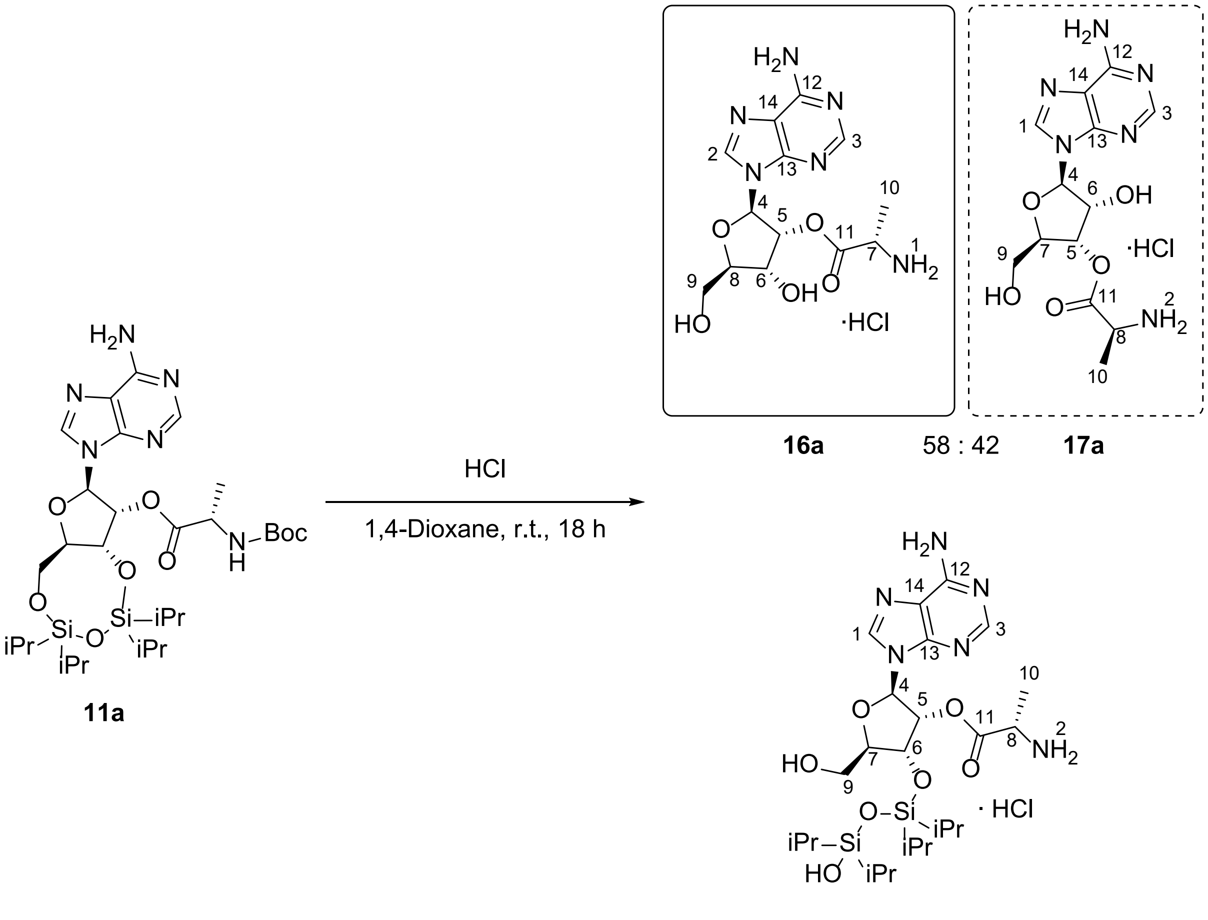

Compound **11a** (210 mg, 308 µmol, 1.00 equiv.) was dissolved in 1,4-dioxane (6 mL). HCl in 1,4-dioxane (4 M, 2.00 mL) was added and the mixture was stirred for 18 h. After TLC analysis indicated full conversion, all volatiles were removed under reduced pressure and the residue was purified *via* flash column chromatography (EtOAc 100 % → EtOAc/MeOH 1:1). Fully deprotected compounds **16a** and **17a** (7.3 mg, 18.5 µmol, 6 %) were obtained as a mixture of regioisomers. Furthermore, a Boc-deprotected

alanine-adenosine **S01** with a partially hydrolysed silyl ether also could be isolated (91.9 mg, 145 µmol, 47 %).

2'-Isomer **16a**:

HR-MS (ESI): m/z calculated for $C_{13}H_{19}N_6O_5^+$: 339.1411; found: 339.1412.

^{1}H-NMR (400 MHz, DMSO): δ [ppm] = 8.75 (s, 2H, H^1), 8.69 (s, 1H, H^2), 8.49 (s, 1H, H^3), 6.29 (d, *J* = 4.3 Hz, 1H, H^4), 5.68 (t, *J* = 4.7 Hz, 1H, H^5), 4.53 (t, *J* = 5.2 Hz, 1H, H^6), 4.20 (m, 1H, H^7), 4.10 – 4.04 (m, 1H, H^8), 3.80 – 3.57 (m, 2H, H^9), 1.45 (d, *J* = 7.2 Hz, 3H, H^{10}).

^{13}C{^{1}H}-NMR (101 MHz, DMSO): δ [ppm] = 168.9 (C^{11}), 151.6 (C^{12}), 148.2 (C^{13}), 146.9 (C^3), 141.7 (C^2), 118.8 (C^{14}), 85.5 (2C, $C^{4,8}$), 76.9 (C^5), 68.4 (C^6), 60.4 (C^9), 47.9 (C^7), 15.7 (C^{10}).

R_f: 0.11 (EtOAc/MeOH 70:30).

3'-Isomer **17a**:

HR-MS (ESI): m/z calculated for $C_{13}H_{19}N_6O_5^+$: 339.1411; found: 339.1412.

^{1}H-NMR (400 MHz, DMSO): δ [ppm] = 8.71 (s, 1H, H^1), 8.68 (s, 2H, H^2), 8.50 (s, 1H, H^3), 6.10 (d, *J* = 6.9 Hz, 1H, H^4), 5.39 (dd, *J* = 5.2, 2.3 Hz, 1H, H^5), 4.88 (dd, *J* = 6.9, 5.2 Hz, 1H, H^6), 4.25 (q, *J* = 3.5 Hz, 1H, H^7), 4.11 (d, *J* = 8.5 Hz, 1H, H^8), 3.80 – 3.57 (m, 2H, H^9), 1.51 (d, *J* = 7.2 Hz, 3H, H^{10}).

^{13}C{^{1}H}-NMR (101 MHz, DMSO): δ [ppm] = 168.9 (C^{11}), 151.7 (C^{12}), 148.7 (C^{13}), 146.7 (C^3), 141.5 (C^1), 118.7 (C^{14}), 86.8 (C^4), 83.2 (C^8), 74.7 (C^5), 72.8 (C^6), 61.0 (C^9), 48.1 (C^8), 15.8 (C^{10}).

R_f: 0.11 (EtOAc/MeOH 70:30).

Partially hydrolysed product **S01**

HR-MS (ESI): m/z calculated for $C_{30}H_{55}N_6O_9Si_2^+$: 699.3564; found: 699.3568.

^{1}H-NMR (400 MHz, DMSO): δ [ppm] = 8.66 (s, 1H, H^1), 8.63 (d, *J* = 5.4 Hz, 3H, H^2), 8.45 (d, *J* = 1.3 Hz, 1H, H^3), 6.30 (d, *J* = 4.6 Hz, 1H, H^4), 5.79 (t, *J* = 4.6 Hz, 1H, H^5), 4.88 (t, *J* = 4.6 Hz, 1H, H^6), 4.23 (q, *J* = 3.4 Hz, 1H, H^7), 4.08 (m, 1H, H^8), 3.81 (dd, *J* = 12.5, 2.8 Hz, 1H, H^{9a}), 3.67 (dd, *J* = 12.3, 3.4 Hz, 1H, H^{9b}), 1.47 (dd, *J* = 17.6, 7.2 Hz, 4H, H^{10}), 1.10 – 0.88 (m, 28H, H^{iPr}).

^{13}C{^{1}H}-NMR (101 MHz, DMSO): δ [ppm] = 169.4 (C^{11}), 151.9 (C^{12}), 148.2 (C^{13}), 147.9 (C^3), 141.4 (C^2), 118.8 (C^{14}), 85.8 (C^7), 85.6 (C^4), 76.6 (C^5), 69.9 (C^6), 60.2 (C^9), 47.8 (C^8), 17.3 – 17.0 (8C, $C^{iPr\text{-}CH3}$), 16.1 (C^{10}), 13.1 - 12.6 (C4, $C^{iPr\text{-}CH}$).

R_f: 0.37 (EtOAc/MeOH 70:30).

(2*R*,3*R*,4*R*,5*R*)-2-(6-amino-9*H*-purin-9-yl)-4-hydroxy-5-(hydroxymethyl)tetrahydrofuran-3-yl L-alaninate (**16a**)

(2*R*,3*R*,4*R*,5*R*)-2-(6-amino-9*H*-purin-9-yl)-4-hydroxy-2-(hydroxymethyl)tetrahydrofuran-3-yl L-alaninate (**17a**)

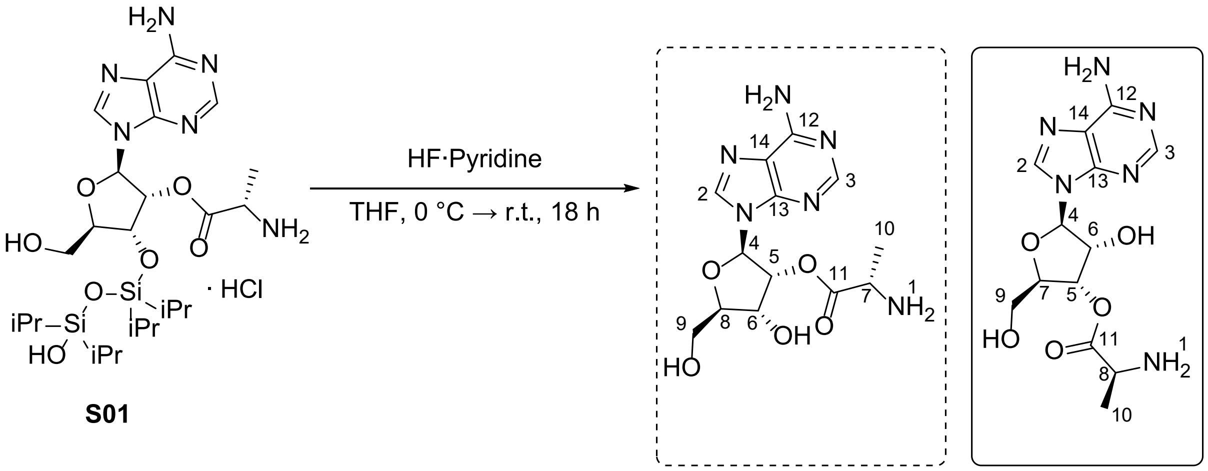

Compound **S01** (52.3 mg, 0.082 mmol, 1.00 equiv.) was dissolved in dry THF (2 mL) and the solution was cooled to 0 °C. HF·pyridine (70 wt.%; 22.0 µL, 0.823 mmol, 10.0 equiv.) was added dropwise and the mixture was stirred for 18 h. Excess fluoride was quenched by adding TMS-OMe (2 mL) and stirring for an additional 30 min. All volatiles were removed *in vacuo* and the residue was purified by flash column chromatography (EtOAc 100 % →EtOAc/MeOH 50:50). A mixture of regioisomers **16a** and **17a** (26.6 mg, 0.079 µmol, 96 %, sum of the two regioisomers) were obtained as a white solid.

HR-MS (ESI): m/z calculated for $C_{13}H_{19}N_6O_5^+$: 339.1411; found: 339.1412.

R_f: 0.11 (No separation between isomers observed; EtOAc/MeOH 70:30).

2'-Isomer **16a**:

^{1}H-NMR (400 MHz, DMSO): δ [ppm] = 8.67 (s, 3H, H^1), 8.60 (s, 1H, H^2), 8.38 (s, 1H, H^3), 6.27 (d, *J* = 4.5 Hz, 1H, H^4), 5.70 (t, *J* = 4.9 Hz, 1H, H^5), 4.53 (t, *J* = 5.1 Hz, 1H, H^6), 4.19 (t, *J* = 6.2 Hz, 1H, H^7), 4.07 (dt, *J* = 5.1, 3.5 Hz, 1H, H^8), 3.79 – 3.56 (m, 2H, H^9), 1.45 (d, *J* = 7.2 Hz, 3H, H^{10}).

$^{13}C\{^{1}H\}$-NMR (101 MHz, DMSO): δ [ppm] = 168.9 (C^{11}), 153.0 (C^{12}), 148.9 (C^{13}), 148.4 (C^{3}), 141.0 (C^{2}), 119.0 (C^{14}), 85.6 (C^{8}), 85.5 (C^{4}), 76.7 (C^{5}), 68.5 (C^{6}), 61.1 (C^{9}), 47.9 (C^{7}), 15.8 (C^{10}).

3'-Isomer **17a**:

^{1}H-NMR (400 MHz, DMSO): δ [ppm] = 8.77 – 8.71 (m, 3H, H^{1}), 8.62 (s, 1H, H^{2}), 8.40 (s, 1H, H^{3}), 6.08 (d, *J* = 7.0 Hz, 1H, H^{4}), 5.39 (dd, *J* = 5.2, 2.2 Hz, 1H, H^{5}), 4.90 (dd, *J* = 7.0, 5.2 Hz, 1H, H^{6}), 4.23 (dd, *J* = 3.9, 2.3 Hz, 1H, H^{7}), 4.19 (t, *J* = 6.2 Hz, 1H, H^{8}), 3.79 – 3.56 (m, 2H, H^{9}), 1.51 (d, *J* = 7.2 Hz, 3H, H^{10}).

$^{13}C\{^{1}H\}$-NMR (101 MHz, DMSO): δ [ppm] = 168.9 (C^{11}), 153.0 (C^{12}), 148.9 (C^{13}), 148.4 (C^{3}), 141.0 (C^{2}), 119.0 (C^{14}), 86.9 (C^{4}), 83.2 (C^{7}), 74.9 (C^{5}), 72.6 (C^{6}), 61.1 (C^{9}), 47.9 (C^{8}), 15.8 (C^{10}).

(6a*R*,8*R*,9*R*,9a*R*)-8-(6-amino-9*H*-purin-9-yl)-2,2,4,4-tetraisopropyltetrahydro-6*H*-furo[3,2-*f*][1,3,5,2,4]trioxadisilocin-9-yl acetyl-L-alaninate (**13a**)

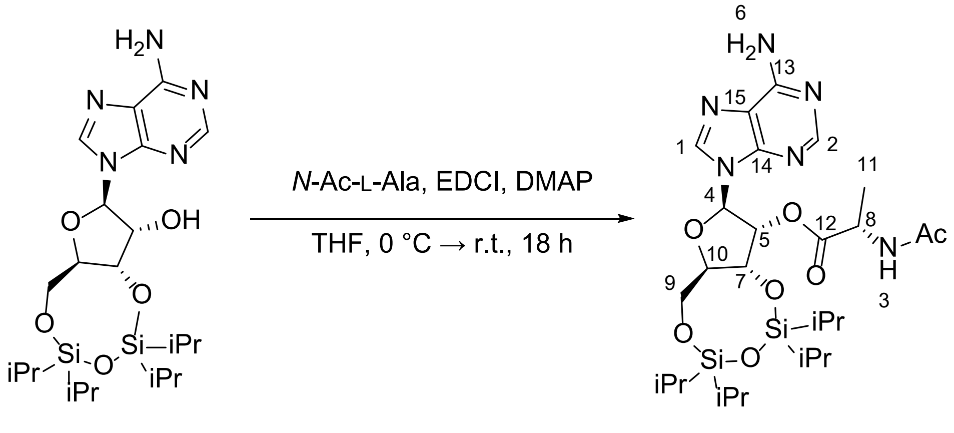

2a **13a**

N-Ac-L-Alanine (257 mg, 1.96 mmol, 1.00 equiv.) was dissolved in dry THF (21 mL) and cooled to 0 °C. EDCI·HCl (414 mg, 2.16 mmol, 1.10 equiv.) was added and the white suspension was stirred for 15 min. TIPDS-Adenosine **2a** (1.00 g, 1.96 mmol, 1.00 equiv.) and DMAP (360 mg, 2.94 mmol, 1.50 equiv.) were added and the reaction was warmed to r.t. and was stirred overnight. After TLC indicated conversion, removal of the solvents *in vacuo* and purification by column chromatography (EtOAc/MeOH 98:2) yielded **13a** (1.06 g, 1.71 mmol, 87 %) as a white solid.

HR-MS (ESI): m/z calculated for $C_{27}H_{47}N_6O_7Si_2^+$: 623.3039; found: 623.3040.

^{1}H-NMR (400 MHz, $CDCl_3$): δ [ppm] = 8.28 (s, 1H, H^1), 7.97 (s, 1H, H^2), 6.07 (d, J = 6.7 Hz, 1H, H^3), 6.01 (s, 1H, H^4), 5.90 (d, J = 5.2 Hz, 1H, H^5), 5.54 (d, J = 6.6 Hz, 2H, H^6), 5.19 (dd, J = 8.9, 5.2 Hz, 1H, H^7), 4.76 (p, J = 7.2 Hz, 1H, H^8), 4.23 – 4.13 (m, 1H, H^{9a}), 4.04 (d, J = 1.6 Hz, 1H, H^{10}), 4.01 (q, J = 2.8 Hz, 1H, H^{9b}), 2.02 (s, 3H, $H^{Ac\text{-}CH3}$), 1.48 (d, J = 7.1 Hz, 3H, H^{11}), 1.17 – 0.99 (m, 28H, H^{iPr}).

^{13}C{^{1}H}-NMR (101 MHz, $CDCl_3$): δ [ppm] = 172.2 (C^{12}), 169.7 ($C^{Ac\text{-}CO}$), 155.5 (C^{13}), 153.4 (C^1), 149.3 (C^{14}), 139.7 (C^2), 120.5 (C^{15}), 87.6 (C^4), 81.9 (C^{10}), 76.4 (C^5), 68.8 (C^7), 60.1 (C^9), 48.2 (C^8), 23.3 ($C^{Ac\text{-}CH3}$), 19.2 (C^{11}), 17.6 - 16.9 (8C, $C^{iPr\text{-}CH3}$), 13.5 - 12.6 (4C, $C^{iPr\text{-}CH}$).

R_f: 0.43 (EtOAc/MeOH 98:2).

(2*R*,3*S*,4*R*,5*R*)-5-(6-amino-9*H*-purin-9-yl)-4-hydroxy-2-(hydroxymethyl)tetrahydrofuran-3-yl acetyl-L-alaninate (**S02**)

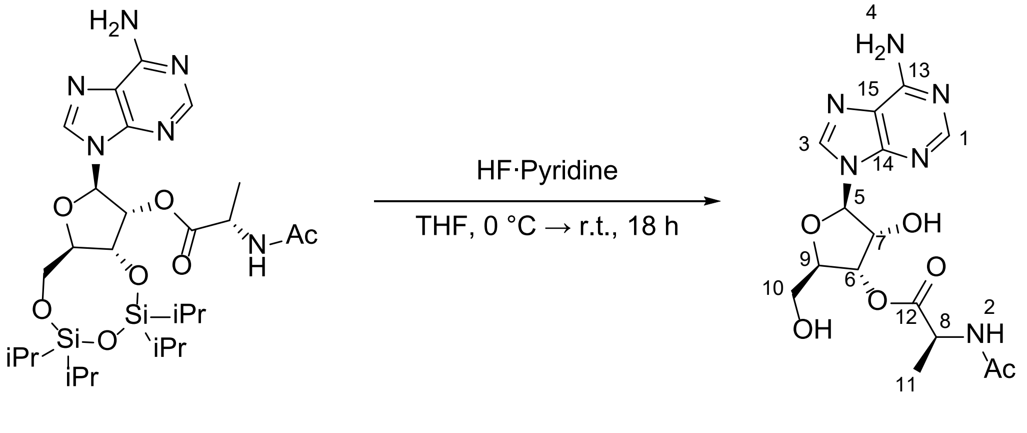

13a **S02**

Silyl-Ac-Ala-Adenosine **13a** (249 mg, 400 µmol, 1.00 equiv.) was dissolved in THF (2.4 mL) and the solution was cooled to 0 °C. HF·pyridine (70 wt.%; 250 µL, 8.00 mmol, 20.0 equiv.) was added. The reaction was allowed to warm to room temperature and was stirred overnight. Excess fluoride was quenched *via* addition of TMS-OMe (3.0 mL) and further stirring for 30 min. Purification by column chromatography (EtOAc/MeOH 95:5) yielded **S02** (106 mg, 280 µmol, 70 %) as a white solid.

HR-MS (ESI): m/z calculated for $C_{15}H_{21}N_6O_6^+$: 381.1517; found: 381.1517.

^{1}H-NMR (400 MHz, DMSO-*d*$_6$): δ [ppm] = 8.39 (s, 1H, H^1), 8.37 (s, 1H, H^2), 8.16 (s, 1H, H^3), 7.43 (s, 2H, H^4), 5.91 (d, *J* = 7.3 Hz, 1H, H^5), 5.28 (dd, *J* = 5.3, 1.9 Hz, 1H, H^6), 4.92 (td, *J* = 6.9, 5.3 Hz, 1H, H^7), 4.41 (p, *J* = 7.3 Hz, 1H, H^8), 4.10 (td, *J* = 3.3, 1.8 Hz, 1H, H^9), 3.78 – 3.55 (m, 2H, H^{10}), 1.88 (s, 3H, $H^{Ac\text{-}CH3}$), 1.35 (d, *J* = 7.3 Hz, 3H, H^{11}).

^{13}C{^{1}H}-NMR (101 MHz, CDCl$_3$): δ [ppm] = 172.0 (C^{12}), 169.4 ($C^{Ac\text{-}CO}$), 156.3 (C^{13}), 152.6 (C^3), 149.2 (C^{14}), 139.9 (C^1), 119.4 (C^{15}), 87.6 (C^5), 83.7 (C^9), 74.1 (C^6),71.8 (C^7) 61.7 (C^{10}), 47.8 (C^8), 22.4 ($C^{Ac\text{-}CH3}$), 17.3 (C^{11}).

R$_f$: 0.16 (EtOAc/MeOH 95:5).

(6a*R*,8*R*,9*R*,9a*R*)-8-(6-amino-9*H*-purin-9-yl)-2,2,4,4-tetraisopropyltetrahydro-6*H*-furo[3,2-*f*][1,3,5,2,4]trioxadisilocin-9-yl (*tert*-butoxycarbonyl)-L-phenylalaninate (**15a**)

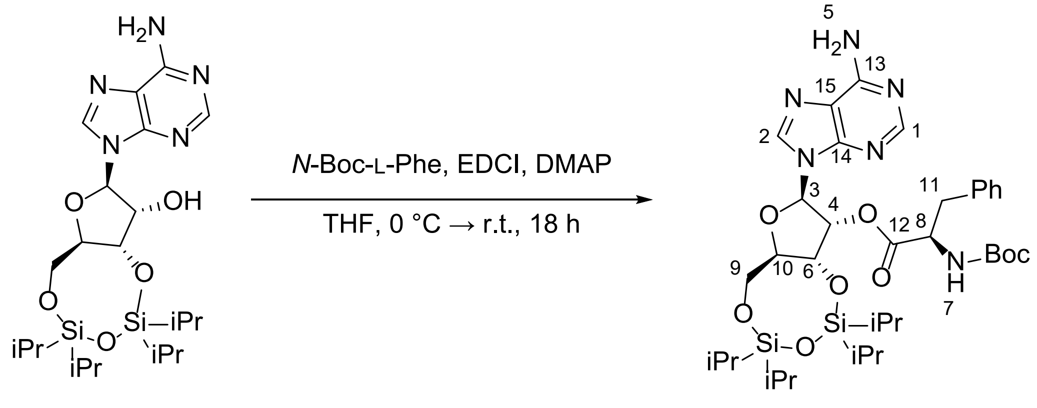

2a **15a**

N-Boc-L-Phenylalanine (1.33 g, 5.00 mmol, 1.00 equiv.) was dissolved in dry THF (54 mL). The solution was cooled to 0 °C and EDCI·HCl (1.05 g, 5.50 mmol, 1.10 equiv.) was added. After 15 min, **2a** (2.55 g, 5.00 mmol, 1.00 equiv.) and DMAP (920 mg, 7.50 mmol, 1.50 equiv.) were added. The reaction mixture was allowed to warm to r.t., stirred overnight and subsequently concentrated *in vacuo*. The crude material was purified by column chromatography (cHex/EtOAc 1:2) to give compound **15a** as white foam (3.22 g, 4.25 mmol, 85 %).

HR-MS (ESI): m/z calculated for $C_{36}H_{57}N_6O_8Si_2^+$: 757.3771; found: 757.3783.

^{1}H-NMR (400 MHz, $CDCl_3$): δ [ppm] = 8.21 (s, 1H, H^1), 7.85 (s, 1H, H^2), 7.29 – 7.07 (m, 5H, Ph), 5.84 (s, 1H, H^3), 5.81 (d, *J* = 5.3 Hz, 1H, H^4), 5.65 (s, 2H, H^5), 5.24 (dd, *J* = 8.9, 5.3 Hz, 1H, H^6), 4.95 (d, *J* = 8.4 Hz, 1H, H^7), 4.67 (td, *J* = 8.1, 4.8 Hz, 1H, H^8), 4.19 – 4.03 (m, 1H, H^{9a}), 4.00 – 3.88 (m, 2H, $H^{9b,10}$), 3.21 (dd, *J* = 14.1, 5.0 Hz, 1H, H^{11a}), 2.91 (dd, *J* = 14.1, 7.8 Hz, 1H, H^{11b}), 1.31 (s, 9H, H^{Boc}), 1.10 – 0.92 (m, 29H, H^{iPr}).

^{13}C{^{1}H}-NMR (101 MHz, $CDCl_3$): δ [ppm] = 171.1 (C^{12}), 155.6 (C^{13}), 155.2 ($C^{Boc\text{-}CO}$), 153.3 (C^1), 149.3 (C^{14}), 139.9 (C^2), 136.2 (C^{Ph}), 129.4 (C^{Ph}), 128.7 (C^{Ph}), 127.2 (C^{Ph}), 120.5 (C^{15}), 87.8 (C^3), 81.9 (C^{10}), 80.2 ($C^{Boc\text{-}quart.}$), 76.6 (C^4), 68.9 (C^6), 60.2 (C^9), 54.5 (C^8), 38.8 (C^{11}), 28.4 ($C^{Boc\text{-}CH3}$), 17.6 - 17.0 (8C, $C^{iPr\text{-}CH3}$), 13.5 - 12.7 (4C, $C^{iPr\text{-}CH}$).

R_f: 0.28 (cHEx/EtOAc 1:2).

(2*R*,3*S*,4*R*,5*R*)-5-(6-amino-9*H*-purin-9-yl)-4-hydroxy-2-(hydroxymethyl)tetrahydrofuran-3-yl (*tert*-butoxycarbonyl)-L-phenylalaninate (**S03**)

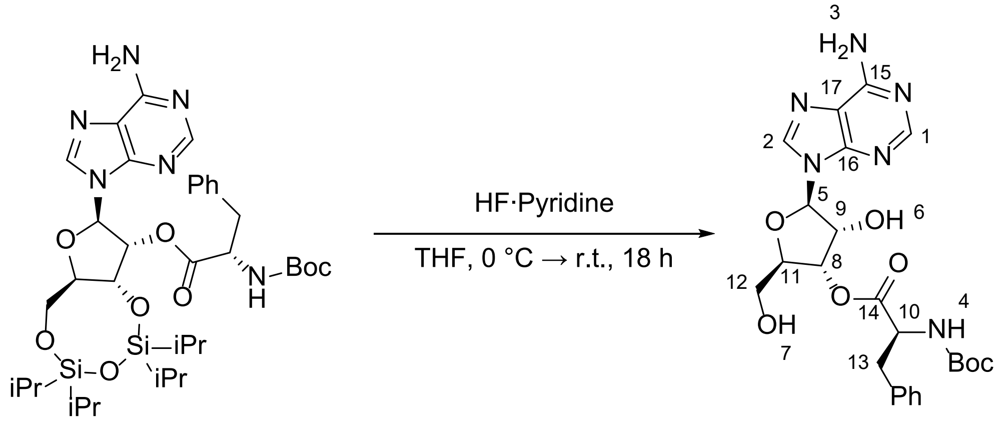

15a **S03**

15a (269 mg, 355 µmol, 1.00 equiv.) was dissolved in THF (2.1 mL). The reaction was cooled down to 0 °C. HF·pyridine (70 wt.%; 180 µL, 7.11 mmol, 20.0 equiv.) was added. The reaction was allowed to warm up to r.t. and stirred for 18 h before it was quenched with MeO-TMS (3.0 mL). It was concentrated *in vacuo* and purified by column chromatography (EtOAc 100 %) to give compound **S03** as white foam (171 mg, 334 µmol, 94 %).

HR-MS (ESI): m/z calculated for $C_{24}H_{31}N_6O_7^+$: 515.2249; found: 515.2249.

^{1}H-NMR (400 MHz, DMSO): δ [ppm] = 8.37 (d, *J* = 3.9 Hz, 1H, H^1), 8.15 (s, 1H, H^2), 7.44 – 7.35 (m, 3H, $H^{3,4}$), 7.35 – 7.26 (m, 3H, H^{Ph}), 7.21 (dtt, *J* = 17.7, 7.3, 3.8 Hz, 2H, H^{Ph}), 5.91 (d, *J* = 7.4 Hz, 1H, H^5), 5.76 (d, *J* = 6.9 Hz, 1H, H^6), 5.64 (dd, *J* = 7.4, 4.6 Hz, 1H, H^7), 5.26 (dd, *J* = 5.3, 1.8 Hz, 1H, H^8), 4.90 (td, *J* = 7.2, 5.3 Hz, 1H, H^9), 4.33 (ddd, *J* = 9.9, 8.2, 5.2 Hz, 1H, H^{10}), 3.97 (p, *J* = 2.8 Hz, 1H, H^{11}), 3.66 (dt, *J* = 12.2, 4.1 Hz, 1H, H^{12a}), 3.57 (ddd, *J* = 11.7, 7.5, 3.3 Hz, 1H, H^{12b}), 3.16 – 3.02 (m, 1H, H^{13a}), 2.94 (dd, *J* = 13.8, 10.0 Hz, 1H, H^{13b}), 1.34 (s, 9H, H^{Boc}).

^{13}C{^{1}H}-NMR (101 MHz, DMSO): δ [ppm] = 171.3 (C^{14}), 156.3 (C^{15}), 155.5 ($C^{Boc\text{-}CO}$), 152.5 (C^2), 149.2 (C^{16}), 139.7 (C^1), 137.6 ($C^{Ph\text{-}quart.}$), 129.2 (C^{Ph}), 128.3 (C^{Ph}), 126.5 (C^{Ph}), 119.3 (C^{17}), 87.2 (C^5), 83.7 (C^{11}), 78.4 ($C^{Boc\text{-}quart.}$), 74.1 (C^8), 71.9 (C^9), 61.6 (C^{12}), 55.5 (C^{10}), 36.8 (C^{13}), 28.1 ($C^{Boc\text{-}CH3}$).

R_f: 0.08 (EtOAc 100 %).

(6a*R*,8*R*,9*R*,9a*R*)-8-(6-amino-9*H*-purin-9-yl)-2,2,4,4-tetraisopropyltetrahydro-6*H*-furo[3,2-*f*][1,3,5,2,4]trioxadisilocin-9-yl (*tert*-butoxycarbonyl)-L-valinate (**14a**)

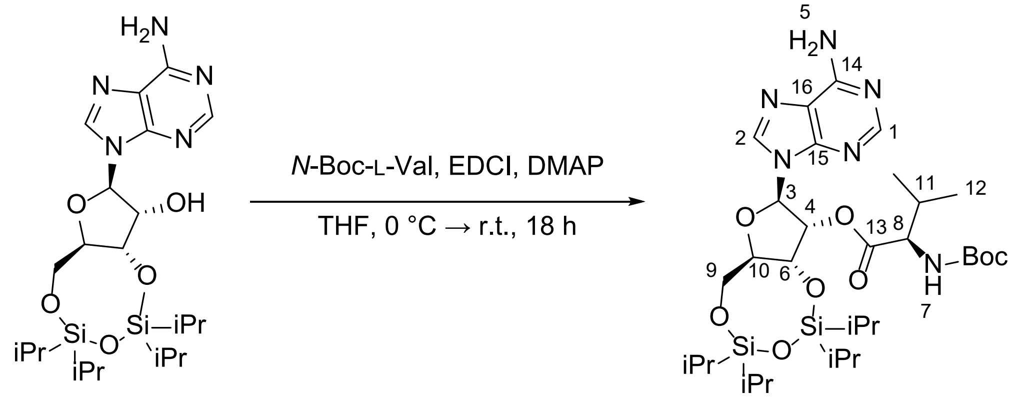

2a **14a**

N-Boc-L-Valine (1.09 g, 5.00 mmol, 1.00 equiv.) was dissolved in dry THF (54 mL). The solution was cooled to 0 °C and EDCI·HCl (1.05 g, 5.50 mmol, 1.10 equiv.) was added. After 15 min, **2a** (2.55 g, 5.00 mmol, 1.00 equiv.) and DMAP (920 mg, 7.50 mmol, 1.50 equiv.) were added. The reaction mixture was allowed to warm to r.t. and stirred overnight before subsequently concentrated *in vacuo*. The crude material was purified by column chromatography (cHex/EtOAc 1:2) to give compound **14a** as white foam (2.76 g, 3.90 mmol, 78 %).

HR-MS (ESI): m/z calculated for $C_{32}H_{57}N_6O_8Si_2^+$: 709.3771; found: 709.3773.

^{1}H-NMR (400 MHz, CDCl$_3$): δ [ppm] = 8.28 (s, 1H, H^1), 7.99 (s, 1H, H^2), 5.99 (s, 1H, H^3), 5.88 (d, *J* = 5.1 Hz, 1H, H^4), 5.59 (s, 2H, H^5), 5.19 (dd, *J* = 9.1, 5.1 Hz, 1H, H^6), 5.12 (d, *J* = 9.0 Hz, 1H, H^7), 4.39 (dd, *J* = 9.0, 4.4 Hz, 1H, H^8), 4.20 (d, *J* = 13.0 Hz, 1H, H^{9a}), 4.09 – 3.97 (m, 2H, $H^{9b,10}$), 2.20 (ddd, *J* = 13.0, 6.5, 3.9 Hz, 1H, H^{11}), 1.44 (s, 9H, H^{Boc}), 1.10 – 1.00 (m, 34H, $H^{12,\ iPr}$).

^{13}C{^{1}H}-NMR (101 MHz, CDCl$_3$): δ [ppm] = 171.3 (C^{13}), 155.6 (Boc), 155.3 (C^{14}), 153.1 (C^1), 149.2 (C^{15}), 139.7 (C^2), 120.3 (C^{16}), 87.9 (C^3), 81.7 (C^{10}), 79.9 (Boc), 76.1 (C^4), 68.2 (C^6), 59.6 (C^9), 58.6 (C^8), 31.6 (C^{11}), 28.3 (Boc), 17.7 - 16.7 (9C, $C^{12/iPr\text{-}CH3}$), 13.4 - 12.3 (4C, $C^{iPr\text{-}CH}$).

R$_f$: 0.21 (cHEx/EtOAc 1:2).

(2*R*,3*S*,4*R*,5*R*)-5-(6-amino-9*H*-purin-9-yl)-4-hydroxy-2-(hydroxymethyl)tetrahydrofuran-3-yl (*tert*-butoxycarbonyl)-L-valinate (**S04**)

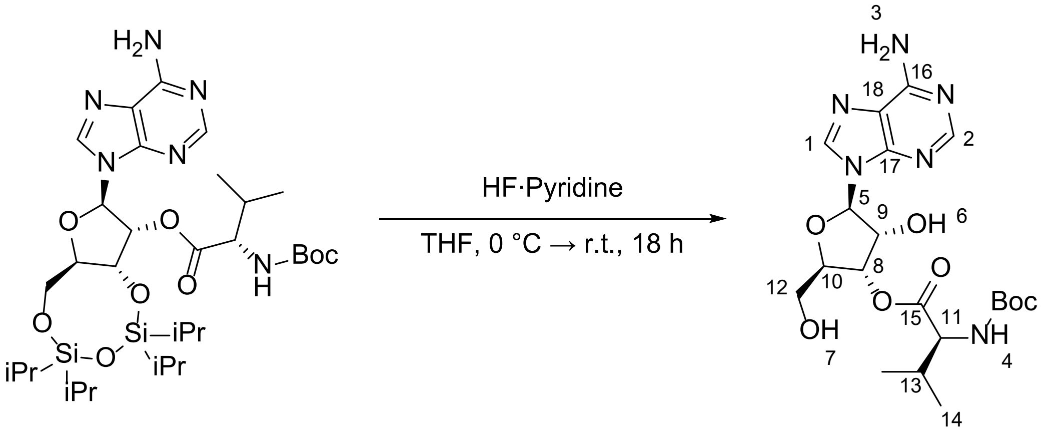

14a **S04**

14a (268 mg, 380 µmol, 1.00 equiv.) was dissolved in THF (2.3 mL) and cooled down to 0 °C. HF·pyridine (70 wt.%; 200 µL, 7.55 mmol, 20.00 equiv.) was added and the reaction was allowed to warm up to r.t. overnight. After quenching with MeO-TMS (3.0 mL) it was concentrated *in vacuo* and purified by column chromatography (EtOAc 100 %) to give compound **S04** as white foam (170 mg, 367 µmol, 97 %).

HR-MS (ESI): m/z calculated for $C_{20}H_{31}N_6O_7^+$: 467.2249; found: 467.2252.

^{1}H-NMR (400 MHz, DMSO): δ [ppm] = 8.38 (s, 1H, H^1), 8.15 (s, 1H, H^2), 7.38 (s, 2H, H^3), 7.23 (d, J = 8.4 Hz, 1H, H^4), 5.93 (d, J = 7.4 Hz, 1H, H^5), 5.75 (d, J = 6.8 Hz, 1H, H^6), 5.61 (dd, J = 7.2, 4.7 Hz, 1H, H^7), 5.27 (dd, J = 5.2, 1.8 Hz, 1H, H^8), 4.90 (q, J = 6.6 Hz, 1H, H^9), 4.03 (ddd, J = 14.2, 7.3, 2.2 Hz, 2H, $H^{10,11),}$ 3.75 – 3.54 (m, 2H, H^{12}), 2.13 (p, J = 6.7 Hz, 1H, H13), 1.38 (d, J = 25.5 Hz, 9H, H^{Boc}), 0.93 (t, J = 7.0 Hz, 6H, H^{14}).

^{13}C{^{1}H}-NMR (101 MHz, DMSO): δ [ppm] = 171.0 (C^{15}), 156.2 (C^{16}), 155.9 ($C^{Boc\text{-}CO}$), 152.5 (C^2), 149.2 (C^{17}), 139.6 (C^1), 119.2 (C^{18}), 87.2 (C^5), 83.8 (C^{10}), 78.3 ($C^{Boc\text{-}quart.}$), 73.9 (C^8), 71.9 (C^9), 61.5 (C^{12}), 59.5 (C^{11}), 29.8 (C^{13}), 28.2 ($C^{Boc\text{-}CH3}$), 19.1 (C14).

R_f: 0.07 (EtOAc 100%).

(6a*R*,8*R*,9*R*,9a*R*)-8-(6-amino-9*H*-purin-9-yl)-2,2,4,4-tetraisopropyltetrahydro-6*H*-furo[3,2-*f*][1,3,5,2,4]trioxadisilocin-9-yl trifluoromethanesulfonate (**18a**)

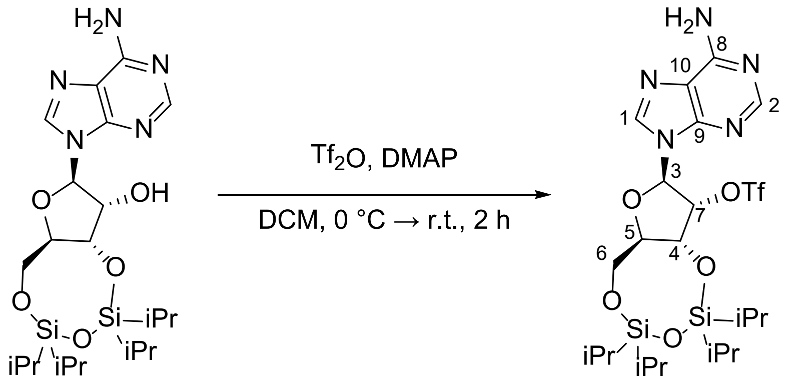

2a **18a**

3',5'-TIPDS-Adenosine (**2a**) (12.9 g, 25.4 mmol, 1.00 equiv.) and DMAP (9.29 g, 76.0 mmol, 3.00 equiv.) were dissolved in dry DCM (260 mL) and cooled to 0 °C. Trifluoromethanesulfonacid anhydride (Tf_2O; 5.11 mL, 30.4 mmol, 1.20 equiv.) was added dropwise to the white suspension resulting in a yellow mixture. The reaction warmed up to r.t. and stirred for 3 h until TLC analysis indicated full conversion. After adding cold water (30 mL) the organic phase was washed with water (50 mL), sat. aqueous NH_4Cl solution (50 mL) and sat. aqueous NaCl solution (50 mL) and dried over Na_2SO_4. The solvents were removed *in vacuo* and the product was purified *via* flash column chromatography (cHex/EtOAc, 1:1) to yield 2'-OTf-3',5'-TIPDS-adenosine **18a** (13.0 g, 20.3 mmol, 80 %) as colourless solid.

HR-MS (ESI): m/z calculated for $C_{23}H_{39}F_3N_5O_7SSi_2^+$: 642.2055; found: 642.2059.

^{1}H-NMR (400 MHz, DMSO): δ [ppm] = 8.26 (s, 1H, H^1), 8.03 (s, 1H, H^2), 7.45 (s, 2H, H^{NH2}), 6.46 (s, 1H, H^3), 6.08 (d, J = 4.9 Hz, 1H, H^4), 5.37 (dd, J = 9.0, 4.9 Hz, 1H, H^5), 4.12 – 3.92 (m, 3H, H^6, H^7), 1.14 – 0.91 (m, 28H, H^{iPr}).

^{13}C{^{1}H}-NMR (101 MHz, DMSO): δ [ppm] = 156.2 (C^8), 152. 6 (C^2), 148.5 (C^9), 140.0 (C^1), 119.1 (C^{10}), 89.5 (C^4), 85.5 (C^3), 80.3 (C^7), 68.3 (C^5), 59.8 (C^6), 17.3 – 12.0 (12C, C^{iPr}).

R_f: 0.76 (EtOAc 100 %).

(2*R*,3*S*,4*R*,5*R*)-5-(6-amino-9*H*-purin-9-yl)-4-hydroxy-2-(hydroxymethyl)tetrahydrofuran-3-yl trifluoromethanesulfonate (**19a**)

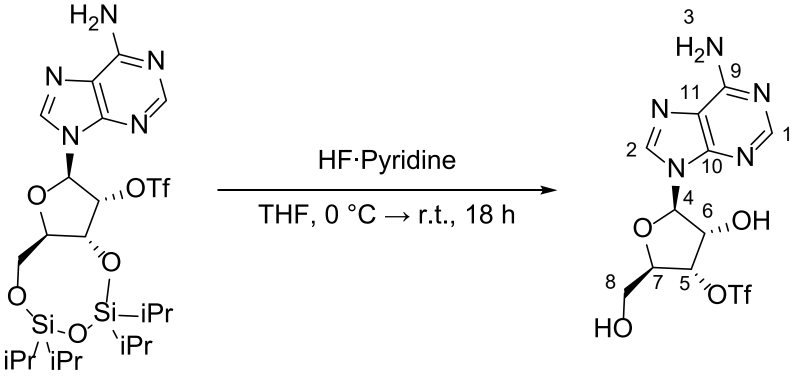

18a **19a**

Compound **18a** (2.63 g, 4.10 mmol, 1.00 equiv.) was dissolved in dry THF (12 mL) and the solution was cooled to 0 °C. HF·pyridine (70 wt.%; 1.06 mL, 40.1 mmol, 10.0 equiv.) was added dropwise and the mixture was stirred for 18 h until TLC confirmed full conversion. Excess fluoride was quenched by adding TMS-OMe (10 mL) and stirring for an additional 30 min. All volatiles were removed *in vacuo* and the residue was purified by flash column chromatography (cHex/EtOAc 4:1 → EtOAc 100 %). Compound **19a** (1.43 g, 3.57 mmol, 87 %) was obtained as a white foam.

HR-MS (ESI): m/z calculated for $C_{11}H_{13}F_3N_5O_6S^+$: 400.0533; found: 400.0533.

^{1}H-NMR (400 MHz, CD_3CN): δ [ppm] = 8.24 (s, 1H, H^1), 8.13 (s, 1H, H^2), 6.50 (s, 2H, H^3), 6.31 (d, *J*=5.8, 1H, H^4), 5.87 (t, *J*=5.3, 1H, H^5), 4.68 (s, 1H, H^6), 4.23 (q, *J*=2.3, 1H, H^7), 3.78 (m, 2H, H^8).

^{13}C{^{1}H}-NMR (101 MHz, CD_3CN): δ [ppm] = 156.6 (C^9), 152.3 (C^1), 149.6 (C^{10}), 141.7 (C^2), 121.3 (C^{11}), 88.0 (C^7), 87.3 (C^4), 87.1 (C^5), 70.4 (C^6), 62.3 (C^8).

R_f: 0.22 (EtOAc 100 %).

(2*R*,5*S*)-2-(6-amino-9*H*-purin-9-yl)-3-hydroxy-5-(hydroxymethyl)tetrahydrofuran-3-carbonitrile (**22a**)

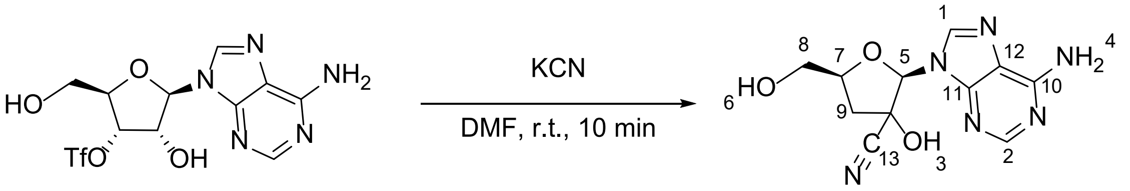

19a **22a**

Triflate **19a** (45.5 mg, 114 µmol, 1.00 equiv.) was dissolved in dry DMF (4 mL). KCN (37.1 mg, 570 µmol, 5.00 equiv.) was added and the solution was stirred at r.t. for 10 min. An aliquot was taken and quenched with basic aqueous NaClO solution. TLC analysis indicated full consumption of the starting material, the solution was diluted with 2 mL H_2O and excess cyanide was precipitated from the solution using $FeCl_3$ (100 mg). The mixture was extracted with EtOAc (3 × 5 mL) and the combined organic phases were dried over Na_2SO_4. The volatiles were removed under reduced pressure and the residue was purified *via* flash column chromatography (DCM 100 % → DCM/MeOH 70:30) to obtain cyanhydrine **22a** (840 mg, 114 µmol, 27 %) with minor impurities.

HR-MS (ESI): m/z calculated for $C_{11}H_{13}N_6O_3^+$: 277.1044; found: 277.1045.

^{1}H-NMR (400 MHz, DMSO): δ [ppm] = 8.55 (s, 1H, H^1), 8.18 (s, 1H, H^2), 7.76 (s, 1H, H^3), 7.39 (d, *J* = 5.5, 2H, H^4), 6.22 (s, 1H, H^5), 5.34 (t, *J* = 5.3, 1H, H^6), 4.42 (ddd, *J* = 11.3, 4.8, 2.7, 1H, H^7), 3.76 (dddd, *J* = 70.6, 12.3, 5.5, 3.3, 2H, H^8), 2.81 – 2.38 (m, 2H, H^9).

^{13}C{^{1}H}-NMR (101 MHz, DMSO): δ [ppm] = 156.1 (C^{10}), 152.8 (C^2), 149.1 (C^{11}), 138.08 (C^1), 118.7 (C^{12}), 11.0 (C^{13}), 90.4 (C^5), 81.6 (C^7), 76.5 (C^{14}), 60.7 (C^8), 38.0 (C^9).

R$_f$: 0.10 (EtOAc 100 %).

Vidarabine-based Compounds

(6a*R*,8*R*,9*S*,9a*S*)-8-(6-amino-9*H*-purin-9-yl)-2,2,4,4-tetraisopropyltetrahydro-6*H*-furo[3,2-*f*][1,3,5,2,4]trioxadisilocin-9-ol (**S05**)

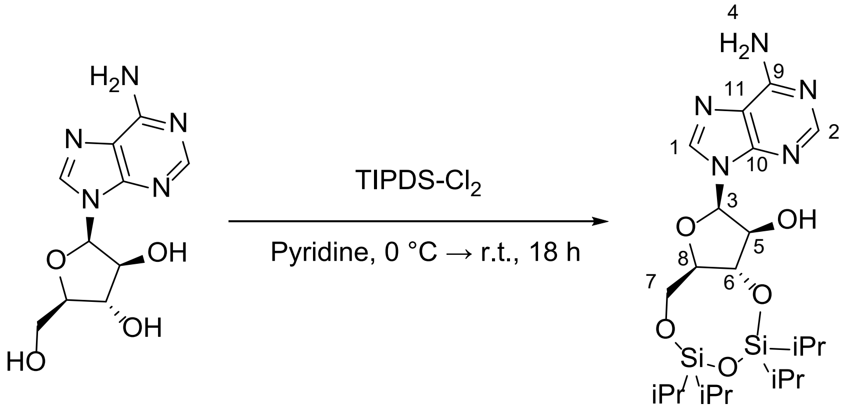

S05

Pyridine (60 mL) was added to vidarabine (4.72 g, 17.7 mmol, 1.00 equiv.). The suspension was cooled to 0 °C and TIPDS-Cl_2 (6.20 mL, 19.4 mmol, 1.10 equiv.) was added over 20 min. The reaction mixture was stirred and allowed to warm to r.t. over 18 h at which point TLC indicated full conversion. Toluene (100 mL) was added and the mixture was concentrated *in vacuo*. The crude material was purified by column chromatography (EtOAc/MeOH 98:2 → EtOAc/MeOH 95:5) to give compound **S05** as a colourless foam (7.83 g, 15.4 mmol, 87 %).

HR-MS (ESI): m/z calculated for $C_{22}H_{40}N_5O_5Si_2^+$: 510.2562; found: 510.2562.

^{1}H-NMR (400 MHz, $CDCl_3$): δ [ppm] = 8.22 (s, 1H, H^1), 8.09 (s, 1H, H^2), 6.16 (d, *J* = 5.8, 1H, H^3), 5.89 (s, 2H, H^4), 4.72 – 4.61 (m, 2H, $H^{5,6}$), 4.01 (d, *J* = 3.4, 2H, H^7), 3.84 (dt, *J* = 9.3, 3.3, 1H, H^8), 1.16 – 0.98 (m, 28H, H^{iPr}).

^{13}C{^{1}H}-NMR (101 MHz, $CDCl_3$): δ [ppm] = 155.7 (C^9), 152.8 (C^2), 149.4 (C^{10}), 140.6 (C^1), 120.0 (C^{11}), 84.2 (C^3), 81.3 (C^8), 77.2 (C^5), 74.4 (C^6), 61.5 (C^7), 17.8 – 12.2 (12C, C^{iPr}).

R_f: 0.63 (EtOAc/MeOH 9:1 with 1 % Et_3N).

(6a*R*,8*R*,9*S*,9a*R*)-8-(6-amino-9*H*-purin-9-yl)-2,2,4,4-tetraisopropyltetrahydro-6*H*-furo[3,2-*f*][1,3,5,2,4]trioxadisilocin-9-yl (*tert*-butoxycarbonyl)-L-alaninate (**11g**)

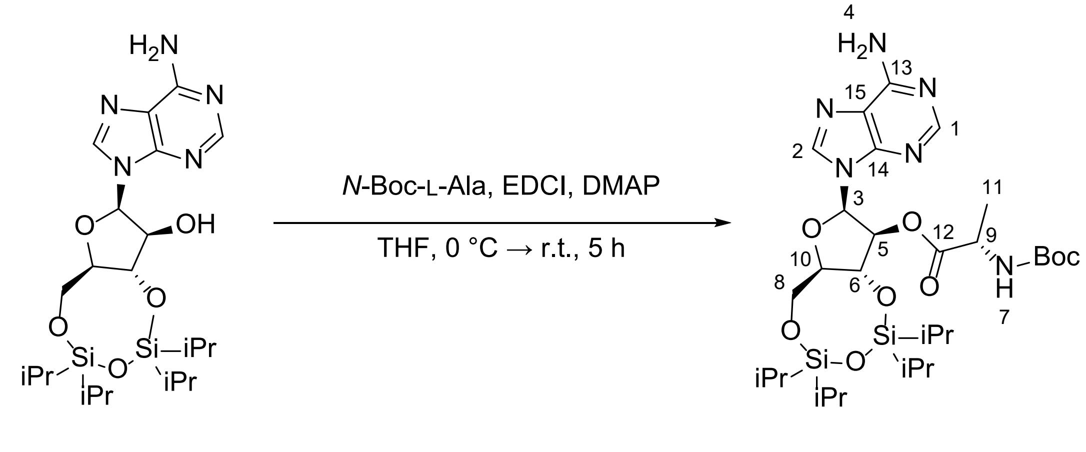

S05 **11g**

N-Boc-L-Alanine (445 mg, 2.35 mmol, 1.20 equiv.) was dissolved in dry THF (25 mL). The solution was cooled to 0 °C and EDCI·HCl (564 mg, 1.50 mmol, 1.50 equiv.) was added. After 15 min, **S05** (500 mg, 980 µmol, 1.00 equiv.) and DMAP (240 mg, 1.96 mmol, 2.00 equiv.) were added. The reaction mixture was allowed to warm to r.t. and stirred for 2 h and subsequently concentrated *in vacuo*. The crude material was purified by column chromatography (cHex/EtOAc 1:4) to give compound **11g** as white foam (668 mg, 2.35 mmol, 100 %).

HR-MS (ESI): m/z calculated for $C_{30}H_{53}N_6O_8Si_2^+$: 681.3458; found: 681.3469.

^{1}H-NMR (400 MHz, CDCl$_3$): δ [ppm] = 8.3 (s, 1H, H^1), 7.9 (s, 1H, H^2), 6.5 (d, *J* = 6.6, 1H, H^3), 5.7 (s, 2H, H^4), 5.5 (t, *J* = 7.3, 1H, H^5), 5.3 (t, *J* = 8.1, 1H, H^6), 4.8 (d, *J* = 8.1, 1H, H^7), 4.3 (dd, *J* = 12.5, 4.9, 1H, H^{8a}), 4.1 (qd, *J* = 7.3, 3.7, 1H, H^9), 4.0 (dd, *J* = 12.5, 3.2, 1H, H^{8b}), 3.9 (ddd, *J* = 8.1, 4.8, 3.1, 1H, H^{10}), 1.4 (s, 9H, H^{Boc}), 1.2 – 1.0 (m, 28H, H^{iPr}), 0.5 (d, *J* = 7.2, 3H, H^{11}).

^{13}C{^{1}H}-NMR (101 MHz, CDCl$_3$): δ [ppm] = 172.5 (C^{12}), 155.5 (C^{13}), 154.9 (C$^{Boc\text{-}CO}$), 153.2 (C^1), 149.8 (C^{14}), 140.4 (C^2), 119.9 (C^{15}), 81.4 (C^3), 80.6 (C^{10}), 78.9 (C^5), 77.4 (C$^{Boc\text{-}quart.}$), 72.8 (C^6), 61.8 (C^8), 48.7 (C^9), 28.4 (C$^{Boc\text{-}CH3}$), 17.8 – 12.4 (13C, C^{11,iPr}).

R$_f$: 0.51 (EtOAc 100%).

(2R,3S,4R,5R)-2-(6-amino-9H-purin-9-yl)-4-hydroxy-5-(hydroxymethyl)tetrahydrofuran-3-yl (*tert*-butoxycarbonyl)-L-alaninate (**S06**)

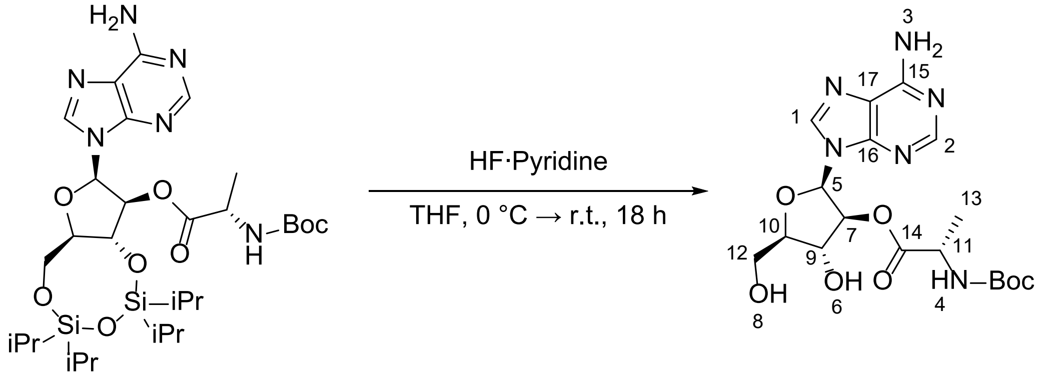

Compound **11g** (86.2 mg, 0.127 mmol, 1.00 equiv.) was dissolved in dry THF (0.75 mL) and the solution was cooled to 0 °C. HF·pyridine (70 wt.%; 0.030 mL, 1.14 mmol, 9.00 equiv.) was added dropwise and the mixture was stirred for 72 h until TLC confirmed full conversion. Excess fluoride was quenched by adding TMS-OMe (150 µL) and stirring for an additional 30 min. All volatiles were removed *in vacuo* and the residue was purified by flash column chromatography (H_2O/ACN 95:5 → ACN 100 %). Compound **S06** (54.9 mg,125 µmol, 99 %) was obtained as a white solid.

HR-MS (ESI): m/z calculated for $C_{18}H_{27}N_6O_7^+$: 439.1936; found: 439.1939.

^{1}H-NMR (400 MHz, $CDCl_3$): δ [ppm] = 8.21 (s, 1H, H^1), 8.11 (s, 1H, H^2), 7.30 (s, 1H, H^3), 7.17 (d, *J* = 7.3, 1H, H^4), 6.45 (d, *J* = 6.1, 1H, H^5), 5.90 (s, 1H, H^6), 5.28 (t, *J* = 6.4, 1H, H^7), 5.08 (t, *J* = 5.6, 1H, H^8), 4.56 (t, *J* = 6.8, 1H, H^9), 3.87 – 3.77 (m, 1H, $H^{10,11}$), 3.75 – 3.62 (m, 1H, H^{12}), 0.45 (d, *J* = 7.3, 3H, H^{13}).

^{13}C{^{1}H}-NMR (101 MHz, $CDCl_3$): δ [ppm] = 172.2 (C^{14}), 156.0 (C^{15}), 155.2 ($C^{Boc\text{-}CO}$), 152.7 (C^2), 149.1 (C^{16}), 139.9 (C^1), 118.4 (C^{17}), 82.6 (C^{11}), 80.7 (C^5), 78.2 (C^7), 78.0 ($C^{Boc\text{-}quart.}$), 71.4 (C^9), 60.5 (C^{10}), 48.4 (C^{13}), 28.2 ($C^{Boc\text{-}CH3}$), 15.3 (C^{12}).

R_f: 0.30 (DCM/MeOH 9:1).

(2*R*,3*S*,4*R*,5*R*)-2-(6-amino-9*H*-purin-9-yl)-4-hydroxy-5-(hydroxymethyl)tetrahydrofuran-3-yl L-alaninate hydrochloride (**16g**)

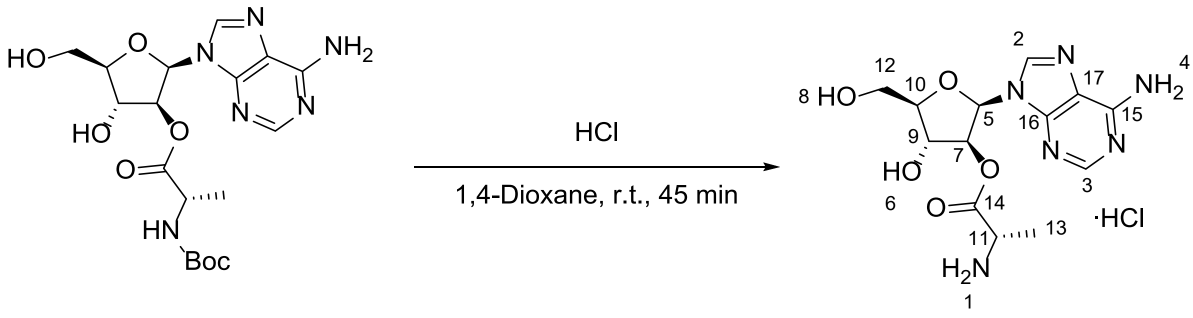

S06 **16g**

S06 (65.7 mg, 0.150 mmol, 1.00 equiv.) was dissolved in 1,4-dioxane (1 mL) and HCl in 1,4-dioxane (4 M; 0.375 mL, 0.740 mmol, 5.00 equiv.) was added. The reaction was stirred for 0.75 h, filtered and washed with MeOH. The mixture was concentrated *in vacuo* and purified by column chromatography (18-C, H_2O/ACN 95:5) to give compound **16g** as white foam (30.6 mg, 0.093 mmol, 62 %).

HR-MS (ESI): m/z calculated for $C_{18}H_{27}N_6O_7^+$: 339.1411; found: 339.1413.

^{1}H-NMR (400 MHz, $CDCl_3$): δ [ppm] = 8.40 (s, 2H, H^1), 8.32 (s, 1H, H^2), 8.13 (s, 1H, H^3), 7.37 (s, 2H, H^4), 6.48 (d, *J* = 6.1 Hz, 1H, H^5), 6.00 (d, *J* = 5.6 Hz, 1H, H^6), 5.46 (dd, *J* = 6.3, 6.3 Hz, 1H, H^7), 5.16 (s, 1H, H^8), 4.58 (q, *J* = 6.4 Hz, 1H, H^9), 3.98 – 3.85 (m, 2H, $H^{10,11}$), 3.74 (dd, *J* = 12.2, 3.3 Hz, 1H, H^{12a}), 3.66 (dd, *J* = 12.2, 4.9 Hz, 1H, $H^{12b'}$), 0.64 (d, *J* = 7.2 Hz, 3H, H^{13}).

^{13}C{^{1}H}-NMR (101 MHz, $CDCl_3$): δ [ppm] = 169.3 (C^{14}), 155.9 (C^{15}), 152.7 (C^3), 149.1 (C^{16}), 139.8 (C^2), 118.4 (C^{17}), 82.6 (C^{10}), 80.5 (C^5), 78.7 (C^7), 71.3 (C^9), 60.2 (C^{12}), 47.5 (C^{11}), 14.4 (C^{13}).

R_f: 0.01 (DCM/MeOH 9:1).

(2*R*,3*S*,4*S*,5*R*)-5-(6-amino-9*H*-purin-9-yl)-4-hydroxy-2-(hydroxymethyl)tetrahydrofuran-3-yl L-alaninate (**17g**)

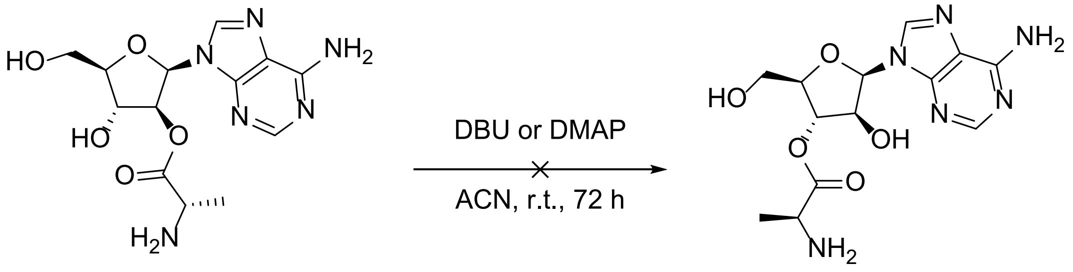

16g **17g**

Both methods listed below are modified literature procedures known to induce an acyl shift in similar substrates.[1]

Method 1: Ester **16g** (15.7 mg, 46.4 µmol, 1.00 equiv.) was dissolved in ACN (800 µL) and either different amounts of DBU (3.50 µL, 23.2 µmol, 0.500 equiv.) up to (14.0 µL, 92.8 µmol, 2.00 equiv.) or DMAP (2.80 mg, 23.2 µmol, 0.500 equiv.) were added. The resulting solution was stirred at r.t. Aliquots of 10 µL were diluted in H_2O (990 µL) and subjected to UPLC-QTOF analysis in regular intervals.

Method 2: Ester **16g** (15.7 mg, 46.4 µmol, 1.00 equiv.) was dissolved in acetonitrile-*d3* (750 µL) and DBU (3.50 µL, 23.5 µmol, 0.500 equiv.) or NEt_3 (3.20 µL, 23.2 µmol, 0.500 equiv.) were added. The resulting solution was subjected to ^{1}H-NMR analysis in regular intervals.

Neither method led to the observation of any kind of ester migration reaction, however degradation of the reactant **16a** to adenosine and adenine could be observed with prolonged time in solution.

(6a*R*,8*R*,9*S*,9a*R*)-8-(6-amino-9*H*-purin-9-yl)-2,2,4,4-tetraisopropyltetrahydro-6*H*-furo[3,2-*f*][1,3,5,2,4]trioxadisilocin-9-yl trifluoromethanesulfonate (**S07**)

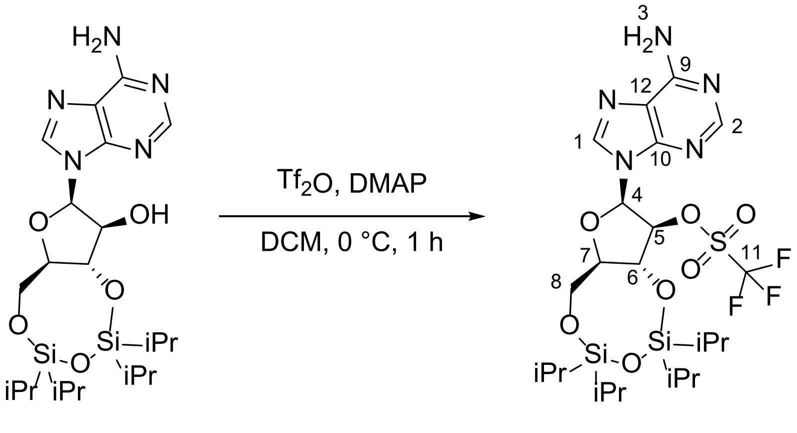

S05 **S07**

S05 (2.55 g, 5.00 mmol, 1.00 equiv.) and 4-Dimethylaminopyridine (1.80 g, 15.0 mmol, 3.00 equiv.) were dissolved in dry DCM (26 mL). The mixture was cooled to 0 °C and Tf_2O (900 µL, 5.50 mmol, 1.10 equiv.) was added slowly over 5 min. The reaction was stirred and allowed to warm to r.t. over 1 h until TLC analysis indicated full conversion. Water (10 mL) was added, the phases were separated and the organic layer was washed with sat. aqueous solution NH_4Cl (50 mL) and sat. aqueous NaCl solution (50 mL). It was then dried over Na_2SO_4 and concentrated *in vacuo*. The crude material was purified by column chromatography (cHex/EtOAc 1:1) to give **S07** (2.45 g, 3.89 mmol, 78 %) as colourless foam.

HR-MS (ESI): m/z calculated for $C_{23}H_{39}F_3N_5O_7SSi_2^+$: 642.2055; found: 642.2057.

^{1}H-NMR (400 MHz, DMSO): δ [ppm] = 8.30 (s, 1H, H^1), 8.08 (s, 1H, H^2), 7.47 (s, 2H, H^3), 6.48 (d, *J* = 7.1 Hz, 1H, H^4), 6.06 (dd, *J* = 7.6, 7.6 Hz, 1H, H^5), 5.67 (dd, *J* = 8.1, 8.1 Hz, 1H, H^6), 4.26 – 3.89 (m, 3H, $H^{7,8}$), 1.34 – 0.99 (m, 28H, H^{iPr}).

^{13}C{^{1}H}-NMR (101 MHz, DMSO): δ [ppm] = 156.0 (C^9), 152.3 (C^2), 149.1 (C^{10}), 140.6 (C^1), q, 122.5 – 113.0 (C^{11}), 116.1 (C^{12}), 89.3 – 78.5 ($C^{4,5,7}$), 74.2 (C^6), 62.1 (C^8), 17.3 – 12.3 (12C, C^{iPr}).

R_f: 0.68 (EtOAc).

S-((6a*R*,8*R*,9*R*,9a*R*)-8-(6-amino-9*H*-purin-9-yl)-2,2,4,4-tetraisopropyltetrahydro-6*H*-furo[3,2-*f*][1,3,5,2,4]trioxadisilocin-9-yl) ethanethioate (**7a**)

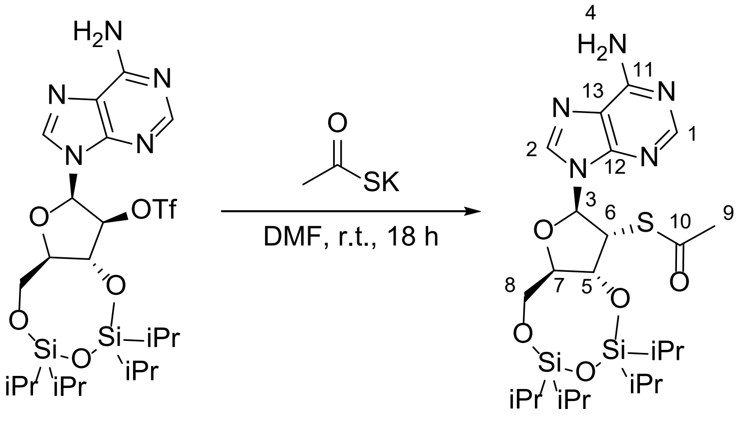

S07 **7a**

S07 (2.48 g, 3.87 mmol, 1.00 equiv.) and potassium thioacetate (685 mg, 6.00 mmol, 1.55 equiv.) were dissolved in dry DMF (20 mL) and stirred for 18 h. EtOAc (100 mL) was added and the solution was washed with water, sat. aqueous lithium chloride solution and sat. aqueous NaCl solution. The organic phase was dried over Na_2SO_4, concentrated *in vacuo* and the residue was purified *via* column chromatography (cHex/EtOAc 1:2) to yield **7a** (2.07 g, 3.64 mmol, 94 %) as a white solid.

HR-MS (ESI): m/z calculated for $C_{24}H_{42}N_5O_5SSi_2^+$: 568.2440; found: 568.2455.

^{1}H-NMR (400 MHz, DMSO): δ [ppm] = 8.31 (s, 1H, H^1), 7.95 (s, 1H, H^2), 6.06 (d, *J* = 4.7 Hz, 1H, H^3), 5.67 (d, *J* = 12.1 Hz, 2H, H^4), 5.31 – 5.23 (m, 1H, H^5), 4.70 (dd, *J* = 7.5, 4.7 Hz, 1H, H^6), 4.10 – 3.99 (m, 3H, $H^{7,8}$), 2.32 (s, 3H, H^9), 1.18 – 0.92 (m, 28H, H^{iPr}).

^{13}C{^{1}H}-NMR (101 MHz, DMSO): δ [ppm] = 195.0 (C^{10}), 155.6 (C^{11}), 153.2 (C^1), 149.8 (C^{12}), 139.8 (C^2), 120.3 (C^{13}), 88.8 (C^3), 85.0 (C^7), 71.5 (C^5), 62.8 (C^8), 51.6 (C^6), 30.7 (C^9), 17.6 -17.1 (8C, $C^{iPr\text{-}CH3}$), 13.4 - 12.8 (4C, $C^{iPr\text{-}CH}$).

R_f: 0.13 (cHex/EtOAc 1:2).

S-((2*R*,3*R*,4*R*,5*R*)-2-(6-amino-9*H*-purin-9-yl)-4-hydroxy-5-(hydroxymethyl)tetrahydrofuran-3-yl) ethanethioate (**8a**)

(2*R*,3*R*,4*R*,5*R*)-5-(6-amino-9*H*-purin-9-yl)-2-(hydroxymethyl)-4-mercaptotetrahydrofuran-3-yl acetate (**9a**)

(2*R*,3*R*,4*R*,5*R*)-4-(acetylthio)-5-(6-amino-9*H*-purin-9-yl)-2-(hydroxymethyl)tetrahydrofuran-3-yl acetate (**10a**)

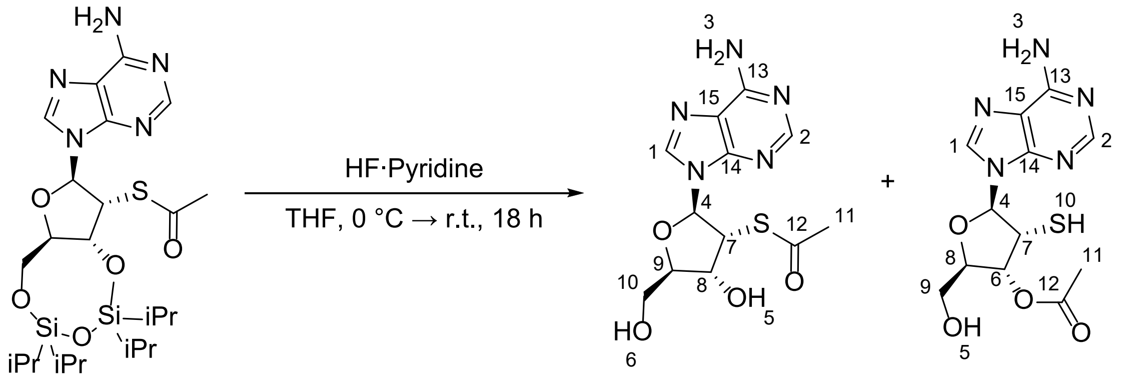

7a **8a** **9a**

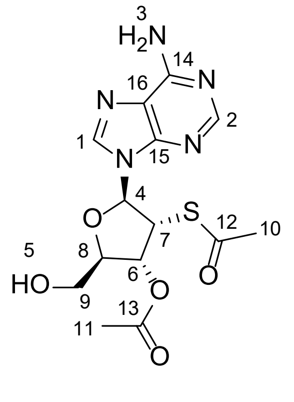

10a

7a (400 mg, 704 µmol, 1.00 equiv.) was dissolved in dry THF (10 mL) and the solution was cooled to 0 °C. HF·pyridine (70 wt.%; 370 µL, 14.5 µmol, 20.0 equiv.) was added, the reaction was allowed to warm to r.t. and was stirred for 18 h until complete conversion was indicated by TLC analysis. Excess fluoride was quenched by addition of TMS-OMe (3.0 mL) and after 30 min the volatiles were removed under diminished pressure. The residue was purified by flash column chromatography (EtOAc 100 % → EtOAc/MeOH 70:30) to yield three different products **8a** (16.0 mg, 49.3 µmol, 7 %), **9a** (65.9 µg, 203 µmol, 29 %) and **10a** (56.1 mg, 153 µmol, 22 %).

2'-Isomer **8a**:

HR-MS (ESI): m/z calculated for $C_{12}H_{16}N_5O_4S^+$: 326.0918; found: 326.0921.

^{1}H-NMR (400 MHz, DMSO): δ [ppm] = 8.32 (s, 1H, H^1), 8.14 (s, 1H, H^2), 7.36 (s, 2H, H^3), 6.10 (d, J = 9.1, 1H, H^4), 6.03 (d, J = 4.9, 1H, H^5), 5.77 (s, 1H, H^6), 4.76 (dd, J = 9.2, 5.0, 1H, H^7), 4.36 – 4.33 (m, 1H, H^8), 4.10 – 4.07 (m, 1H, H^9), 3.76 – 3.57 (m, 2H, H^{10}), 2.19 (s, 3H, H^{11}).

^{13}C{^{1}H}-NMR (101 MHz, DMSO): δ [ppm] = 193.71 (C^{12}), 156.15 (C^{13}), 152.37 (C^2), 149.16 (C^{14}), 139.74 (C^1), 119.24 (C^{15}), 88.29 (C^9), 87.74 (C^4), 72.34 (C^8), 61.96 (C^{10}), 50.07 (C^7), 30.26 (C^{11}).

R_f: 0.37 (EtOAc/MeOH 95:5).

3'-Isomer **9a**:

HR-MS (ESI): m/z calculated for $C_{12}H_{16}N_5O_4S^+$: 326.0918; found: 326.0921.

^{1}H-NMR (400 MHz, DMSO): δ [ppm] = 8.37 (s, 1H, H^1), 8.15 (s, 1H, H^2), 7.42 (s, 2H, H^3), 5.97 (d, J = 9.4, 1H, H^4), 5.65 (t, J = 6.1, 1H, H^5), 5.38 (dd, J = 5.4, 1.1, 1H, H^6), 4.40 (td, J = 9.7, 5.3, 1H, H^7), 4.15 (td, J = 3.5, 1.1, 1H, H^8), 3.65 (dddd, J = 19.3, 11.9, 7.7, 3.7, 2H, H^9), 3.05 (d, J = 9.9, 1H, H^{10}), 2.17 (s, 3H, H^{11}).

^{13}C{^{1}H}-NMR (101 MHz, DMSO): δ [ppm] = 169.6 (C^{12}), 156.3 (C^{13}), 152.6 (C^2), 149.21 (C^{14}), 139.8 (C^1), 119.3 (C^{15}), 89.7 (C^{12}), 84.9 (C^8), 75.2 (C^6), 61.6 (C^9), 42.5 (C^7), 20.7 (C^{11}).

R_f: 0.37 (EtOAc/MeOH 95:5).

2'- and 3'-acetylated **10a**:

HR-MS (ESI): m/z calculated for $C_{14}H_{18}N_5O_5S^+$: 368.1023; found: 368.1021.

^{1}H-NMR (400 MHz, DMSO): δ [ppm] = 8.35 (s, 1H, H^1), 8.15 (s, 1H, H^2), 7.44 (s, 2H, H^3), 6.15 (d, J = 9.4, 1H, H^4), 6.02 – 5.98 (m, 1H, H^5), 5.46 (d, J = 5.4, 1H, H^6), 4.95 (dd, J = 9.4, 5.4, 1H, H^7), 4.24 (q, J = 2.5, 1H, H^8), 3.80 – 3.56 (m, 2H, H^9), 2.22 (s, 3H, H^{10}), 2.13 (s, 3H, H^{11}).

$^{13}C\{^1H\}$-NMR (101 MHz, DMSO): δ [ppm] = 192.6 (C^{12}), 169.5 (C^{13}), 156.3 (C^{14}), 152.6 (C^{2}), 149.0 (C^{15}), 139.5 (C^{1}), 119.2 (C^{16}), 87.1 (C^{4}), 85.6 (C^{8}), 75.0 (C^{6}), 61.7 (C^{9}), 42.5 (C^{7}), 30.2 (C^{10}), 20.6 (C^{11}).

R_f: 0.41 (EtOAc/MeOH 95:5).

Guanosine-based Compounds

2-Amino-9-((6a*R*,8*R*,9*R*,9a*S*)-9-hydroxy-2,2,4,4-tetraisopropyltetrahydro-6*H*-furo[3,2-*f*][1,3,5,2,4]trioxadisilocin-8-yl)-1,9-dihydro-6*H*-purin-6-one (**2b**)

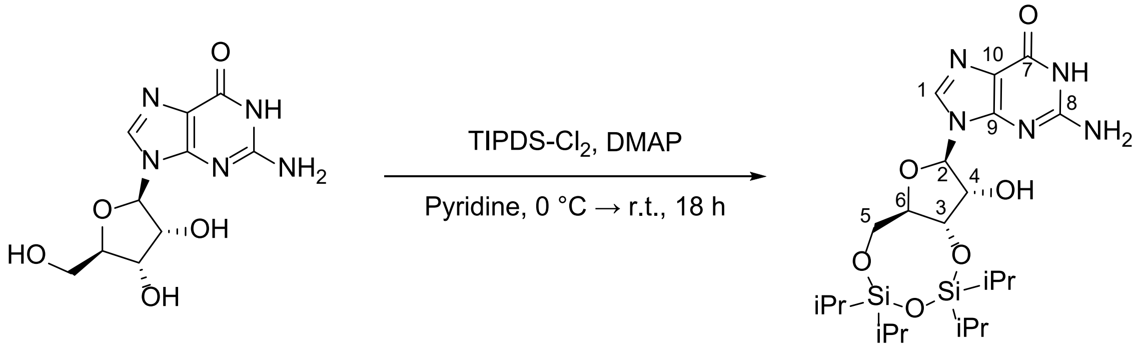

1b **2b**

Guanosine (**1b**) (2.83 g, 10.0 mmol, 1.00 equiv.) and DMAP (610 mg, 5.00 mmol, 0.500 equiv.) were dissolved in dry pyridine (25 mL) and cooled to 0 °C. TIPDS-Cl_2 (4.80 mL, 15.0 mmol, 1.50 equiv.) was added dropwise to the mixture and warmed to r.t. overnight. After adding water (10 mL) the solvents were removed *in vacuo* and the product was purified *via* flash column chromatography (RP, H_2O 100 % → ACN 100 %) to yield 3',5'-TIPDS-guanosine **2b** (2.37 g, 4.50 mmol, 45 %) as a colourless foam.

HR-MS (ESI): m/z calculated for $C_{22}H_{40}N_5O_6Si_2^+$: 526.2512; found: 526.2518.

^{1}H-NMR (400 MHz, DMSO): δ [ppm] = 7.75 (s, 1H, H^1), 6.50 (s, 2H, H^{NH2}), 5.67 (d, *J* = 1.8 Hz, 1H, H^2), 4.34 (dd, *J* = 8.1, 5.0 Hz, 1H, H^3), 4.25 (td, *J* = 5.1, 1.8 Hz, 1H, H^4), 4.12 – 3.86 (m, 3H, $H^{5,6}$), 1.10 – 0.94 (m, 28H, H^{iPr}).

^{13}C{^{1}H}-NMR (101 MHz, DMSO): δ [ppm] = 156.7 (C^7), 153.9 (C^8), 150.6 (C^9), 134.1 (C^1), 116.8 (C^{10}), 87.8 (C^2), 81.0 (C^6), 73.9 (C^4), 69.6 (C^3), 60.7 (C^5) 17.3 – 12.1 (12C, C^{iPr}).

R_f: 0.19 (EtOAc 100 %).

(6a*R*,8*R*,9*S*,9a*R*)-8-(2-Amino-6-oxo-1,6-dihydro-9*H*-purin-9-yl)-2,2,4,4-tetraisopropyltetrahydro-6*H*-furo[3,2-*f*][1,3,5,2,4]trioxadisilocin-9-yl (*tert*-butoxycarbonyl)-L-alaninate (**11b**)

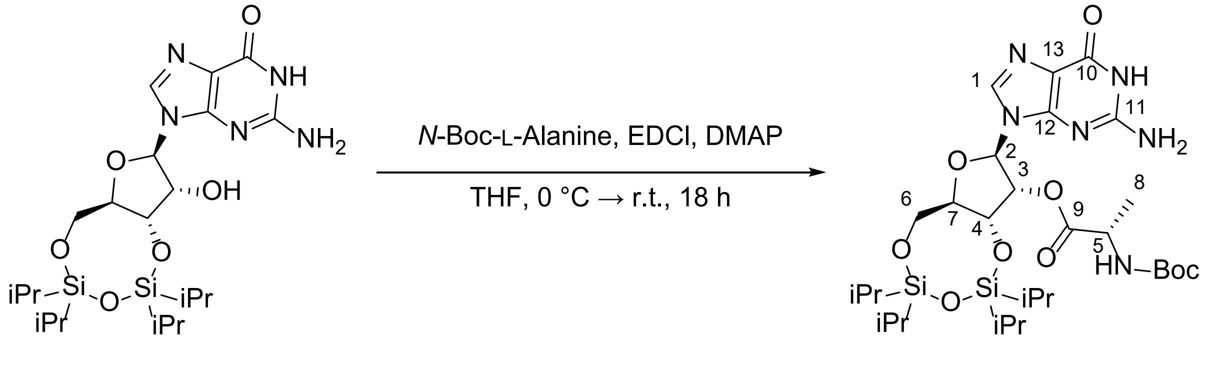

2b **11b**

N-Boc-L-Alanine (298 mg, 1.60 mmol, 1.05 equiv.) was dissolved in dry THF (15 mL) and cooled to 0 °C. EDCI·HCl (316 mg, 1.70 mmol, 1.10 equiv.) was added and the mixture was stirred for 15 min. 3',5'-TIPDS-Guanosine (**2b**) (788 mg, 1.50 mmol, 1.00 equiv.) and DMAP (275 mg, 2.25 mmol, 1.50 equiv.) were added and the reaction was allowed to warm to r.t. overnight. Once TLC analysis indicated full conversion, the volatiles were removed *in vacuo* and the residue was purified *via* flash column chromatography (DCM 100 % → DCM/MeOH 85:15) to yield 2'-*N*-Boc-alanine-3',5'-TIPDS-guanosine **11b** (603 mg, 900 µmol, 57 %) as colourless solid.

HR-MS (ESI): m/z calculated for $C_{30}H_{53}N_6O_9Si_2^+$: 697.3407; found: 697.3410.

^{1}H-NMR (400 MHz, DMSO): δ [ppm] = 7.85 (s, 1H, H^1), 5.89 (d, *J* = 1.6 Hz, 1H, H^2), 5.63 (dd, *J* = 5.2, 1.7 Hz, 1H, H^3), 4.64 (dd, *J* = 8.3, 5.2 Hz, 1H. H^4), 4.19 – 4.05 (m, 2H, $H^{5,6a}$), 4.01 – 3.89 (m, 2H, $H^{6b,7}$), 1.36 (s, 9H, H^{Boc}), 1.31 (d, *J* = 7.4 Hz, 3H, H^8), 1.09 – 0.95 (m, 28H, H^{iPr}).

^{13}C{^{1}H}-NMR (101 MHz, DMSO): δ [ppm] = 171.9 (C^9), 156.6 (C^{10}), 155.2 ($C^{Boc\text{-}CO}$), 154.0 (C^{11}), 150.5 (C^{12}), 134.7 (C^1), 116.7 (C^{13}), 84.9 (C^2), 81.2 (C^7), 78.3 ($C^{Boc\text{-}quart.}$), 75.0 (C^3), 68.7 (C^4), 60.1 (C^6), 48.8 (C^5), 28.2 ($C^{Boc\text{-}CH3}$), 17.4 – 16.6 (13C, C^8, C^{iPr}).

R_f: 0.42 (EtOAc 100 %).

(2*R*,3*S*,4*S*,5*R*)-5-(2-amino-6-oxo-1,6-dihydro-9*H*-purin-9-yl)-4-hydroxy-2-(hydroxymethyl)tetrahydrofuran-3-yl (*tert*-butoxycarbonyl)-L-alaninate (**12b**)

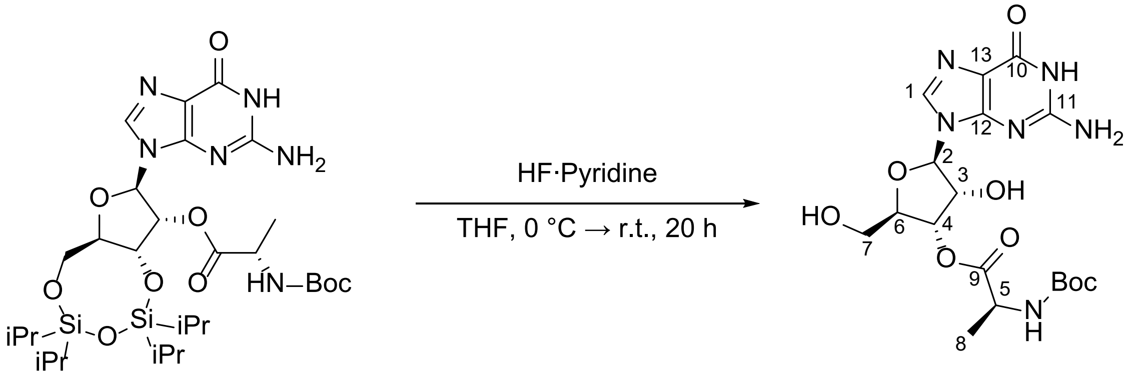

11b **12b**

2'-*N*-Boc-Alanine-3',5'-TIPDS-guanosine (**11b**) (105 mg, 0.150 mmol, 1.00 equiv.) was dissolved in THF (1 mL) and cooled to 0 °C. HF·pyridine (70 wt.%; 78.0 µL, 3.75 mmol, 25.0 equiv.) was added to the suspension and stirred overnight. After quenching of the excess fluoride using TMS-OMe (1 mL), the residue was purified *via* flash column chromatography (DCM 100 % → DCM/MeOH 85:15). 3'-*N*-Boc-alanine-guanosine **12b** (57.1 mg, 151 µmol, 83 %) was obtained with minor impurities.

HR-MS (ESI): m/z calculated for $C_{18}H_{27}N_6O_8^+$: 455.1885; found: 455.1887.

^{1}H-NMR (400 MHz, DMSO): δ [ppm] = 7.96 (s, 1H, H^1), 5.69 (dd, *J* = 6.7, 4.9 Hz, 1H, H^2), 5.21 (dd, *J* = 5.3, 2.0 Hz, 1H, H^3), 4.71 (q, *J* = 6.3 Hz, 1H, H^4), 4.14 (t, *J* = 7.4 Hz, 1H, H^5), 3.99 (q, *J* = 2.6, 1.9 Hz, 1H, H^6), 3.70 – 3.47 (m, 2H, H^7), 1.42 – 1.28 (m, 12H, H^8, H^{Boc}).

^{13}C{^{1}H}-NMR (101 MHz, DMSO): δ [ppm] = 172.3 (C^9), 156.7 (C^{10}), 155.3 ($C^{Boc\text{-}CO}$), 153.8 (C^{11}), 151.3 (C^{12}), 135.3 (C^1), 116.7 (C^{13}), 85.9 (C^2), 83.3 (C^6), 78.3 ($C^{Boc\text{-}quart.}$), 73.8 (C^4), 72.1 (C^3), 61.3 (C^7), 49.2 (C^5), 28.2 (2C, C^8, $C^{Boc\text{-}CH3}$).

R_f: 0.11 (EtOAc/MeOH 98:2).

Uridine-based Compounds

1-((6a*R*,8*R*,9*R*,9a*S*)-9-hydroxy-2,2,4,4-tetraisopropyltetrahydro-6*H*-furo[3,2-*f*][1,3,5,2,4]trioxadisilocin-8-yl)pyrimidine-2,4(1*H*,3*H*)-dione (**2d**)

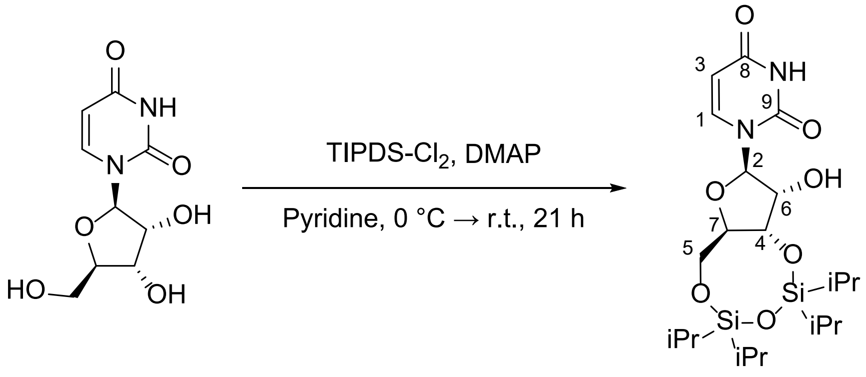

1d **2d**

Uridine (**1d**) (2.44 g, 10.0 mmol, 1.00 equiv.) and DMAP (610 mg, 5.00 mmol, 0.500 equiv.) were dissolved in dry pyridine (25 mL) and cooled to 0 °C. TIPDS-Cl_2 (4.80 mL, 15.0 mmol, 1.50 equiv.) was added dropwise to the mixture and warmed to r.t. overnight. After adding water (10 mL) the solvents were removed *in vacuo* and the residue was dissolved in DCM (100 mL). The organic phase was washed with sat. aqueous NaCl solution (50 mL) and the aqueous phase was re-extracted with DCM (3 × 50 mL). The combined organic phases were dried over Na_2SO_4 and the solvent was removed *in vacuo*. The residue was purified *via* flash column chromatography (DCM/MeOH 97:3 → DCM/MeOH 85:15). 3',5'-TIPDS-uridine **2d** (4.63 g, 9.52 mmol, 95 %) was obtained as a colourless foam.

HR-MS (ESI): m/z calculated for $C_{21}H_{39}N_2O_7Si_2^+$: 487.2290; found: 487.2295.

^{1}H-NMR (400 MHz, DMSO): δ [ppm] = 11.37 (s, 1H, H^{NH}), 7.68 (d, *J* = 8.1 Hz, 1H, H^1), 5.61 (d, *J* = 4.5 Hz, 1H, H^{OH}), 5.53 (s, 1H, H^2), 5.51 (s, 1H, H^3), 4.18 – 4.07 (m, 3H, $H^{4,5a,6}$), 3.97 (dt, *J* = 8.6, 2.5 Hz, 1H, H^7), 3.90 (dd, *J* = 13.2, 2.7 Hz, 1H, H^{5b}), 1.11 – 0.89 (m, 28H, H^{iPr}).

^{13}C{^{1}H}-NMR (101 MHz, DMSO): δ [ppm] = 163.3 (C^8), 150.2 (C^9), 139.8 (C^1), 101.0 (C^3), 90.6 (C^2), 80.9 (C^7), 73. 6 (C^6), 68.8 (C^4), 60.2 (C^2) 17.5 – 11.9 (12C, C^{iPr}).

R_f: 0.22 (DCM/MeOH 99:1).

(6a*R*,8*R*,9*R*,9a*R*)-8-(2,4-dioxo-3,4-dihydropyrimidin-1(2*H*)-yl)-2,2,4,4-tetraisopropyltetrahydro-6*H*-furo[3,2-*f*][1,3,5,2,4]trioxadisilocin-9-yl (*tert*-butoxycarbonyl)-L-alaninate (**11d**)

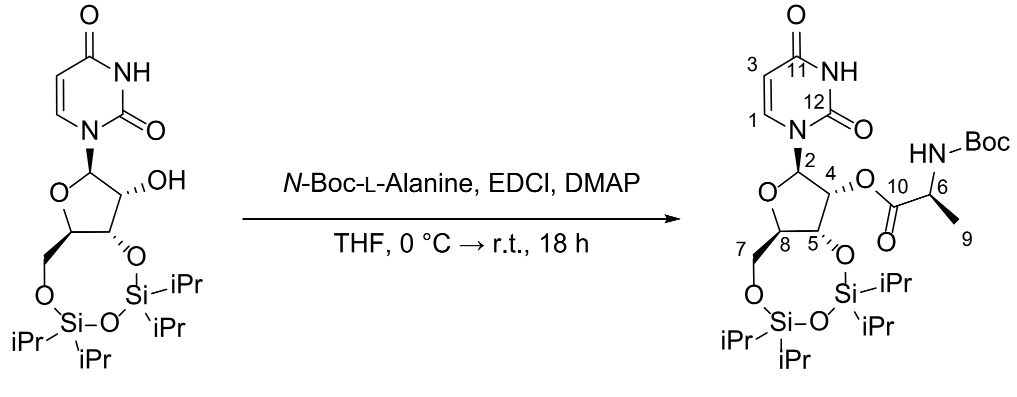

2d **11d**

N-Boc-Alanine (298 mg, 1.60 mmol, 1.05 equiv.) was dissolved in dry THF (15 mL) and cooled to 0 °C. EDCI·HCl (316 mg, 1.70 mmol, 1.10 equiv.) was added and the mixture was stirred for 15 min. 3',5'-TIPDS-Uridine **2d** (730 mg, 1.50 mmol, 1.00 equiv.) and DMAP (275 mg, 2.25 mmol, 1.50 equiv.) were added and the reaction was allowed to warm to r.t. overnight. The solvents were removed *in vacuo* and the residue was purified *via* flash column chromatography (DCM 100 % → DCM/MeOH 85:15) to yield 2'-*N*-Boc-alanine-3',5'-TIPDS-uridine **11d** (780 mg, 1.20 mmol, 80 %) as colourless solid.

HR-MS (ESI): m/z calculated for $C_{29}H_{52}N_3O_{10}Si_2^+$: 658.3186; found: 658.3191.

^{1}H-NMR (400 MHz, DMSO): δ [ppm] = 11.43 (s, 1H, H^{NH}), 7.66 (d, *J* = 8.0 Hz, 1H, H^1), 7.35 (d, *J* = 7.5 Hz, 1H, H^{NH}), 5.66 – 5.65 (m, 1H, H^2), 5.63 – 5.54 (m, 2H, H^3, H^4), 4.51 (dd, *J* = 9.2, 5.1 Hz, 1H, H^5), 4.18 – 4.07 (m, 2H, $H^{6,7a}$), 3.96 – 3.85 (m, 2H, $H^{7b,8}$), 1.36 (s, 9H, H^{Boc}), 1.34 – 1.21 (m, 3H, H^9), 1.06 – 0.81 (m, 37H, H^{iPr}).

^{13}C{^{1}H}-NMR (101 MHz, DMSO): δ [ppm] = 171.7 (C^{10}), 163.3 (C^{11}), 155.1 ($C^{Boc\text{-}CO}$), 150.1 (C^{12}), 140. 9 (C^1), 101.5 (C^3), 88.9 (C^2), 81.1 (C^5), 78.2 ($C^{Boc\text{-}quart.}$), 74.6 (C^4), 68.1 (C^5), 59.8 (C^7), 48.8 (C^6), 28.2 ($C^{Boc\text{-}CH3}$), 17.6 – 11.7 (12C, C^{iPr}).

R_f: 0.68 (EtOAc 100 %).

(2*R*,3*S*,4*R*,5*R*)-5-(2,4-dioxo-3,4-dihydropyrimidin-1(2*H*)-yl)-4-hydroxy-2-(hydroxymethyl)tetrahydrofuran-3-yl (*tert*-butoxycarbonyl)-L-alaninate (**12d**)

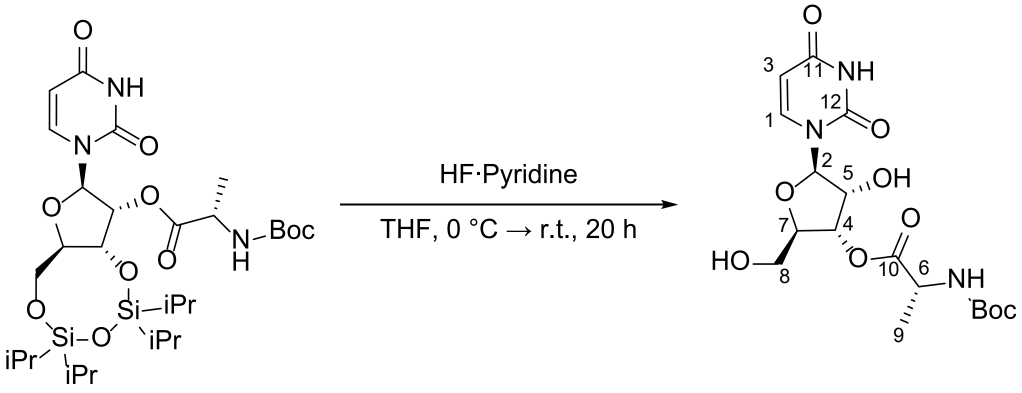

11d **12d**

2'-*N*-Boc-Alanine-3',5'-TIPDS-Uridine **11d** (99.0 mg, 150 µmol, 1.00 equiv.) was suspended in THF (1 mL) and cooled to 0 °C. HF·pyridine (70 wt.%; 78.0 µL, 3.75 mmol, 25.0 equiv.) was added to the suspension and stirred overnight until TLC analysis indicated full conversion. After quenching of the excess fluoride with TMS-OMe (1 mL), the product was purified *via* flash column chromatography (DCM 100 % → DCM/MeOH 85:15) to yield 2'-*N*-Boc-alanine-uridine **12d** (46.1 mg, 111 µmol, 73 %) as colourless solid. The product contained minor impurities after purification indicated by ^{1}H-NMR.

HR-MS (ESI): m/z calculated for $C_{17}H_{25}N_3NaO_9^+$: 438.1483; found: 438.1488.

^{1}H-NMR (400 MHz, DMSO): δ [ppm] = 7.88 (dd, *J* = 17.5, 8.1 Hz, 1H, H^1), 5.88 (d, *J* = 7.2 Hz, 1H, H^2), 5.75 – 5.62 (m, 1H, H^3), 5.24 – 5.07 (m, 1H, H^4), 4.34 – 4.20 (m, 1H, H^5), 4.08 (dp, *J* = 14.5, 7.3 Hz, 1H, H^6), 3.94 (q, *J* = 2.7 Hz, 1H, H^7), 3.63 – 3.52 (m, 2H, H^8), 1.39 (s, 9H, H^{Boc}) 1.27 (d, *J* = 8.1 Hz, 3H, H^9).

^{13}C{^{1}H}-NMR (101 MHz, DMSO): δ [ppm] = 172.4 (C^{10}), 163.0 (C^{11}), 155.4 ($C^{Boc\text{-}CO}$), 150.9 (C^{12}), 140.7 (C^1), 102.5 (C^3), 86.7 (C^2), 83.1 (C^7), 78.3 ($C^{Boc\text{-}quart.}$), 73.5 (C^4), 71.8 (C^5), 61.1 (C^8), 49.4 (C^6), 28.2 ($C^{Boc\text{-}CH3}$), 17.0 (C^9).

R_f: 0.41 (EtOAc 100 %).

Cytidine-based Compounds

4-Amino-1-((6a*R*,8*R*,9*R*,9a*S*)-9-hydroxy-2,2,4,4-tetraisopropyltetrahydro-6*H*-furo[3,2-*f*][1,3,5,2,4]trioxadisilocin-8-yl)pyrimidin-2(1*H*)-one (**2c**)

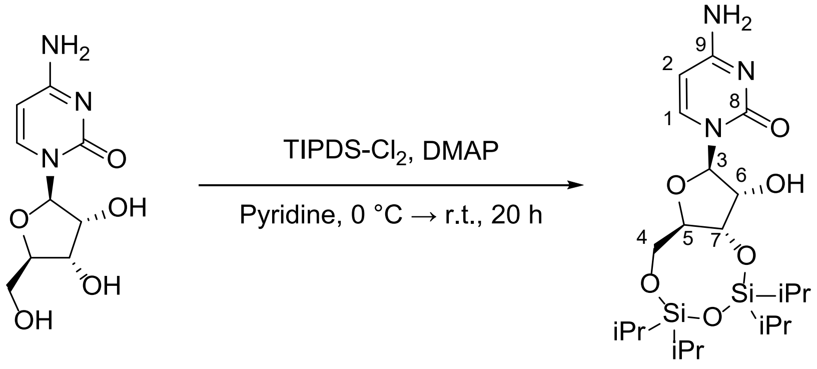

1c **2c**

Cytidine (**1c**) (2.43 g, 10.0 mmol, 1.00 equiv.) and DMAP (612 mg, 5.00 mmol, 0.500 equiv.) were dissolved in dry pyridine (25 mL) and cooled to 0 °C. TIPDS-Cl_2 (4.80 mL, 15.0 mmol, 1.50 equiv.) was added dropwise to the mixture and the reaction was warmed to r.t. overnight. After adding water (10 mL) the solvents were removed *in vacuo* and the product was purified *via* flash column chromatography (DCM/MeOH 97:3) to yield 3',5'-TIPDS-cytidine **2c** (4.62 g, 9.53 mmol, 95 %) as colourless foam.

HR-MS (ESI): m/z calculated for $C_{21}H_{40}N_3O_6Si_2^+$: 486.2450; found: 486.2453.

^{1}H-NMR (400 MHz, DMSO): δ [ppm] = 7.71 (d, J = 7.5 Hz, 1H, H^1), 7.16 (d, J = 22.5 Hz, 2H, H^{NH2}), 5.66 (d, J = 7.4 Hz, 1H, H^2), 5.55 (s, 1H, H^3), 4.20 – 4.11 (m, 1H, H^4), 4.07 (dd, J = 9.1, 4.4 Hz, 1H, H^5), 4.00 (dt, J = 9.1, 2.1 Hz, 1H, H^6), 3.96 – 3.86 (m, 2H, H^7, H^4), 1.11 – 0.89 (m, 28H, H^{iPr}).

^{13}C{^{1}H}-NMR (101 MHz, DMSO): δ [ppm] = 165.7 (C^8), 154.8 (C^9), 139.9 (C^1), 93.2 (C^2), 90.7 (C^3), 80.5 (C^6), 74.1 (C^7), 68.44 (C^5), 60.0 (C^4) 17.5 – 11.8 (12C, C^{iPr}).

R_f: 0.39 (EtOAc 100 %).

(6a*R*,8*R*,9*S*,9a*R*)-8-(4-Amino-2-oxopyrimidin-1(2*H*)-yl)-2,2,4,4-tetraisopropyltetrahydro-6*H*-furo[3,2-*f*][1,3,5,2,4]trioxadisilocin-9-yl (*tert*-butoxycarbonyl)-L-alaninate (**11c**)

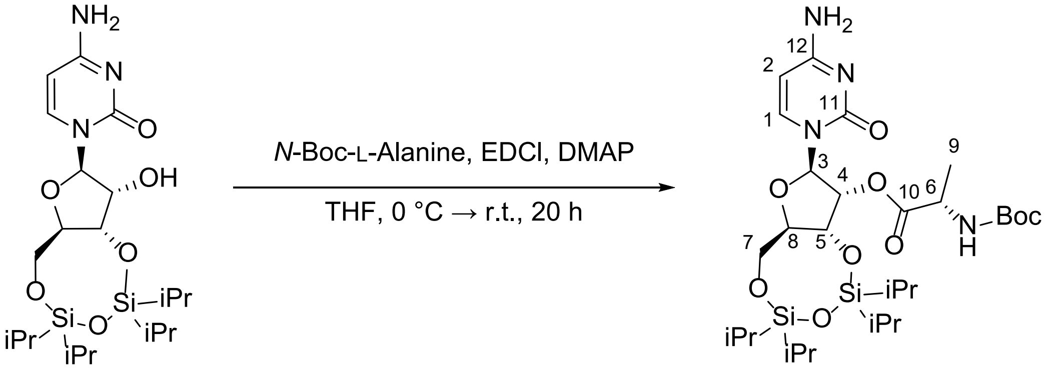

2c **11c**

N-Boc-Alanine (298 mg, 1.60 mmol, 1.05 equiv.) was dissolved in dry THF (15 mL) and cooled to 0 °C. EDCI·HCl (316 mg, 1.70 mmol, 1.10 equiv.) was added and the mixture was stirred for 15 min. 3',5'-TIPDS-cytidine **2c** (729 mg, 1.50 mmol, 1.00 equiv.) and DMAP (275 mg, 2.25 mmol, 1.50 equiv.) were added and the reaction was warmed to r.t. overnight. Once TLC analysis indicated full conversion, the solvents were removed *in vacuo* and the residue was purified *via* column chromatography (DCM 100 % → DCM/MeOH 85:15) to yield 2'-*N*-Boc-alanine-3',5'-TIPDS-cytidine **11c** (831 mg, 1.30 mmol, 84 %) as colourless solid.

HR-MS (ESI): m/z calculated for $C_{29}H_{53}N_4O_9Si_2^+$: 657.3346; found: 657.3359.

^{1}H-NMR (400 MHz, DMSO): δ [ppm] = 7.65 (d, *J* = 7.4 Hz, 1H, H^1), 7.35 (d, *J* = 7.6 Hz, 1H, H^{NH}), 7.28 (s, 2H, H^{NH2}), 5.71 (d, *J* = 7.4 Hz, 1H, H^2), 5.65 (s, 1H, H^3), 5.38 (d, *J* = 4.9 Hz, 1H, H^4), 4.44 (dd, *J* = 9.3, 4.9 Hz, 1H, H^5), 4.20 – 4.08 (m, 2H, H^6, H^{7a}), 3.97 – 3.87 (m, 2H, $H^{7b,8}$), 1.38 (s, 9H, H^{Boc}), 1.35 – 1.21 (m, 3H, H^9), 1.05 – 0.82 (m, 28H, H^{iPr}).

^{13}C{^{1}H}-NMR (101 MHz, DMSO): δ [ppm] = 171. 8 (C^{10}), 165. 9 (C^{11}), 155.1 (C^{Boc-CO}), 154.5 (C^{12}), 140.8 (C^1), 93.9 (C^2), 89.0 (C^3), 81.0 (C^8), 78.2 ($C^{Boc-quart.}$), 75.2(C^4), 68.0 (C^5), 59.9 (C^7), 48.8 (C^6), 28.2 ($C^{Boc-CH3}$), 17.6 – 11.8 (13C, C^9, C^{iPr}).

R$_f$: 0.62 (EtOAc 100 %).

(2*R*,3*S*,4*R*,5*R*)-5-(4-amino-2-oxopyrimidin-1(2*H*)-yl)-4-hydroxy-2-(hydroxymethyl)tetrahydrofuran-3-yl (*tert*-butoxycarbonyl)-L-alaninate (**12c**)

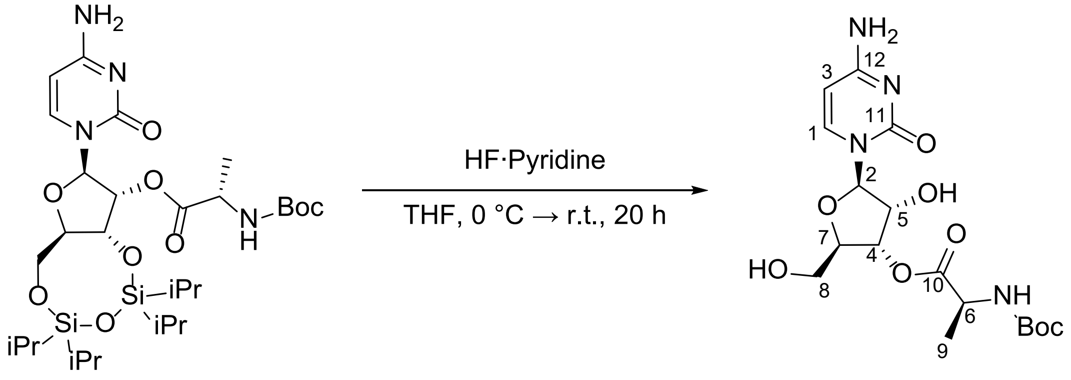

11c **12c**

2'-*N*-Boc-Alanine-3',5'-TIPDS-cytidine **11c** (96.2 mg, 150 µmol, 1.00 equiv.) was dissolved in THF (1 mL) and cooled to 0 °C. HF·pyridine (70 wt.%; 78.0 µL, 3.75 mmol, 25.0 equiv.) was added to the suspension and the reaction was stirred overnight at r.t. until MS analysis confirmed full conversion. After quenching with TMS-OMe (1 mL), the residue was purified *via* flash column chromatography (THF/DCM 50:50 → THF/DCM 90:10) to obtain 3'-*N*-Boc-alanine-cytidine **12c** (54.0 mg, 120 mmol, 80 %) as colourless solid.

HR-MS (ESI): m/z calculated for $C_{17}H_{27}N_4O_8^+$: 415.1823; found: 415.1828.

^{1}H-NMR (400 MHz, DMSO): δ [ppm] = = 7.68 (d, *J* = 7.5 Hz, 1H, H^1), 5.79 (d, *J* = 6.6 Hz, 1H, H^2), 5.62 (dd, *J* = 14.5, 7.4 Hz, 1H, H^3), 5.05 – 4.91 (m, 1H, H^4), 4.11 (p, *J* = 6.3 Hz, 1H, H^5), 3.98 (h, *J* = 7.5 Hz, 1H, H^6), 3.82 (q, *J* = 3.1 Hz, 1H, H^7), 3.54 – 3.38 (m, 2H, H^8), 1.26 (s, 9H, H^{Boc}) 1.17 (dd, *J* = 7.5, 3.5 Hz, 3H, H^9).

^{13}C{^{1}H}-NMR (101 MHz, DMSO): δ [ppm] = 172.4 (C^{10}), 165.5 (C^{11}), 155.4 (C^{12}), 141.3 (C^1), 139.2 ($C^{Boc\text{-}CO}$), 94.5 (C^3), 88.2 (C^2), 82.3 (C^7), 78.3 (C^{quart}), 73.2 (C^4), 72.1 (C^5), 61.0 (C^8), 49.3 (C^6), 28.2 ($C^{Boc\text{-}CH3}$), 17.2 (C^9).

R_f: 0.29 (EtOAc 100 %).

N^6,N^6-Dimethyladenosine-based Compounds

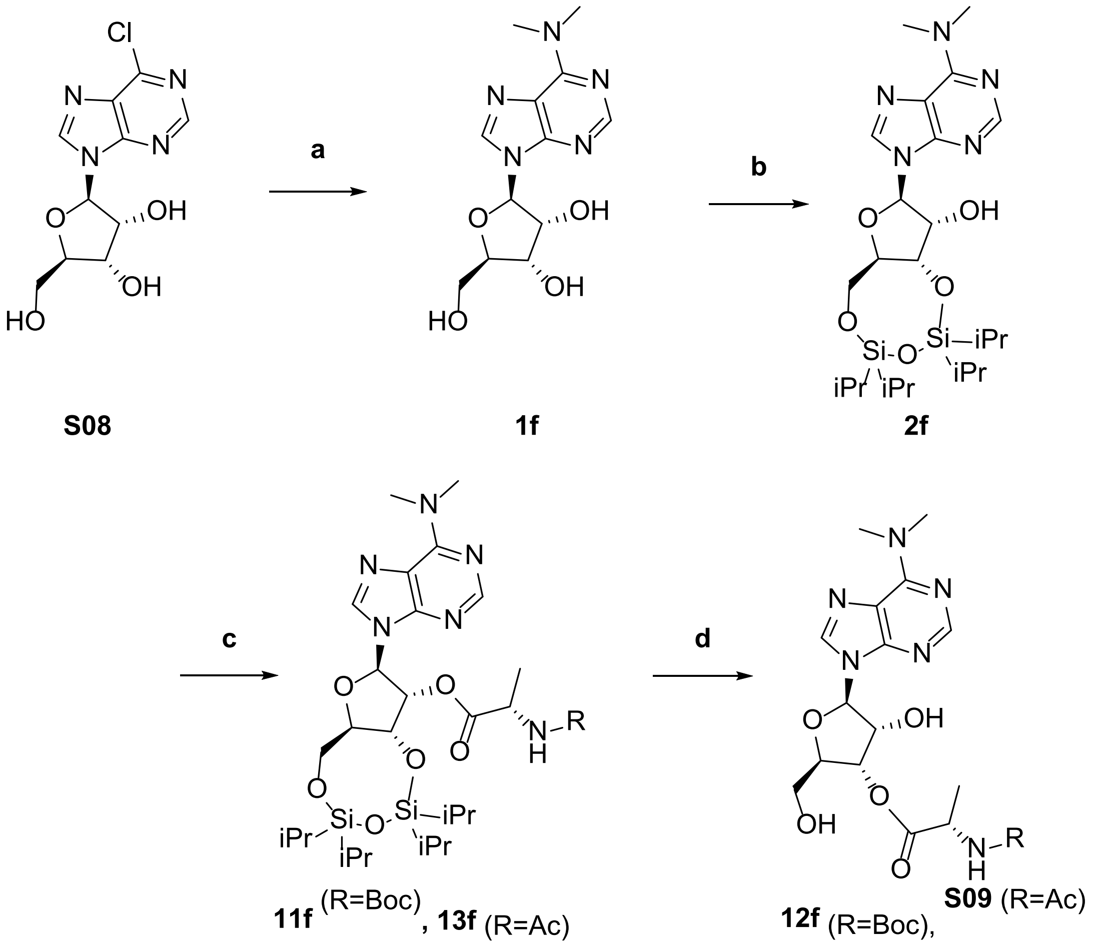

Scheme 1. Synthesis of the 3'-*N*-protected-alanine-DMAs (**12f** and **S09**). Conditions: a) Dimethylamine hydrochloride, NEt_3, DMF, 68 % b) $TIPDSCl_2$, pyridine, 64 % c) Boc-N-Ala-OH, DMAP, EDCI, THF, up to 72°% d) HF·pyridine, THF, up to 98 %. R = Boc or Ac.

(2*R*,3*R*,4*S*,5*R*)-2-(6-(dimethylamino)-9*H*-purin-9-yl)-5-(hydroxymethyl)tetrahydrofuran-3,4-diol (**1f**)

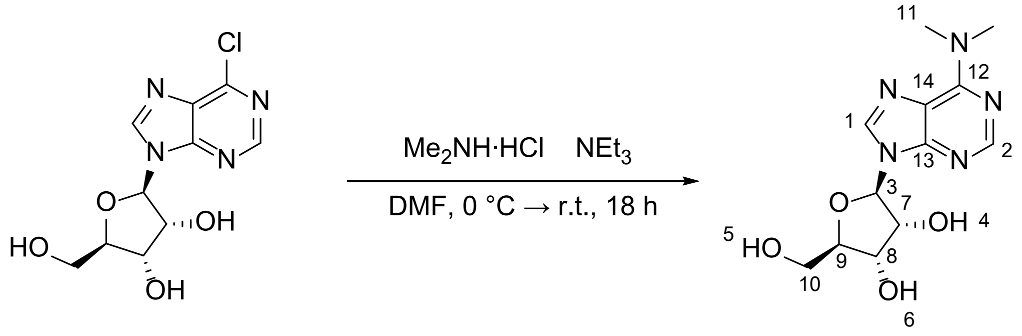

S08 **1f**

The synthesis was conducted similarly as presented by HERBERT *et al.*[2]

6-Chloropurine riboside (**S08**) (2.87 g, 10.0 mmol, 1.00 equiv.) and dimethylamine hydrochloride (1.06 mg, 13.0 mmol, 5.00 equiv.) were suspended in DMF and stirred for 30 min at 0 °C. After addition of Net_3 (2.20 mL, 15.8 mmol, 6.00 equiv.), the solution was stirred for 2 h at 0 °C and overnight at room temperature. The suspension was filtered and washed with ice-cold DMF (50 mL), the solvent was removed *in vacuo* resulting in a yellow solid. After purification by washing in acetone (80 mL) and recrystallizing in EtOH, **1f** was obtained as a white solid (2.01 g, 6.80 mmol, 68 %).

HR-MS (ESI): m/z calculated for $C_{12}H_{18}N_5O_4^+$: 296.1353; found 296.1351.

^{1}H-NMR (400 MHz, $CDCl_3$): δ [ppm] = 8.37 (s, 1H, H^1), 8.21 (s, 1H, H^2), 5.90 (d, J = 6.0 Hz, 1H, H^3), 5.46 (d, J = 6.2 Hz, 1H, H^4), 5.38 (dd, J = 7.0, 4.6 Hz, 1H, H^5), 5.20 (d, J = 4.8 Hz, 1H, H^6), 4.57 (td, J = 6.1, 4.9 Hz, 1H, H^7), 4.14 (td, J = 4.8, 3.2 Hz, 1H, H^8), 3.95 (q, J = 3.5 Hz, 1H, H^9), 3.75 – 3.62 (m, 1H, H^{10a}), 3.55 (ddd, J = 12.1, 7.0, 3.6 Hz, 1H, H^{10b}), 3.43 (d, J = 43.5 Hz, 5H, H^{11}).

^{13}C{^{1}H}-NMR (100 MHz, $CDCl_3$): δ [ppm] = 154.3 (C^{12}), 151.7 (C^2), 149.9 (C^{13}), 138.6 (C^1), 119.8 (C^{14}), 87.8 (C^3), 85.8 (C^9), 73.5 (C^7), 70.6 (C^8), 61.6 (C^{10}).

(6a*R*,8*R*,9*R*,9a*S*)-8-(6-(dimethylamino)-9*H*-purin-9-yl)-2,2,4,4-tetraisopropyltetrahydro-6*H*-furo[3,2-*f*][1,3,5,2,4]trioxadisilocin-9-ol (**2f**)

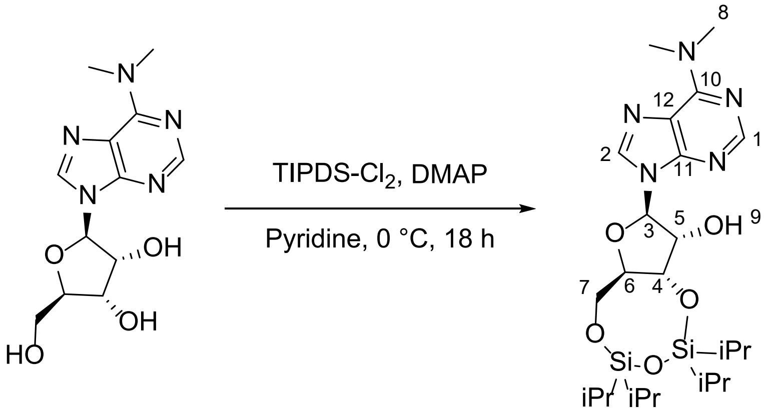

1f **2f**

Dimethyl adenosine (**1f**) (1.43 g, 4.85 mmol, 1.00 equiv.) and DMAP (300 mg, 2.43 mmol, 0.500 equiv.) were suspended in dry pyridine (12 mL) and the mixture was cooled to 0 °C. TIPDS-Cl_2 (1.86 mL, 5.82 mmol, 1.20 equiv.) was added dropwise and the mixture was slowly warmed to r.t. overnight. Cold water (20 mL) was added once TLC indicated full conversion and all volatiles were removed under reduced pressure. The residue was purified *via* column chromatography (cHex/EtOAc 1:3) to yield 3',5'-TIPDS-dimethyl adenosine **2f** (1.66 g, 3.10 mmol, 64 %) as a colourless oil.

HR-MS (ESI): m/z calculated for $C_{24}H_{44}N_5O_5Si_2^+$: 538.2875; found: 538.2877.

^{1}H-NMR (400 MHz, $CDCl_3$): δ [ppm] = 8.26 (s, 1H, H^1), 7.85 (s, 1H, H^2), 5.95 (d, *J* = 1.4 Hz, 1H, H^3), 5.15 (dd, *J* = 7.3, 5.5 Hz, 1H, H^4), 4.55 (dt, *J* = 5.6, 1.5 Hz, 1H, H^5), 4.17 – 4.05 (m, 2H, $H^{6,7a}$), 4.08 – 3.98 (m, 1H, H^{7b}), 3.51 (s, 6H, H^8), 3.3 (d, J = 1.7 Hz, 1H, H9), 1.16 – 1.01 (m, 28H, H^{iPr}).

^{13}C{^{1}H}-NMR (101 MHz, $CDCl_3$): δ [ppm] = 155.1 (C^{10}), 152.5 (C^1), 149.9 (C^{11}), 137.3 (C^2), 121.0 (C^{12}), 89.8 (C^3), 82.4 (C^6), 75.3 (C^5), 71.3 (C^4), 62.3 (C^7), 17.6 – 17.1 (8C, $C^{iPr\text{-}CH3}$), 13.4 – 12.7 (4C, $C^{iPr\text{-}CH}$).

R_f: 0.47 (EtOAc).

(6a*R*,8*R*,9*R*,9a*R*)-8-(6-(dimethylamino)-9*H*-purin-9-yl)-2,2,4,4-tetraisopropyltetrahydro-6*H*-furo[3,2-*f*][1,3,5,2,4]trioxadisilocin-9-yl (*tert*-butoxycarbonyl)-L-alaninate (**11f**)

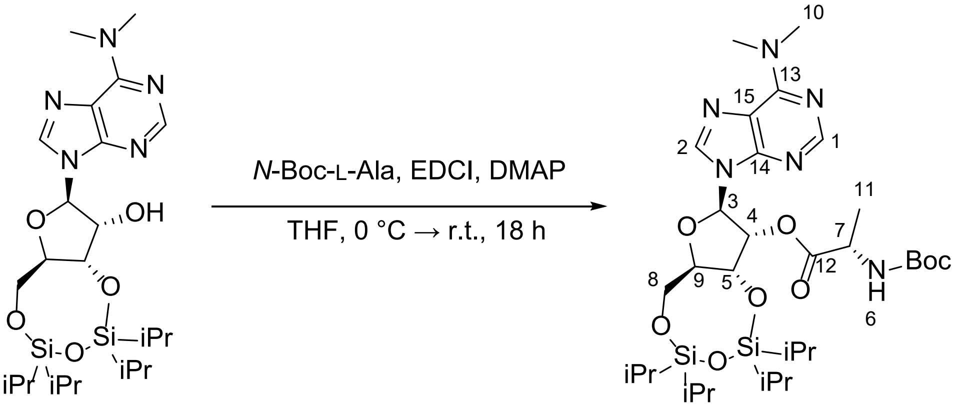

2f **11f**

N-Boc-L-Alanine (348 mg, 1.84 mmol, 1.00 equiv.) was dissolved in dry THF (20 mL). The mixture was cooled to 0 °C and EDCI·HCl (388 mg, 2.02 mmol, 1.10 equiv.) was added. After 15 minutes, **2f** (990 mg, 1.84 mmol, 1.00 equiv.) and DMAP (337 mg, 2.76 mmol, 1.50 equiv.) were added. The reaction mixture was allowed to warm to r.t. and stirred overnight and subsequently concentrated *in vacuo*. The crude material was purified by column chromatography (cHex/EtOAc 1:1) to give compound **11f** as white foam (935 mg, 1.32 mmol, 72 %).

HR-MS (ESI): m/z calculated for $C_{32}H_{57}N_6O_8Si_2^+$: 709.3771; found: 709.3773.

^{1}H-NMR (400 MHz, CDCl$_3$): δ [ppm] = 8.23 (s, 1H, H^1), 7.84 (s, 1H, H^2), 5.98 (s, 1H, H^3), 5.88 (d, *J* = 5.3 Hz, 1H, H^4), 5.23 (dd, *J* = 8.7, 5.3 Hz, 1H, H^5), 5.11 (d, *J* = 8.0 Hz, 1H, H^6), 4.45 (p, *J* = 7.4 Hz, 1H, H^7), 4.15 (dd, *J* = 13.9, 3.6 Hz, 1H, H^{8a}), 4.04 – 3.95 (m, 2H, $H^{8b,9}$), 3.49 (s, 6H, H^{10}), 1.42 (d, *J* = 3.5 Hz, 12H, $H^{11,Boc}$), 1.14 – 0.87 (m, 28H, H^{iPr}).

^{13}C{^{1}H}-NMR (100 MHz, CDCl$_3$): δ [ppm] = 172.3 (C^{12}), 155.1 ($C^{Boc\text{-}CO}$), 155.0 (C^{13}), 152.6 (C^1), 149.8 (C^{14}), 137.2 (C^2), 120.9 (C^{15}), 87.6 (C^3), 81.8 (C^9), 80.0 ($C^{Boc\text{-}quart.}$), 76.3 (C^4), 69.1 (C^5), 60.3 (C^8), 49.3 (C^7), 38.7 (C^{10}), 28.4 ($C^{Boc\text{-}CH3}$), 19.3 (C^{11}), 17.5 – 16.9 (8C, $C^{iPr\text{-}CH3}$), 13.4 – 12.6 (4C, $C^{iPr\text{-}CH}$).

R$_f$: 0.61 (cHex/EtOAc 1:1).

(2*R*,3*S*,4*R*,5*R*)-5-(6-(dimethylamino)-9*H*-purin-9-yl)-4-hydroxy-2-(hydroxymethyl)tetrahydrofuran-3-yl (*tert*-butoxycarbonyl)-L-alaninate (**12f**)

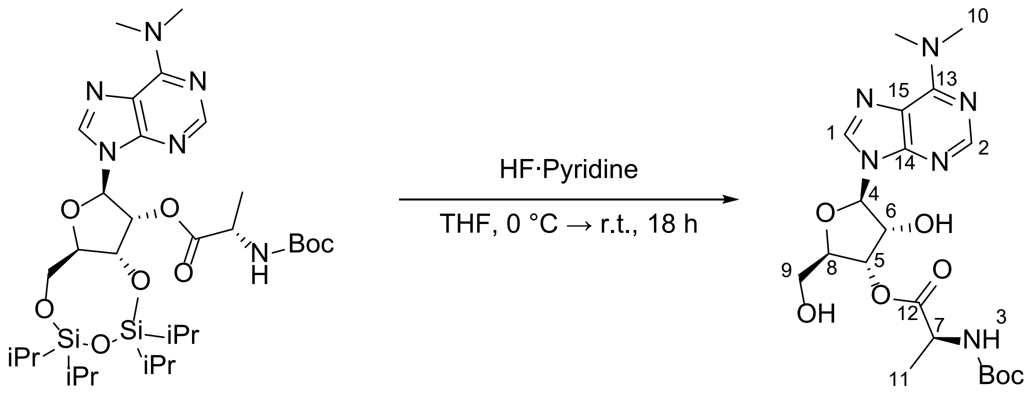

11f **12f**

11f (150 mg, 212 µmol, 1.00 equiv.) was dissolved in THF (1 mL). The reaction was cooled to 0 °C followed by the addition of HF·pyridine (70 wt.%; 50.0 µL, 1.90 mmol, 9.00 equiv.). The reaction was allowed to warm to r.t. overnight. HF·pyridine (25.0 µL, 950 µmol, 4.50 equiv.) was added again and the reaction was further stirred for 5 h at r.t. The reaction was quenched with MeO-TMS (1 mL) followed by additional 30 min of stirring. Concentration of the reaction *in vacuo* and purification by flash column chromatography (EtOAc 100 % → EtOAc/MeOH 90:10) yielded compound **12f** as white foam (54.0 mg, 116 µmol, 55 %).

HR-MS (ESI): m/z calculated for $C_{20}H_{31}N_6O_7^+$: 467.2249; found: 467.2253.

^{1}H-NMR (400 MHz, DMSO): δ [ppm] = 8.43 (s, 1H, H^1), 8.24 (s, 1H, H^2), 7.35 (d, *J* = 7.5 Hz, 1H, H^3), 5.96 (d, *J* = 7.2 Hz, 1H, H^4), 5.26 (dd, *J* = 5.3, 2.0 Hz, 1H, H^5), 4.88 (dd, *J* = 7.3, 5.3 Hz, 1H, H^6), 4.15 (p, *J* = 7.3 Hz, 1H, H^7), 4.06 (q, *J* = 3.1 Hz, 1H, H^8), 3.64 (ddd, *J* = 35.7, 12.3, 3.6 Hz, 2H, H^9), 3.55 – 3.22 (m, 6H, H^{10}), 1.40 (s, 9H, H^{Boc}), 1.31 (d, *J* = 4.9 Hz, 3H, H^{11}).

^{13}C{^{1}H}-NMR (100 MHz, DMSO): δ [ppm] = 172.3 (C^{12}), 155.4 ($C^{Boc\text{-}CO}$), 154.0 (C^{13}), 151.4 (C^2), 149.9 (C^{14}), 138.6 (C^1), 119.7 (C^{15}), 87.2 (C^4), 83.7 (C^8), 78.3 ($C^{Boc\text{-}quart.}$), 73.8 (C^5), 71.9 (C^6), 61.5 (C^9), 49.3 (C^7), 28.2 ($C^{Boc\text{-}CH3}$), 17.2 (C^{11}).

R_f: 0.57 (EtOAc).

(6a*R*,8*R*,9*R*,9a*R*)-8-(6-(dimethylamino)-9*H*-purin-9-yl)-2,2,4,4-tetraisopropyltetrahydro-6*H*-furo[3,2-*f*][1,3,5,2,4]trioxadisilocin-9-yl acetyl-L-alaninate (**13f**)

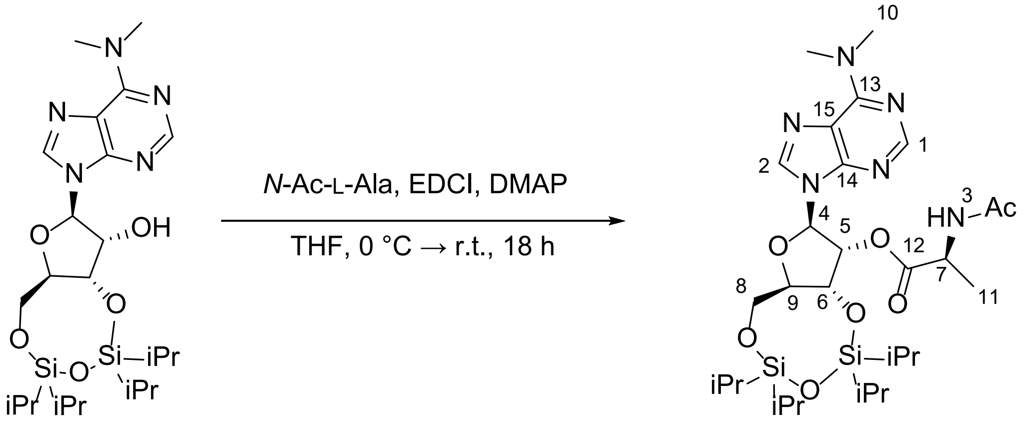

2f **13f**

N-Ac-L-Alanine (244 mg, 1.86 mmol, 1.00 equiv.) was dissolved in dry THF (20 mL). The mixture was cooled to 0 °C and EDCI·HCl (392 mg, 2.05 mmol, 1.10 equiv.) was added. After 15 minutes, **2f** (1.00 g, 1.84 mmol, 1.00 equiv.) and DMAP (341 mg, 2.79 mmol, 1.50 equiv.) were added. The reaction mixture was allowed to warm to r.t. and stirred overnight and was subsequently concentrated *in vacuo*. The crude material was purified by column chromatography (EtOAc 100 %) to give compound **13f** as white foam (816 mg, 1.25 mmol, 68 %).

HR-MS (ESI): m/z calculated for $C_{29}H_{51}N_6O_7Si_2^+$: 651.3352; found 651.3358.

^{1}H-NMR (400 MHz, $CDCl_3$): δ [ppm] = 8.25 (s, 1H, H^1), 7.86 (s, 1H, H^2), 6.10 (d, *J* = 7.4 Hz, 1H, H^3), 5.99 (d, *J* = 0.9 Hz, 1H, H^4), 5.88 (d, *J* = 5.3 Hz, 1H, H^5), 5.21 (dd, *J* = 8.8, 5.3 Hz, 1H, H^6), 4.75 (p, *J* = 7.2 Hz, 1H, H^7), 4.21 – 4.11 (m, 1H, H^{8a}), 4.05 – 3.95 (m, 2H, $H^{8b,9}$), 3.53 – 3.48 (m, 6H, H^{10}), 2.01 (s, 3H, $H^{Ac\text{-}CH3}$), 1.47 (d, *J* = 7.2 Hz, 3H, H^{11}), 1.13 – 0.91 (m, 28H, H^{iPr}).

^{13}C{^{1}H}-NMR (100 MHz, $CDCl_3$): δ [ppm] = 172.1 (C^{12}), 169.7 ($C^{Ac\text{-}CO}$), 155.1 (C^{13}), 152.7 (C^1), 149.8 (C^{14}), 137.1 (C^2), 120.9 (C^{15}), 87.5 (C^4), 81.9 (C^9), 69.0 (C^6), 60.3 (C^8), 48.2 (C^7), 38.8 (C^{10}), 23.3 ($C^{Ac\text{-}CH3}$), 19.2 (C^{11}), 17.6 – 16.9 (8C, $C^{iPr\text{-}CH3}$), 13.5 – 12.6 (4C, $C^{iPr\text{-}CH}$).

R_f: 0.29 (EtOAc 100 %).

(2*R*,3*S*,4*R*,5*R*)-5-(6-(dimethylamino)-9*H*-purin-9-yl)-4-hydroxy-2-(hydroxymethyl)tetrahydrofuran-3-yl acetyl-L-alaninate (**S09**)

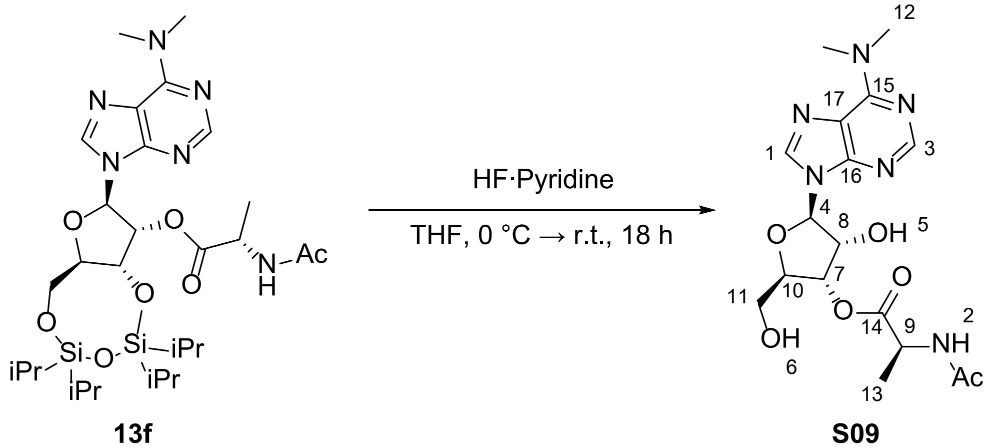

13f **S09**

13f (231 mg, 355 µmol, 1.00 equiv.) was dissolved in THF (2.1 mL). The reaction was cooled to 0 °C. HF·pyridine (180 µL, 7.11 mmol, 20.0 equiv.) was added. The reaction was allowed to warm to r.t. and stirred for 18 h. The reaction was quenched with MeO-TMS (3 mL). The reaction was concentrated *in vacuo* and purified by column chromatography (EtOAc/MeOH 98:2) to give compound **S09** as white foam (142 mg, 348 µmol, 98 %).

HR-MS (ESI): m/z calculated for $C_{17}H_{25}N_6O_6^+$: 409.1830; found: 409.1831.

^{1}H-NMR (400 MHz, DMSO: δ [ppm] = 8.40 (s, 1H, H^1), 8.35 (d, J = 7.1 Hz, 1H, H^2), 8.23 (s, 1H, H^3), 5.93 (d, J = 7.1 Hz, 1H, H^4), 5.75 (d, J = 6.3 Hz, 1H, H^5), 5.62 (dd, J = 7.4, 4.7 Hz, 1H, H^6), 5.31 – 5.23 (m, 1H, H^7), 4.91 (p, J = 5.9 Hz, 1H, H^8), 4.40 (t, J = 7.2 Hz, 1H, H^9), 4.09 (q, J = 2.9 Hz, 1H, H^{10}), 3.74 – 3.52 (m, 2H, H^{11}), 3.47 (s, 6H, H^{12}), 1.88 (s, 3H, H^{Ac}), 1.35 (d, J = 7.2 Hz, 3H, H^{13}).

^{13}C{^{1}H}-NMR (100 MHz, DMSO): δ [ppm] = 172.0 (C^{14}), 169.3 ($C^{Ac\text{-}CO}$), 154.4 (C^{15}), 151.8 (C^3), 150.0 (C^{16}), 138.6 (C^1), 119.8 (C^{17}), 87.4 (C^4), 83.6 (C^{10}), 73.9 (C^7), 71.7 (C^8), 61.5 (C^{11}), 47.7 (C^9), 22.3 ($C^{Ac\text{-}CH3}$), 17.3 (C^{13}).

R_f: 0.07 (EtOAc/MeOH 98:2).

Nebularine-based Compounds

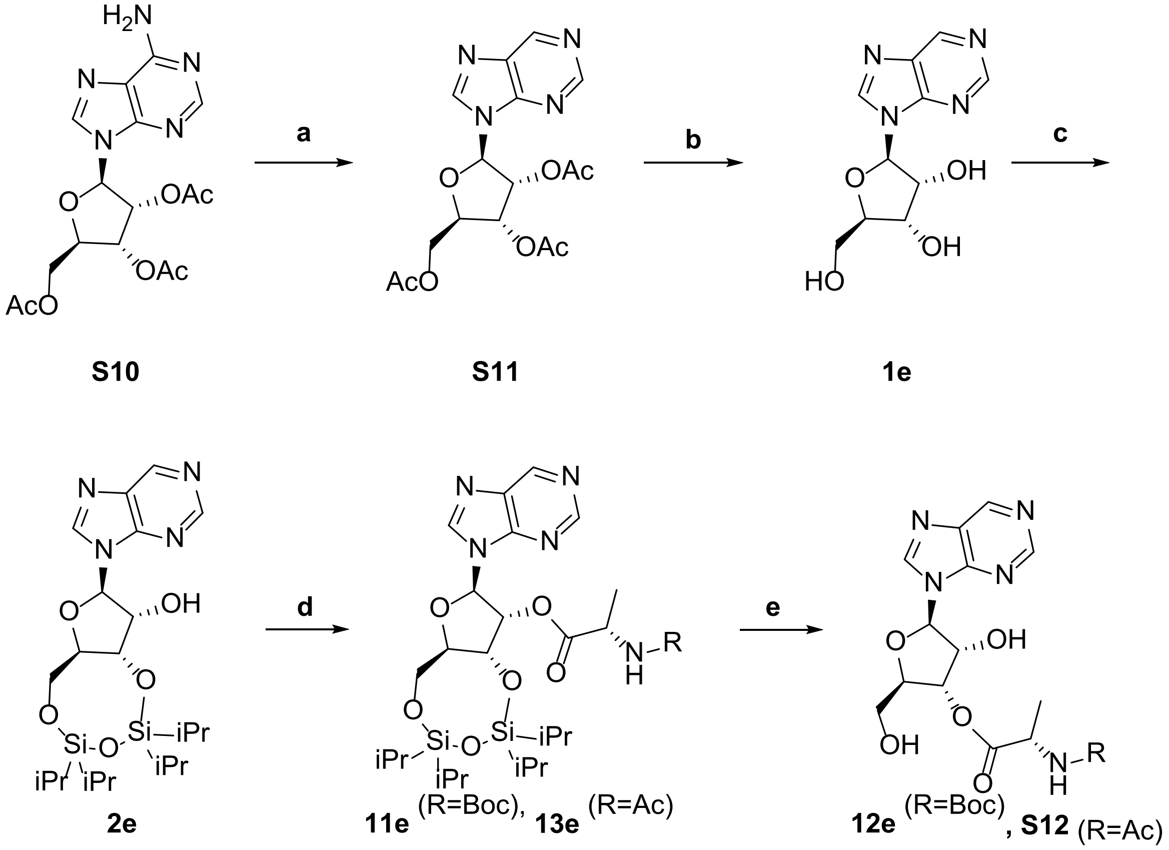

Scheme 2. Synthesis of the 3’-*N*-protected-alanine-nebularines (**12e** and **S12**). Conditions: a) TBN, THF, 53 % b) NH_3 in MeOH, 81 % c) $TIPDSCl_2$, pyridine, 85 % d) Boc-N-Ala-OH, DMAP, EDCI, THF, up to 75°% e) HF·pyridine, THF, up to 98 %. R = Boc or Ac.

(2*R*,3*R*,4*R*,5*R*)-2-(acetoxymethyl)-5-(9*H*-purin-9-yl)tetrahydrofuran-3,4-diyl diacetate (**S11**)

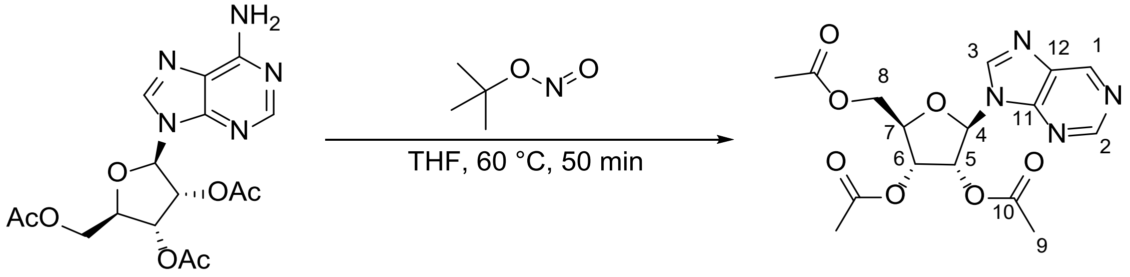

S10 **S11**

Ac-Nebularine was prepared similarly to HERBERT *et al.*[2]

Acetyl-protected adenosine **S10** (3.93 g, 10.0 mmol, 1.00 equiv.) was dissolved in dry THF (120 mL) at 60 °C. TBN (23.8 mL, 200 mmol, 20.0 equiv.) was added and the yellow solution gradually turned red. The solution was stirred at 60 °C for 50 min before concentration of the reaction *in vacuo*. The crude product was purified by column chromatography (EtOAc 100 % → EtOAc/MeOH 98:2) yielding Ac-nebularine **S11** (4.00 g, 5.30 mmol, 53%) as a white solid.

HR-MS (ESI): m/z calculated for $C_{16}H_{19}N_4O_7^+$: 379.1248; found: 379.1247.

^{1}H-NMR (400 MHz, $CDCl_3$): δ [ppm] = 9.17 (d, *J* = 1.7 Hz, 1H, H^1), 9.00 (d, *J* = 1.6 Hz, 1H, H^2), 8.25 (s, 1H, H^3), 6.25 (dd, *J* = 5.1, 1.0 Hz, 1H, H^4), 5.97 (td, *J* = 5.3, 1.1 Hz, 1H, H^5), 5.68 (td, *J* = 5.1, 1.3 Hz, 1H, H^6), 4.50 – 4.46 (m, 1H, H^7), 4.45 – 4.32 (m, 2H, H^8), 2.15 (s, *J* = 1.5 Hz, 3H, H^9), 2.11 (s, *J* = 1.6 Hz, 3H, H^9), 2.07 (s, *J* = 1.5 Hz, 3H, H^9).

^{13}C{^{1}H}-NMR (100 MHz, $CDCl_3$): δ [ppm] = 170.4 (C^{10}), 169.7 (C^{10}), 169.5 (C^{10}), 153.0 (C^2), 151.0 (C^{11}), 149.3 (C^1), 143.7 (C^3), 134.8 (C^{12}), 86.5 (C^4), 80.5 (C^7), 73.2 (C^5), 70.6 (C^6), 63.1 (C^8), 20.9 (C^9), 20.7 (C^9), 20.5 (C^9).

R_f: 0.14 (EtOAc).

(2*R*,3*S*,4*R*,5*R*)-2-(hydroxymethyl)-5-(9*H*-purin-9-yl)tetrahydrofuran-3,4-diol (**1e**)

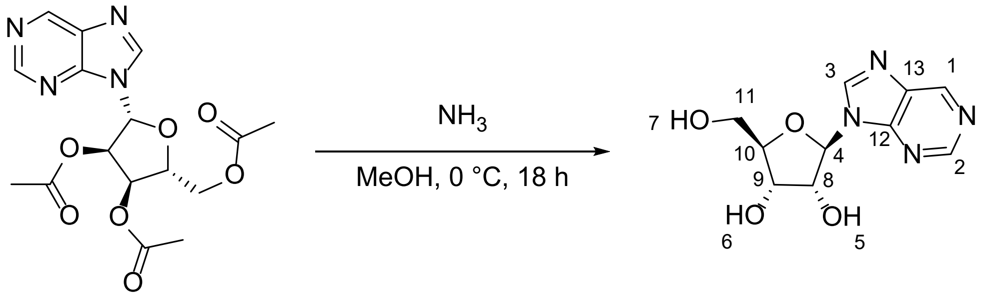

S11 **1e**

Deacetylation was conducted similarly to the synthesis described by Shi *et al.*[3]

Ac-Nebularine **S11** (3.99 g, 10.6 mmol, 1.00 equiv.) was suspended in NH_3/methanol (105 mL, 7 M) and stored at 0 °C overnight. The solvent was removed *in vacuo*. After washing with EtOH and recrystallization (EtOH), nebularine **1e** was obtained as an off-white solid (3.54 g, 8.59 mmol, 81 %).

HR-MS (ESI): m/z calculated for $C_{10}H_{13}N_4O_4^+$: 253.0931; found: 253.0929.

^{1}H-NMR (400 MHz, DMSO): δ [ppm] = 9.21 (s, 1H, H^1), 8.97 (s, 1H, H^2), 8.87 (s, 1H, H^3), 6.06 (d, *J* = 5.7 Hz, 1H, H^4), 5.56 (d, *J* = 6.0 Hz, 1H, H^5), 5.27 (d, *J* = 5.0 Hz, 1H, H^6), 5.11 (t, *J* = 5.5 Hz, 1H, H^7), 4.64 (q, *J* = 5.5 Hz, 1H, H^8), 4.19 (q, *J* = 4.7 Hz, 1H, H^9), 3.98 (q, *J* = 3.9 Hz, 1H, H^{10}), 3.69 (dt, *J* = 12.0, 4.4 Hz, 1H, H^{11a}), 3.58 (ddd, *J* = 12.0, 5.9, 4.0 Hz, 1H, H^{11b}).

^{13}C{^{1}H}-NMR (100 MHz, DMSO): δ [ppm] = 152.2 (C^2), 151.0 (C^{12}), 148.3 (C^1), 145.5 (C^3), 134.2 (C^{13}), 87.5 (C^4), 85.7 (C^{10}), 73.7 (C^8), 70.3 (C^9), 61.3 (C^{11}).

(6a*R*,8*R*,9*R*,9a*S*)-2,2,4,4-tetraisopropyl-8-(9*H*-purin-9-yl)tetrahydro-6*H*-furo[3,2-*f*][1,3,5,2,4]trioxadisilocin-9-ol (**2e**)

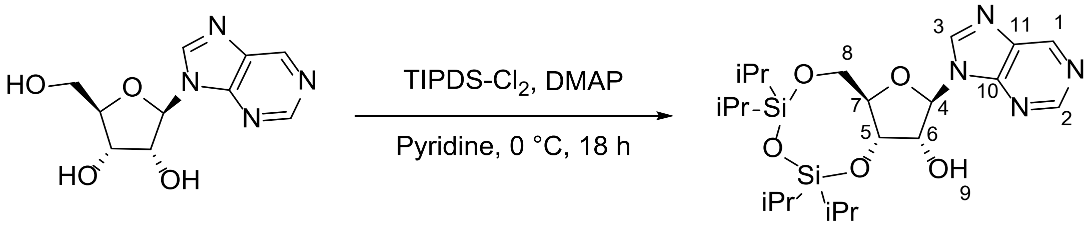

1e **2e**

Nebularine **1e** (2.09 g, 8.30 mmol, 1.00 equiv.) and DMAP (0.51 g, 4.10 mmol, 0.500 equiv.) were suspended in dry pyridine (21 mL) and the mixture was cooled to 0 °C. TIPDS-Cl_2 (3.80 mL, 9.94 mmol, 1.20 equiv.) was added dropwise and the mixture was slowly warmed to r.t. overnight. Cold water (10 mL) was added once TLC indicated full conversion and all volatiles were removed under reduced pressure. The residue was purified *via* column chromatography (EtOAc 100%) to yield 3',5'-TIPDS-nebularine **2e** (3.88 g, 7.89 mmol, 95 %) as an orange oil.

HR-MS (ESI): m/z calculated for $C_{22}H_{39}N_4O_5Si_2^+$: 495.2453; found: 495.2455.

^{1}H-NMR (400 MHz, $CDCl_3$): δ [ppm] = 9.15 (s, 1H, H^1), 8.93 (s, 1H, H^2), 8.26 (s, 1H, H^3), 6.06 (d, *J* = 1.3 Hz, 1H, H^4), 5.15 – 5.06 (m, 1H, H^5), 4.64 (d, *J* = 5.5 Hz, 1H, H^6), 4.17 – 4.09 (m, 2H, $H^{7,8a}$), 4.09 – 4.00 (m, 1H, H^{8b}), 3.19 (s, 1H, H^9), 1.13 (d, *J* = 9.8 Hz, 4H, $H^{iPr\text{-}CH}$), 1.13 – 0.97 (m, 24H, $H^{iPr\text{-}CH3}$).

^{13}C{^{1}H}-NMR (100 MHz, $CDCl_3$): δ [ppm] = 152.8 (C^2), 150.6 (C^{10}), 149.2 (C^1), 144.5 (C^3), 135.1 (C^{11}), 89.7 (C^4), 82.3 (C^7), 75.1 (C^6), 70.7 (C^5), 61.6 (C^8), 17.6 – 17.1 (8C, $C^{iPr\text{-}CH3}$), 13.4 – 12.7 (4C, $C^{iPr\text{-}CH}$).

R_f: 0.55 (EtOAc 100 %).

(6a*R*,8*R*,9*R*,9a*R*)-2,2,4,4-tetraisopropyl-8-(9*H*-purin-9-yl)tetrahydro-*6*H-furo[3,2-*f*][1,3,5,2,4]trioxadisilocin-9-yl (*tert*-butoxycarbonyl)-L-alaninate (**11e**)

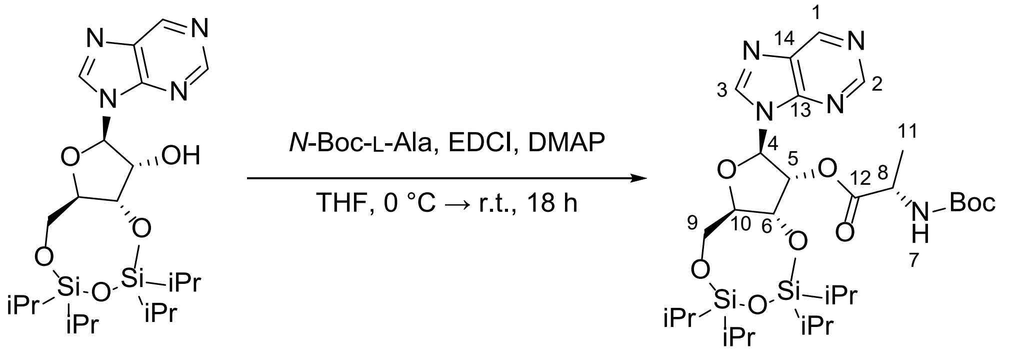

2e **11e**

N-Boc-L-Alanine (382 mg, 2.02 mmol, 1.00 equiv.) was dissolved in dry THF (20 mL). The mixture was cooled to 0 °C and EDCI·HCl (426 mg, 2.22 mmol, 1.10 equiv.) was added. After 15 minutes, **2e** (1.00 g, 2.02 mmol, 1.00 equiv.) and DMAP (370 mg, 3.03 mmol, 1.50 equiv.) were added. The reaction mixture was allowed to warm to r.t., further stirred overnight and subsequently concentrated *in vacuo*. The crude material was purified by column chromatography (cHex/EtOAc 1:1) to give compound **11e** as white foam (1.01 g, 1.52 mmol, 75 %).

HR-MS (ESI): m/z calculated for $C_{30}H_{52}N_5O_8Si_2^+$: 666.3349; found: 666.3355.

^{1}H-NMR (400 MHz, $CDCl_3$): δ [ppm] = 9.16 (s, 1H, H^1), 8.92 (s, 1H, H^2), 8.27 (s, 1H, H^3), 6.10 (s, $1H^4$), 5.95 (d, J = 5.3 Hz, 1H, H^5), 5.20 (dd, J = 9.1, 5.3 Hz, 1H, H^6), 5.07 (d, J = 8.0 Hz, 1H, H^7), 4.48 (p, J = 7.2 Hz, 1H, H^8), 4.19 (d, J = 12.9 Hz, 1H, H^{9a}), 4.08 – 4.00 (m, 2H, $H^{9b,10}$), 1.48 – 1.41 (m, 12H, H^{Boc}), 1.13 – 0.99 (m, 29H, H^{iPr}).

^{13}C{^{1}H}-NMR (100 MHz, $CDCl_3$): δ [ppm] = 172.4 (C12), 155.2 ($C^{Boc\text{-}CO}$), 152.9 (C2), 150.5 (C^{13}), 149.2 (C^1), 144.4 (C^3), 135.0 (C^{14}), 87.8 (C^4), 82.0 (C^{10}), 80.2 ($C^{Boc\text{-}quart.}$), 75.9 (C^5), 68.6 (C^6), 59.9 (C^9) 49.3 (C^8), 28.4 ($C^{Boc\text{-}CH3}$), 19.2 (C^{11}), 17.6 – 16.9 (8C, $C^{iPr\text{-}CH3}$), 13.5 – 12.6 (4C, $C^{iPr\text{-}CH}$).

R_f: 0.63 (EtOAc 100 %).

(2*R*,3*S*,4*R*,5*R*)-4-hydroxy-2-(hydroxymethyl)-5-(9*H*-purin-9-yl)tetrahydrofuran-3-yl (*tert*-butoxycarbonyl)-L-alaninate (**12e**)

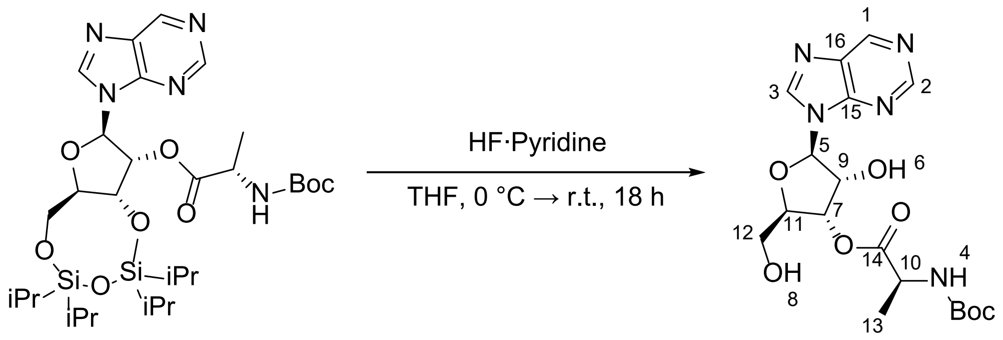

11e **12e**

11e (150 mg, 225 µmol, 1.00 equiv.) was dissolved in THF (1 mL). The reaction was cooled to 0 °C and HF·pyridine (70 wt.%; 0.053 mL, 2.03 mmol, 9.00 equiv.) was added. The reaction was allowed to warm up to r.t. and stirred for 18 h before it was quenched with MeO-TMS (1 mL). It was concentrated *in vacuo* and purified by column chromatography (EtOAc 100 %→ EtOAc/MeOH 98:2) yielding compound **12e** as white foam (98.2 mg, 221 µmol, 98 %).

HR-MS (ESI): m/z calculated for $C_{18}H_{26}N_5O_7^+$: 424.1827; found: 424.1828.

^{1}H-NMR (400 MHz, DMSO): δ [ppm] = 9.23 (s, 1H, H^1), 8.99 (s, 1H, H^2), 8.88 (s, 1H, H^3), 7.35 (d, *J* = 7.5 Hz, 1H, H^4), 6.10 (d, *J* = 7.0 Hz, 1H, H^5), 5.83 (d, *J* = 6.4 Hz, 1H, H^6), 5.31 (dd, *J* = 5.5, 2.2 Hz, 2H, $H^{7,8}$), 4.96 (t, *J* = 6.2 Hz, 1H, H^9), 4.17 (q, *J* = 7.3 Hz, 1H, H^{10}), 4.12 – 4.08 (m, 1H, H^{11}), 3.74 – 3.57 (m, 2H, H^{12}), 1.41 (s, 6H, H^{Boc}), 1.33 (d, *J* = 7.3 Hz, 3H, H^{13}).

^{13}C{^{1}H}-NMR (100 MHz, DMSO): δ [ppm] = 172.3 (C^{14}), 155.3 ($C^{Boc\text{-}CO}$), 152.3 (C^2), 151.1 (C^{15}), 148.4 (C^1), 145.2 (C^3), 134.1 (C^{16}), 86.8 (C^5), 83.7 (C^{11}), 78.3 ($C^{Boc\text{-}quart.}$), 73.6 (C^7), 72.1 (C^9), 61.2 (C^{12}), 49.3 (C^{10}), 28.1 ($C^{Boc\text{-}CH3}$), 17.1 (C^{13}).

R$_f$: 0.20 (EtOAc/MeOH 98:2).

(6a*R*,8*R*,9*R*,9a*R*)-2,2,4,4-tetraisopropyl-8-(9*H*-purin-9-yl)tetrahydro-6*H*-furo[3,2-*f*][1,3,5,2,4]trioxadisilocin-9-yl acetyl-L-alaninate (**13e**)

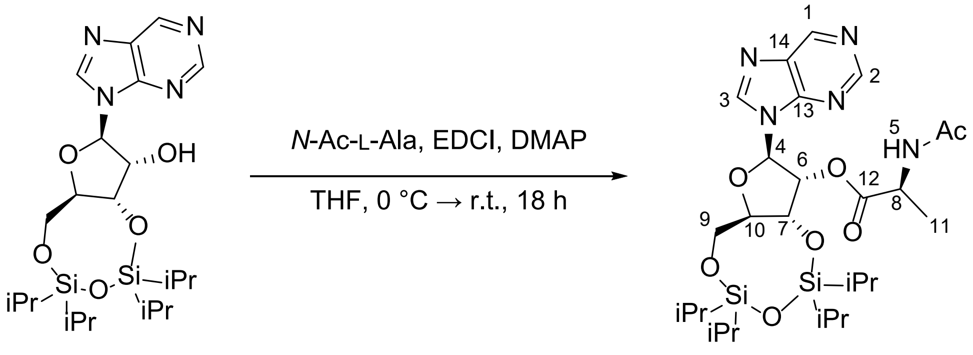

2e **13e**

N-Ac-L-Alanine (268 mg, 2.05 mmol, 1.00 equiv.) was dissolved in dry THF (20 mL). The mixture was cooled to 0 °C and EDCI·HCl (432 mg, 2.25 mmol, 1.10 equiv.) was added. After 15 minutes, **2e** (1.01 g, 2.05 mmol, 1.00 equiv.) and DMAP (375 mg, 3.07 mmol, 1.50 equiv.) were added. The reaction mixture was allowed to warm to r.t. and stirred overnight before it was subsequently concentrated *in vacuo*. The crude material was purified by column chromatography (EtOAc/MeOH 98:2) to give compound **13e** as white foam (730 mg, 1.54 mmol, 75 %).

HR-MS (ESI): m/z calculated for $C_{27}H_{46}N_5O_7Si_2^+$: 608.2930; found: 608.2935.

^{1}H-NMR (400 MHz, $CDCl_3$): δ [ppm] = 9.16 (s, 1H, H^1), 8.93 (s, 1H, H^2), 8.30 (s, 1H, H^3), 6.11 (d, *J* = 0.8 Hz, 1H, H^4), 6.03 (d, *J* = 7.4 Hz, 1H, H^5), 5.94 (d, *J* = 5.2 Hz, 1H, H^6), 5.22 – 5.12 (m, 1H, H^7), 4.83 – 4.71 (m, 1H, H^8), 4.25 – 4.15 (m, 1H, H^{9a}), 4.09 – 3.99 (m, 2H, $H^{9b,10}$), 2.02 (d, *J* = 1.4 Hz, 3H, $H^{Ac\text{-}CH3}$), 1.50 (dd, *J* = 8.9, 7.1 Hz, 3H, H^{11}), 1.13 – 0.98 (m, 28H, H^{iPr}).

^{13}C{^{1}H}-NMR (100 MHz, $CDCl_3$): δ [ppm] = 172.2 (C^{12}), 169.7 ($C^{Ac\text{-}CO}$), 152.9 (C^2), 150.5 (C^{13}), 149.2 (C^1), 144.3 (C^3), 135.0 (C^{14}), 87.7 (C^4), 82.0 (C^{10}), 76.1 (C^6), 68.6 (C^7), 59.8 (C^9), 48.2 (C^8), 23.3 ($C^{Ac\text{-}CH3}$), 19.1 (C^{11}), 17.5 – 16.9 (8C, $C^{iPr\text{-}CH3}$), 13.5 – 12.6 (4C, $C^{iPr\text{-}CH}$).

R_f: 0.14 (EtOAc/MeOH 98:2).

(2*R*,3*S*,4*R*,5*R*)-4-hydroxy-2-(hydroxymethyl)-5-(9*H*-purin-9-yl)tetrahydrofuran-3-yl acetyl-L-alaninate (**S12**)

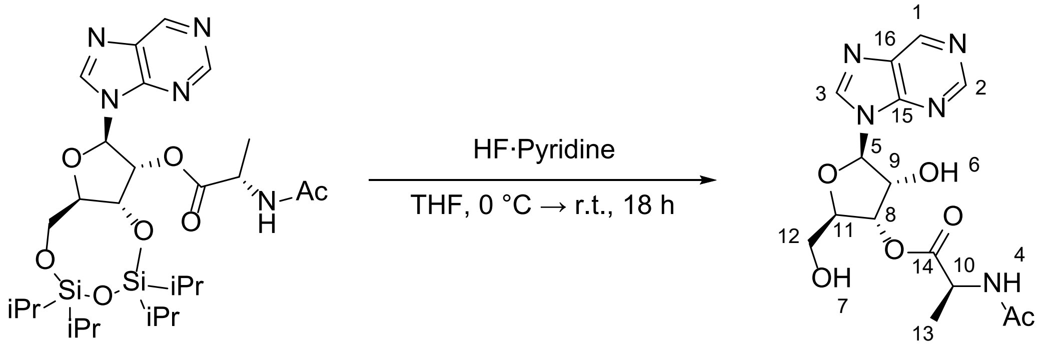

13e → **S12**

13e (271 mg, 445 µmol, 1.00 equiv.) was dissolved in THF (2.7 mL). The reaction was cooled down to 0 °C and HF·pyridine (230 µL, 8.91 mmol, 20.0 equiv.) was added. The reaction was allowed to warm up to r.t. and stirred for 18 h. The reaction was quenched with MeO-TMS (3 mL) and all volatiles were removed under reduced pressure. Purification using column chromatography (EtOAc/MeOH 98:2) yielded compound **S12** as white foam (154 mg, 418 µmol, 94 %).

HR-MS (ESI): m/z calculated for $C_{15}H_{20}N_5O_6^+$: 366.1408; found: 366.1410.

^{1}H-NMR (400 MHz, DMSO): δ [ppm] = 9.24 (s, 1H, H^1), 8.99 (s, 1H, H^2), 8.89 (s, 1H, H^3), 8.37 (d, *J* = 7.2 Hz, 1H, H^4), 6.08 (d, *J* = 6.9 Hz, 1H, H^5), 5.84 (d, *J* = 6.1 Hz, 1H, H^6), 5.32 (td, *J* = 5.1, 2.3 Hz, 2H, $H^{7,8}$), 4.98 (dq, *J* = 11.7, 5.7 Hz, 1H, H^9), 4.47 – 4.32 (m, 1H, H^{10}), 4.12 (q, *J* = 3.7 Hz, 2H, H^{11}), 3.78 – 3.66 (m, 1H, H^{12a}), 3.61 (dq, *J* = 12.3, 6.2 Hz, 1H, H^{12b}), 1.90 – 1.86 (m, 4H, H^{Ac}), 1.36 (d, *J* = 7.3 Hz, 3H, H^{13}).

^{13}C{^{1}H}-NMR (100 MHz, DMSO): δ [ppm] = 171.9 (C^{14}), 169.3 ($C^{Ac\text{-}CO}$), 152.3 (C^2), 151.1 (C^{15}), 148.5 (C^1), 145.3 (C^3), 134.2 (C^{16}), 87.0 (C^5), 83.6 (C^{11}), 73.6 (C^8), 72.1 (C^9), 61.2 (C^{12}), 47.8 (C^{10}), 22.3 ($C^{Ac\text{-}CH3}$), 17.3 (C^{13}).

R_f: 0.07 (EtOAc/MeOH 98:2).

NMR Spectra

1e

1H-NMR (DMSO)

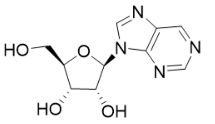

1e

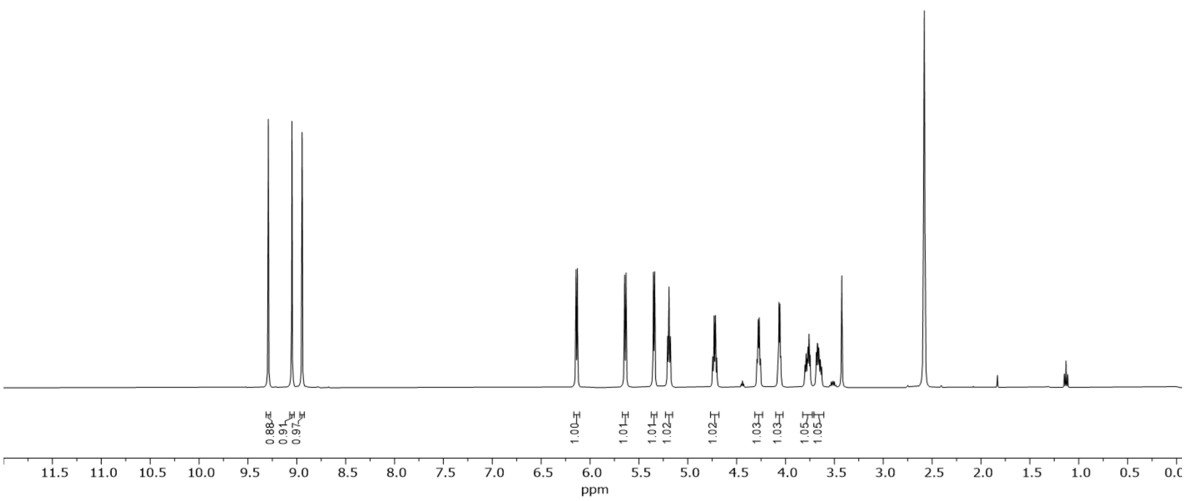

13C-NMR (DMSO)

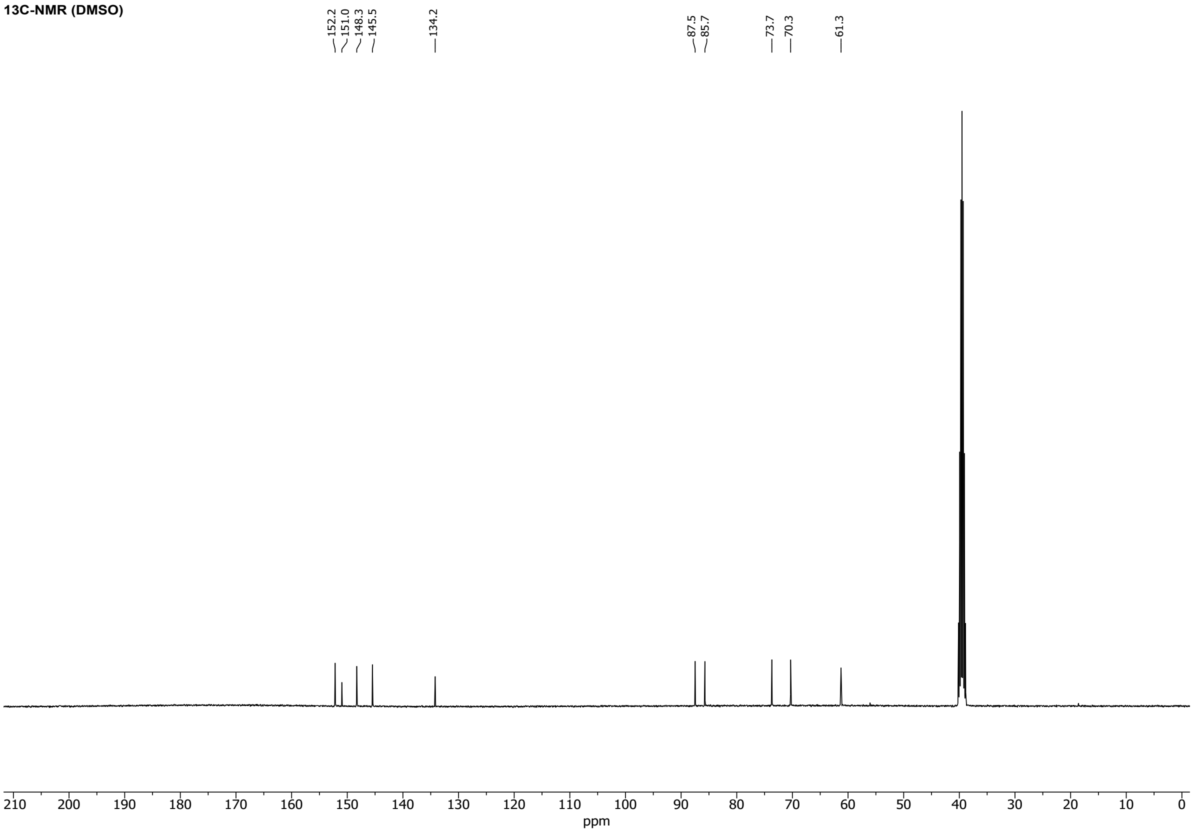

1f

1H-NMR (DMSO)

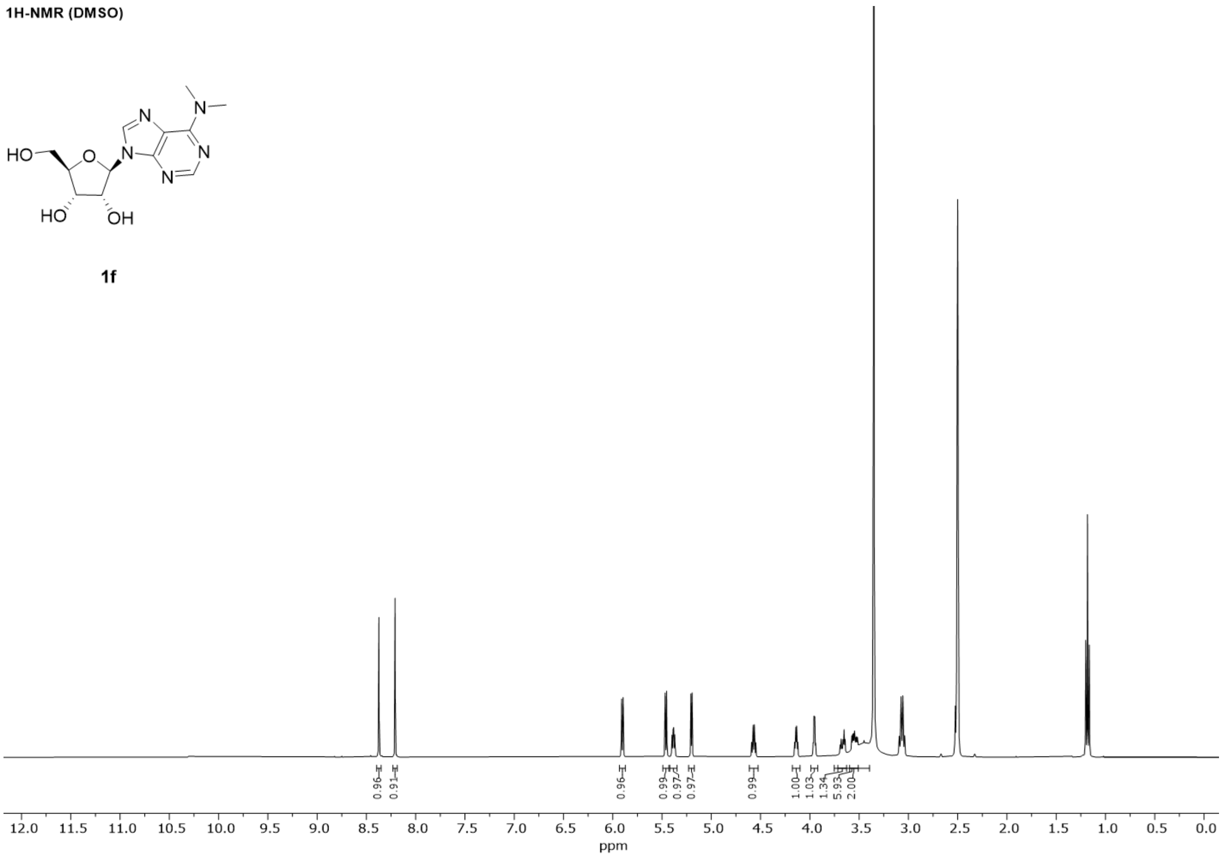

13C-NMR (DMSO)

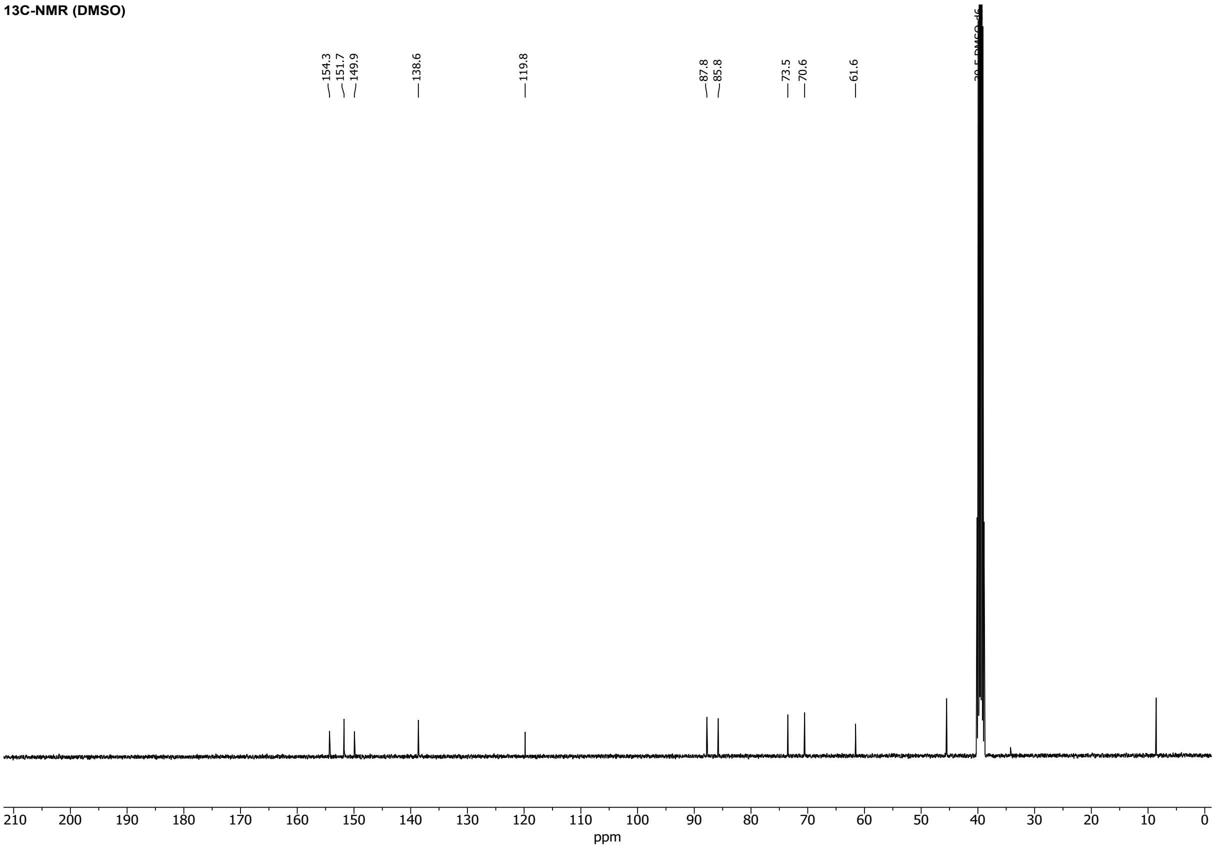

2a

1H-NMR (DMSO)

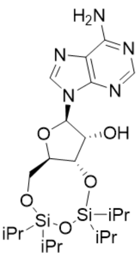

2a

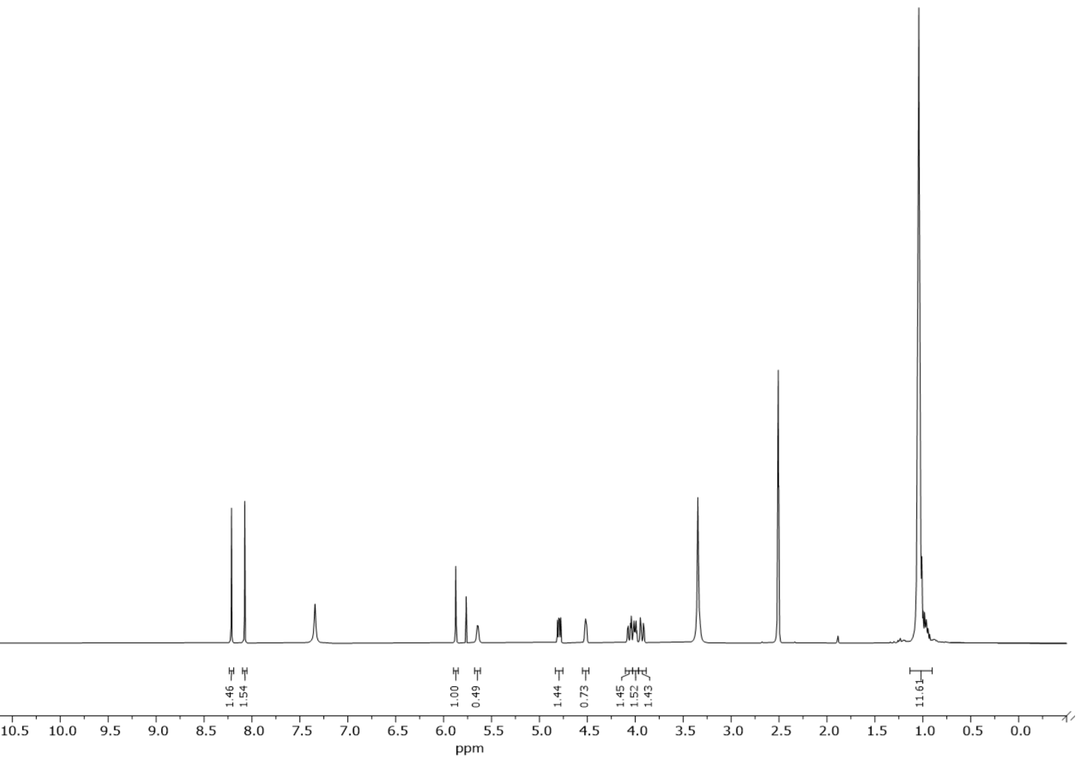

13C-NMR (DMSO)

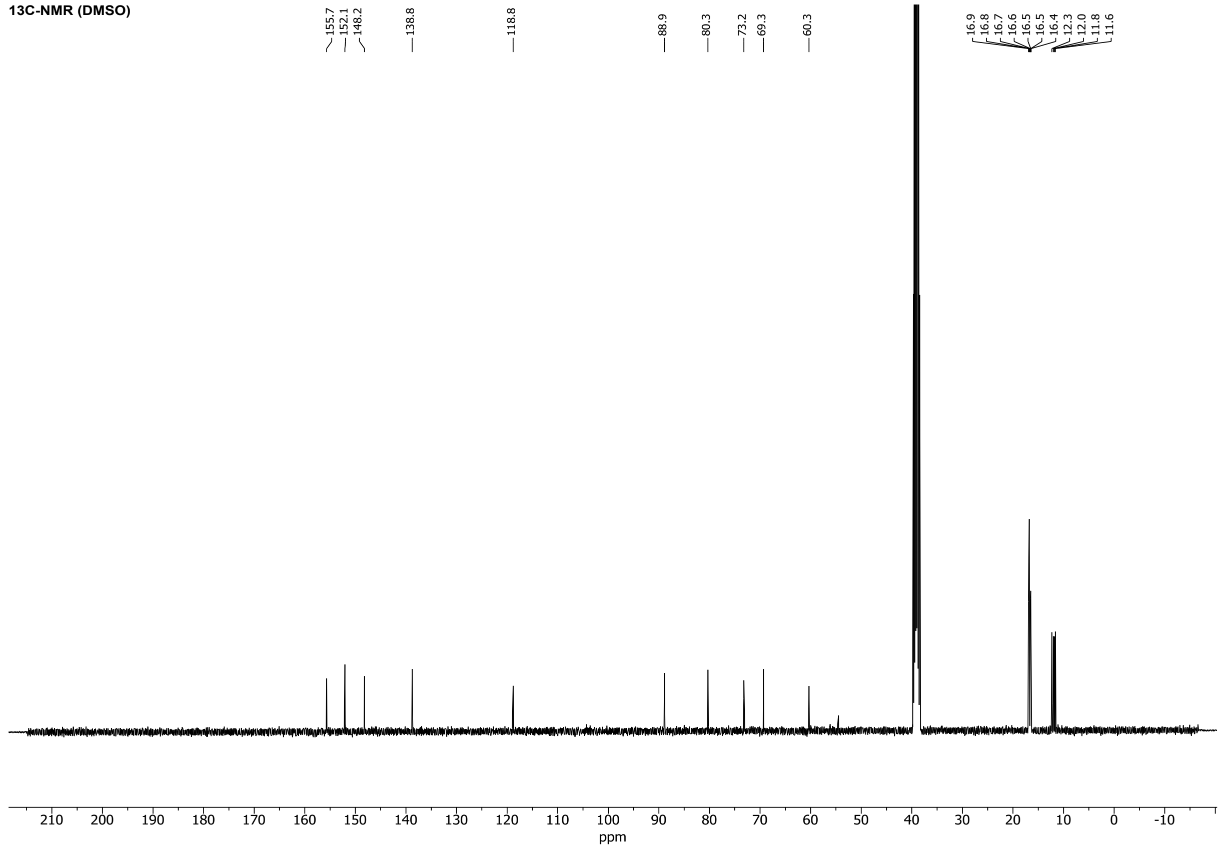

2b

1H-NMR (DMSO)

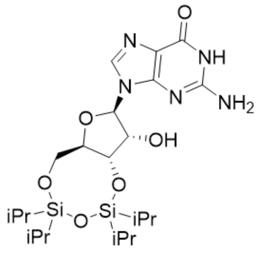

2b

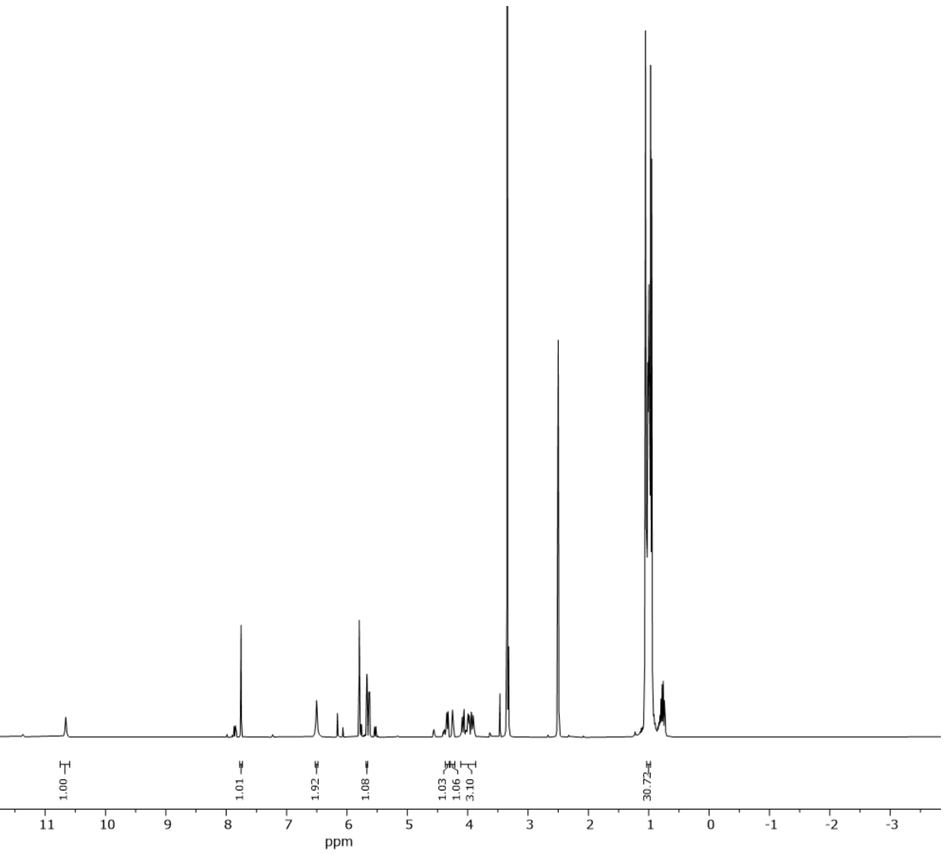

13C-NMR (DMSO)

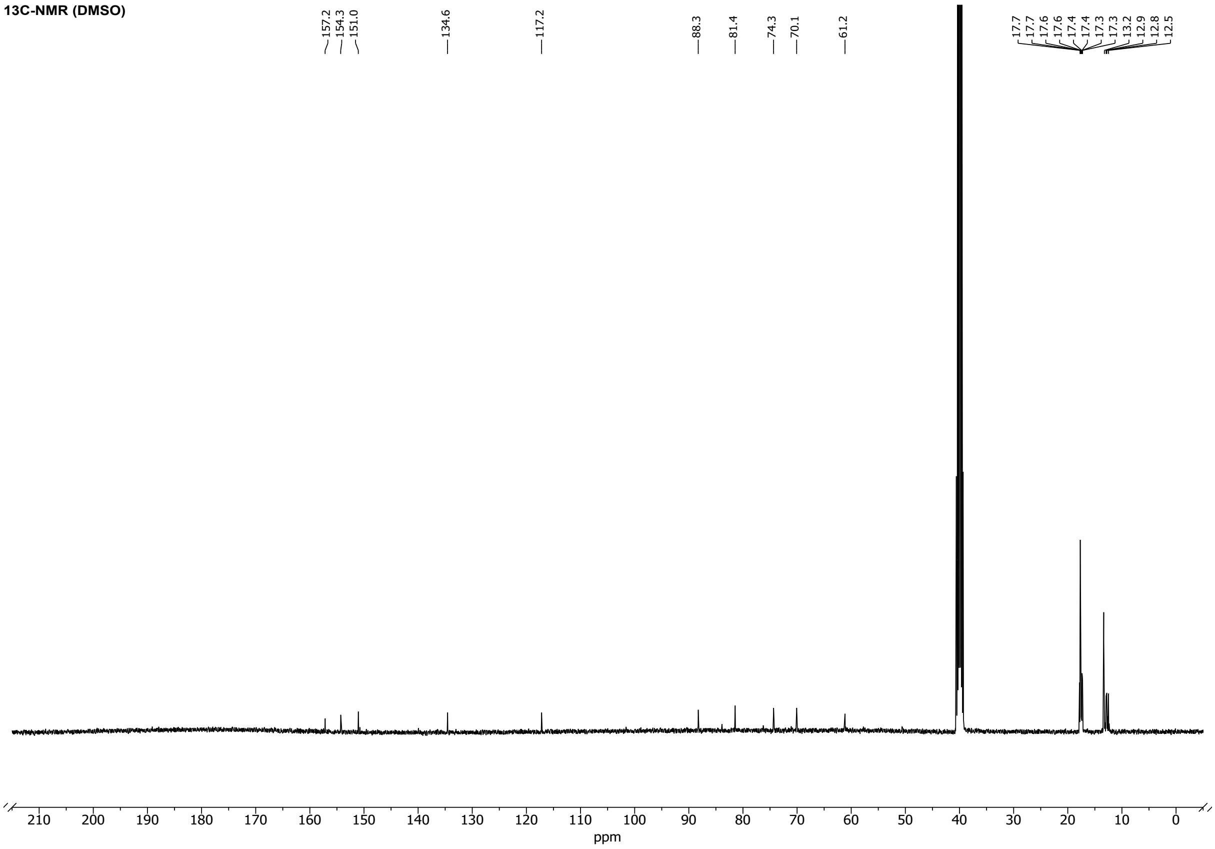

2c

1H-NMR (DMSO)

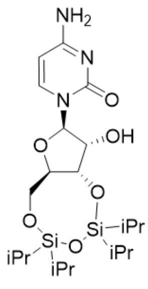

2c

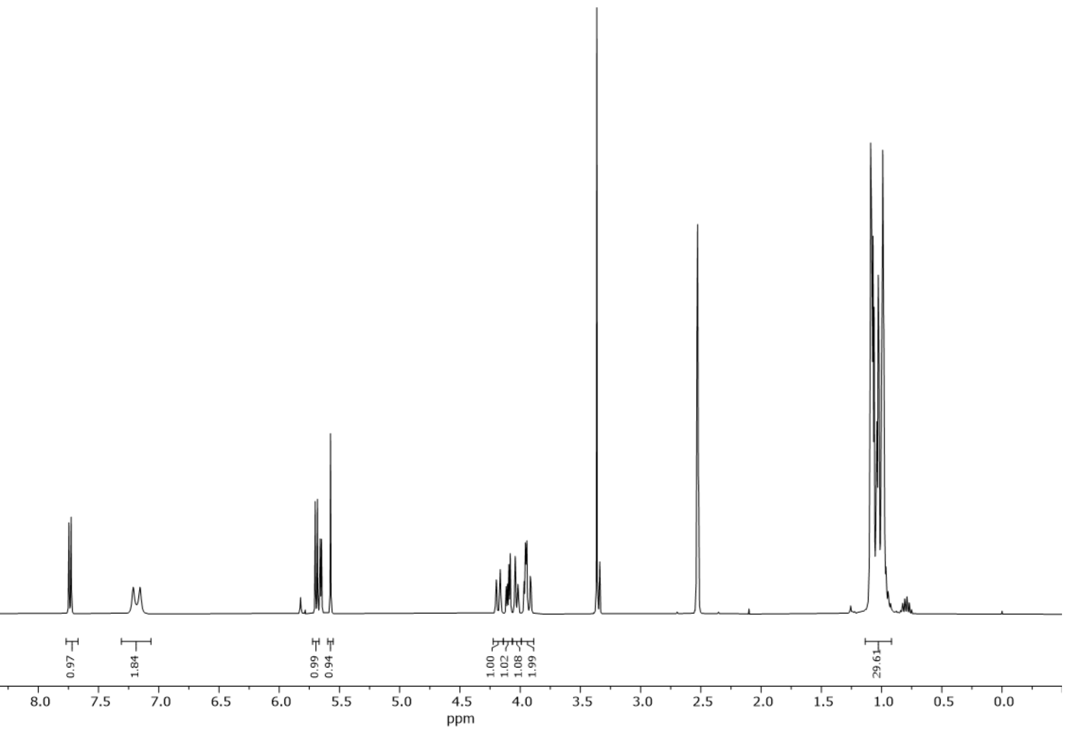

13C-NMR (DMSO)

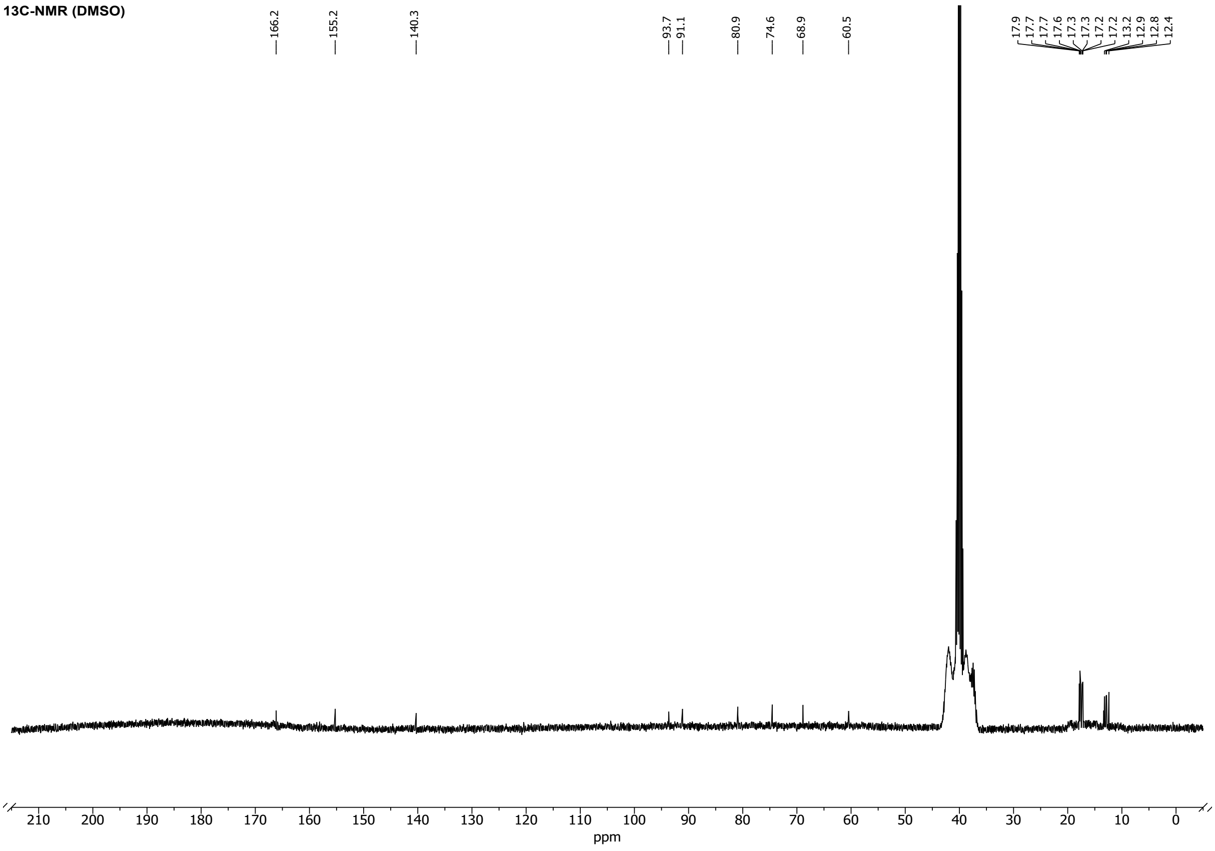

2d

1H-NMR (DMSO)

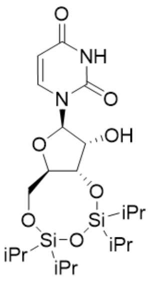

2d

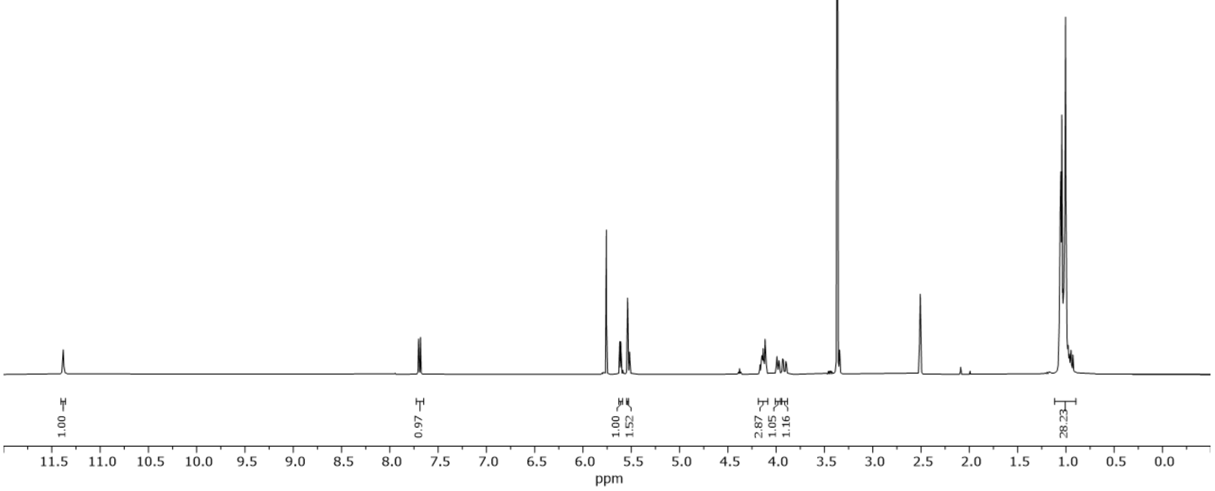

13C-NMR (DMSO)

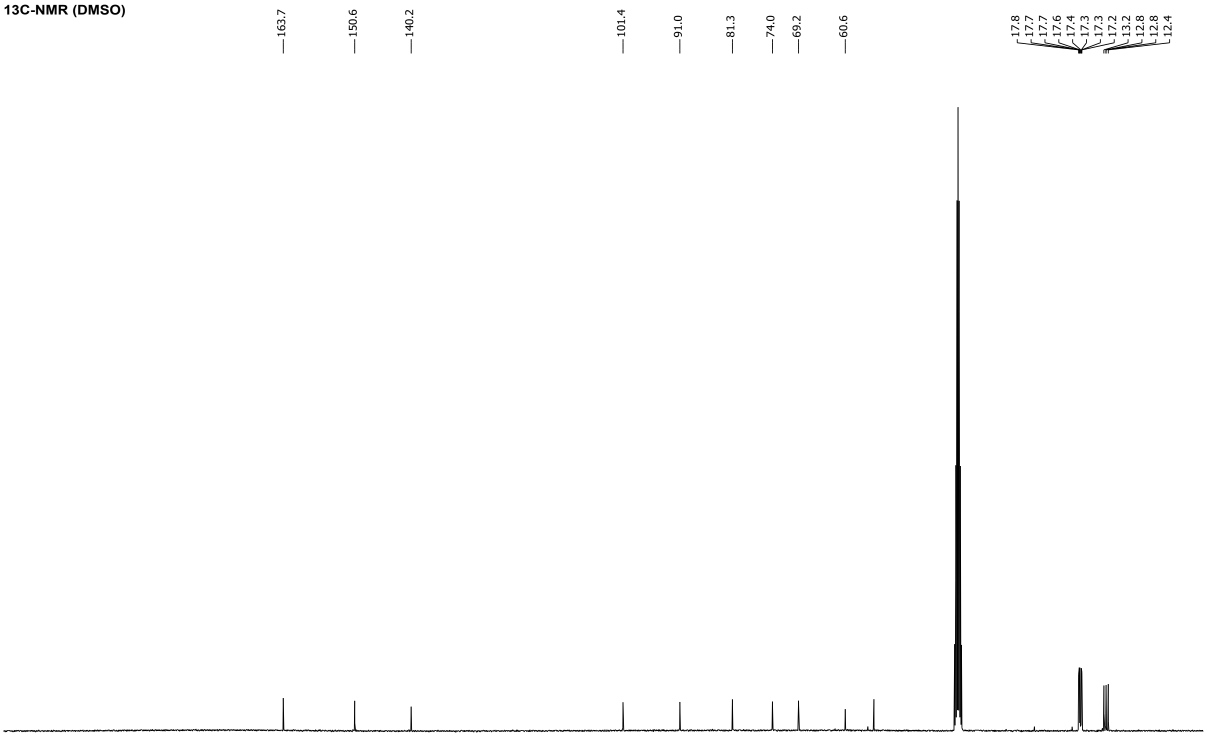

2e

1H-NMR (CDCl3)

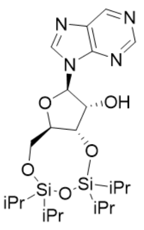

2e

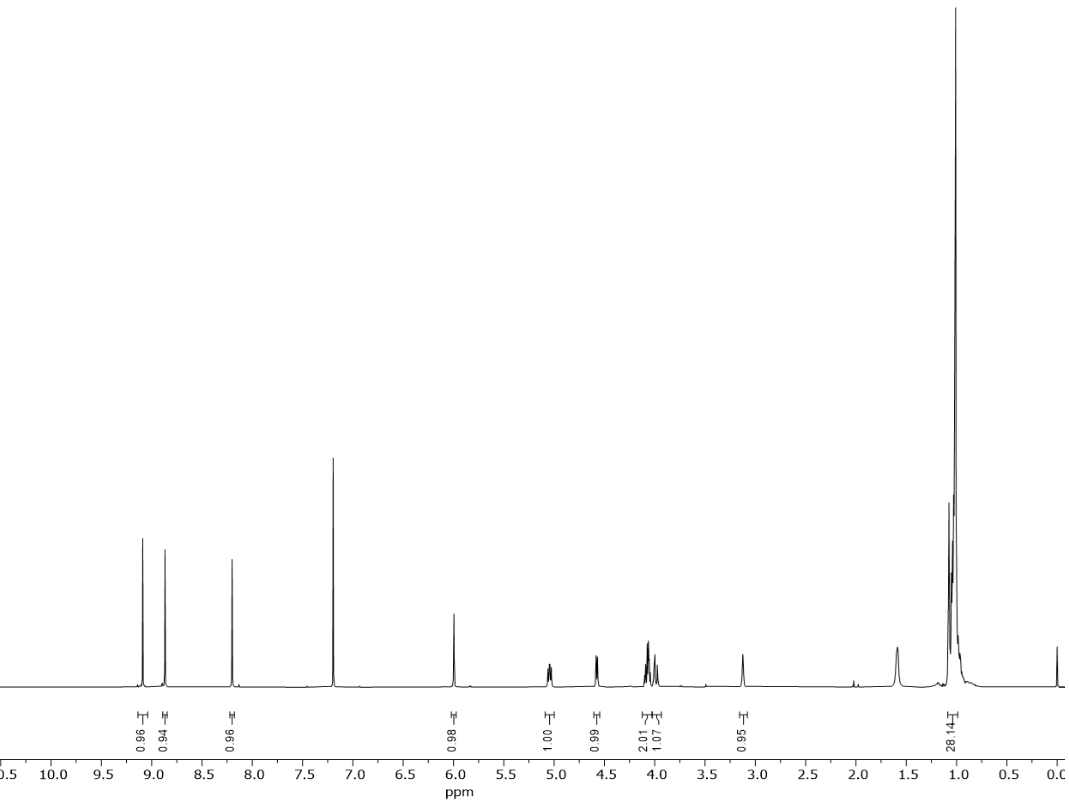

13C-NMR (CDCl3)

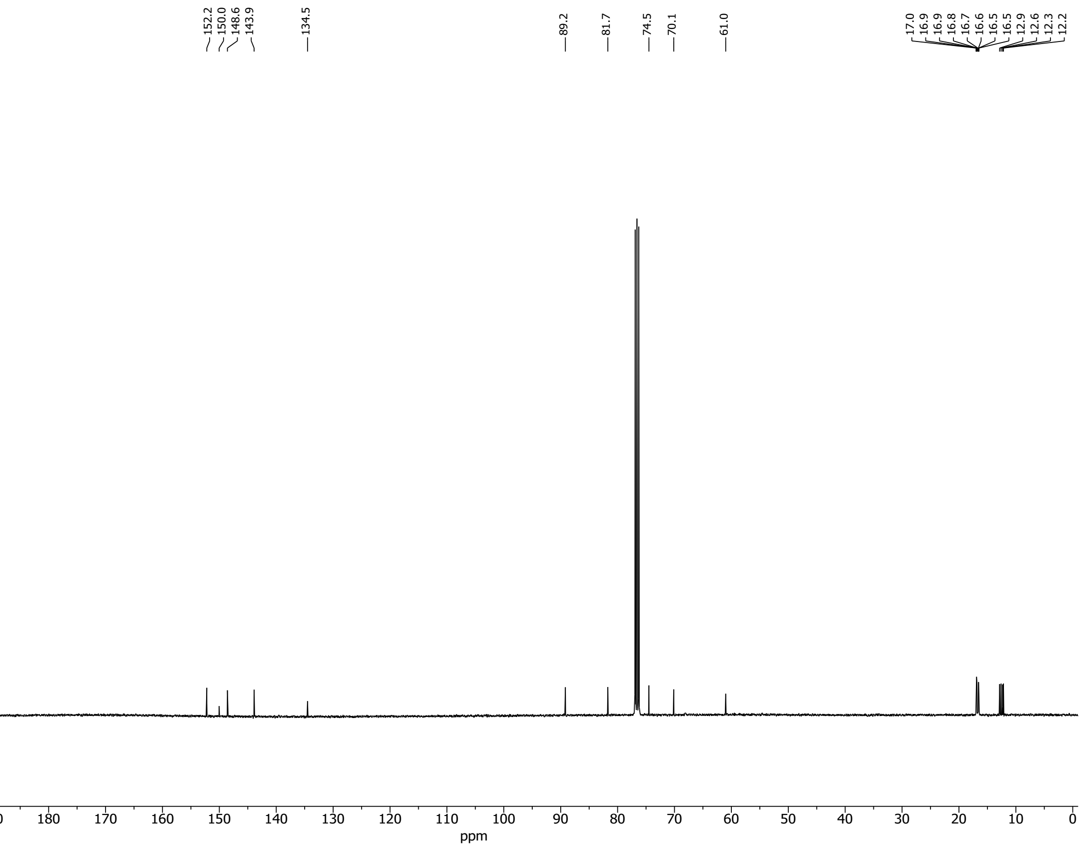

2f

1H-NMR (CDCl3)

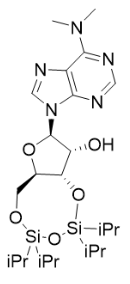

2f

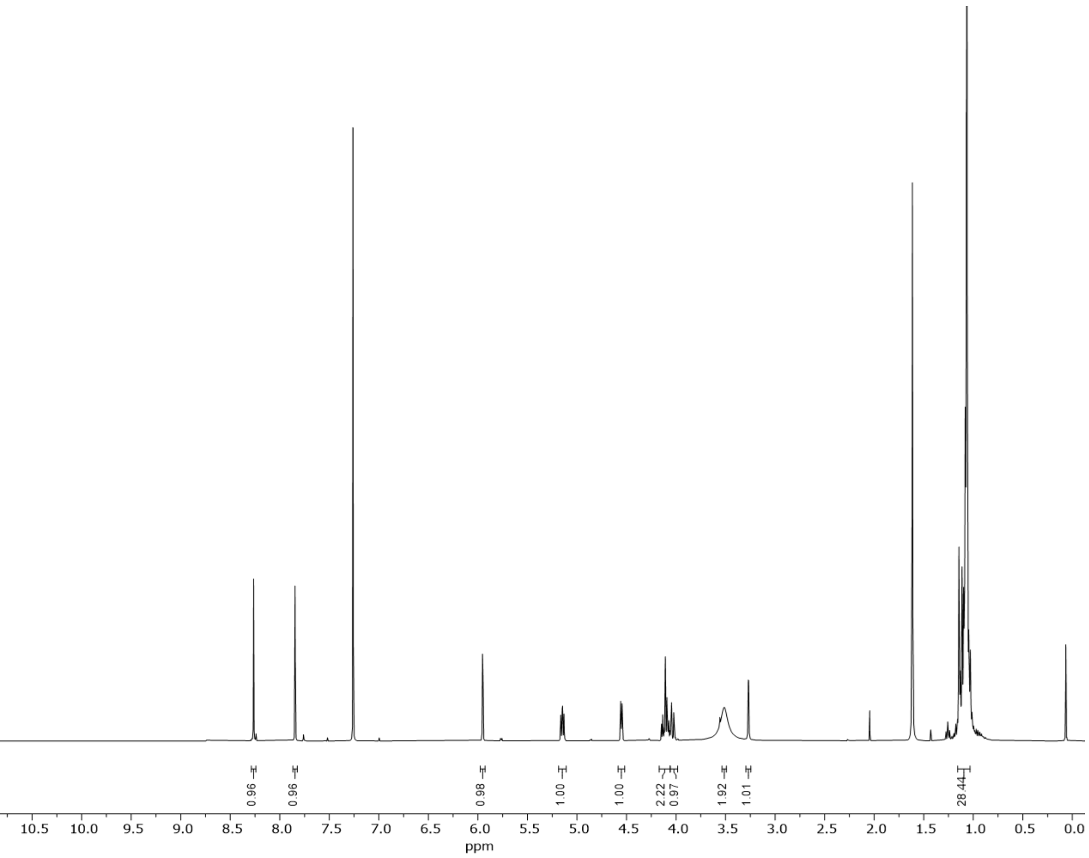

13C-NMR (CDCl3)

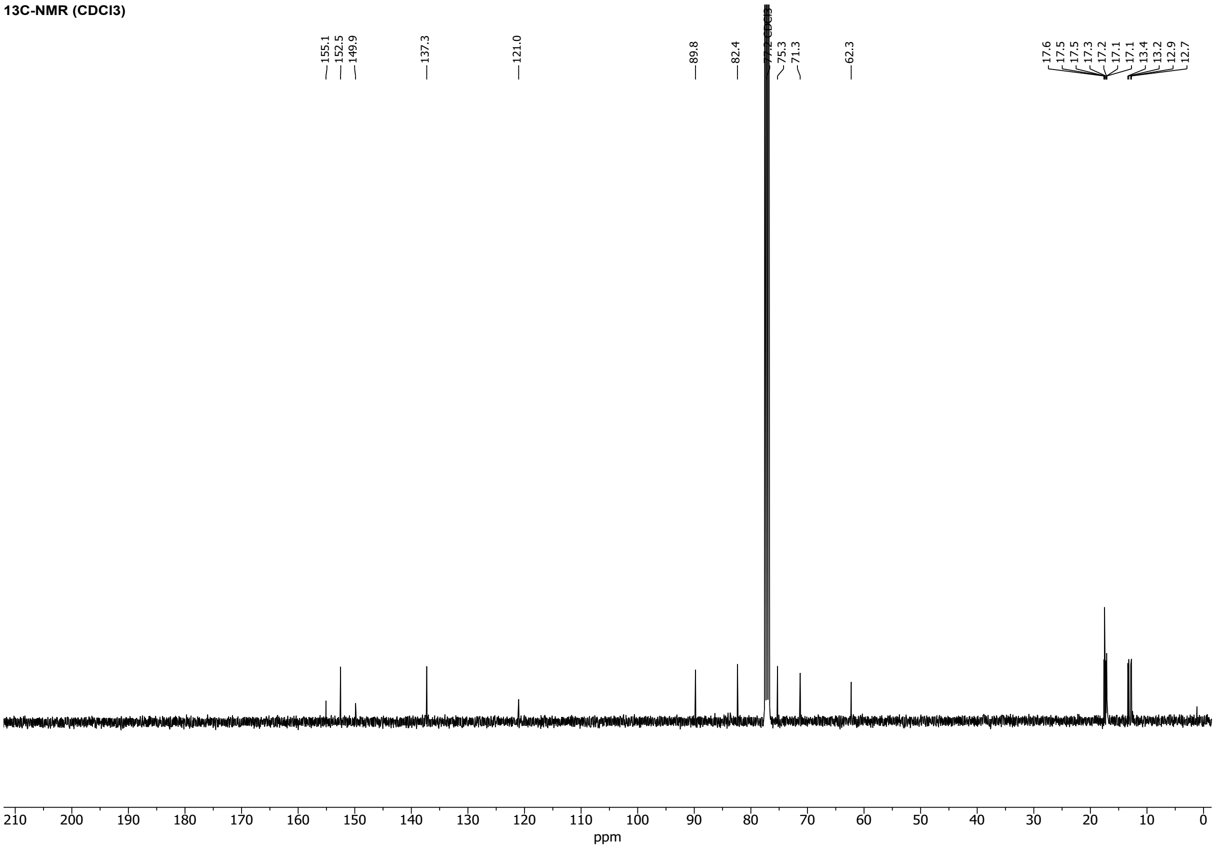

3a

1H-NMR (CDCl3)

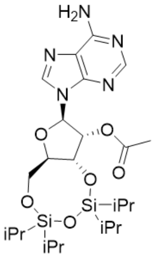

3a

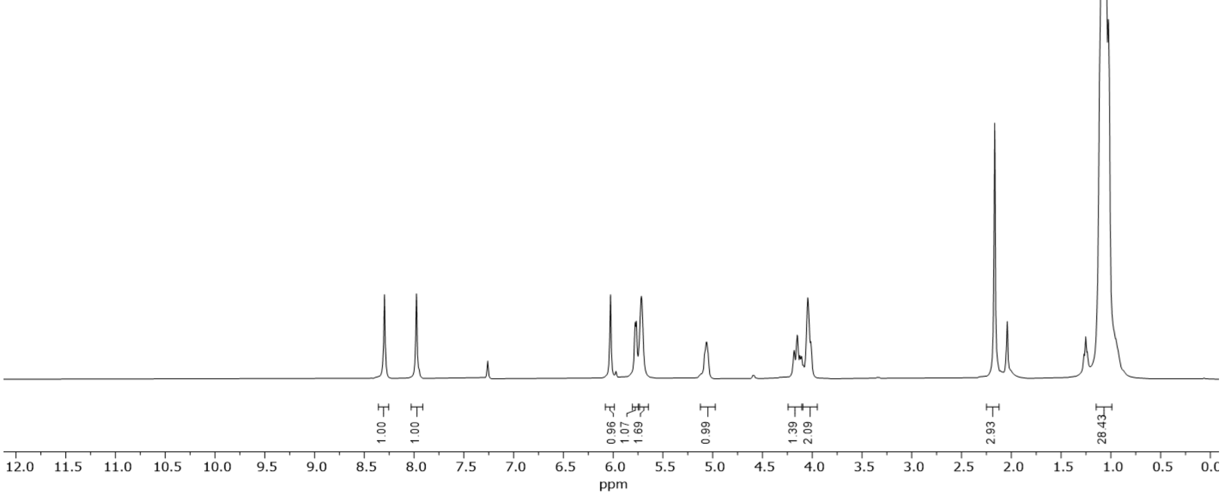

13C-NMR (CDCl3)

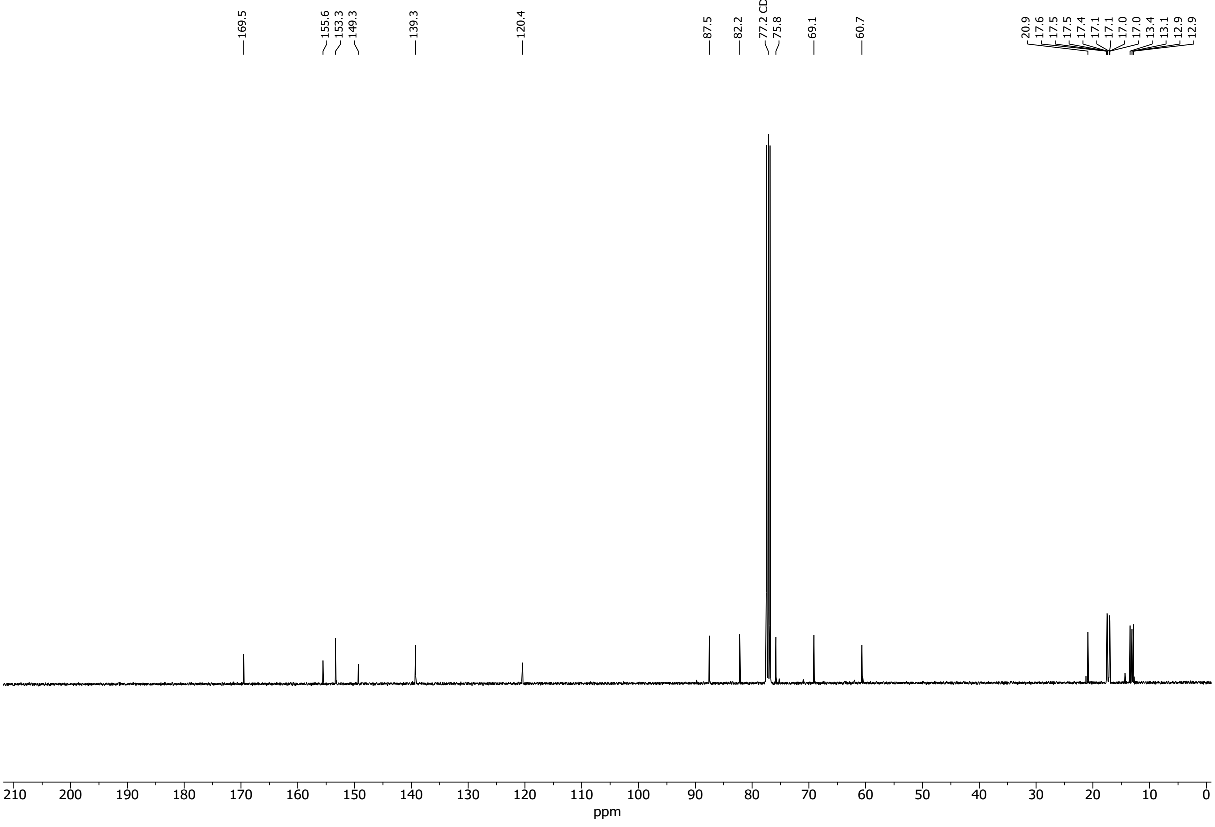

5a

1H-NMR (MeOD)

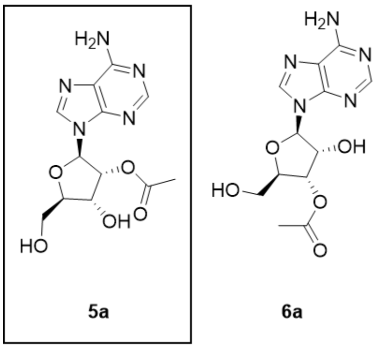

5a **6a**

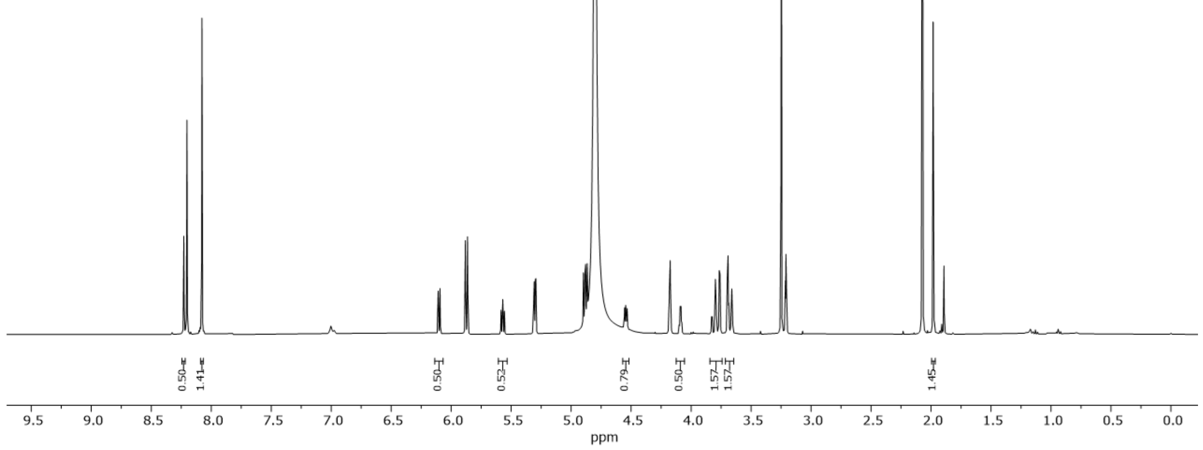

13C-NMR (MeOD)

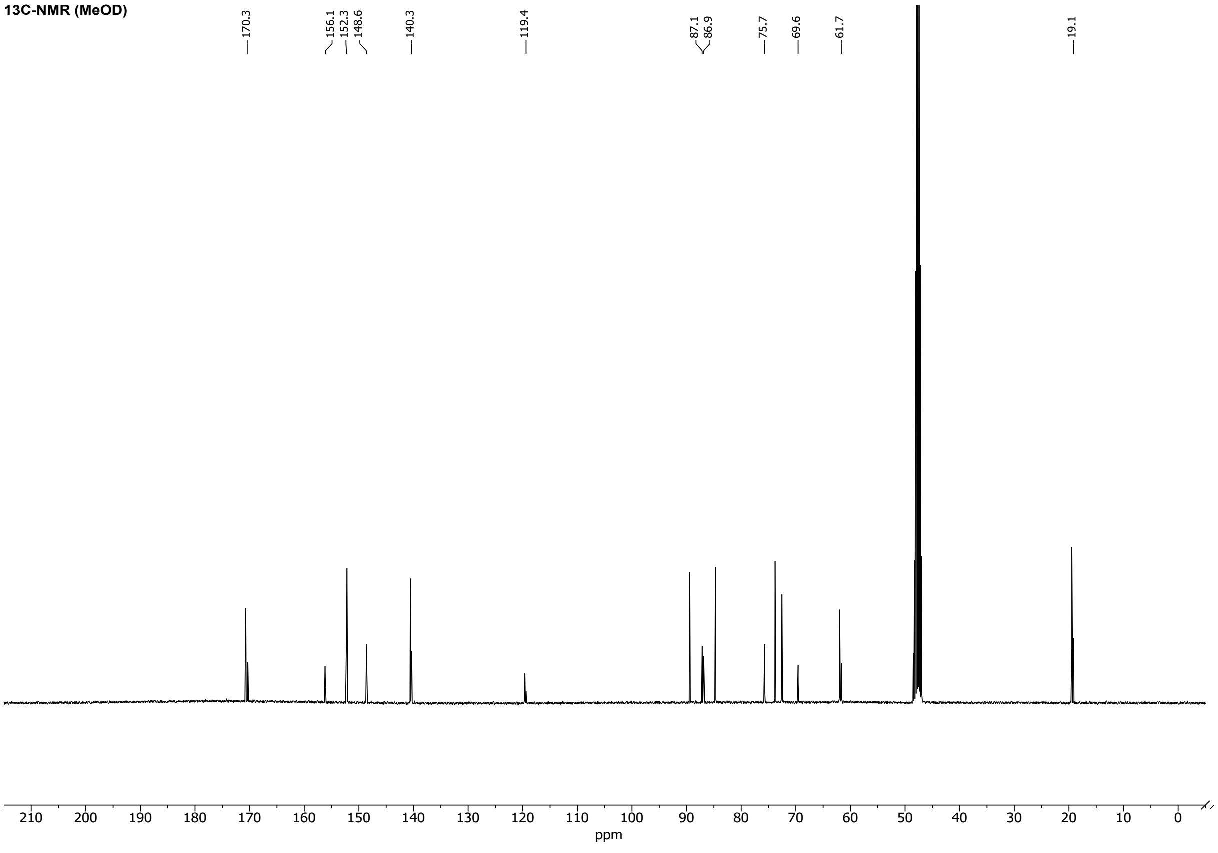

6a

1H-NMR (MeOD)

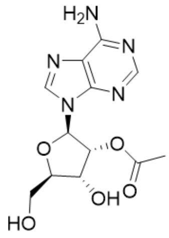

5a

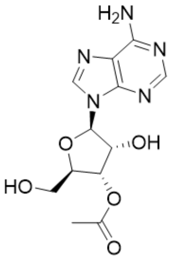

6a

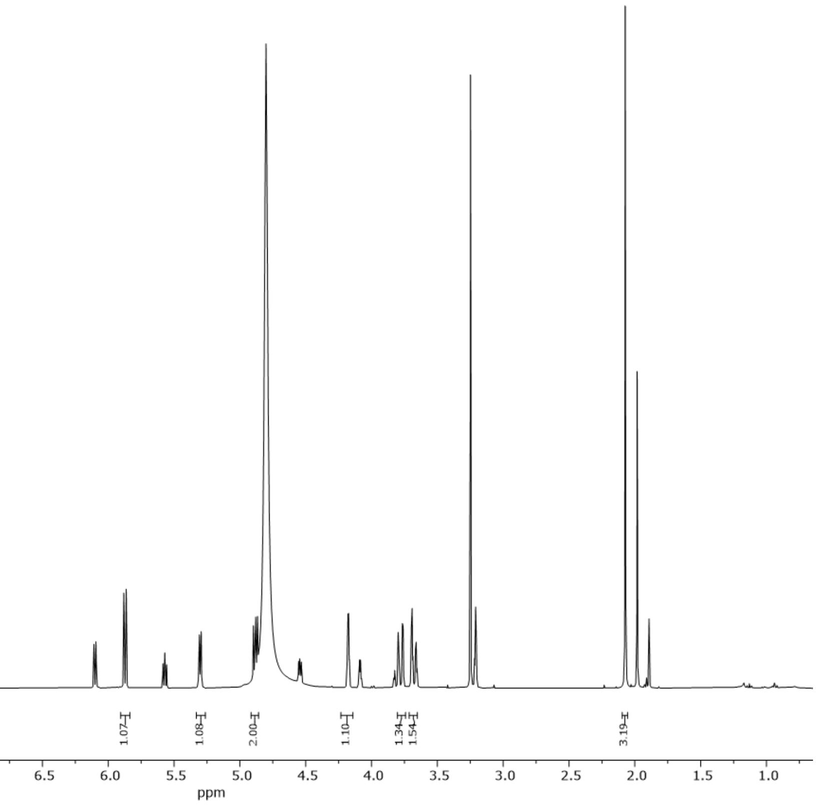

13C-NMR (MeOD)

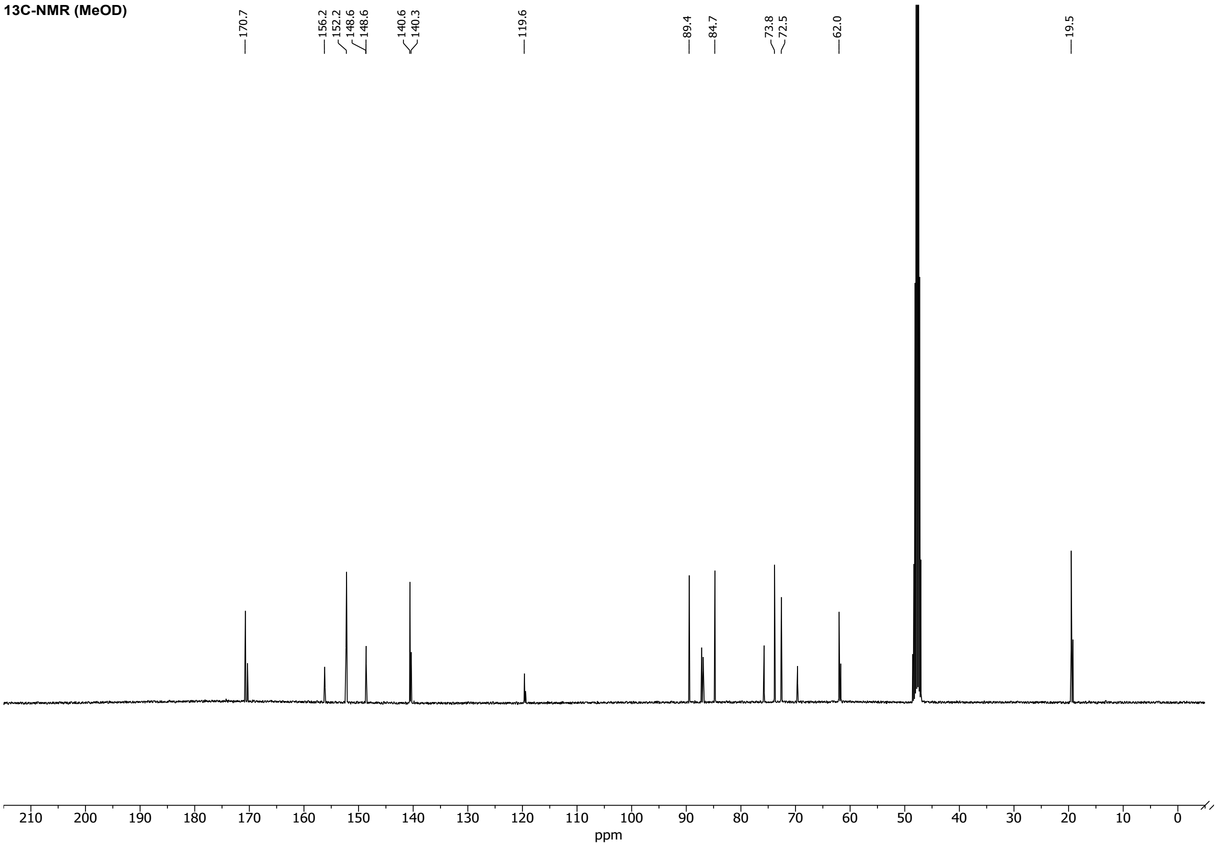

7a

1H-NMR (CDCl3)

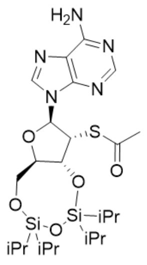

7a

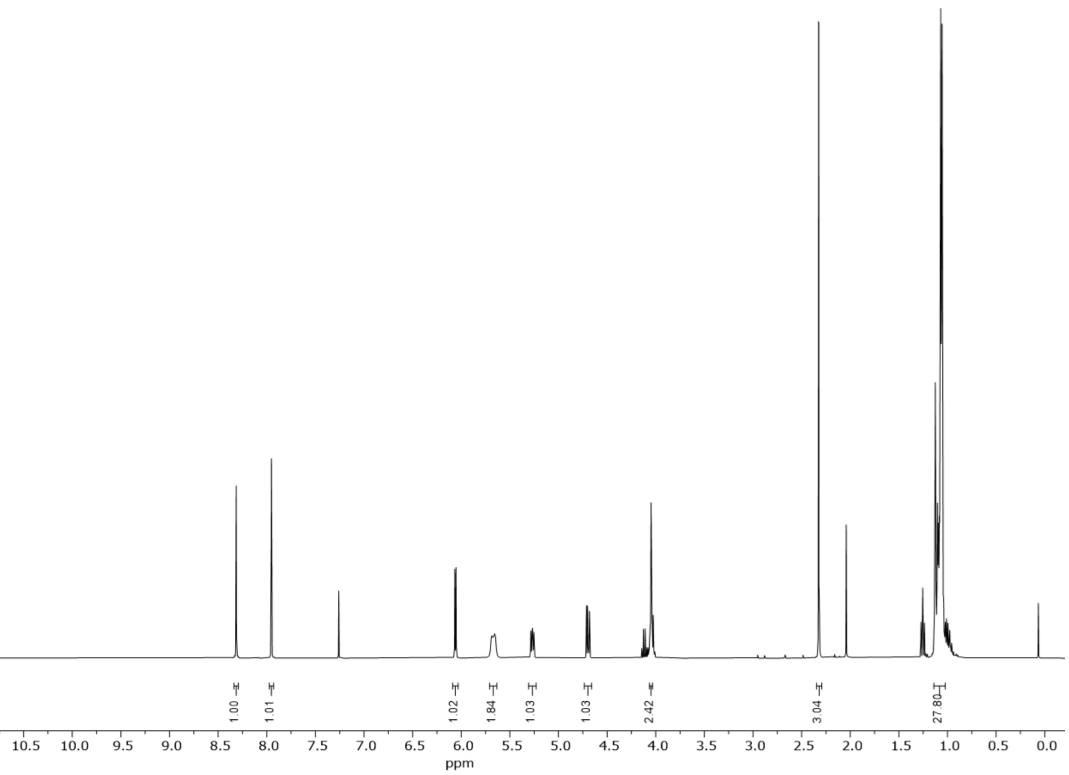

13C-NMR (CDCl3)

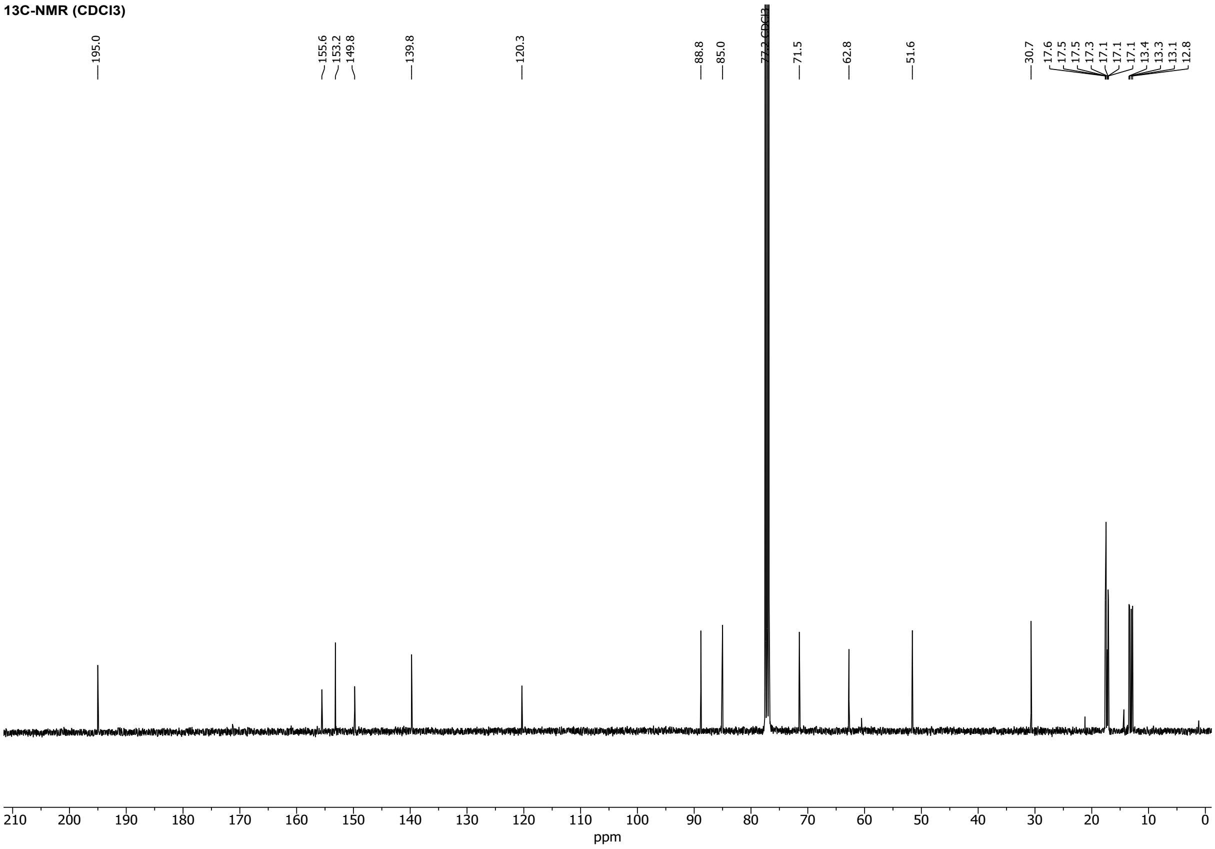

8a

1H-NMR (DMSO)

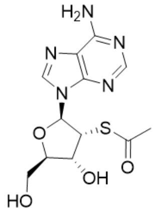

8a

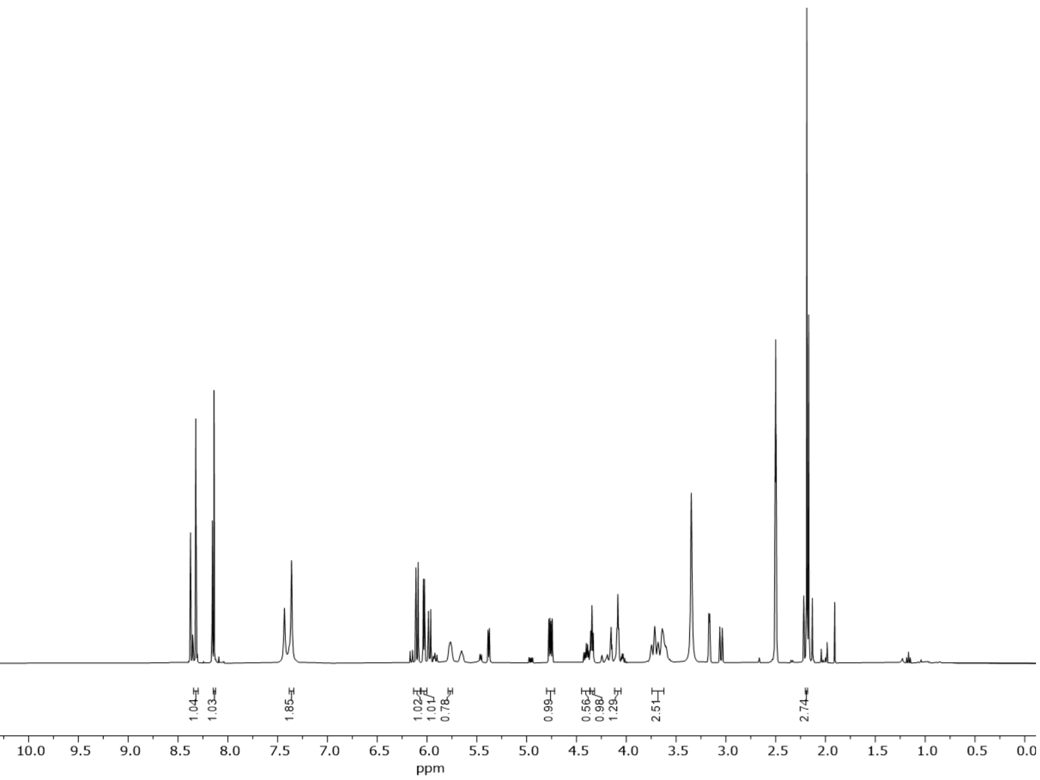

13C-NMR (DMSO)

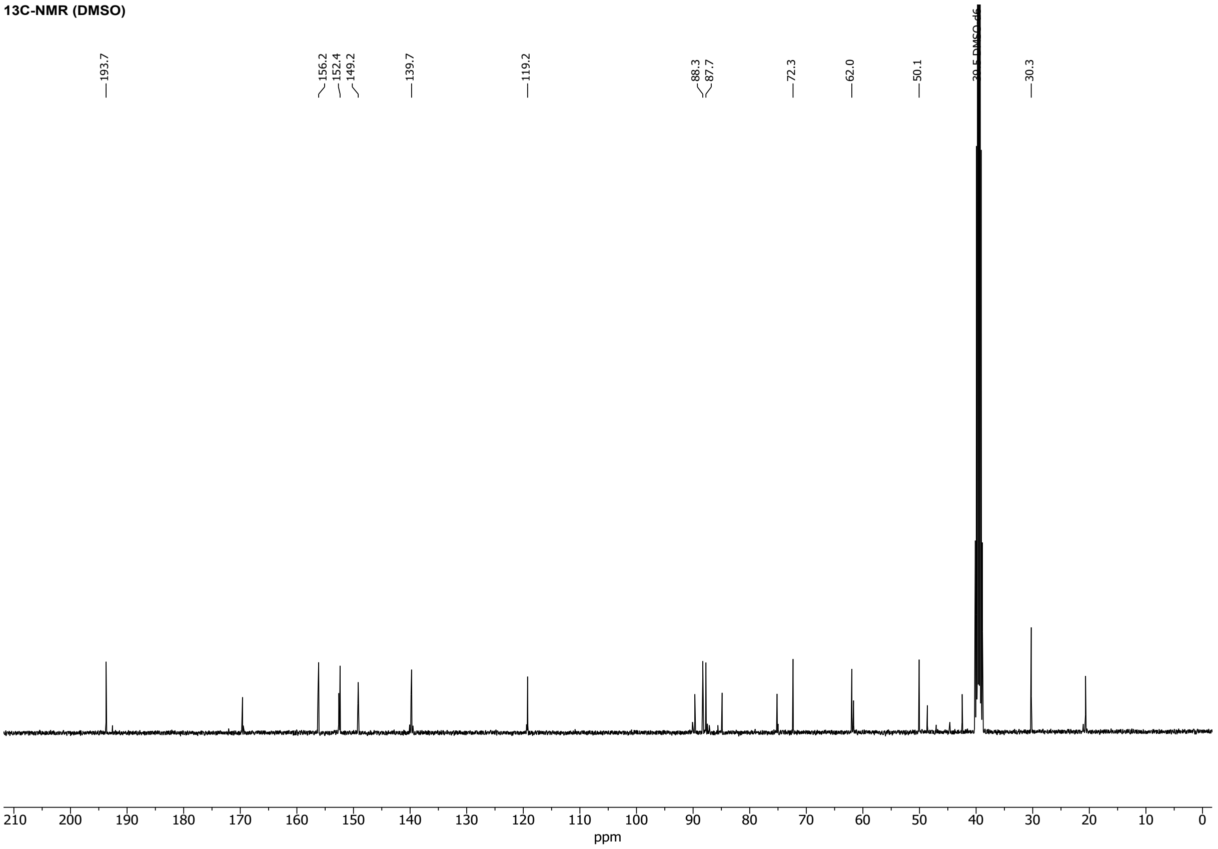

9a

1H-NMR (DMSO)

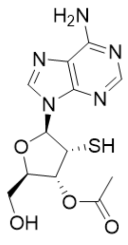

9a

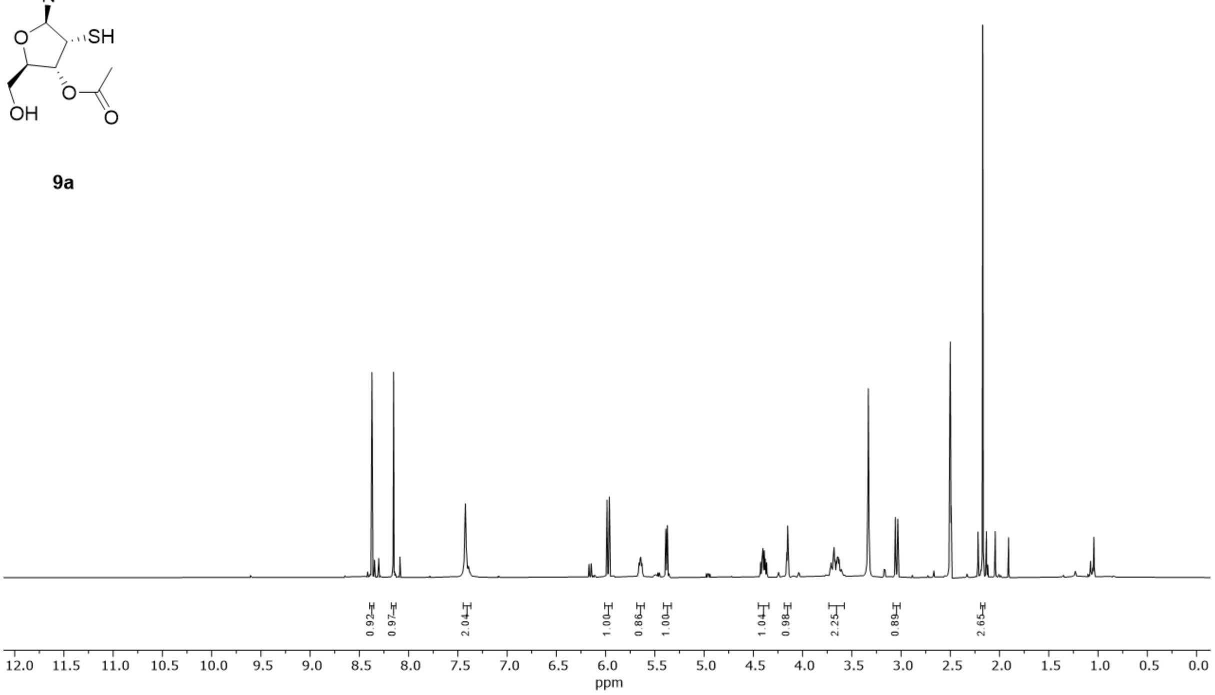

13C-NMR (DMSO)

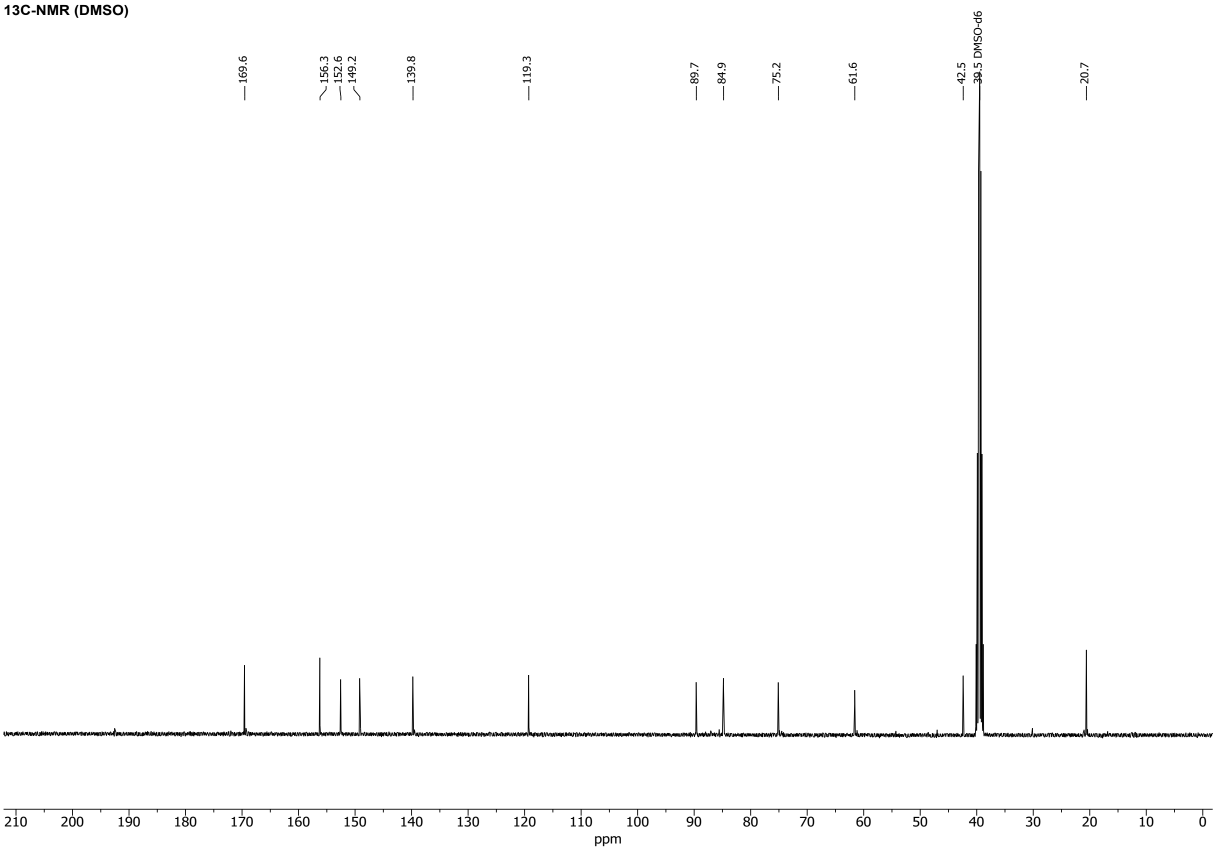

10a

1H-NMR (DMSO)

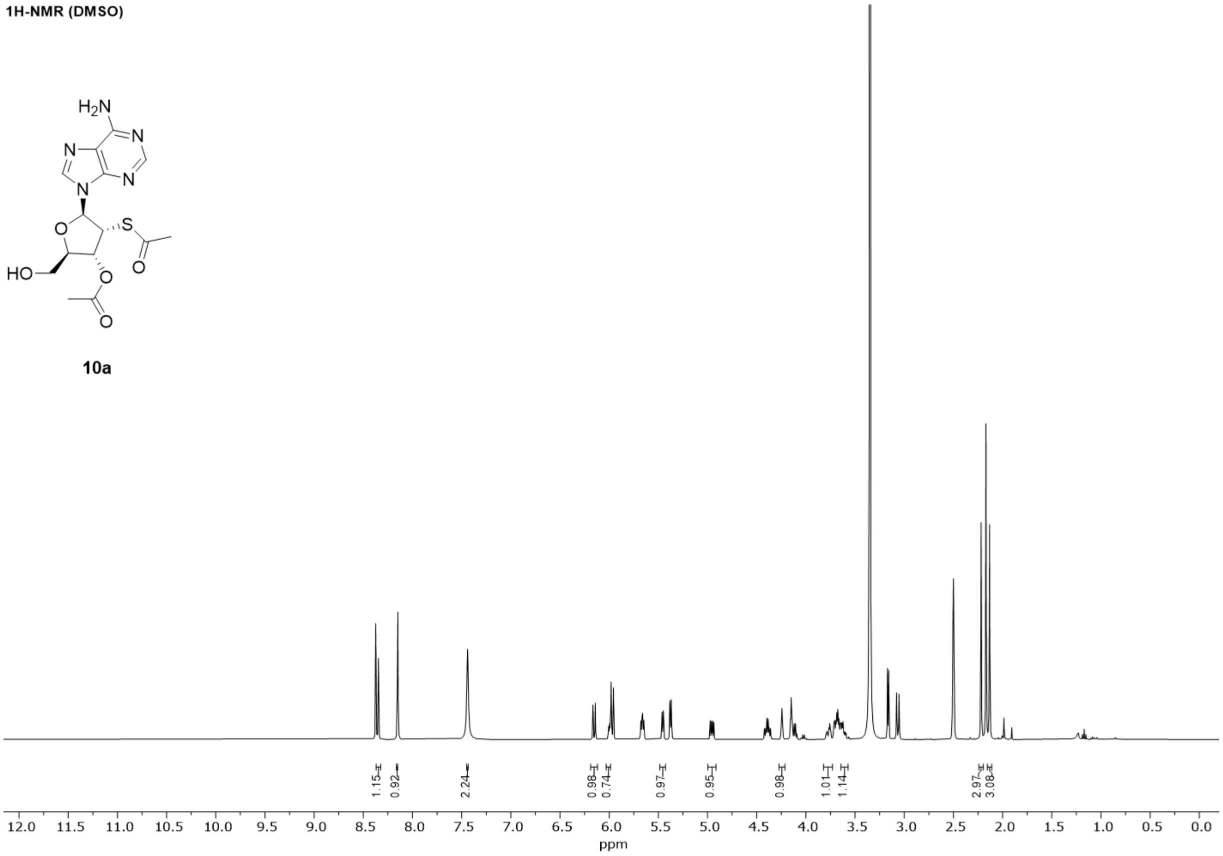

13C-NMR (DMSO)

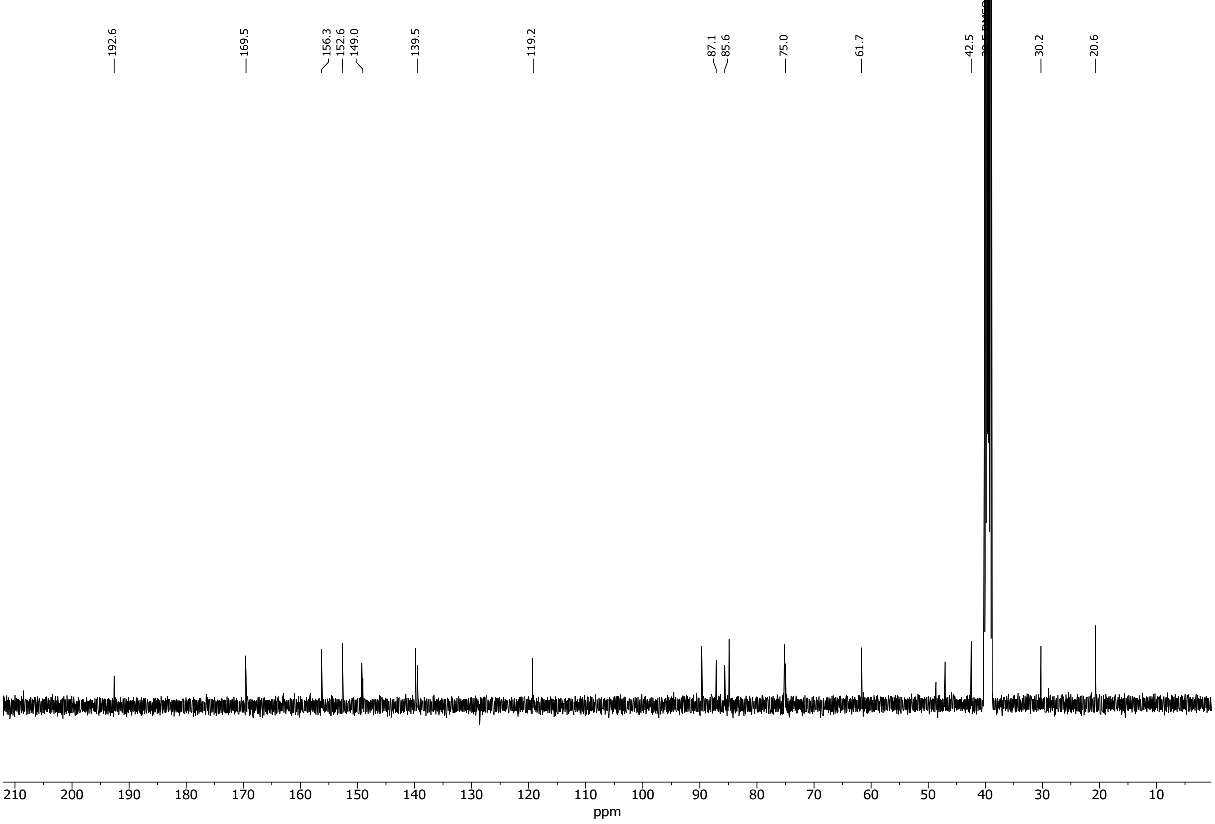

11a

1H-NMR (DMSO)

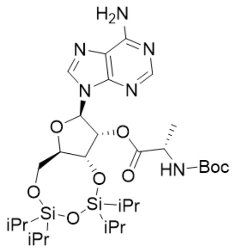

11a

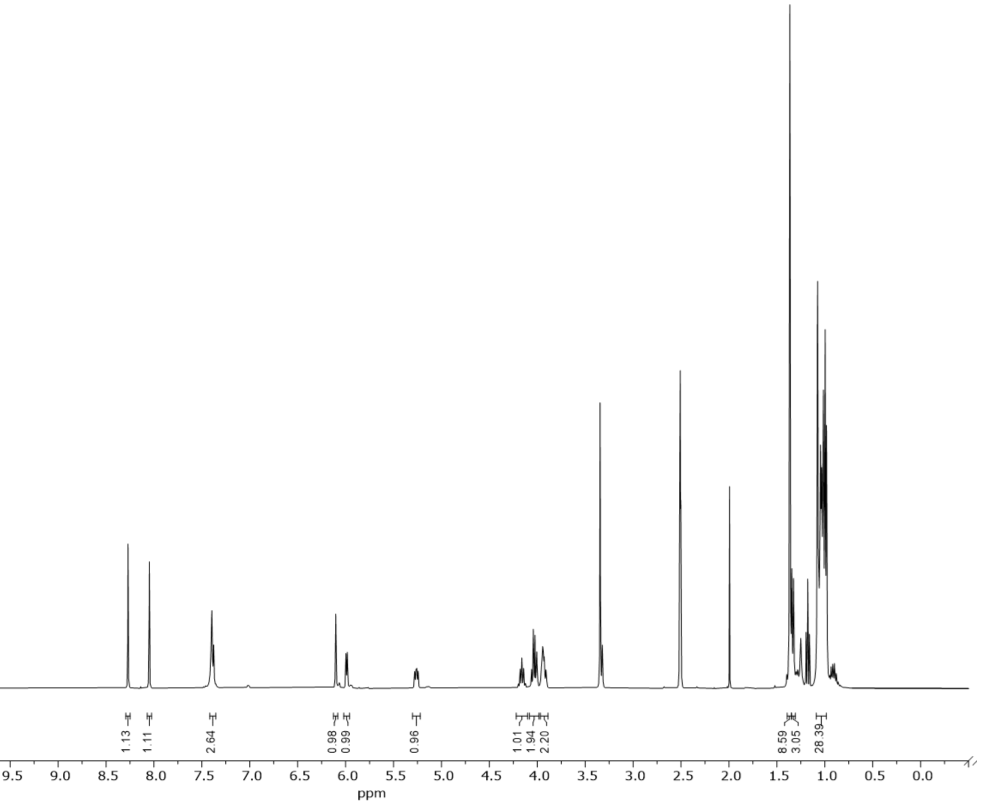

13C-NMR (DMSO)

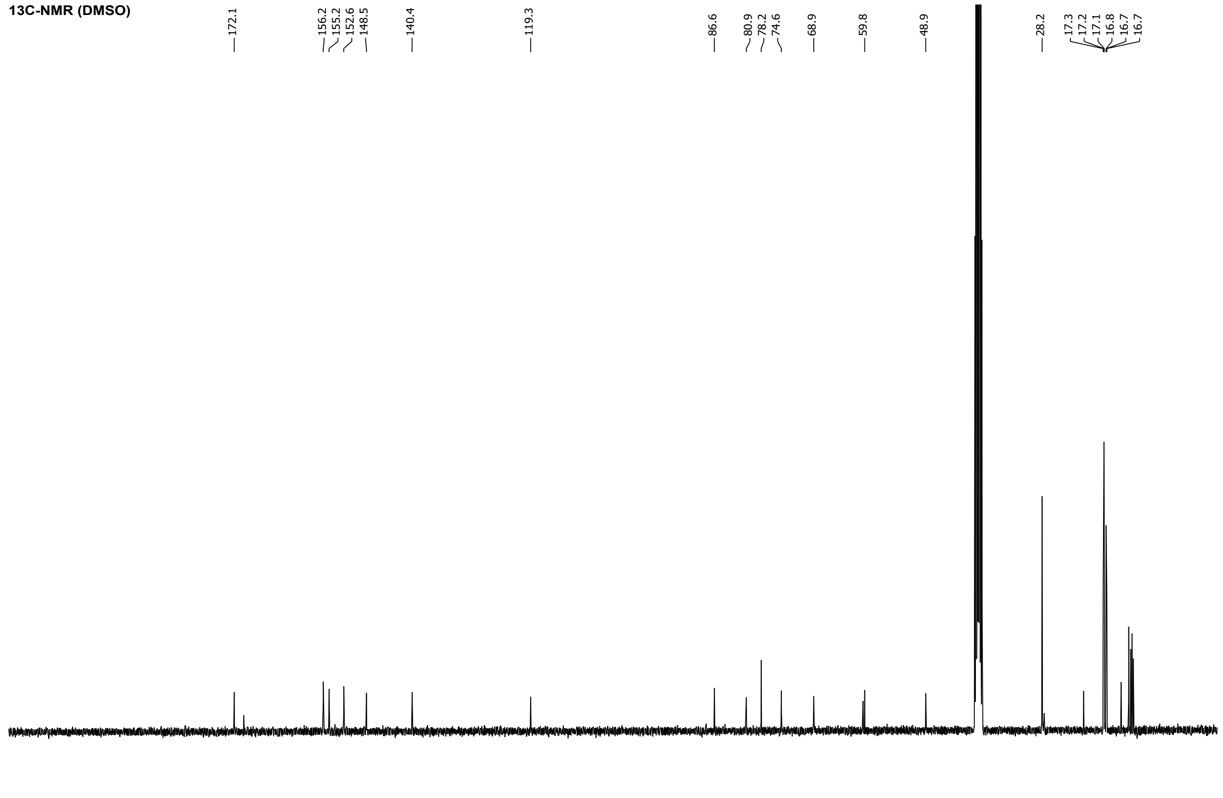

11b

1H-NMR (DMSO)

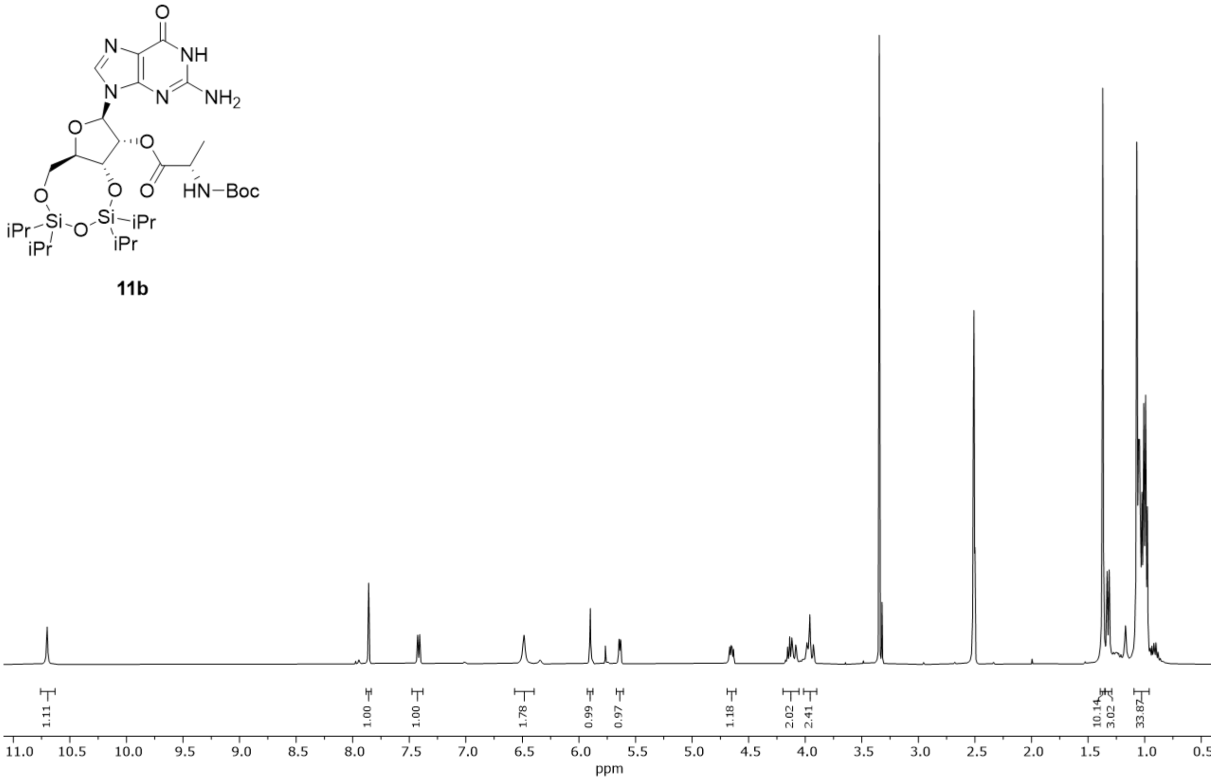

11b

13C-NMR (DMSO)

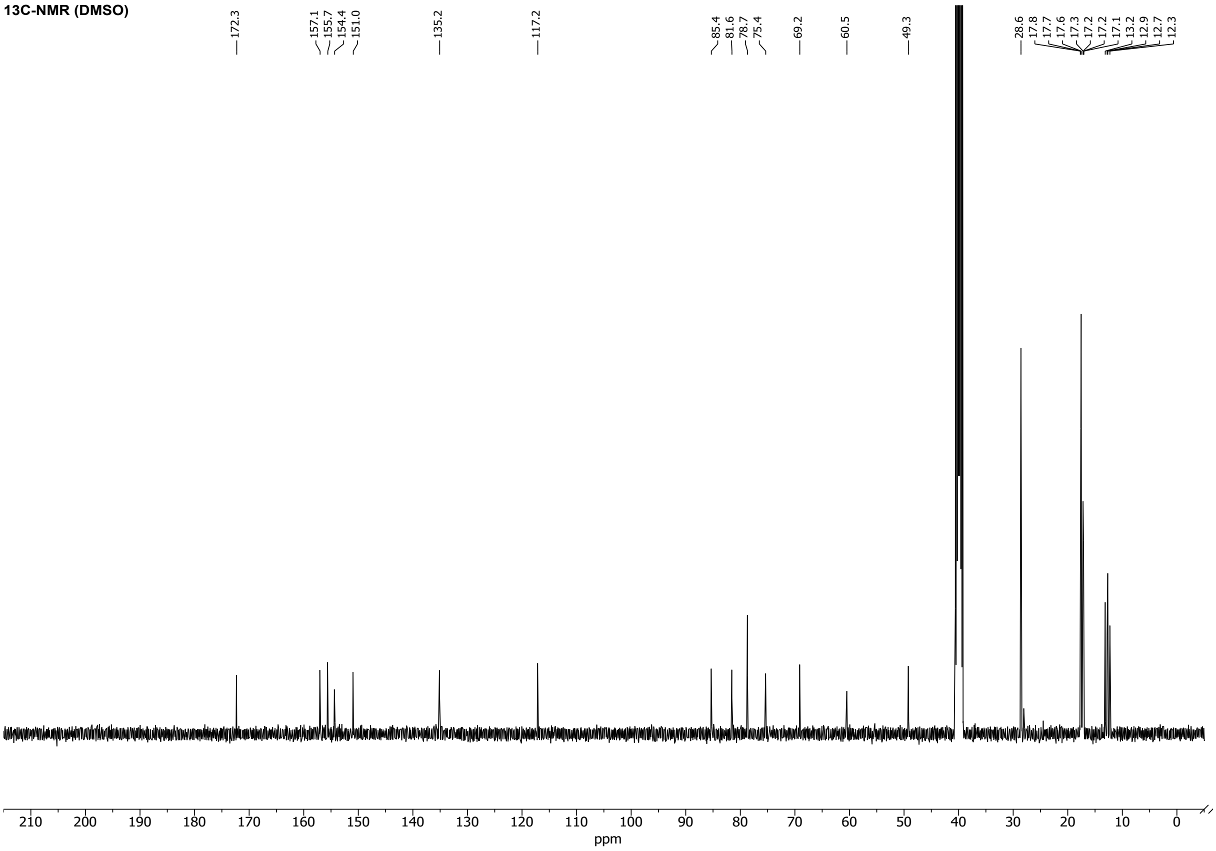

11c

1H-NMR (DMSO)

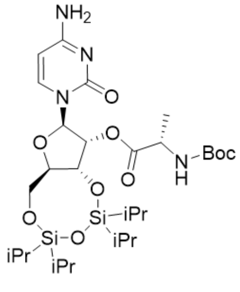

11c

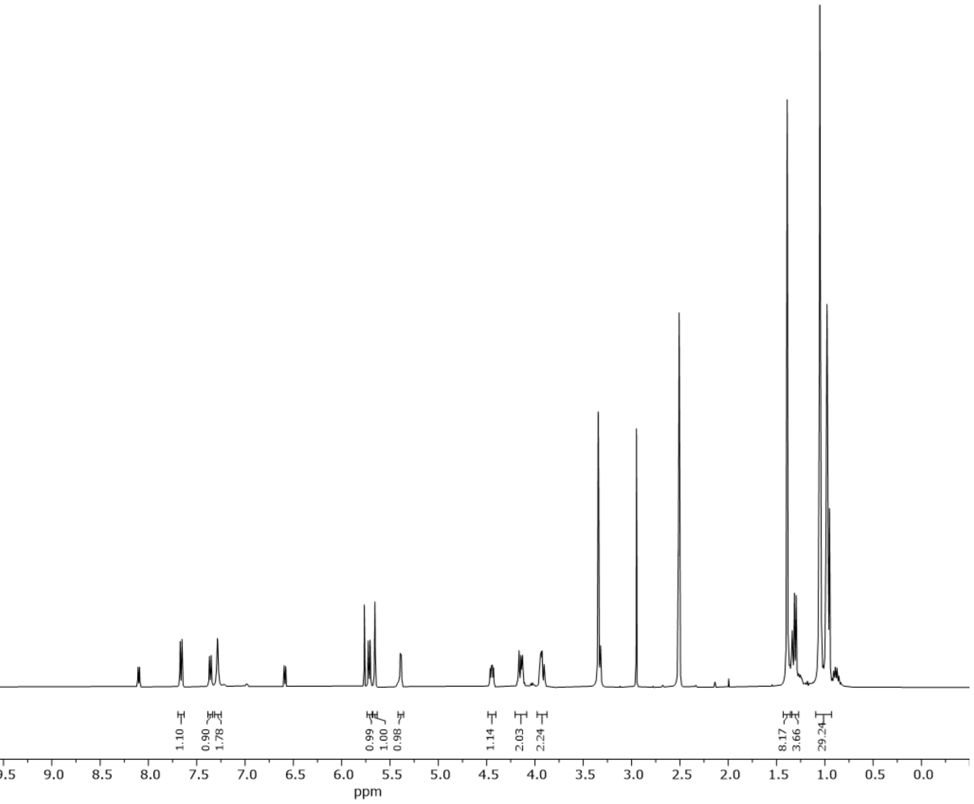

13C-NMR (DMSO)

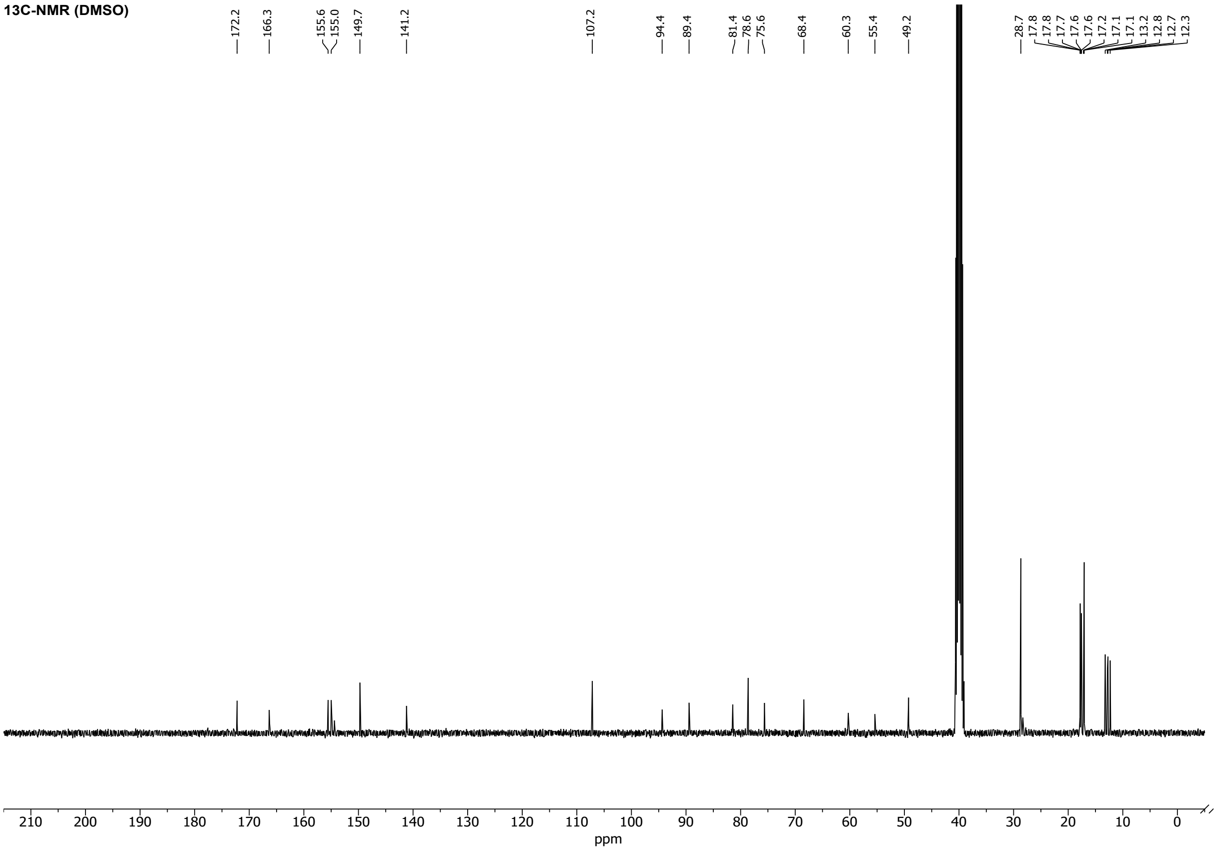

11d

1H-NMR (DMSO)

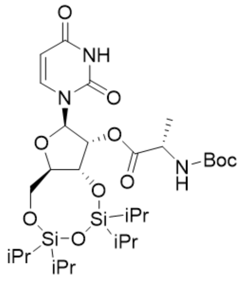

11d

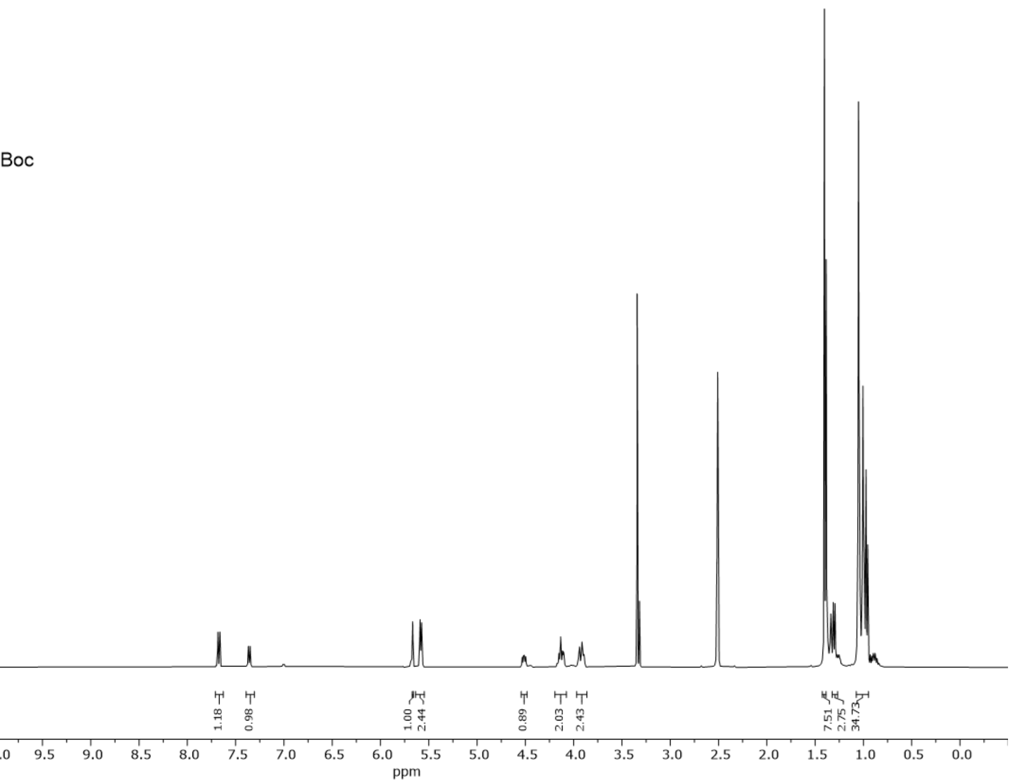

13C-NMR (DMSO)

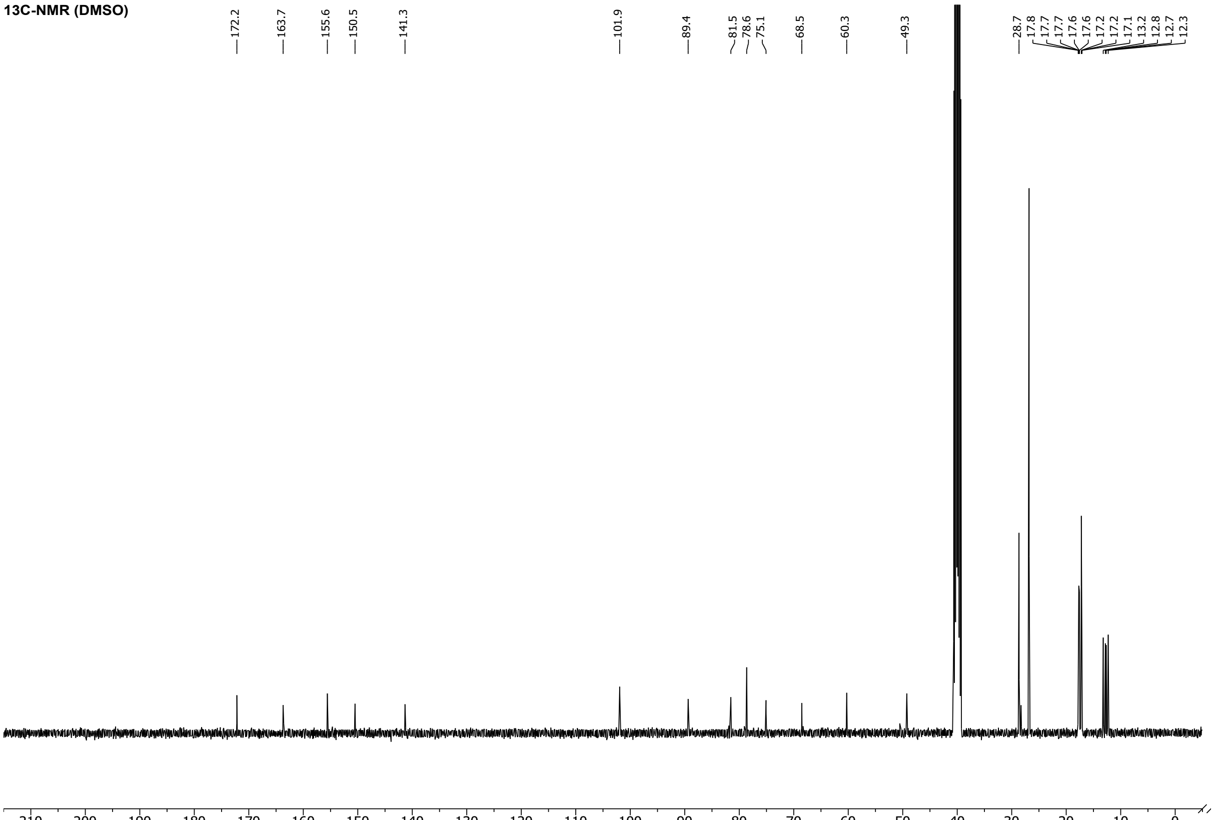

11e

1H-NMR (CDCl3)

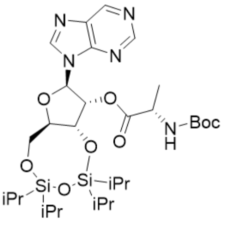

11e

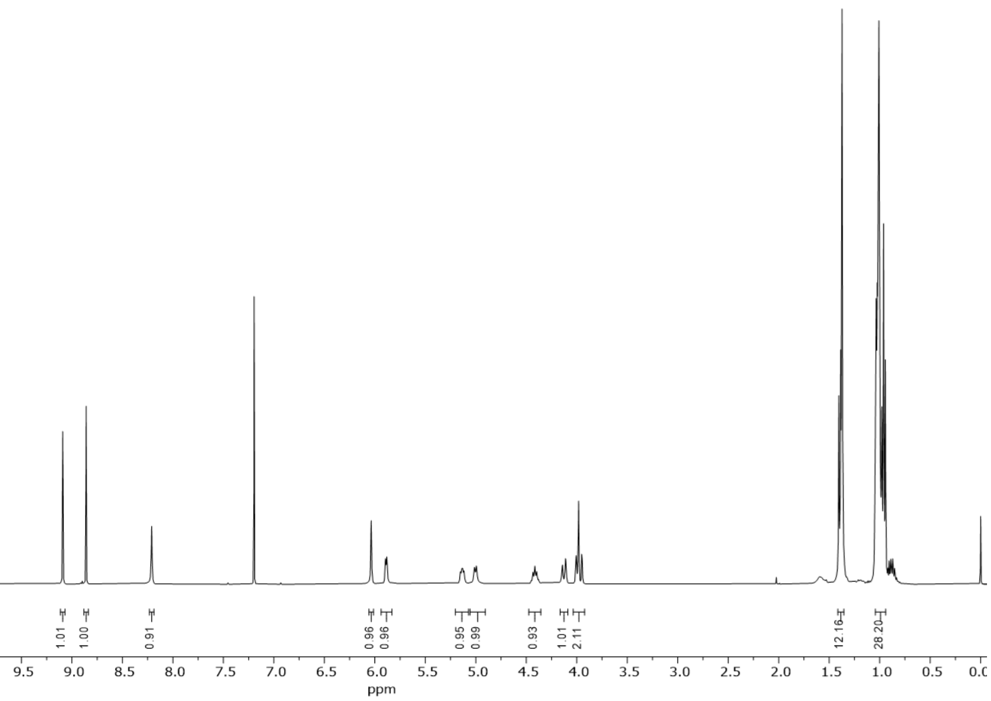

13C-NMR (CDCl3)

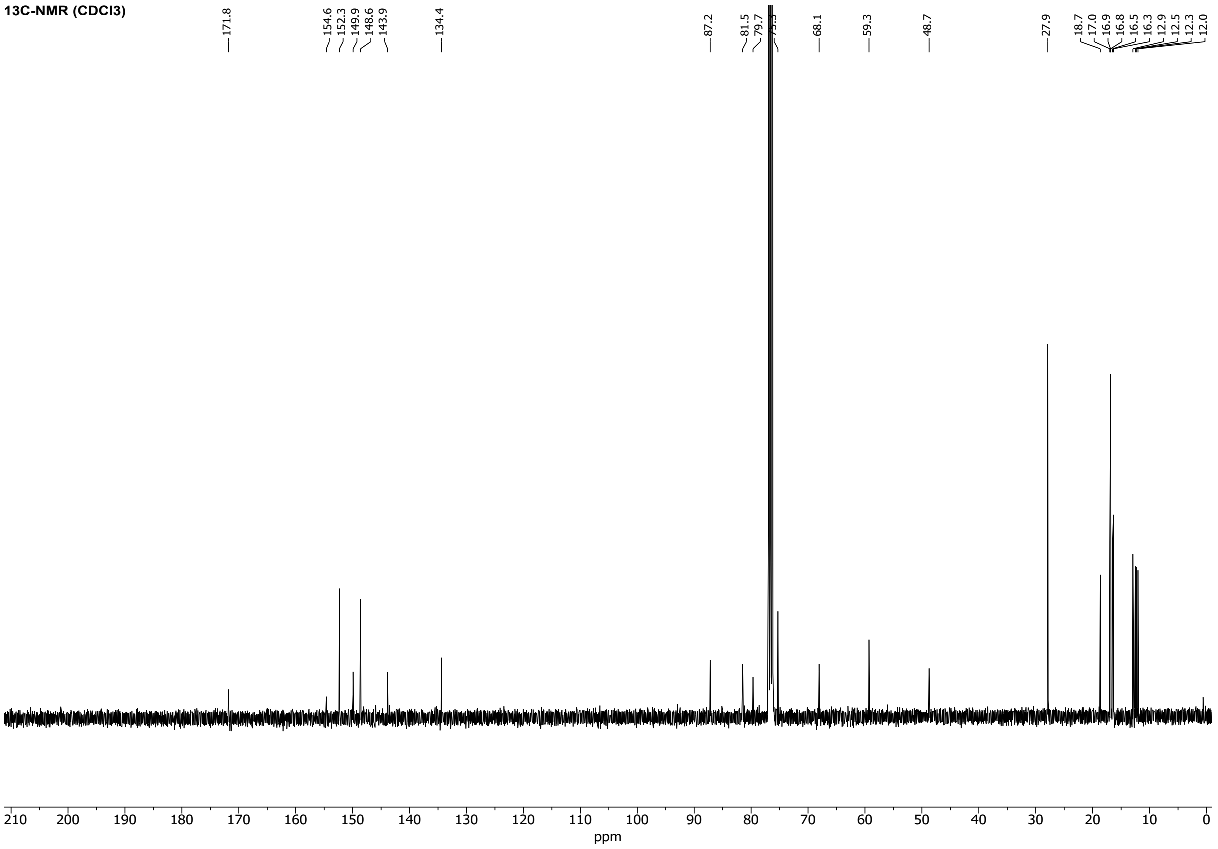

11f

1H-NMR (CDCl3)

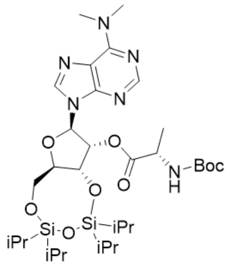

11f

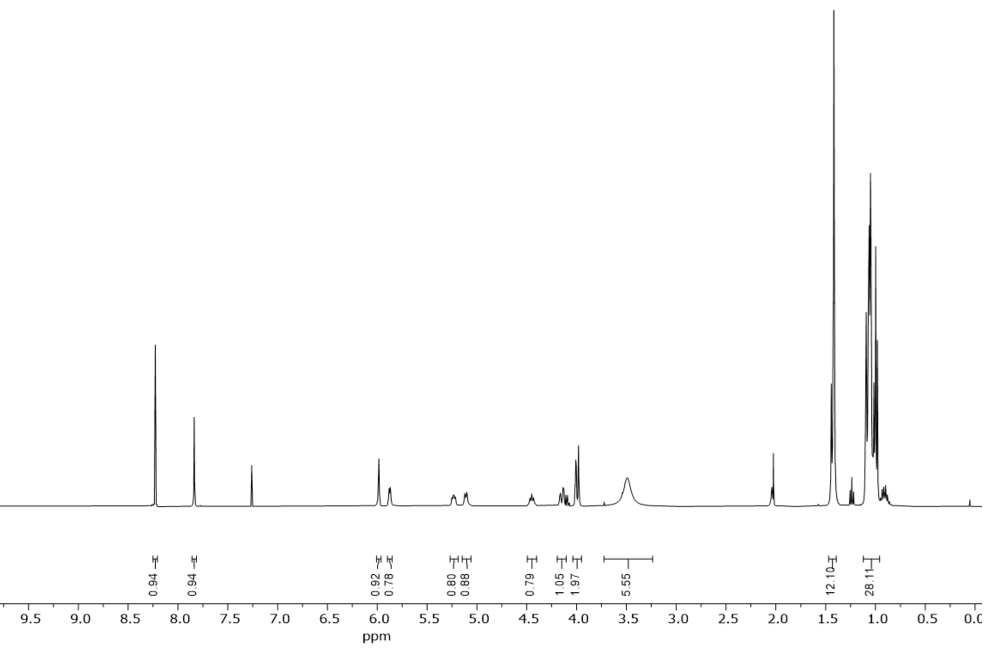

13C-NMR (CDCl3)

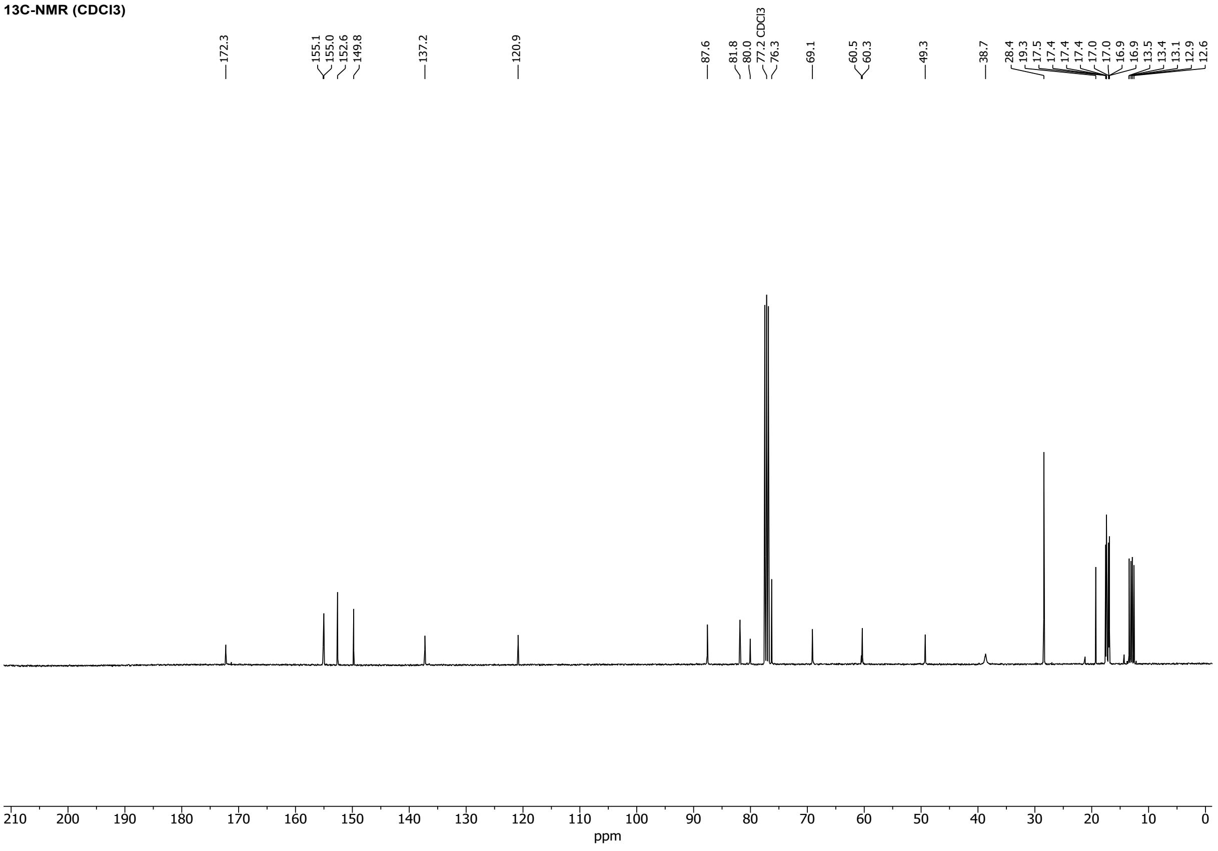

11g

1H-NMR (CDCl3)

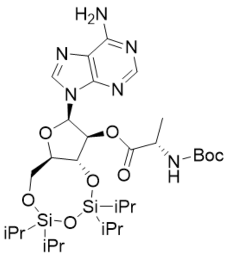

11g

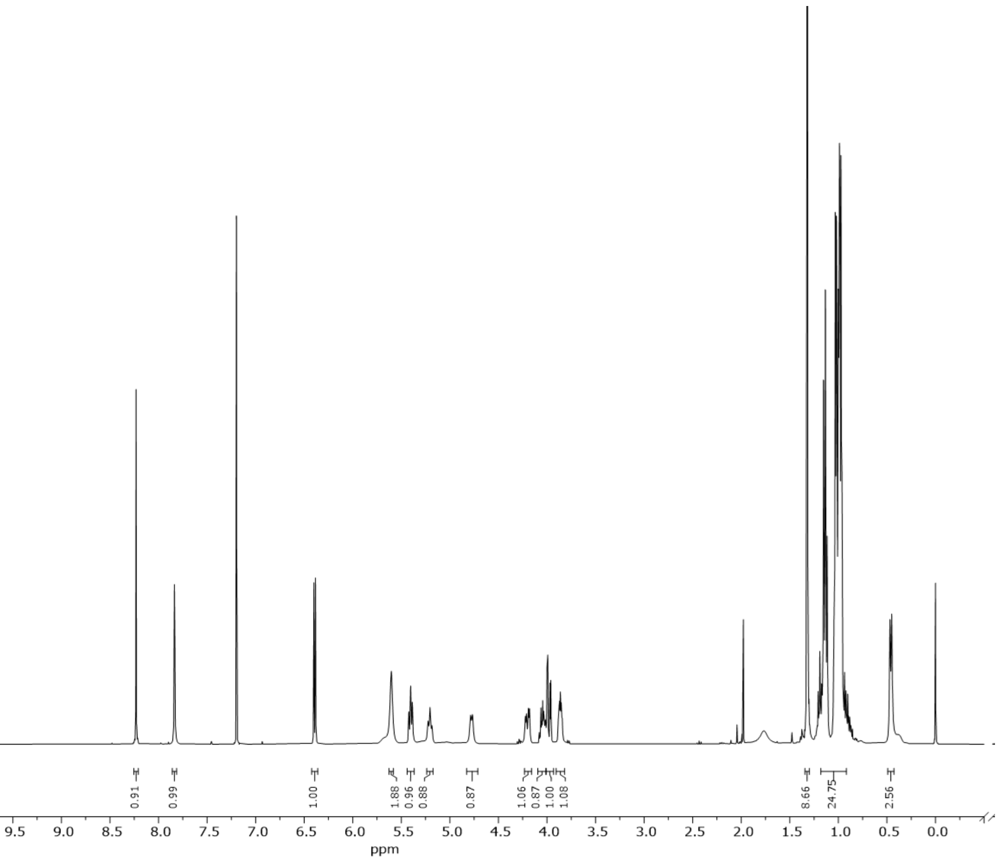

13C-NMR (CDCl3)

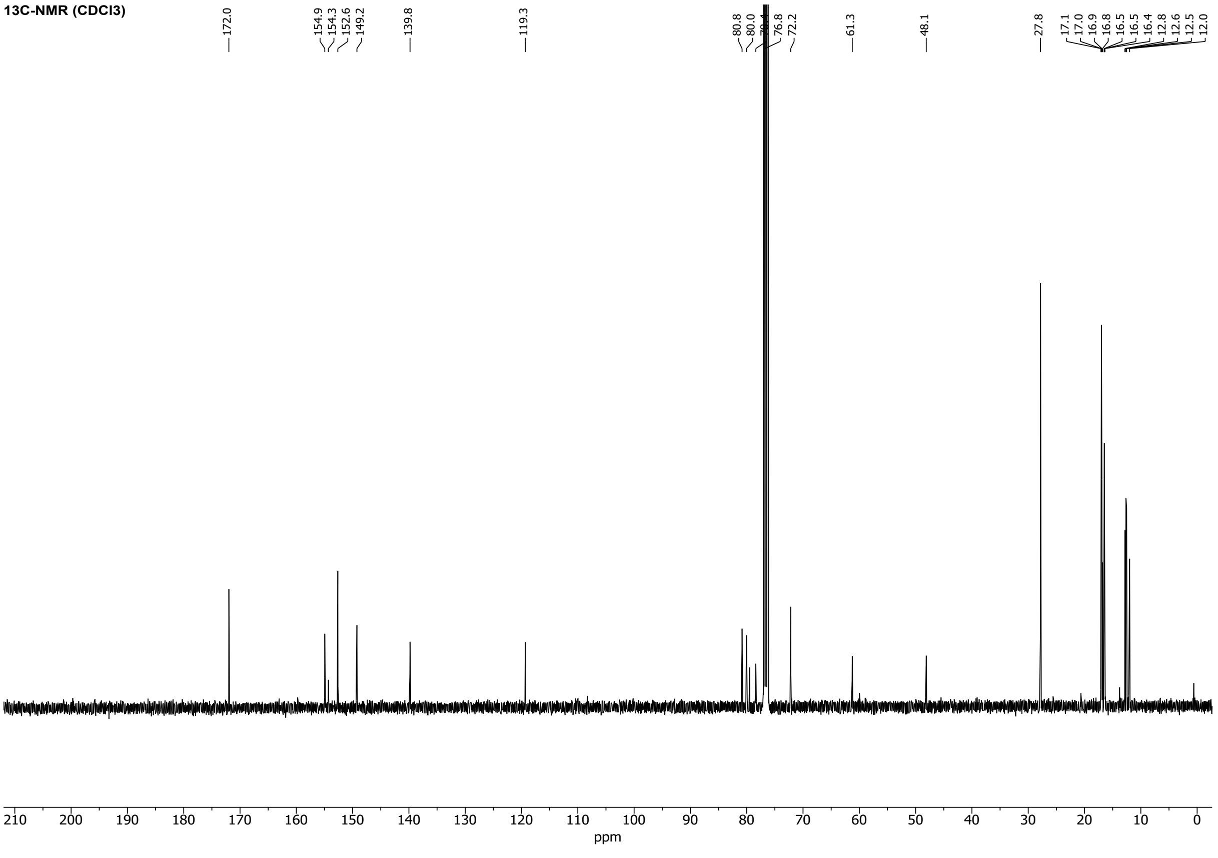

12a

1H-NMR (DMSO)

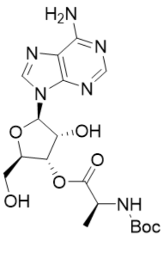

12a

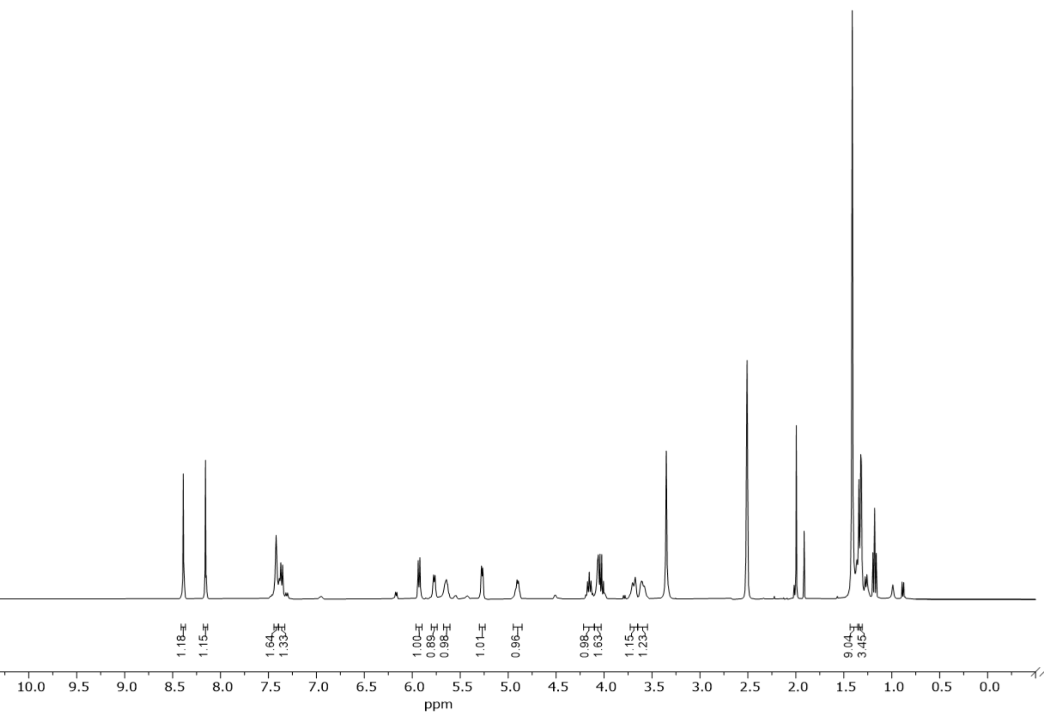

13C-NMR (DMSO)

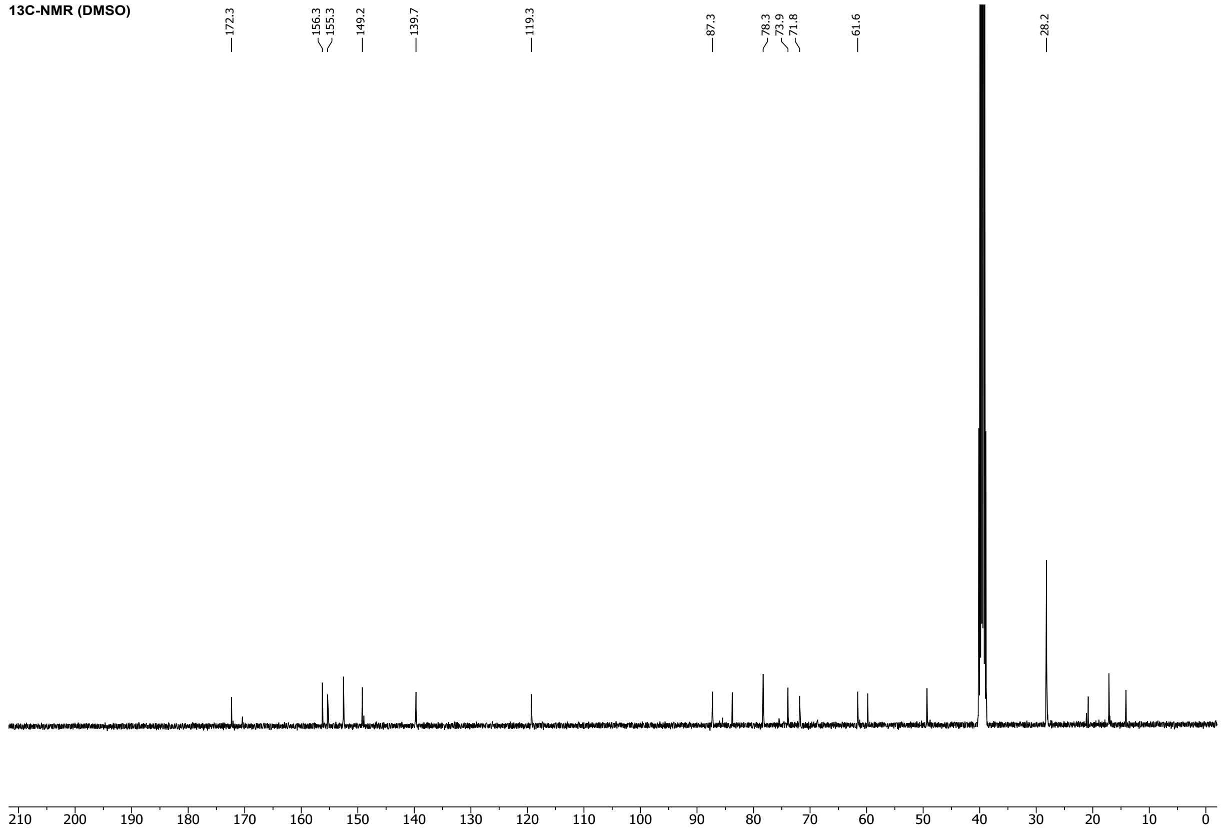

12b

1H-NMR (DMSO)

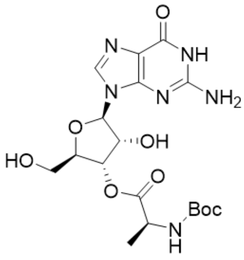

12b

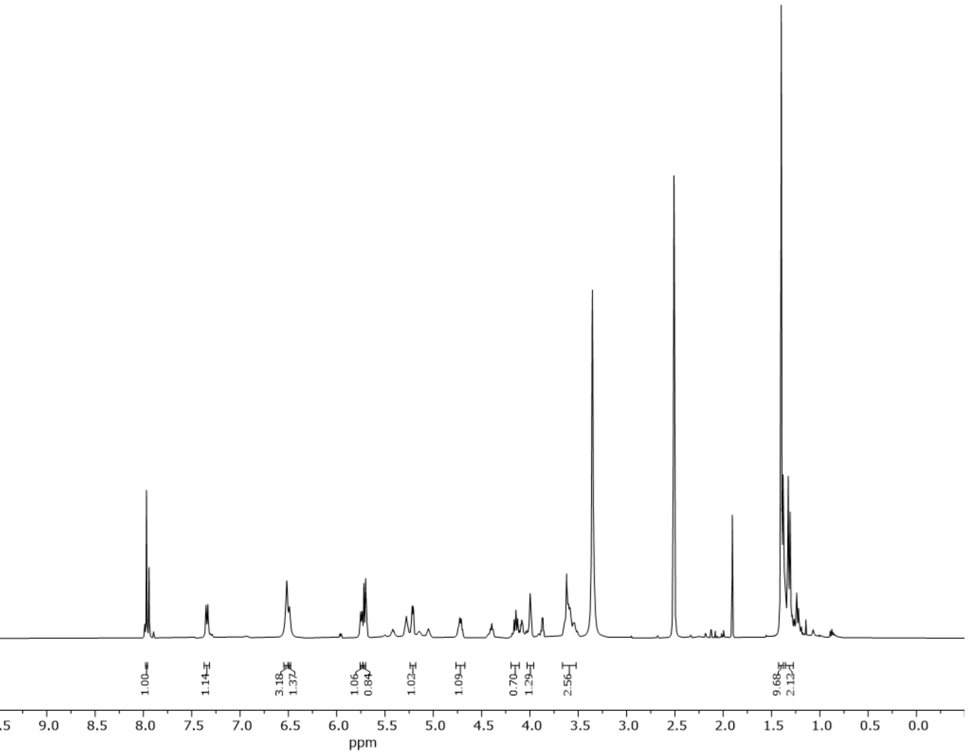

13C-NMR (DMSO)

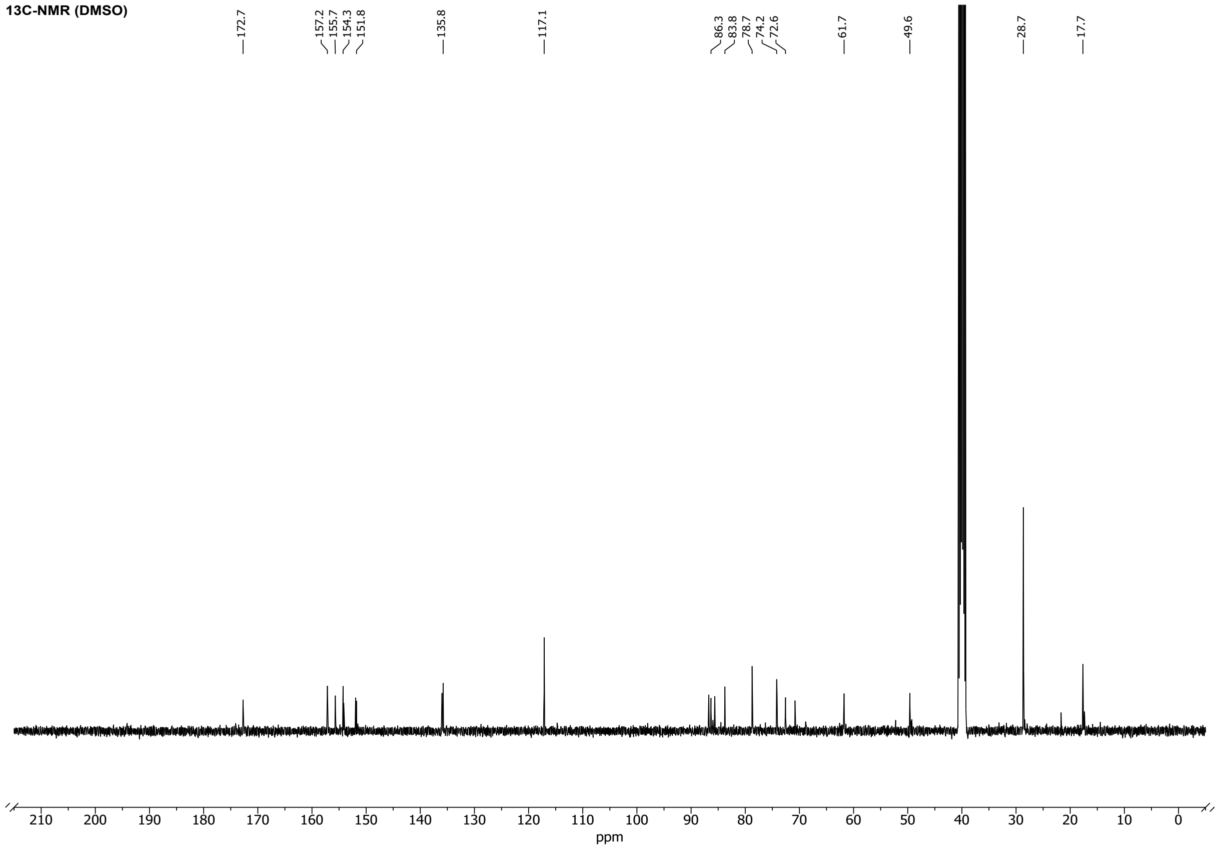

12c

1H-NMR (DMSO)

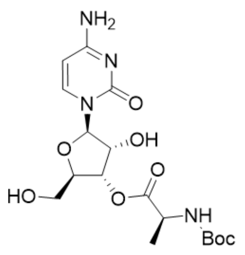

12c

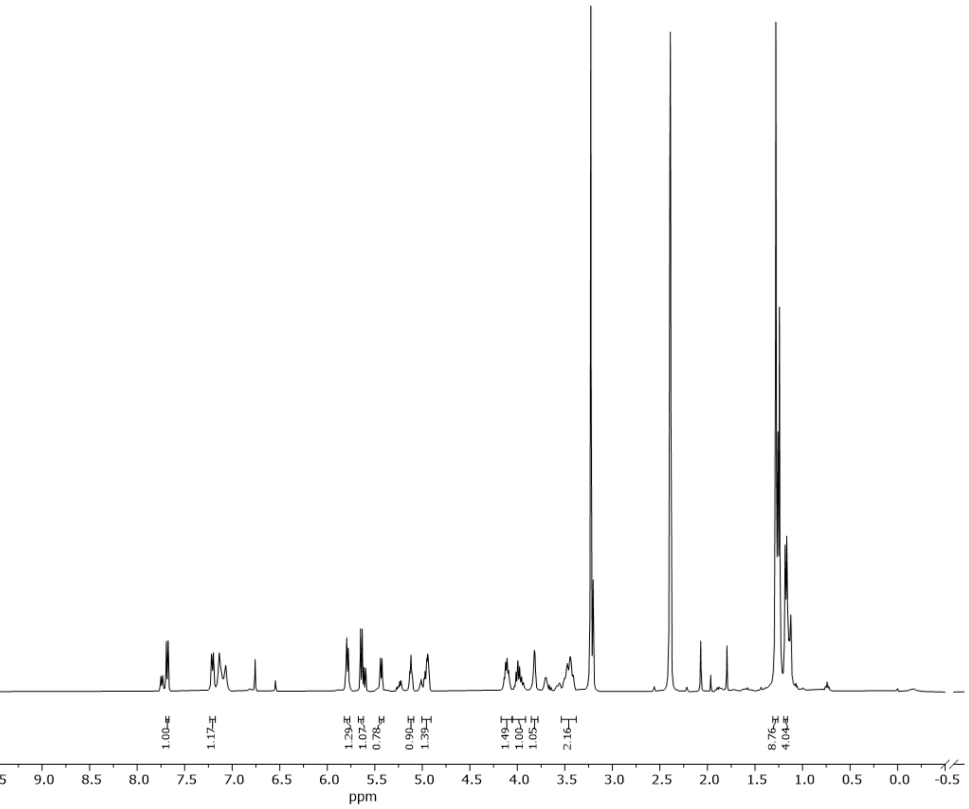

13C-NMR (DMSO)

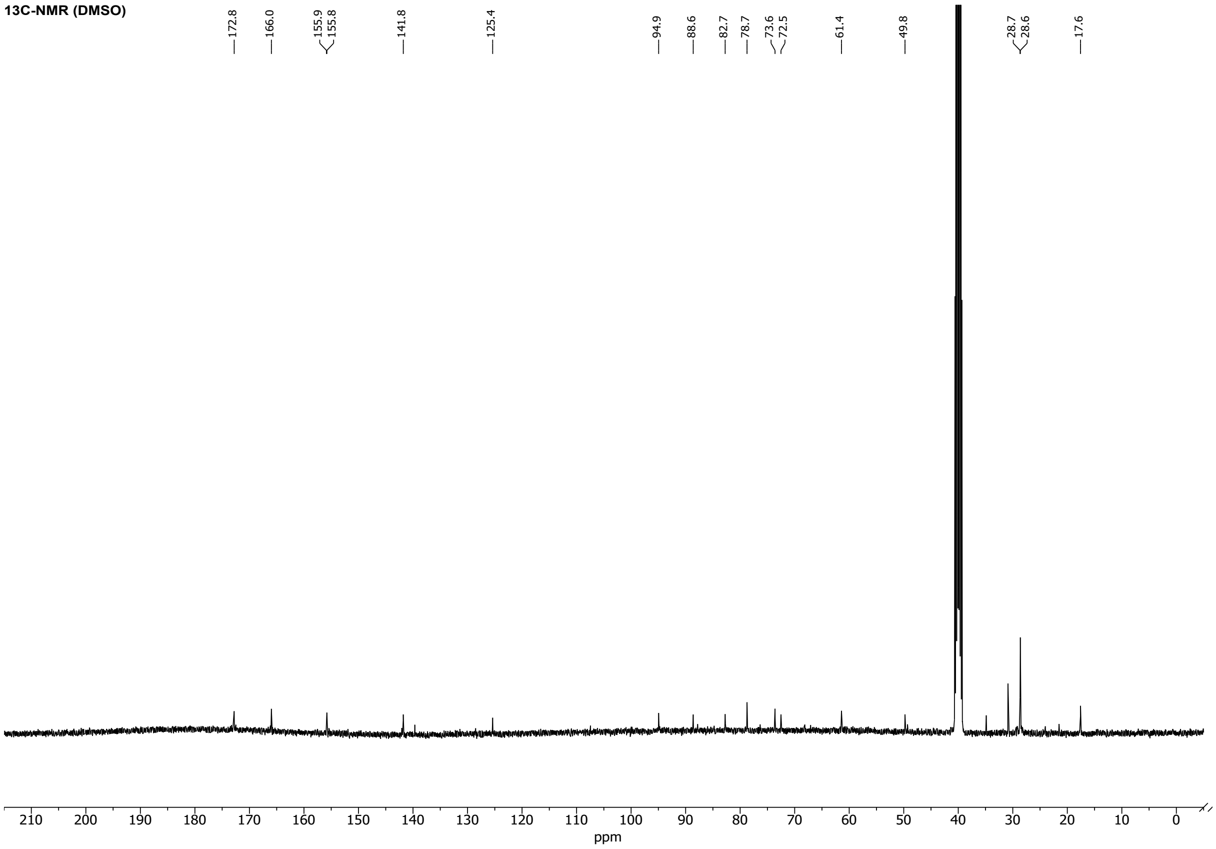

12d

1H-NMR (DMSO)

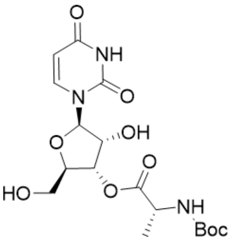

12d

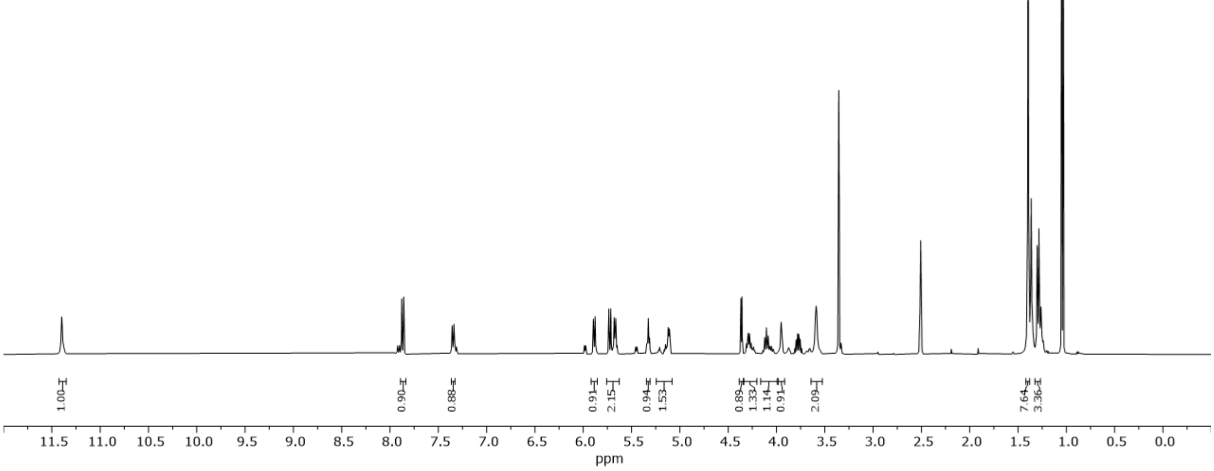

13C-NMR (DMSO)

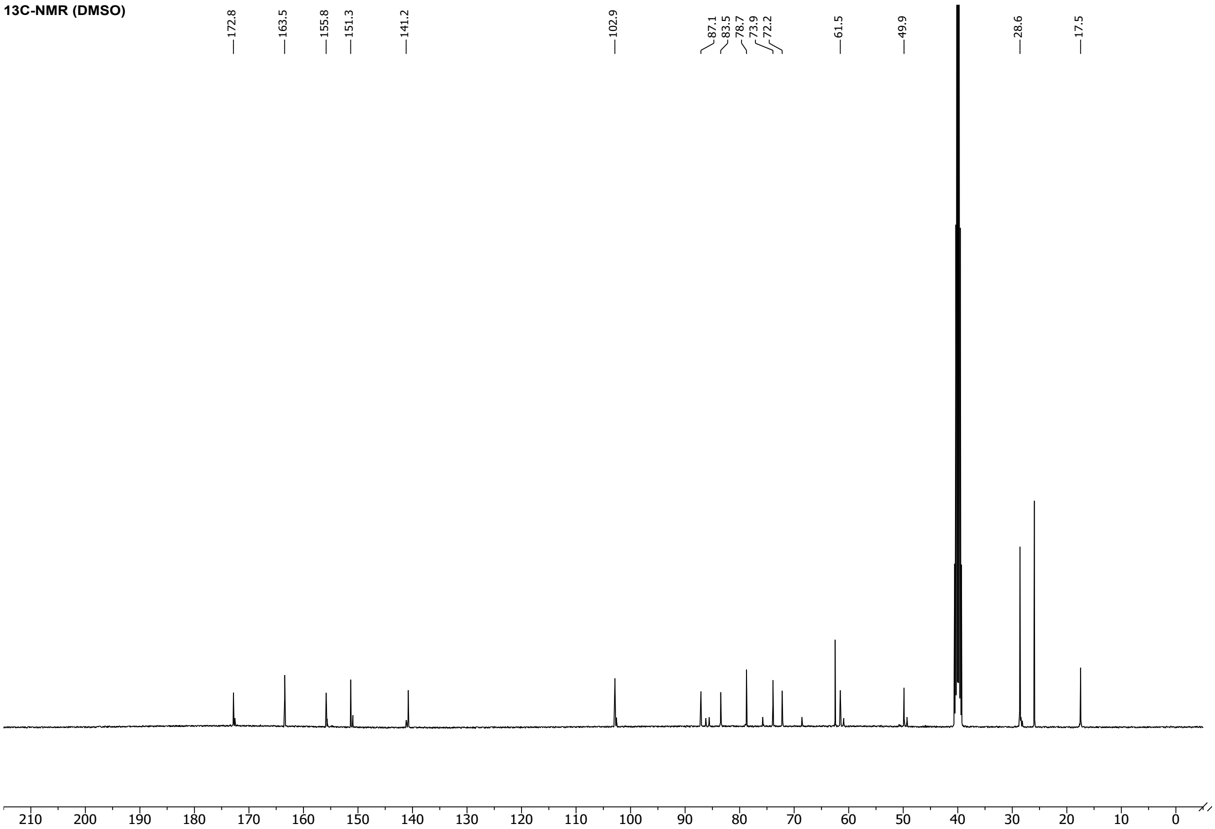

12e

1H-NMR (dmso)

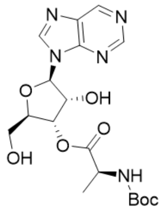

12e

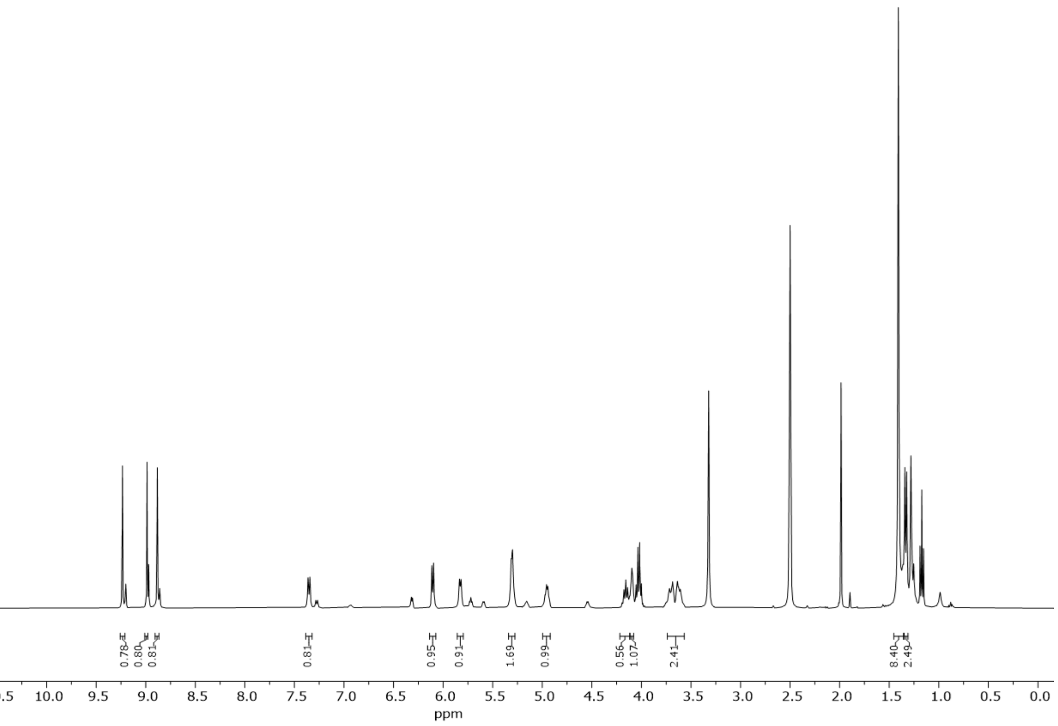

13C-NMR (dmso)

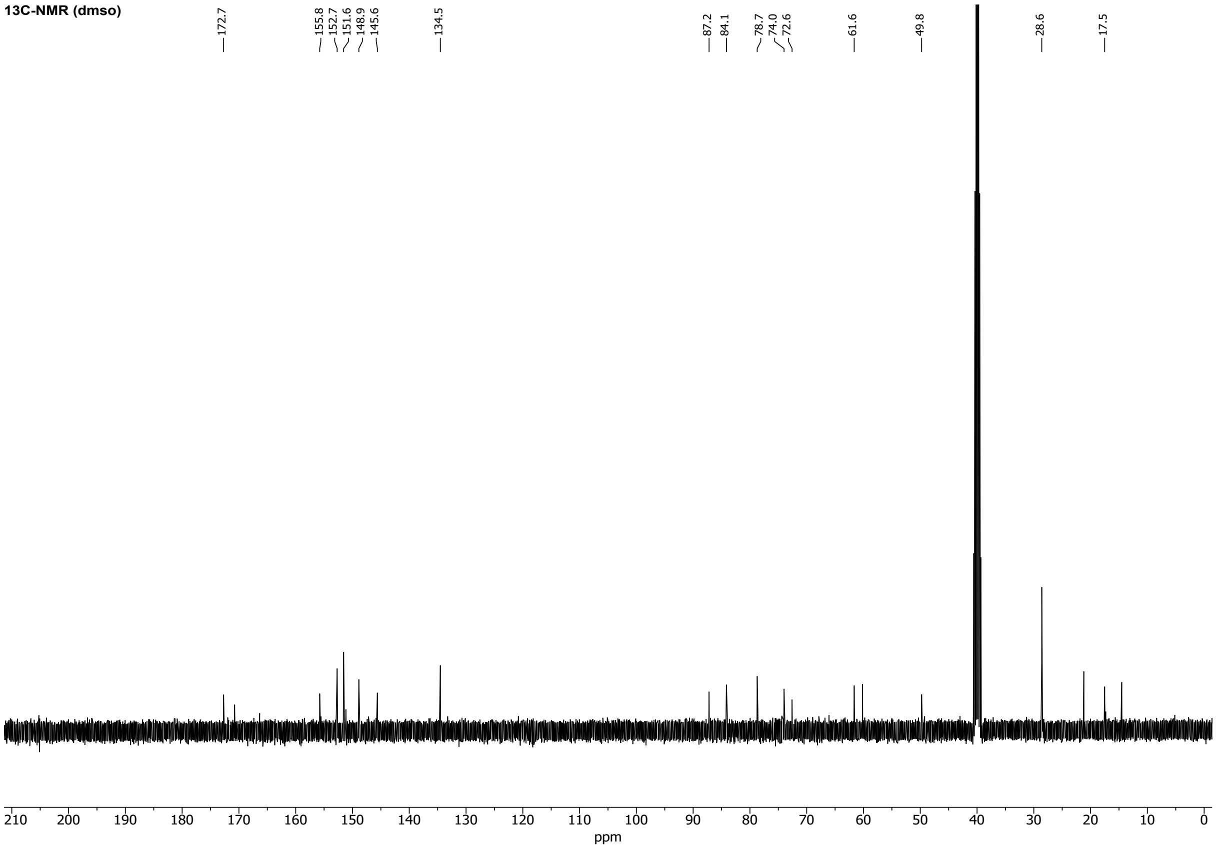

12f

1H-NMR (DMSO)

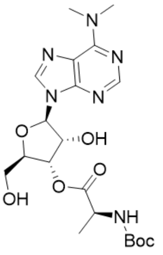

12f

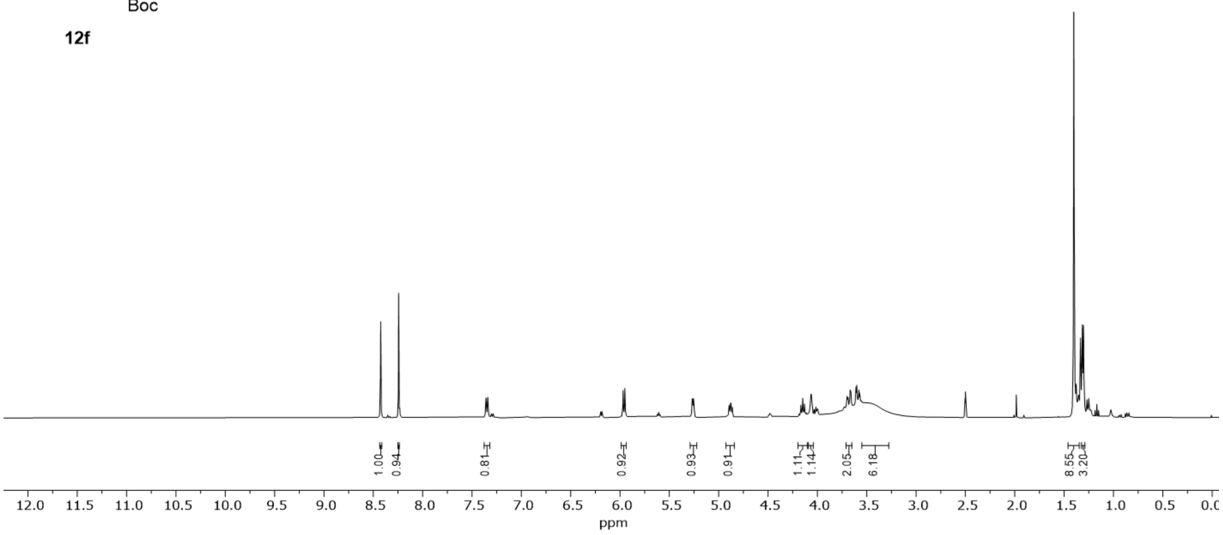

13C-NMR (DMSO)

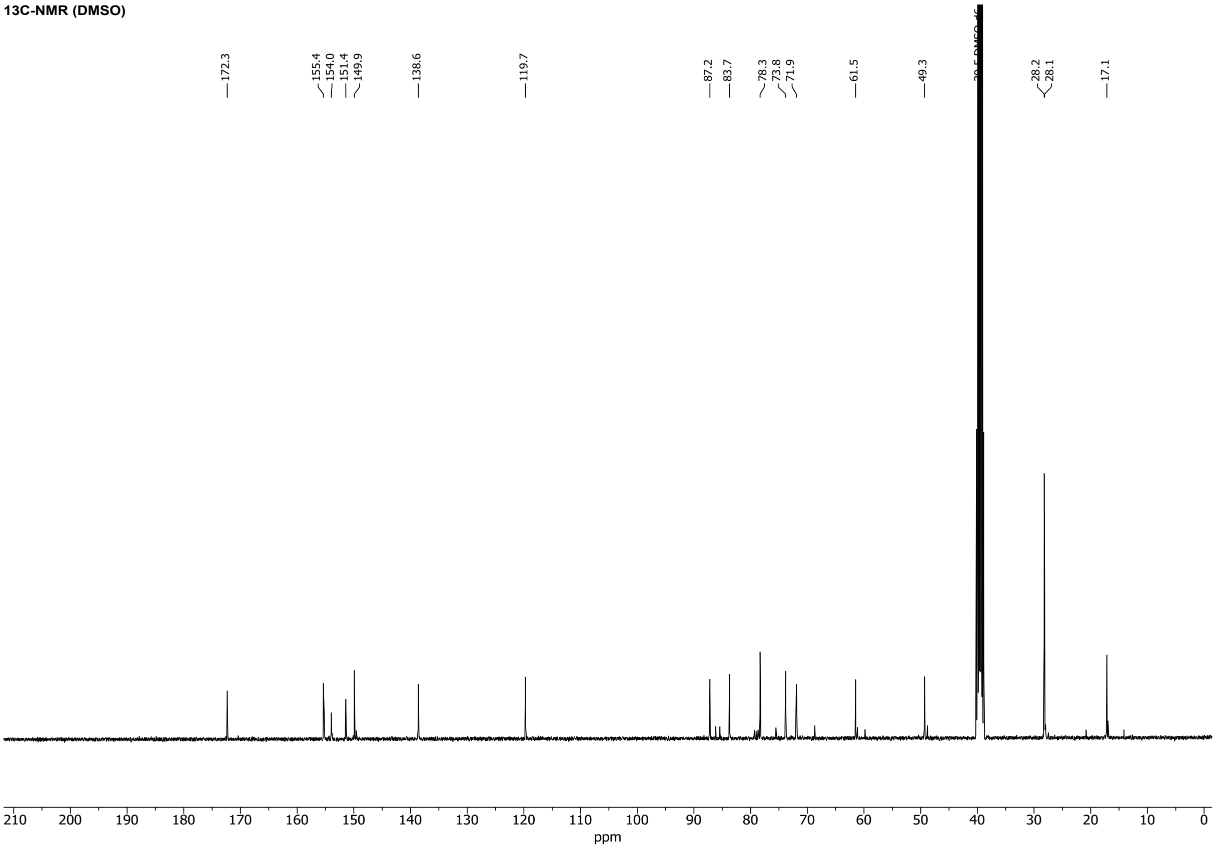

13a

1H-NMR (CDCl3)

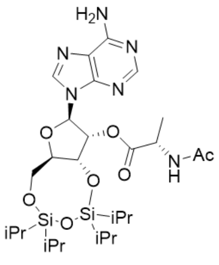

13a

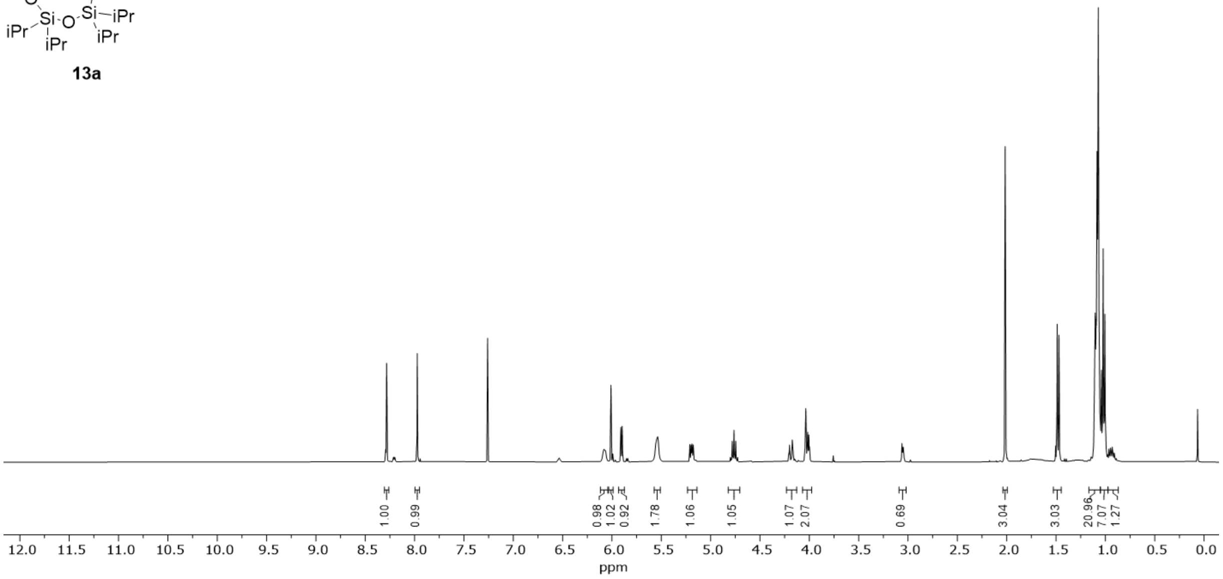

13C-NMR (CDCl3)

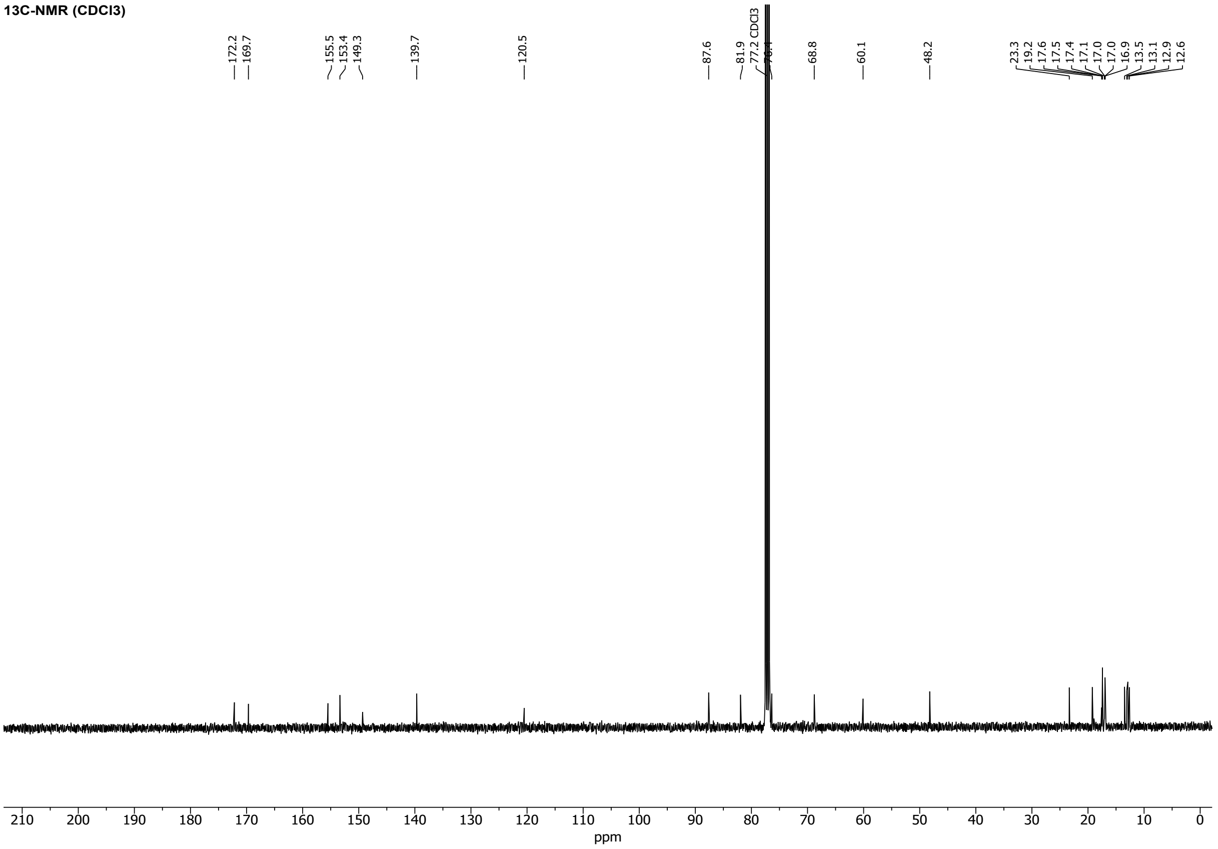

13e

1H-NMR (CDCl3)

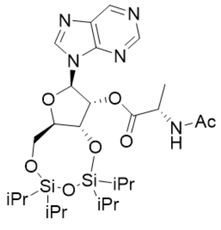

13e

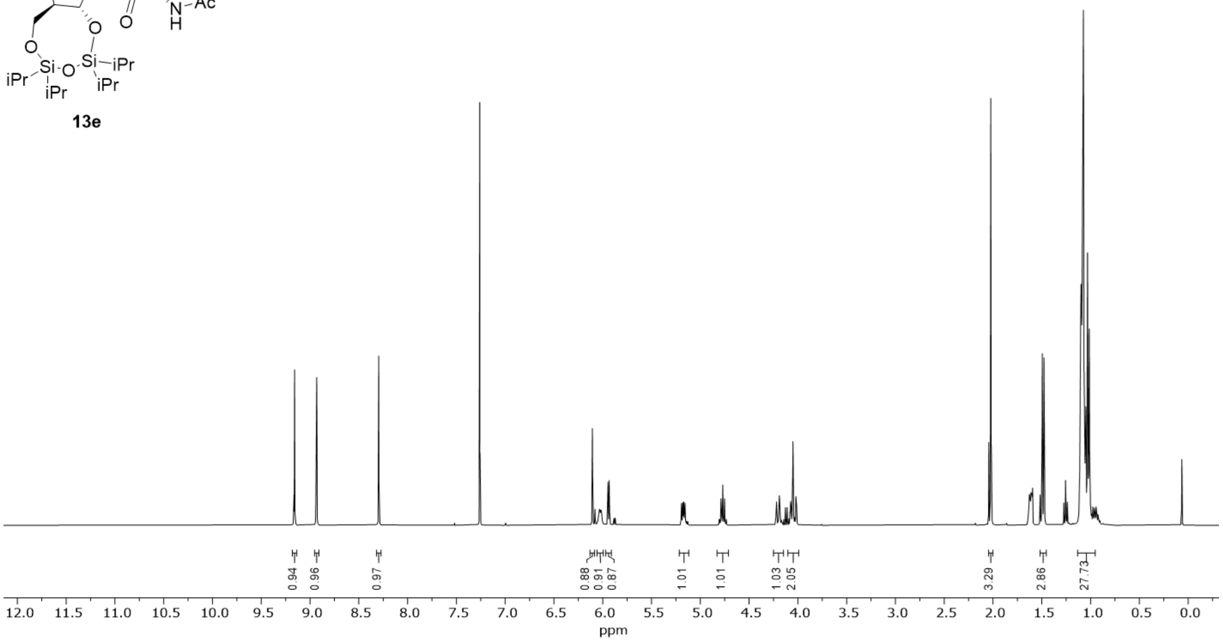

13C-NMR (CDCl3)

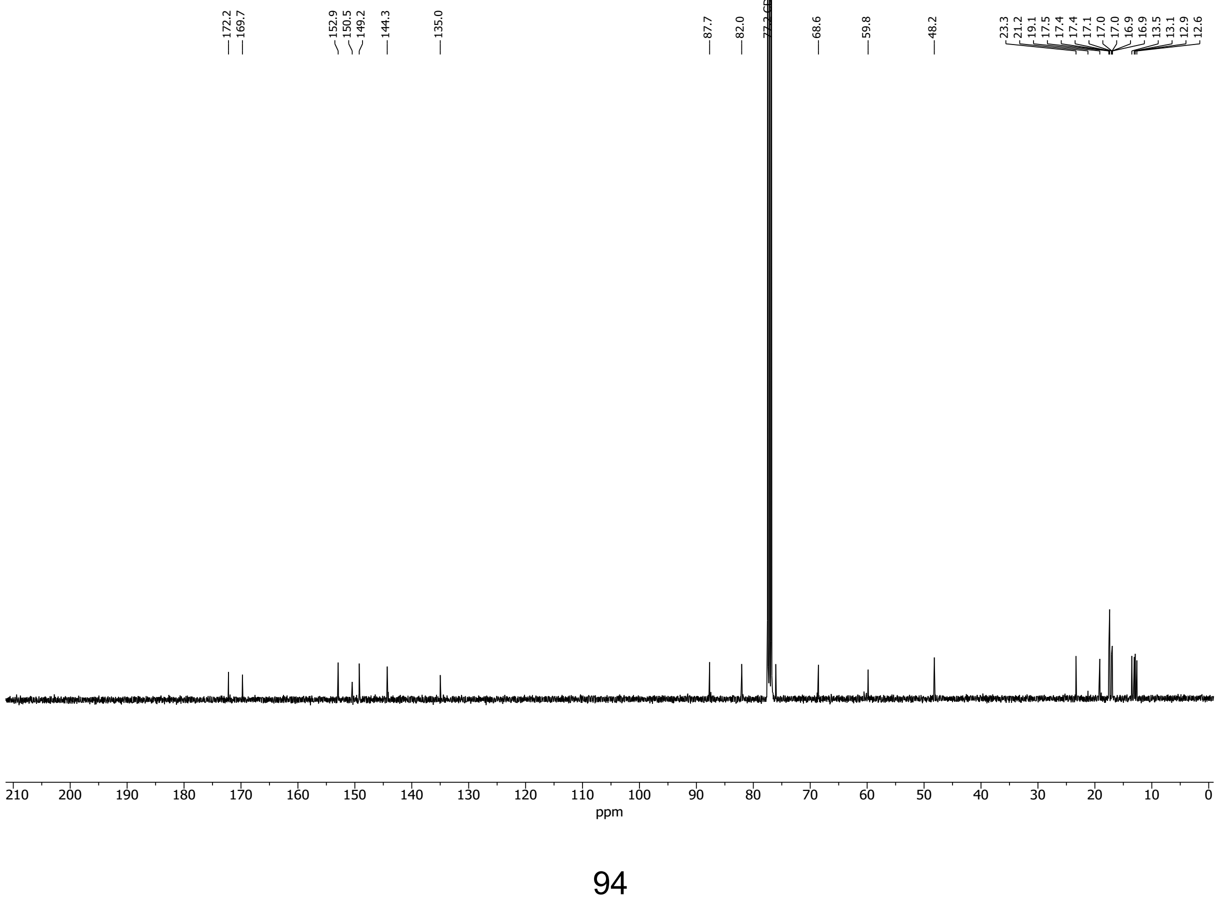

13f

1H-NMR (CDCl3)

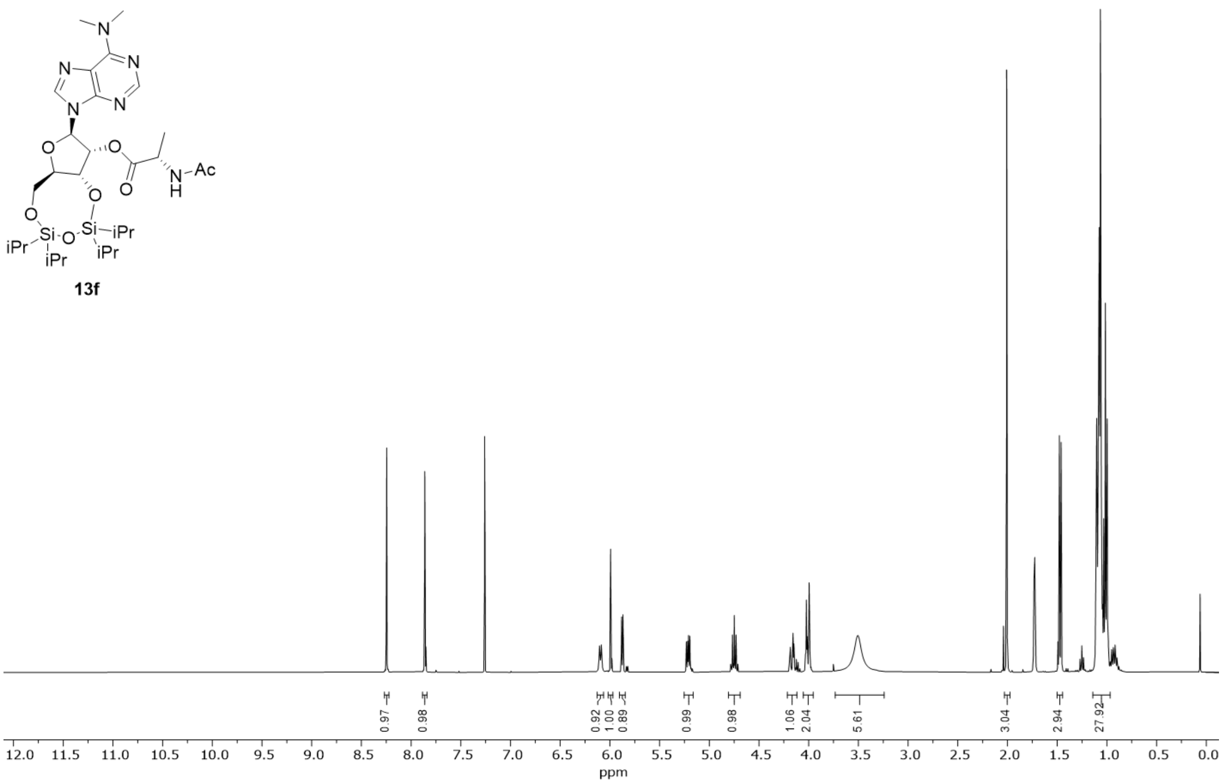

13C-NMR (CDCl3)

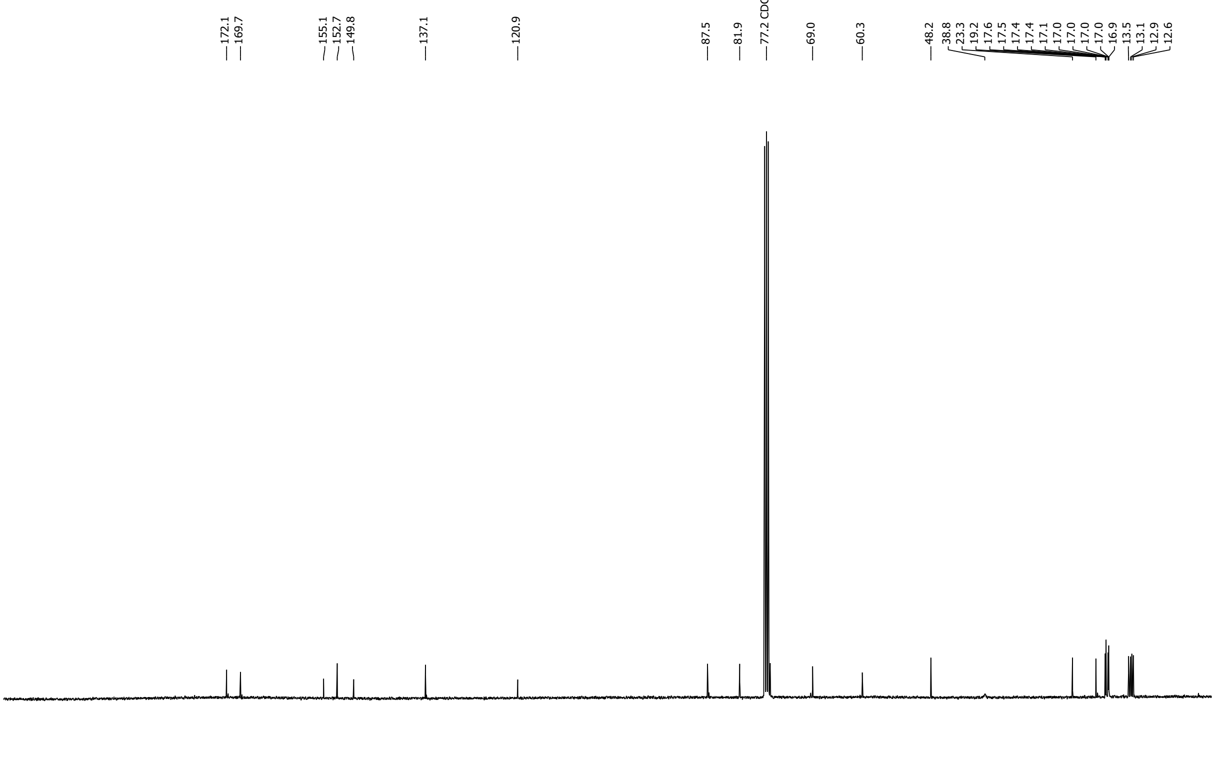

14a

1H-NMR (CDCl3)

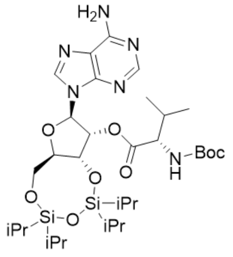

14a

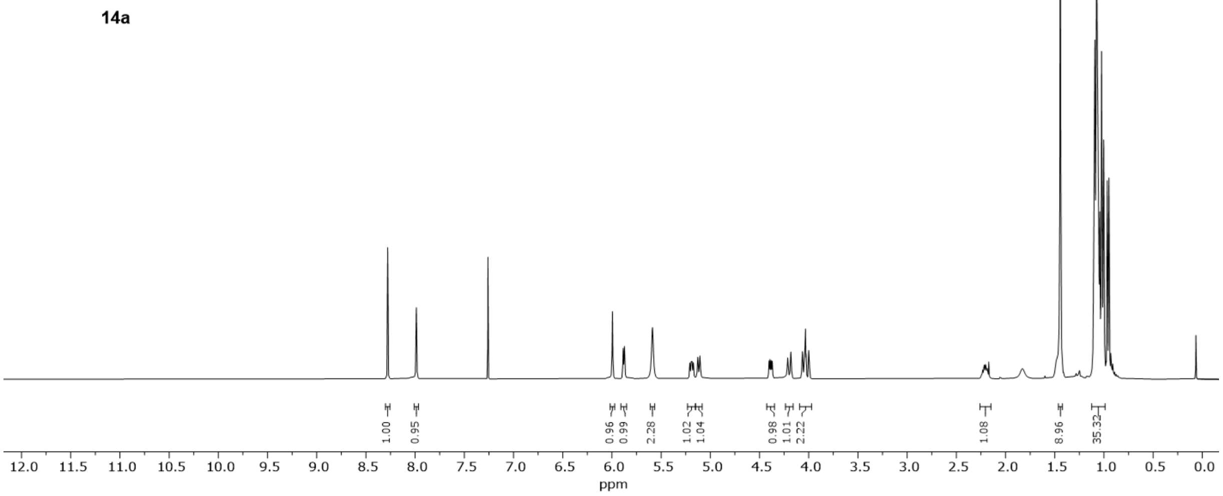

13C-NMR (CDCl3)

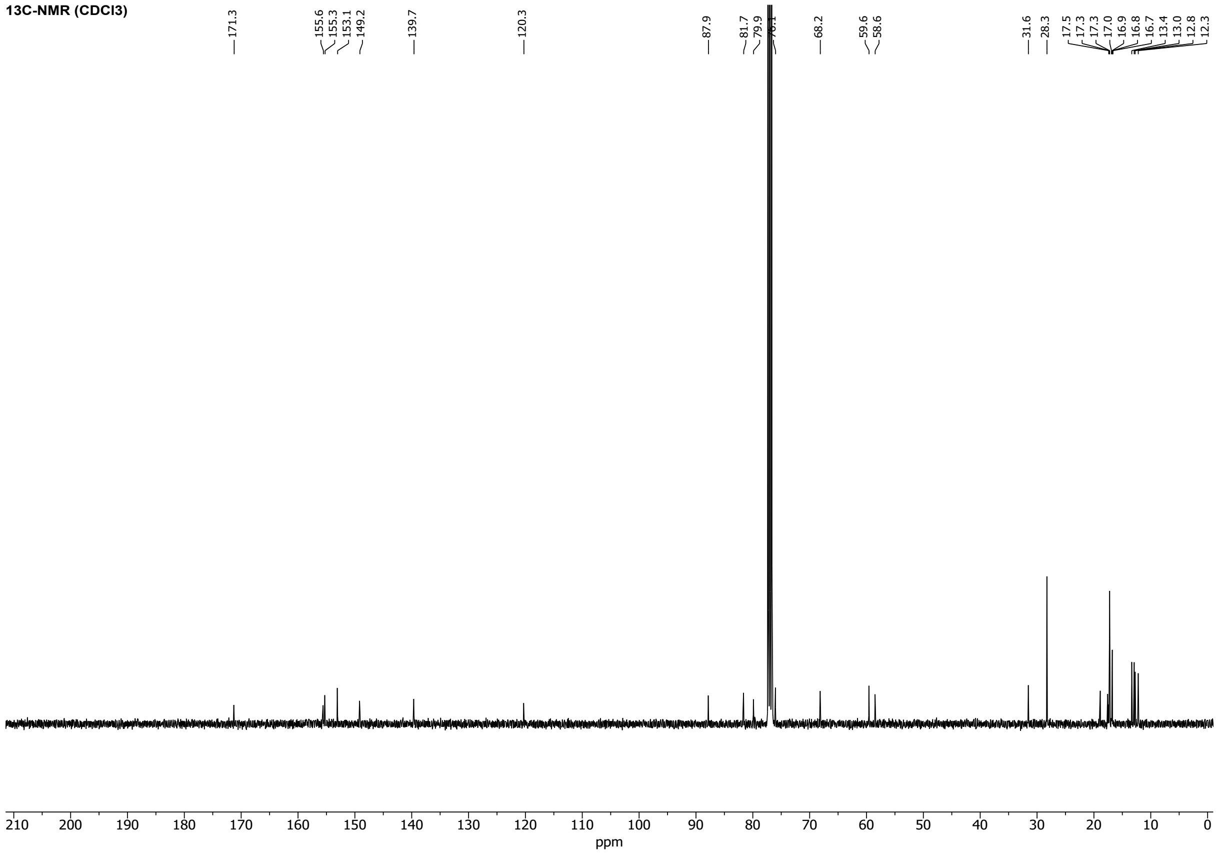

15a

1H-NMR (CDCl3)

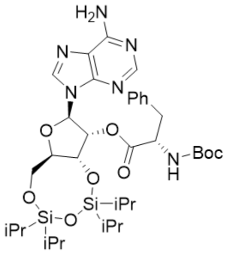

15a

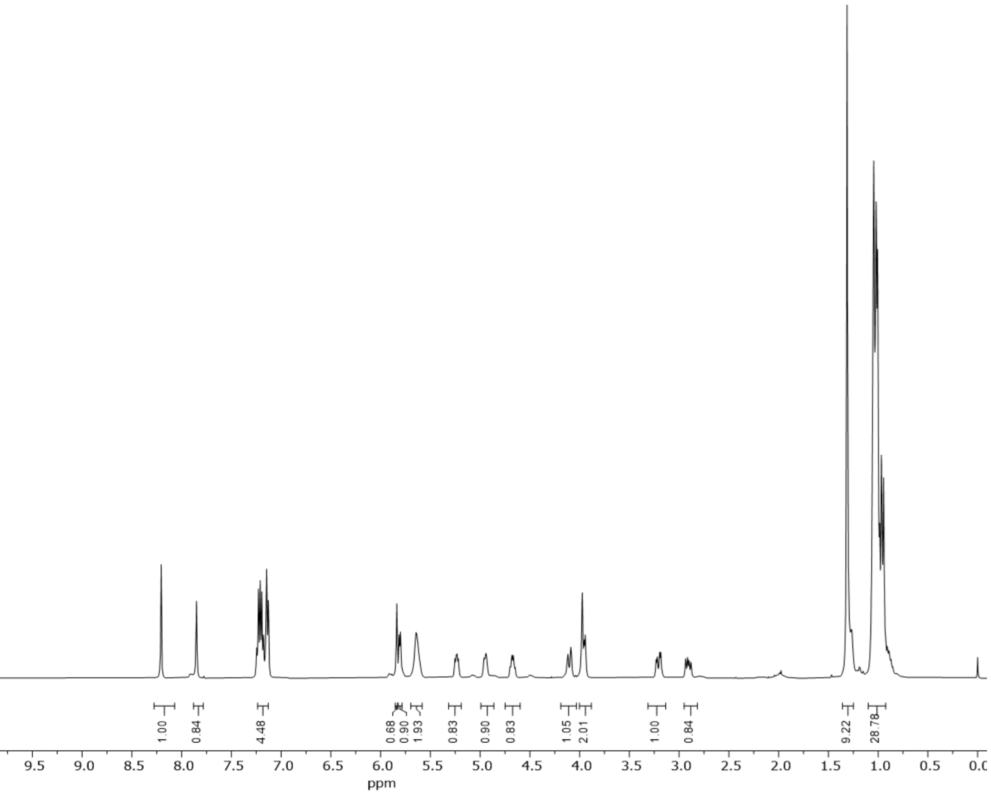

13C-NMR (CDCl3)

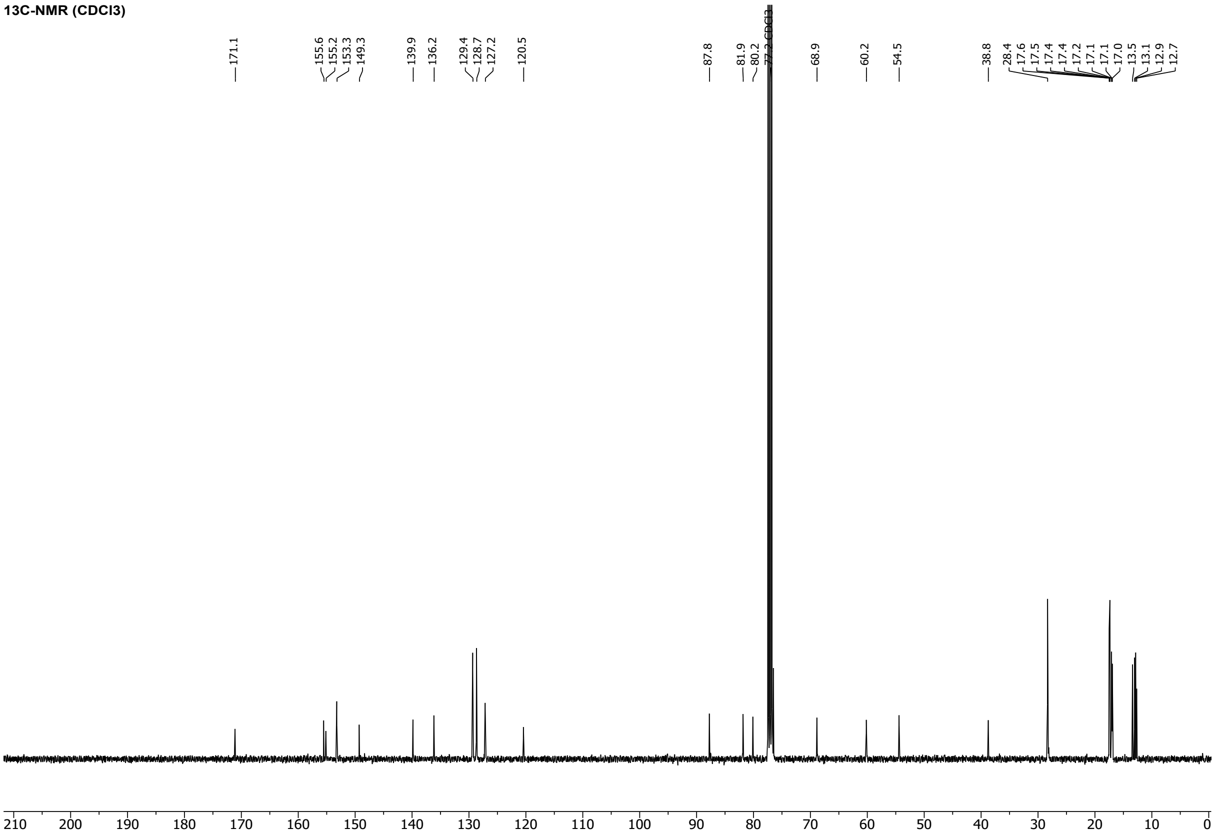

16a

1H-NMR (DMSO)

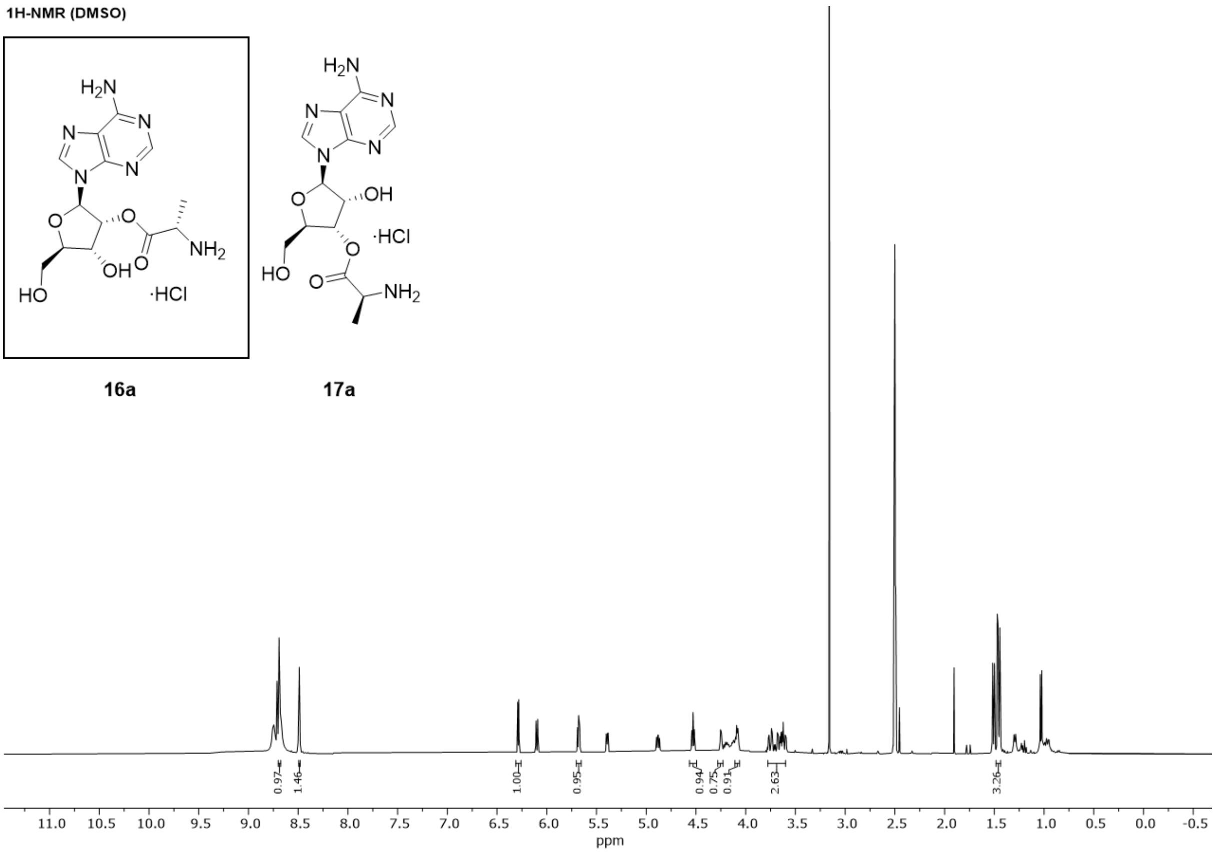

13C-NMR (DMSO)

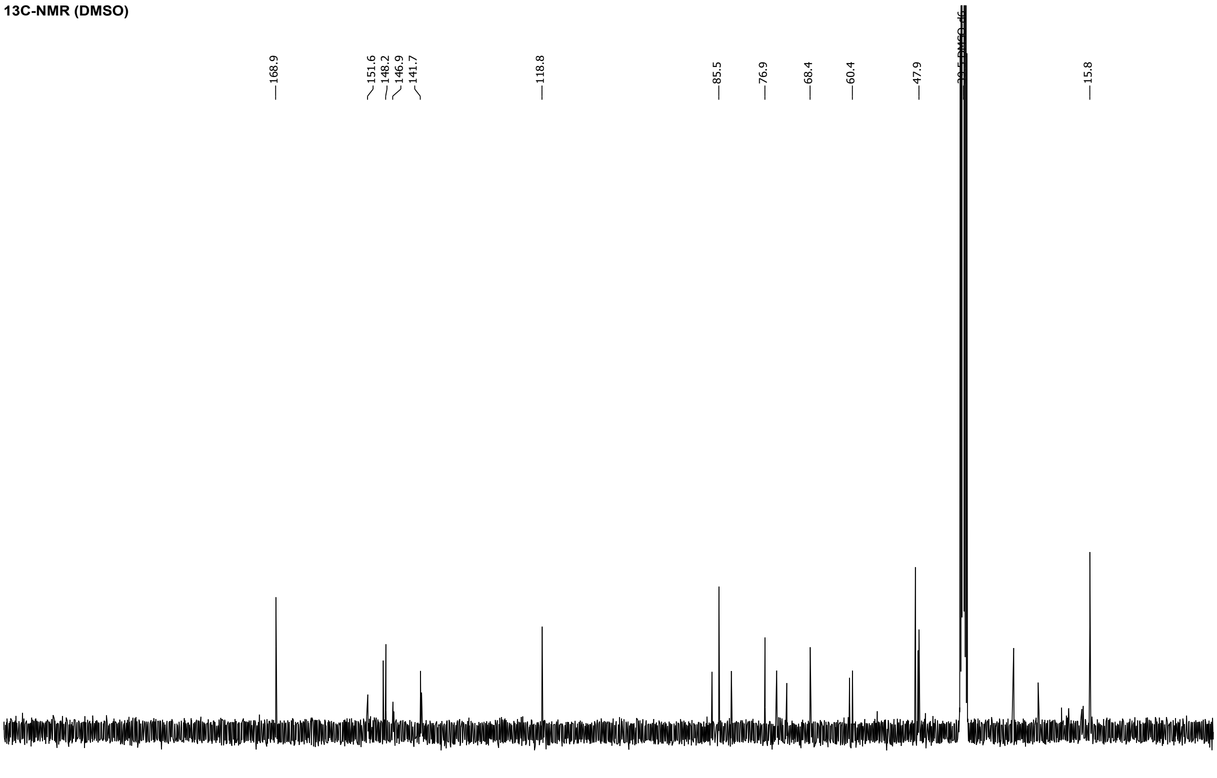

16g

1H-NMR (DMSO)

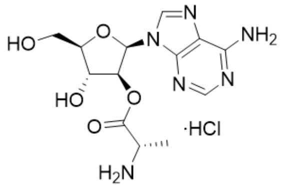

16g

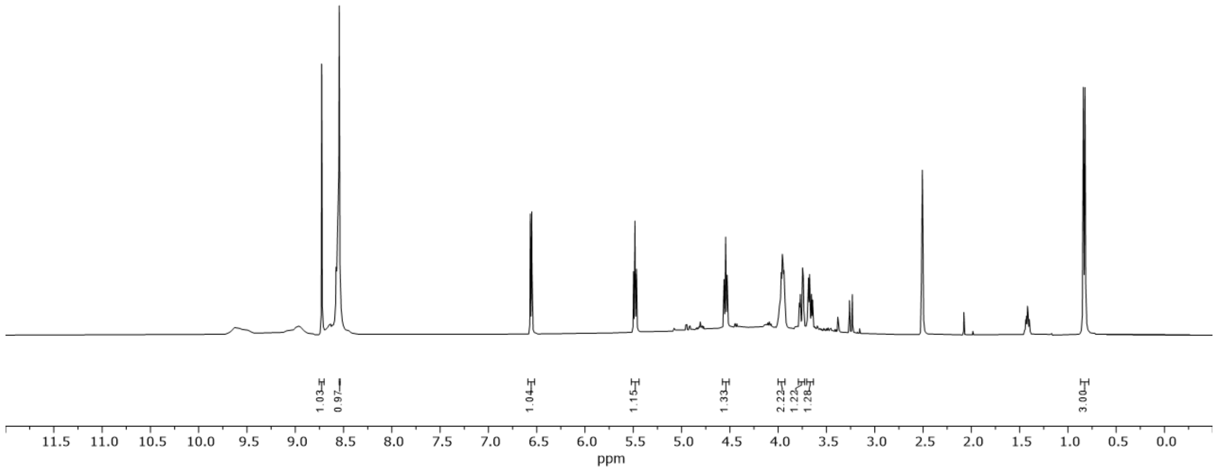

13C-NMR (DMSO)

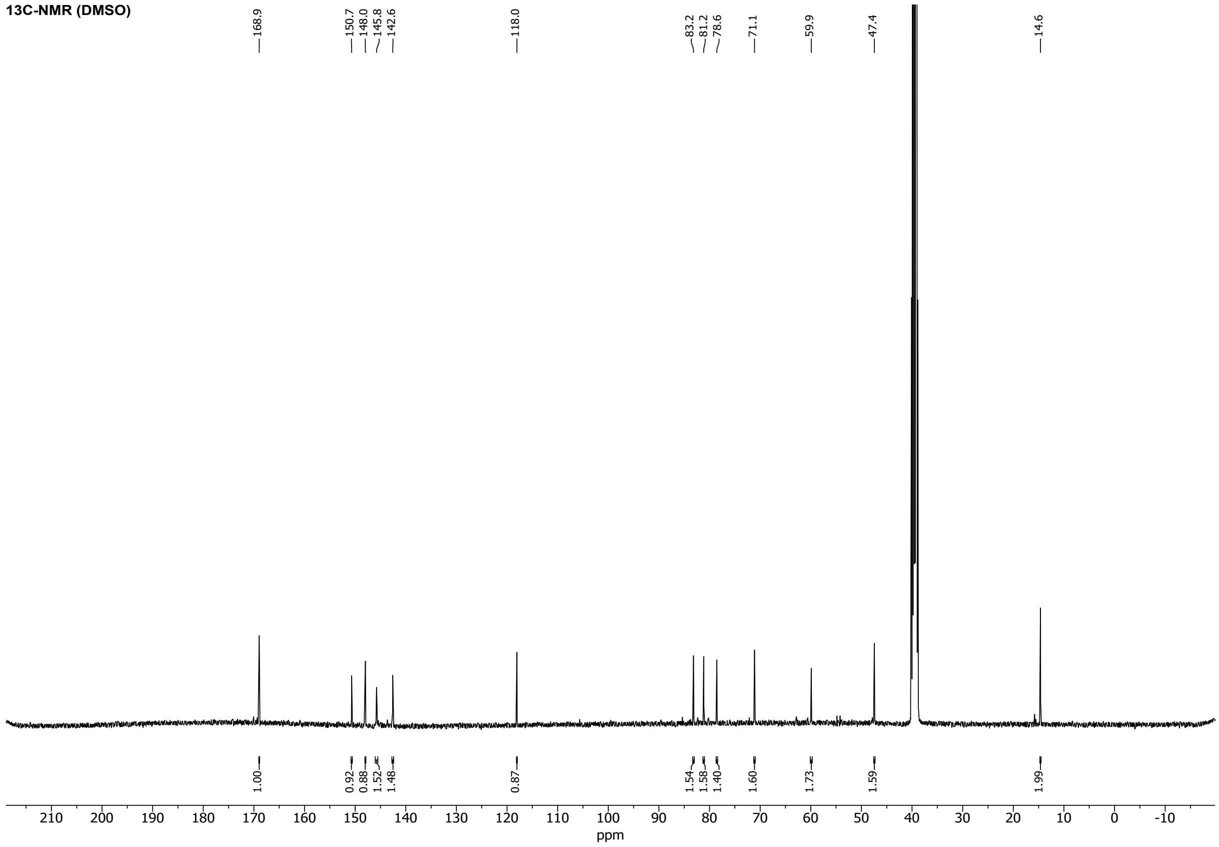

17a

1H-NMR (DMSO)

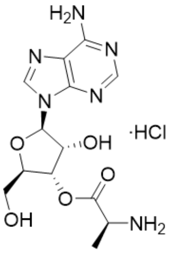

17a

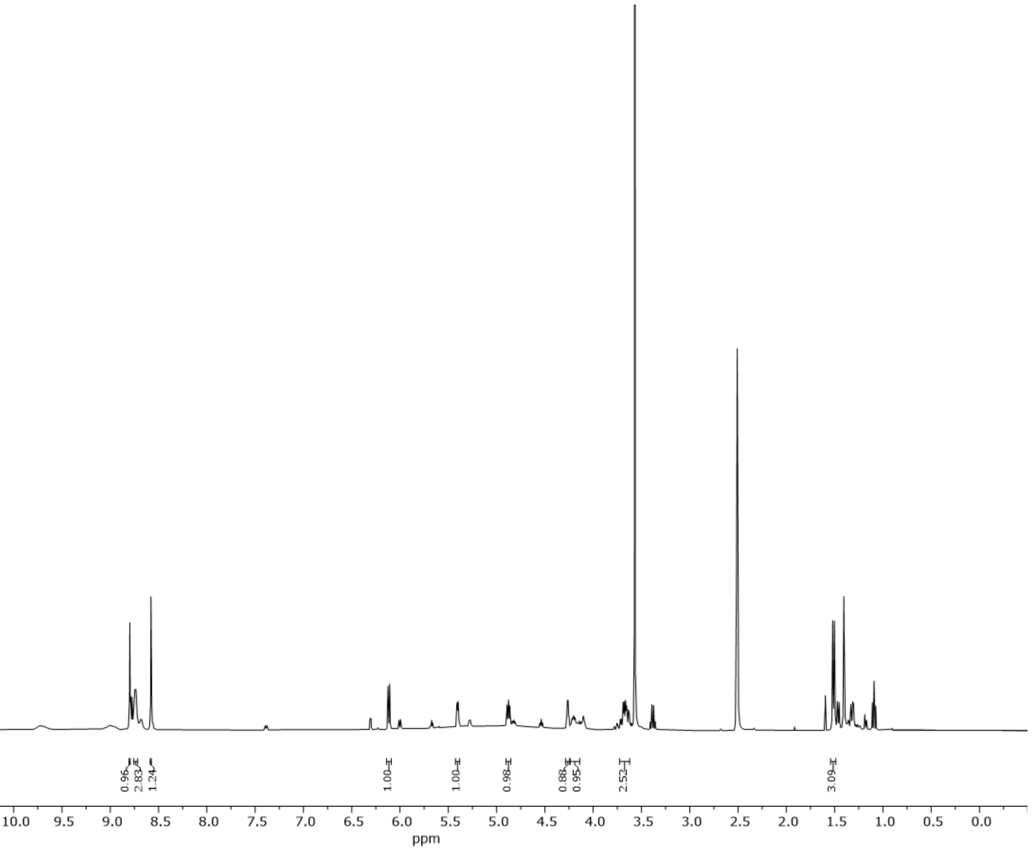

13C-NMR (DMSO)

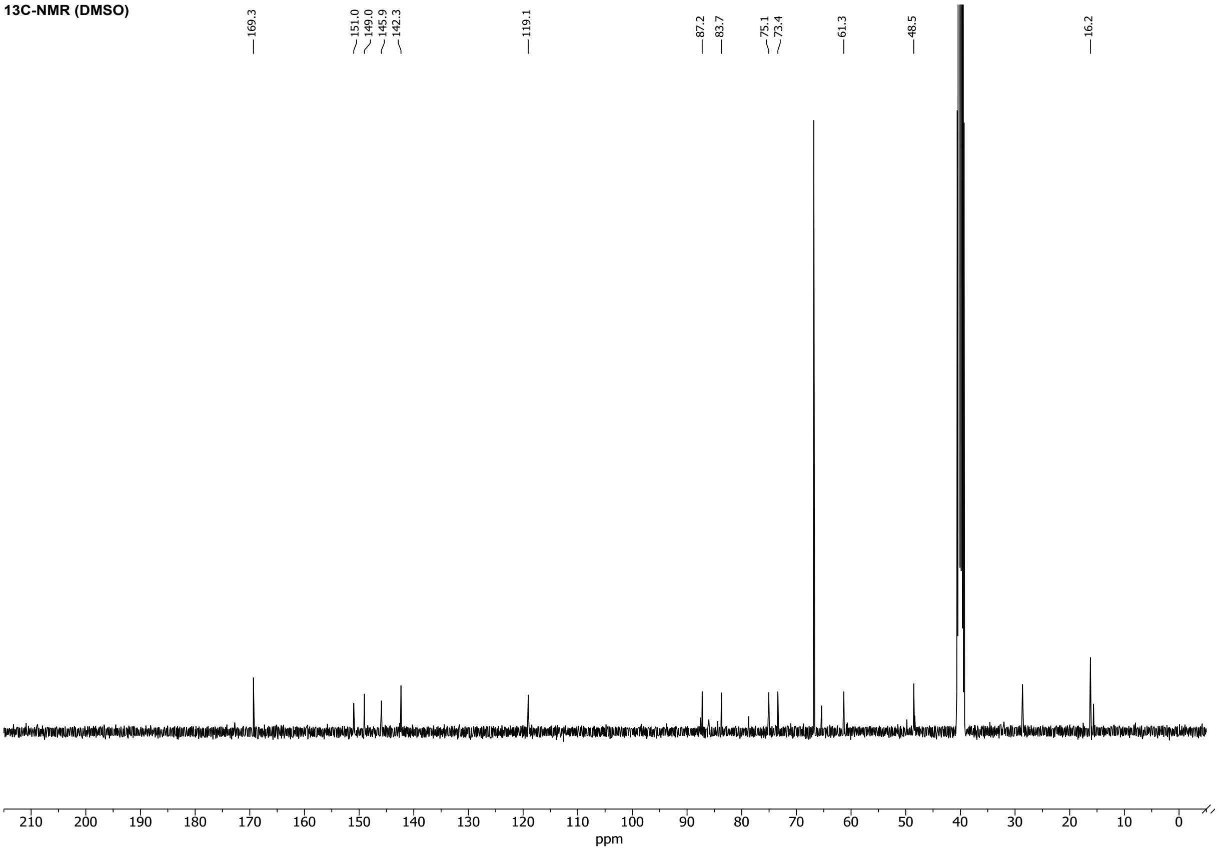

18a

1H-NMR (DMSO)

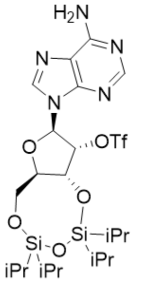

18a

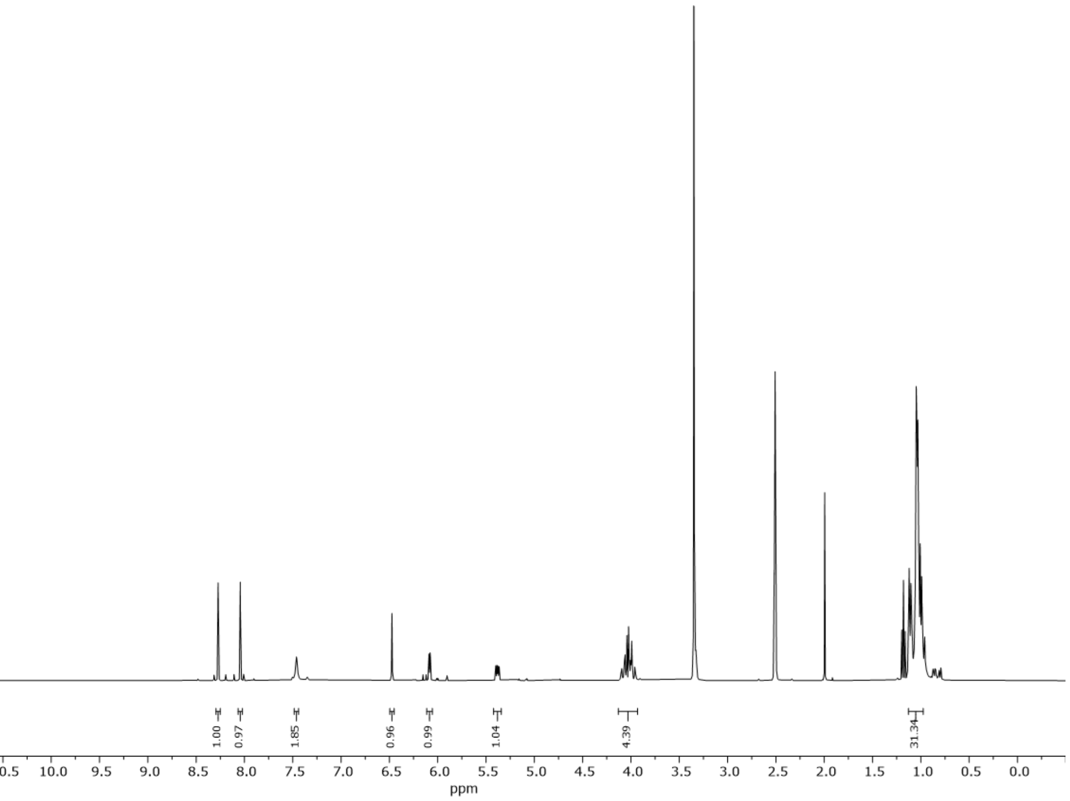

13C-NMR (DMSO)

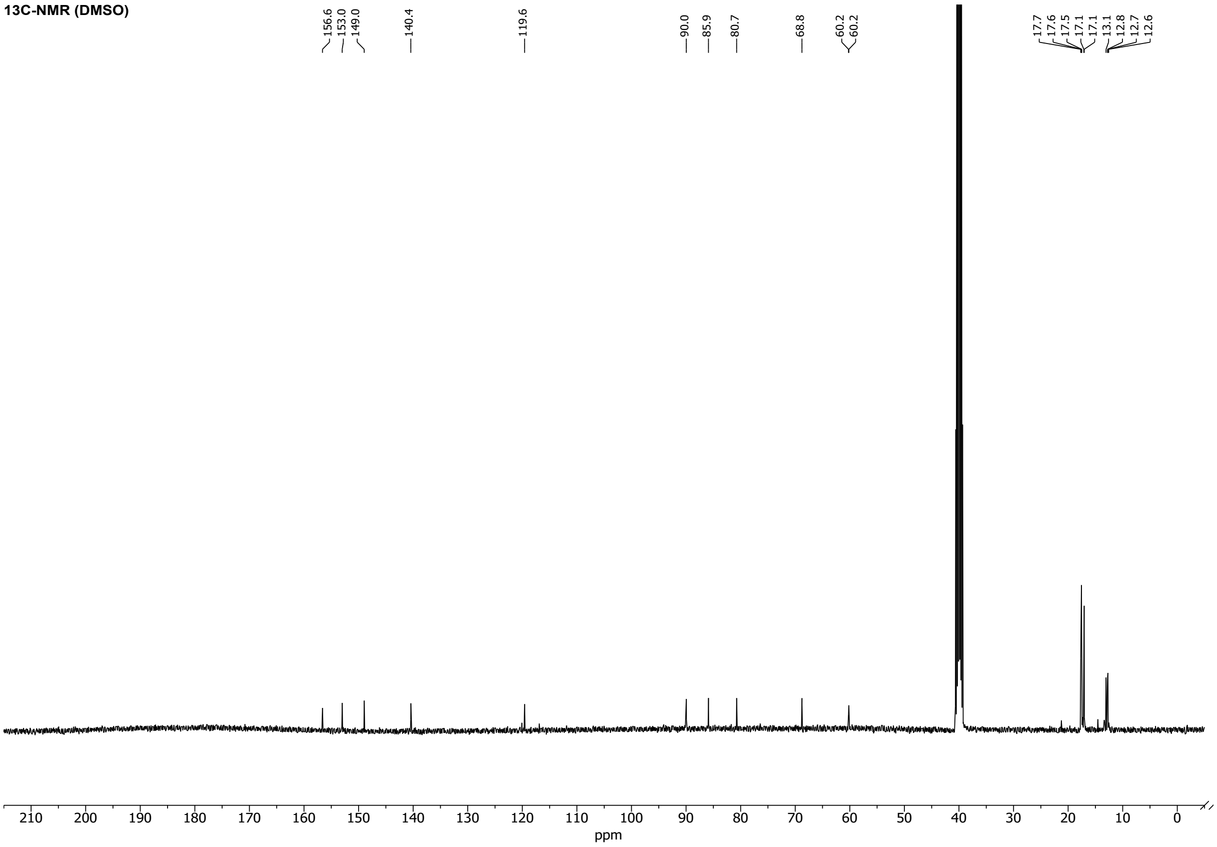

19a

1H-NMR (CD3CN)

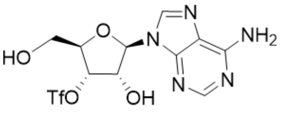

19a

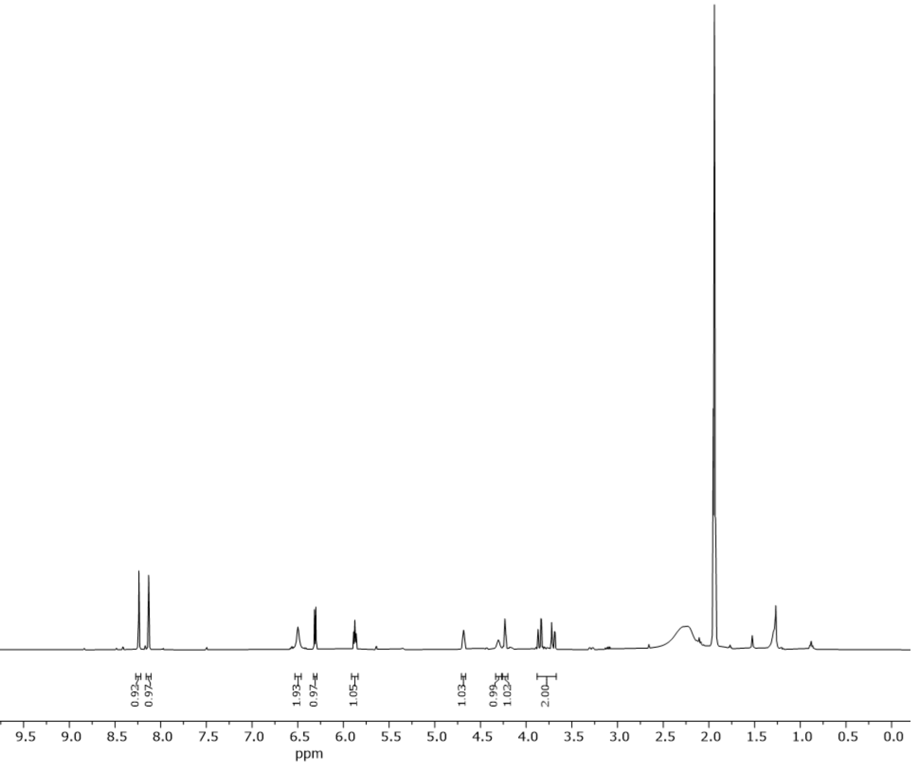

13C-NMR (CD3CN)

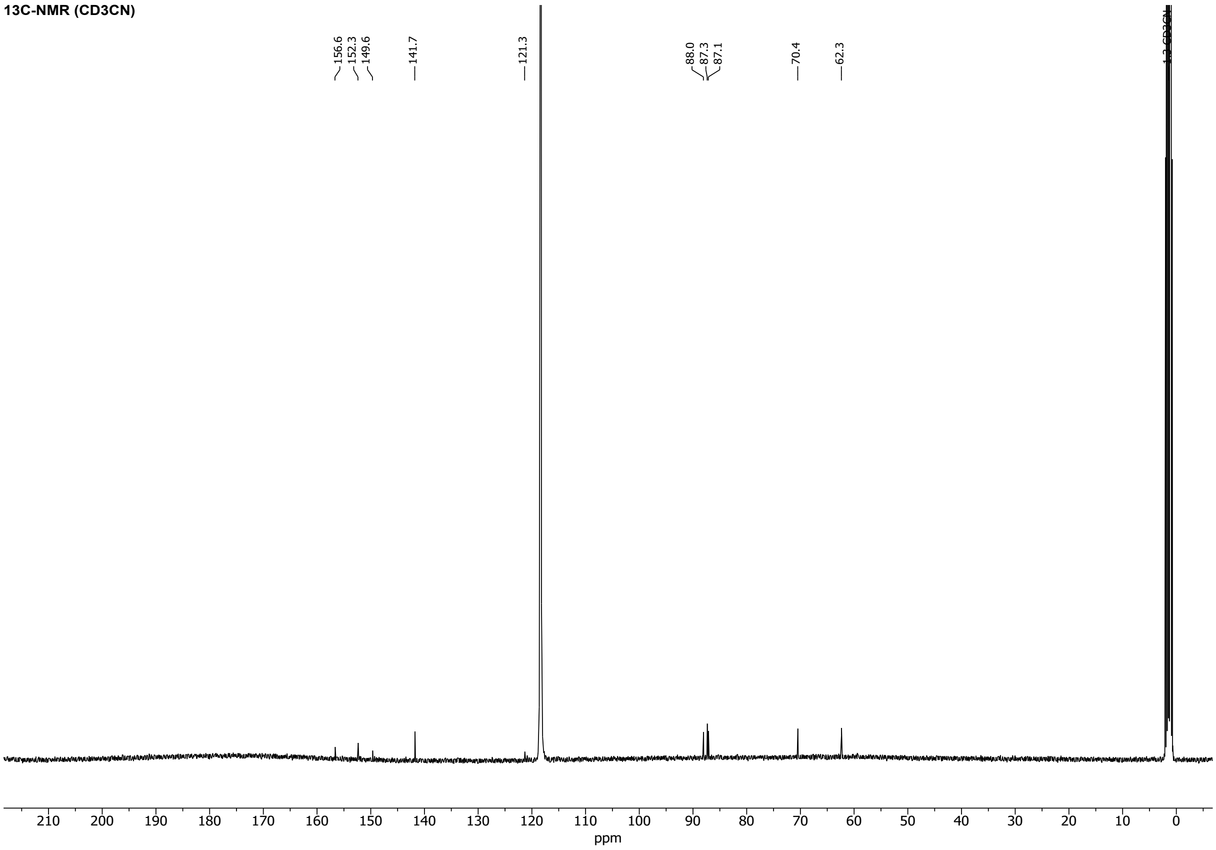

22a

1H-NMR (DMSO)

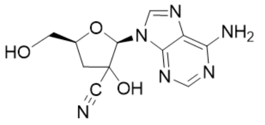

22a

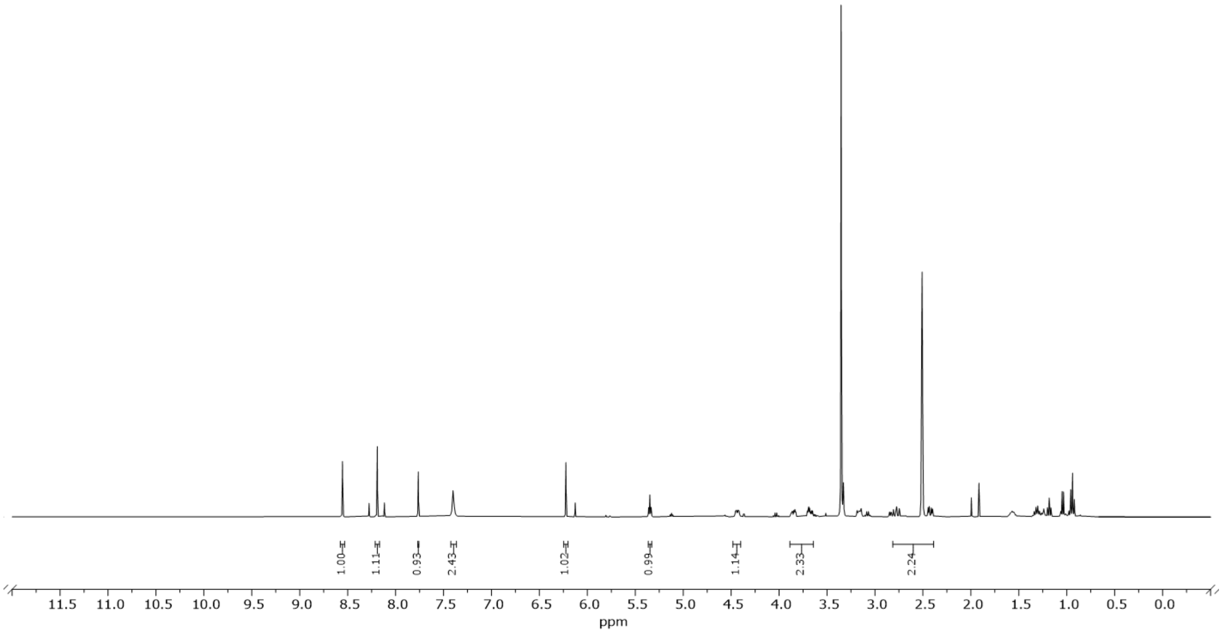

13C-NMR (DMSO)

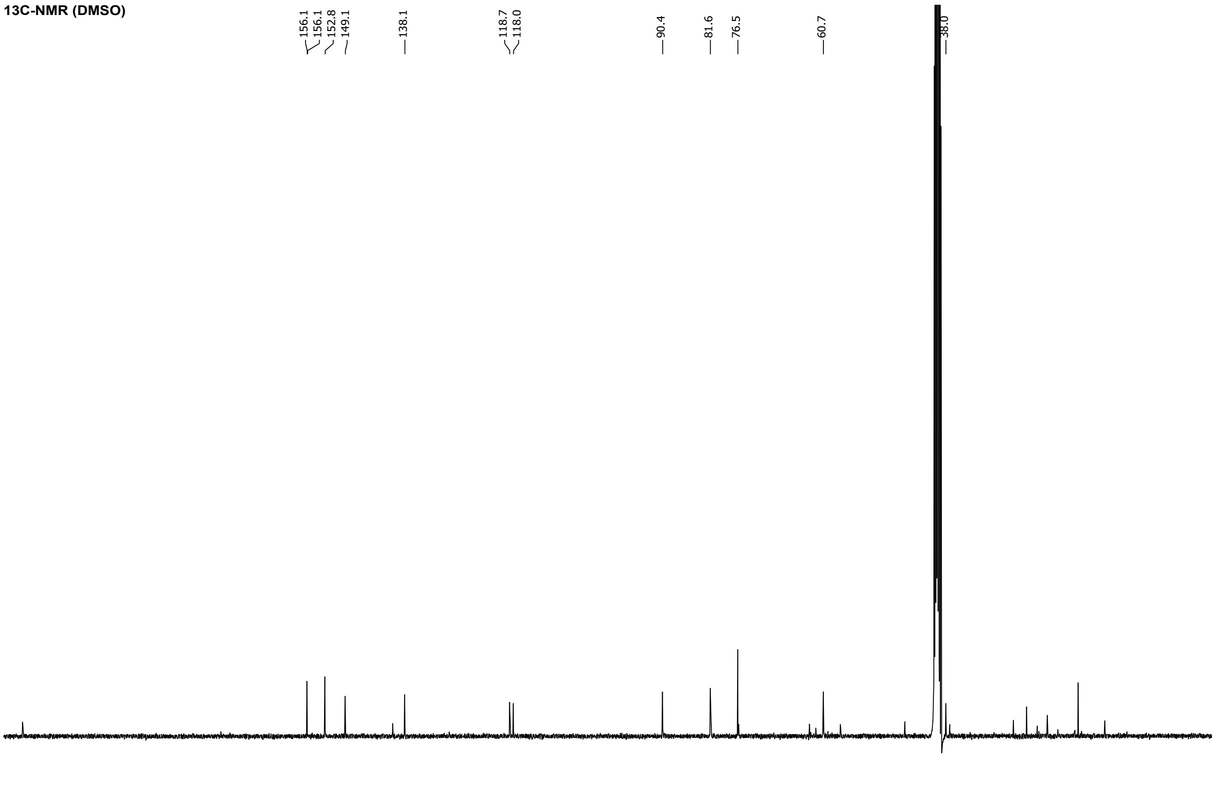

S01

1H-NMR (dmso)

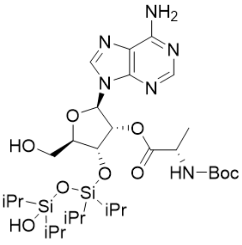

S01 - *Proposed Structure*

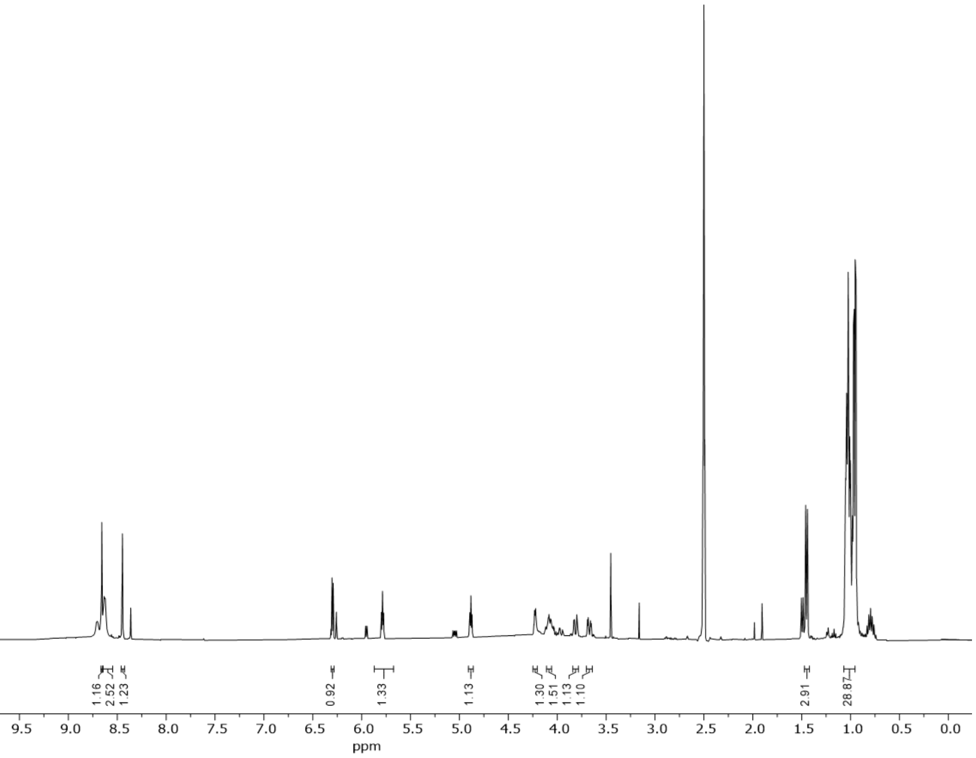

13C-NMR (dmso)

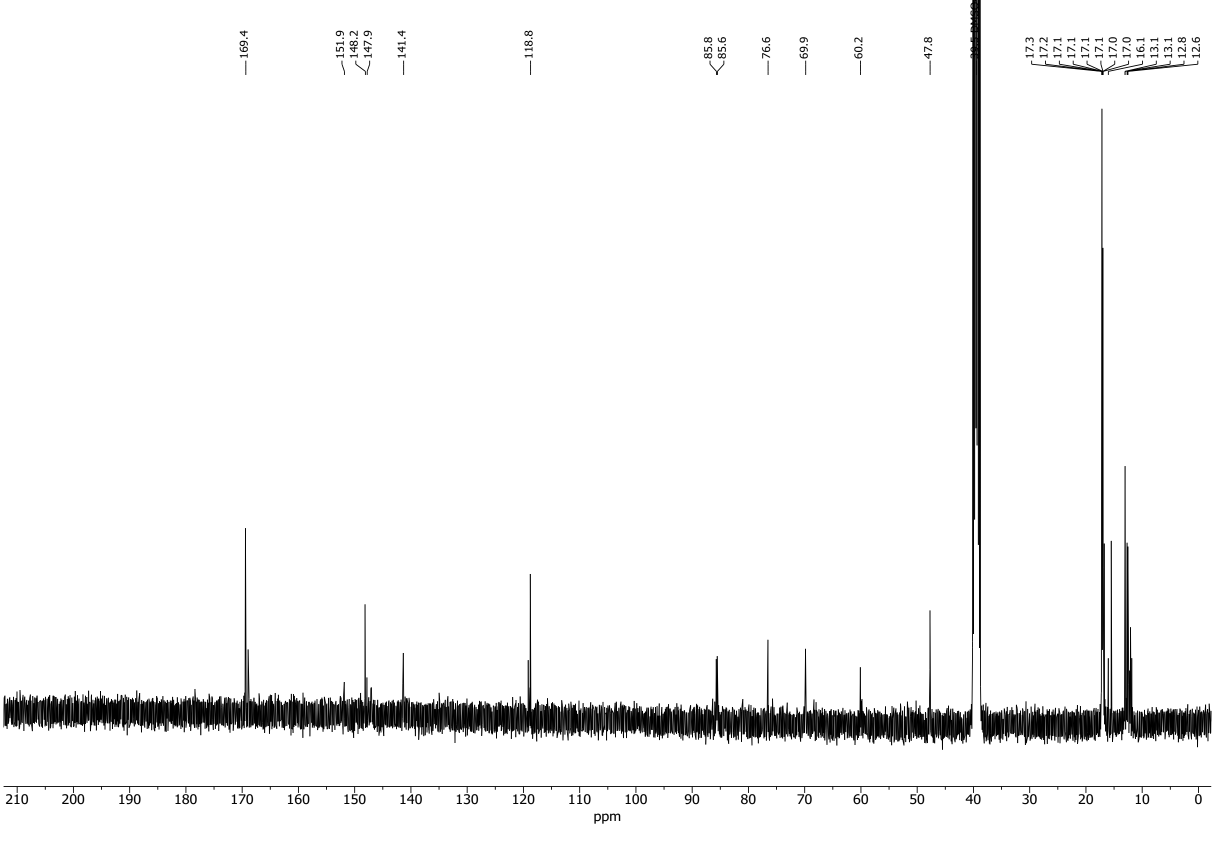

S02

1H-NMR (DMSO)

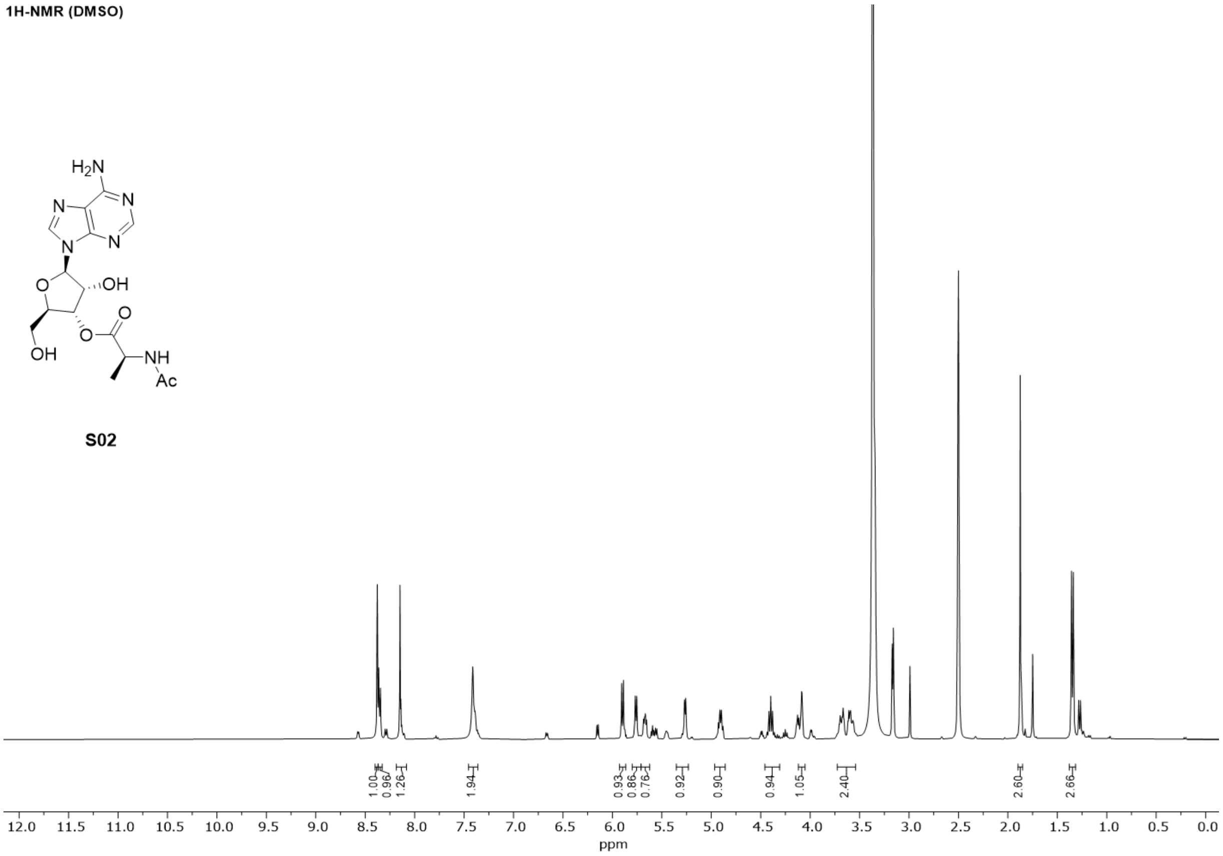

13C-NMR (DMSO)

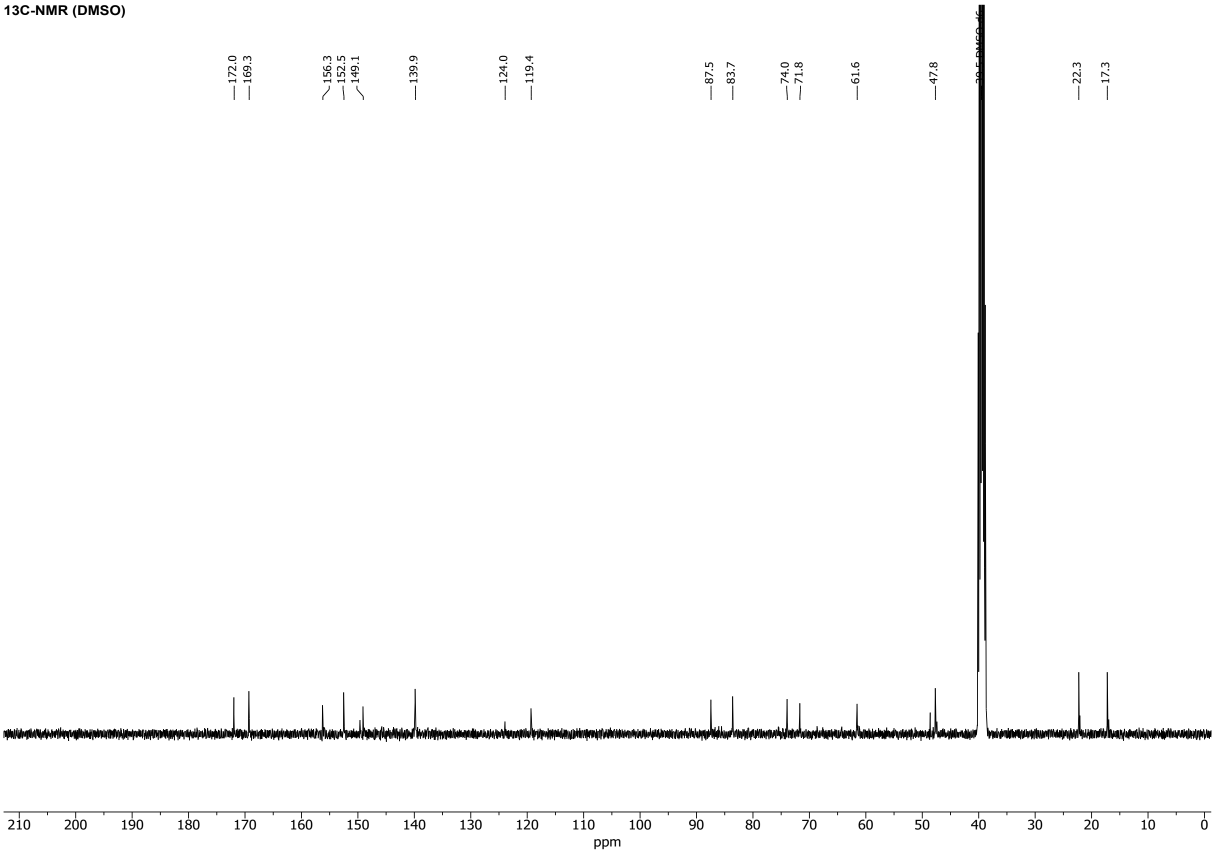

S03

1H-NMR (DMSO)

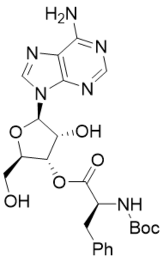

S03

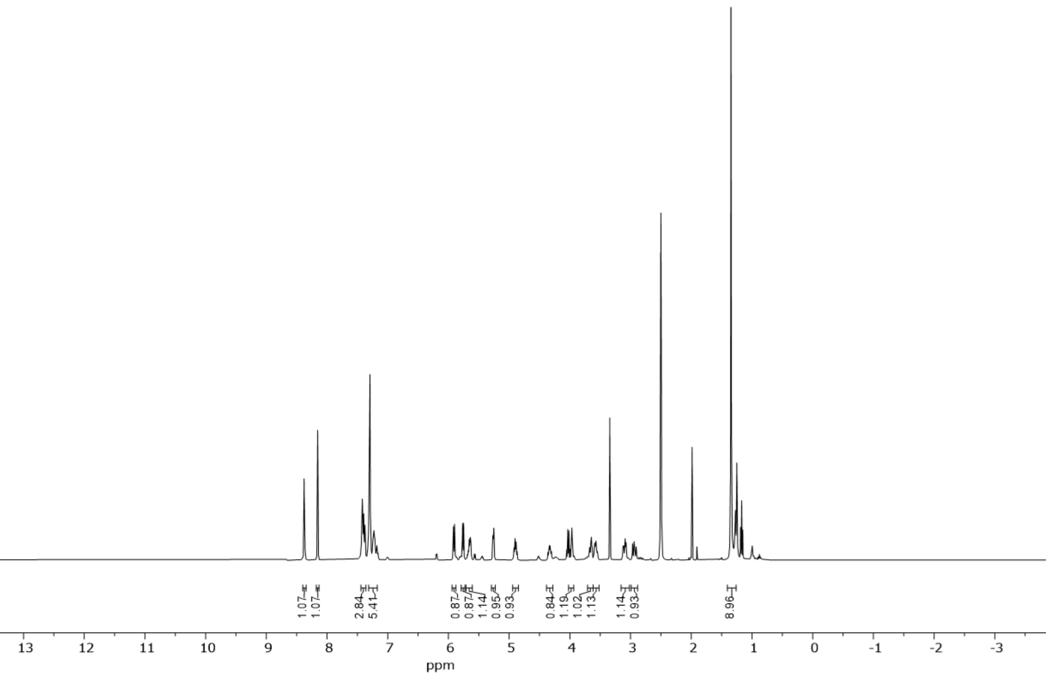

13C-NMR (DMSO)

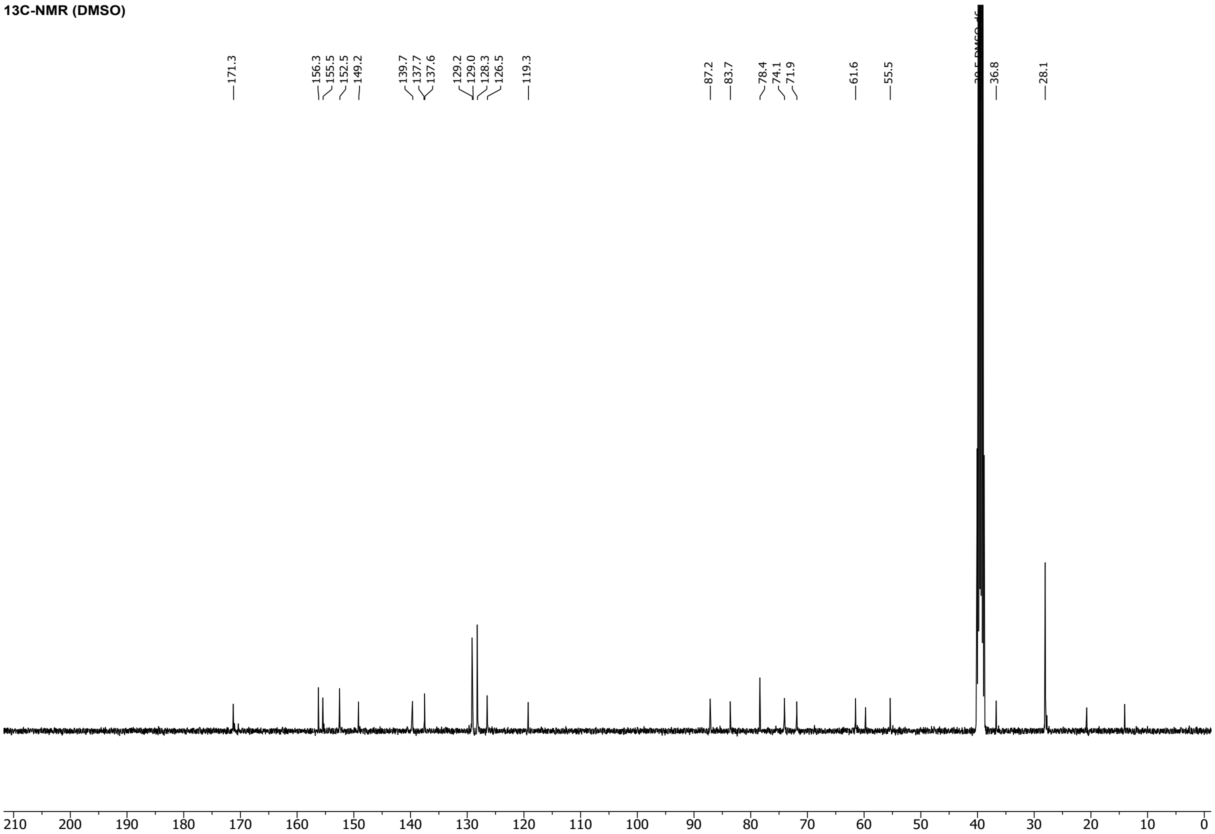

S04

1H-NMR (dmso)

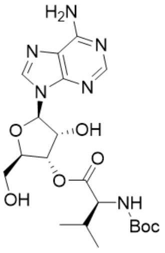

S04

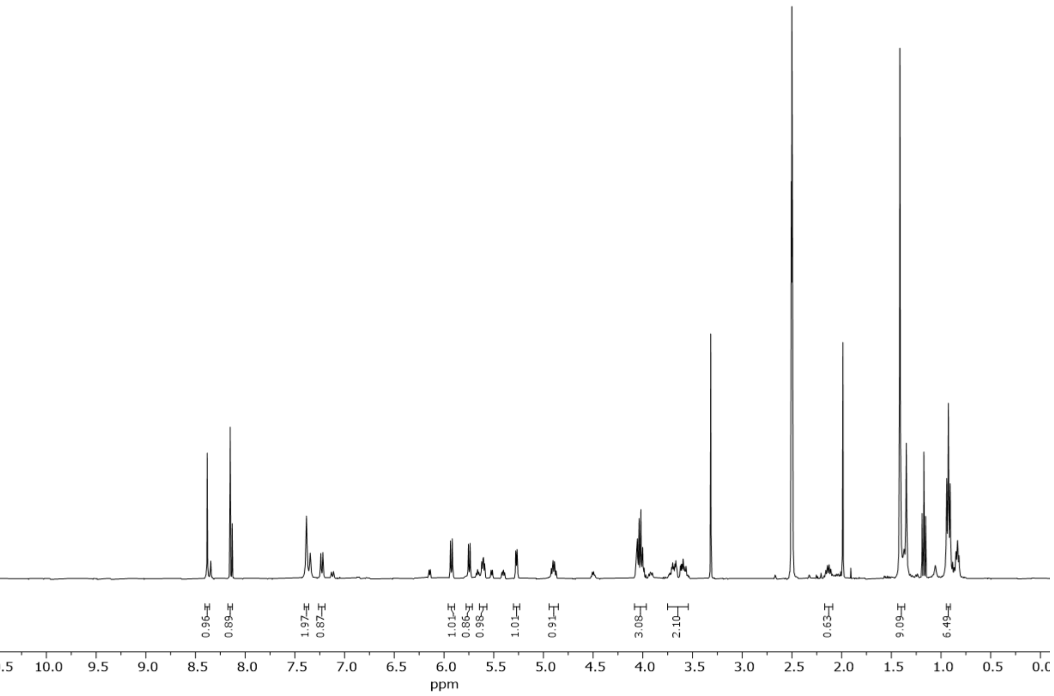

13C-NMR (dmso)

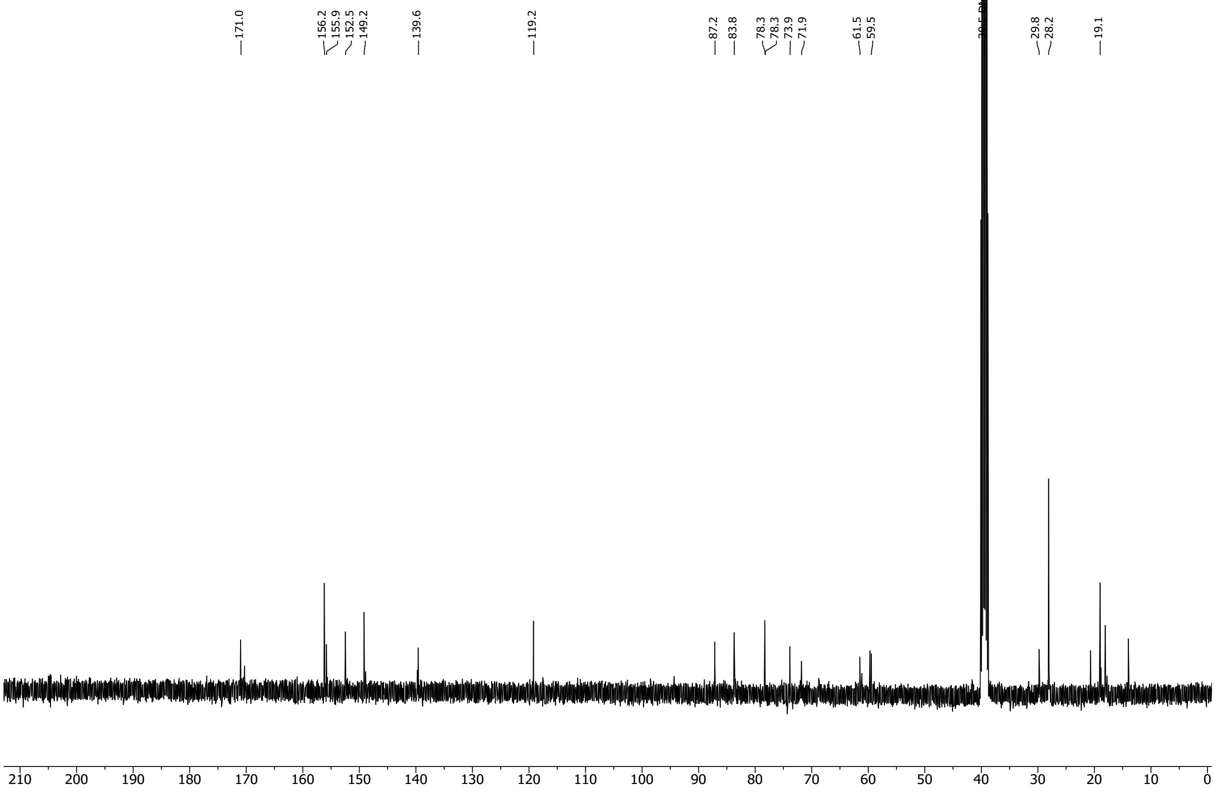

S05

1H-NMR (CDCl3)

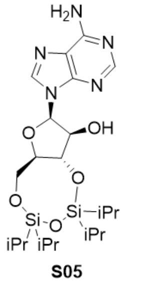

S05

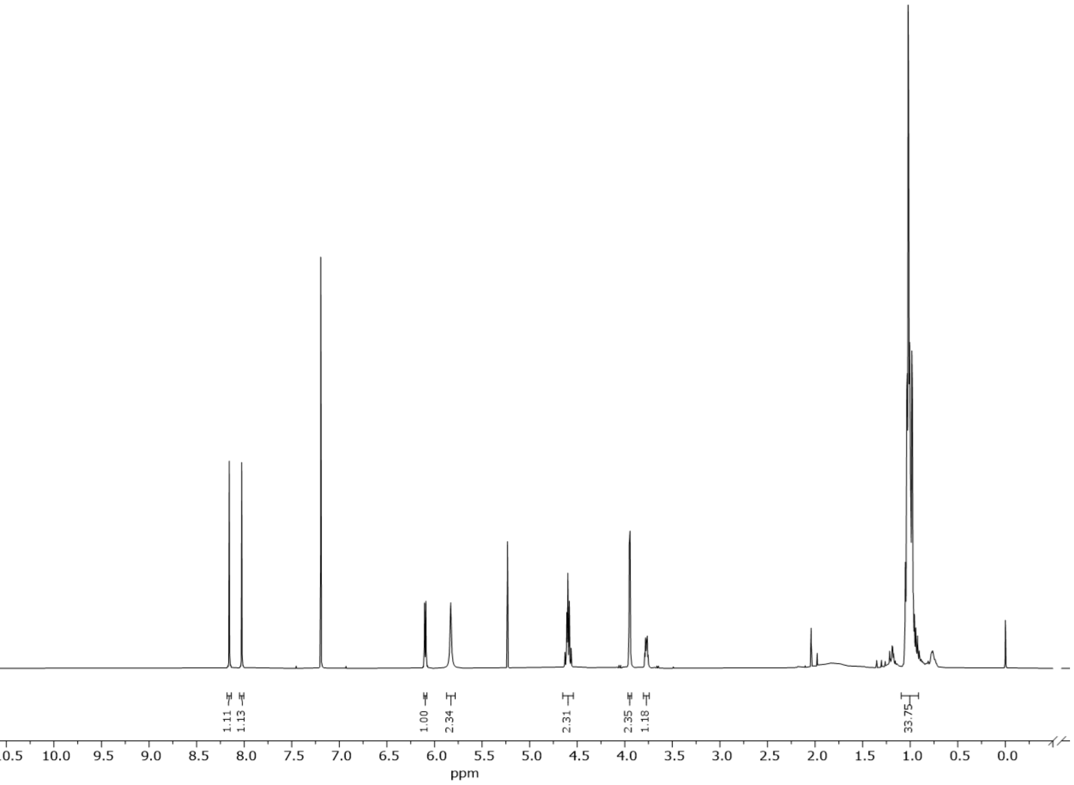

13C-NMR (CDCl3)

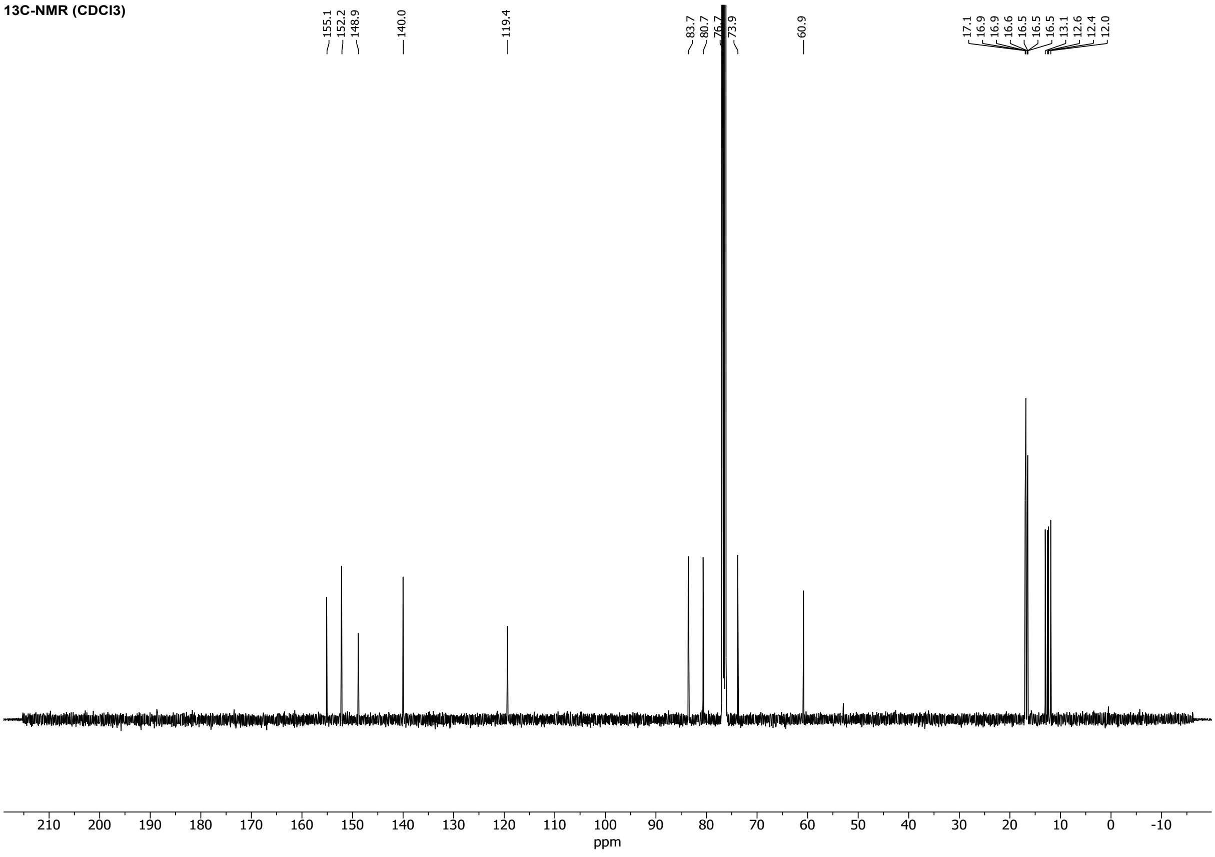

S06

1H-NMR (DMSO)

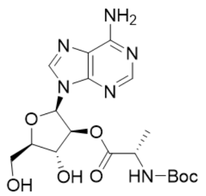

S06

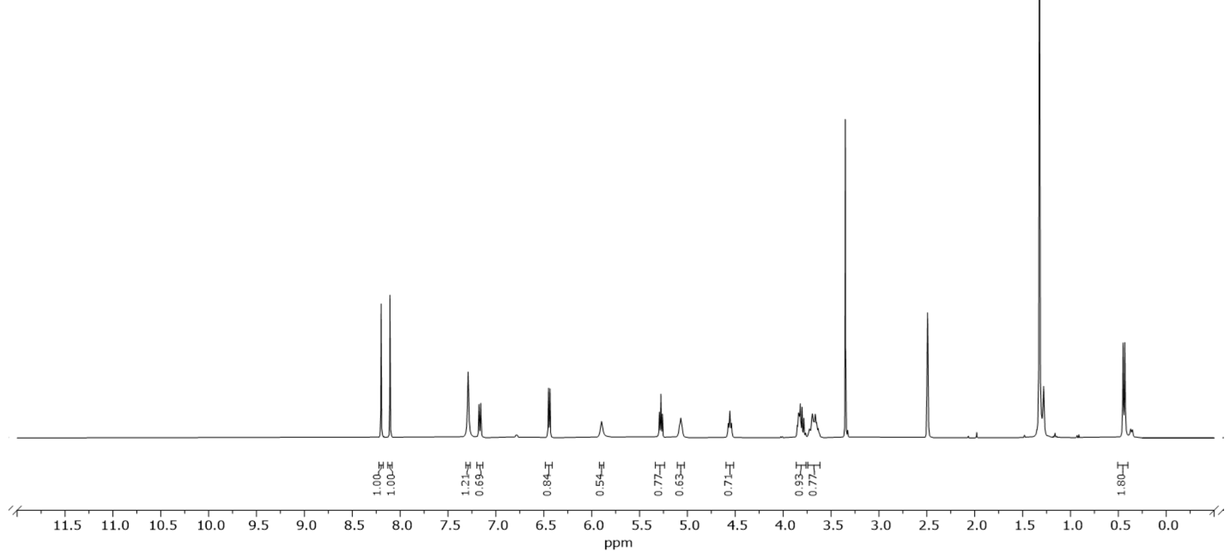

13C-NMR (DMSO)

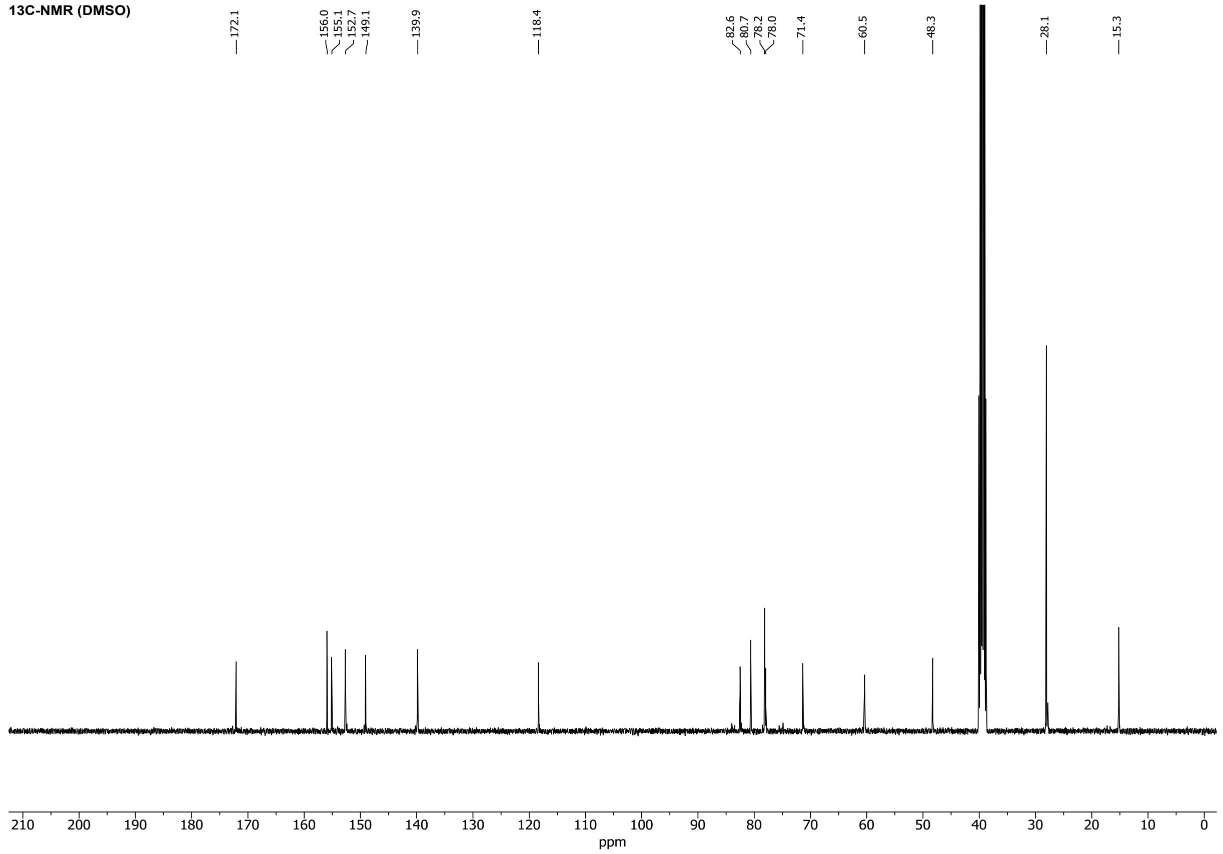

S07

1H-NMR (DMSO)

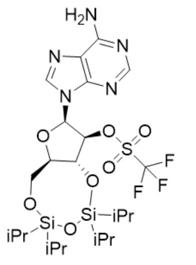

S07

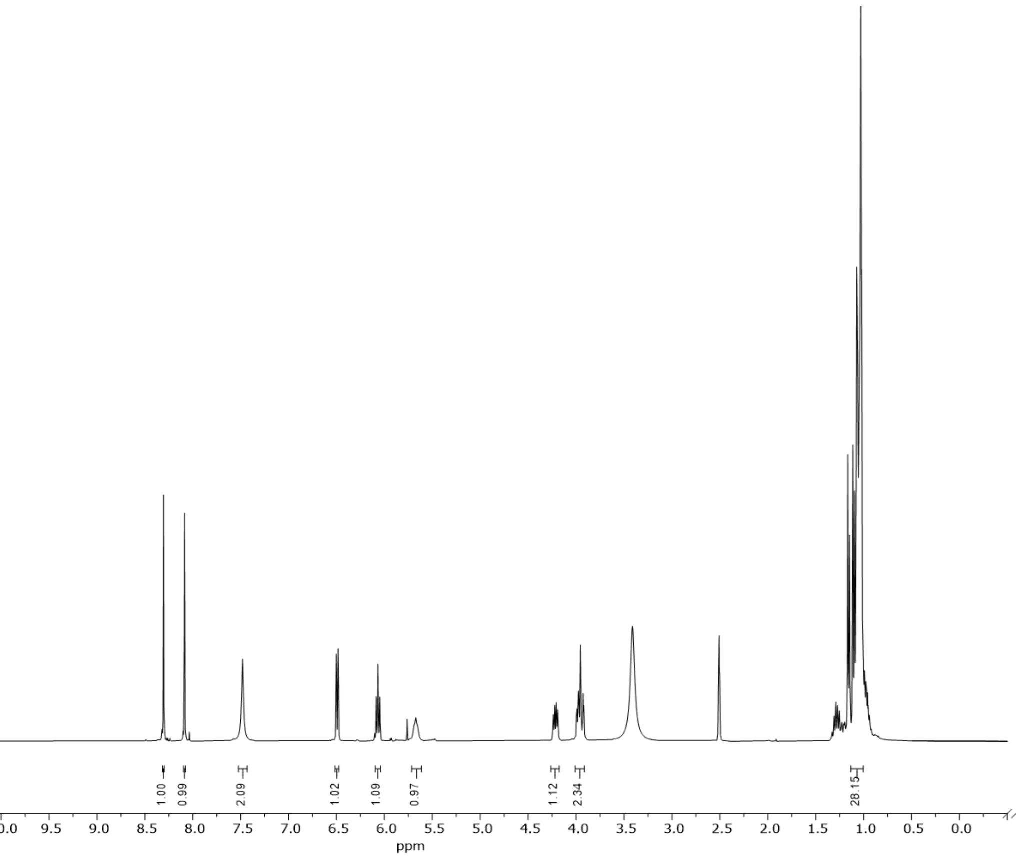

13C-NMR (DMSO)

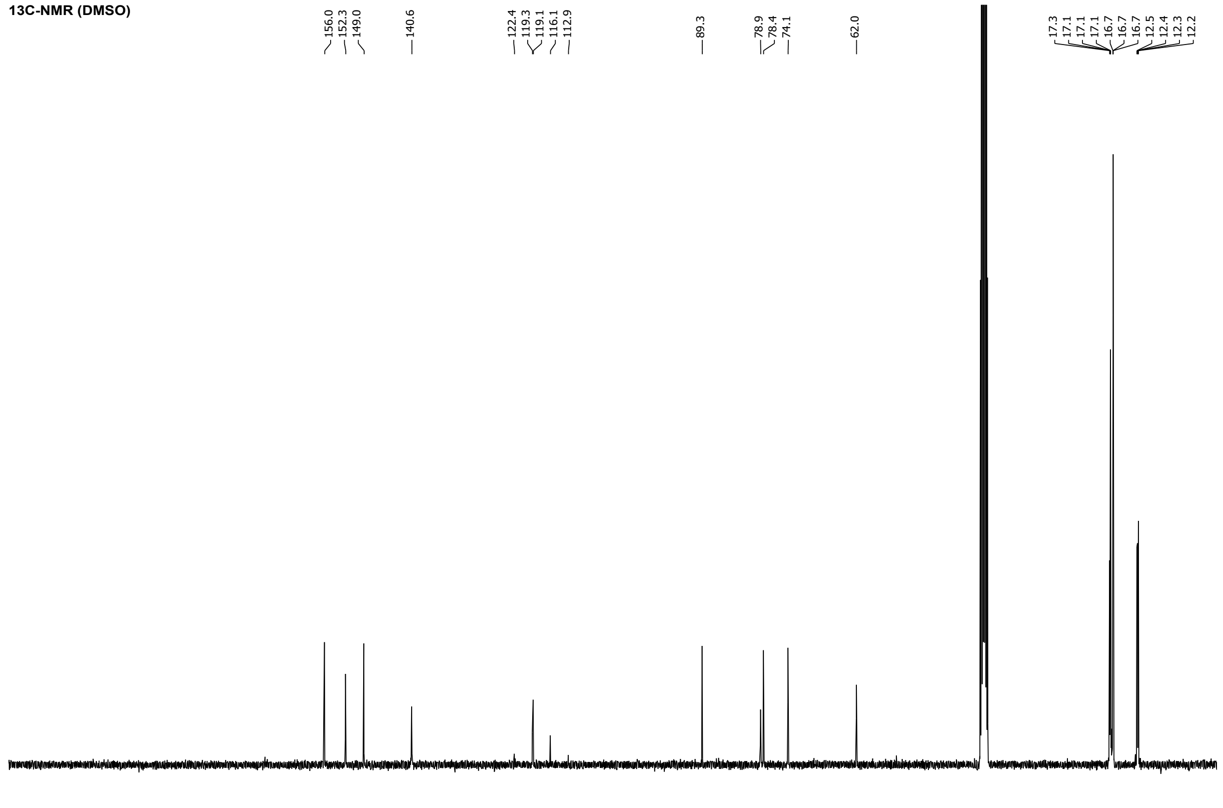

S09

1H-NMR (DMSO)

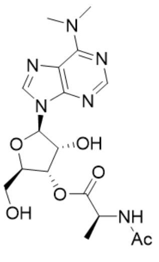

S09

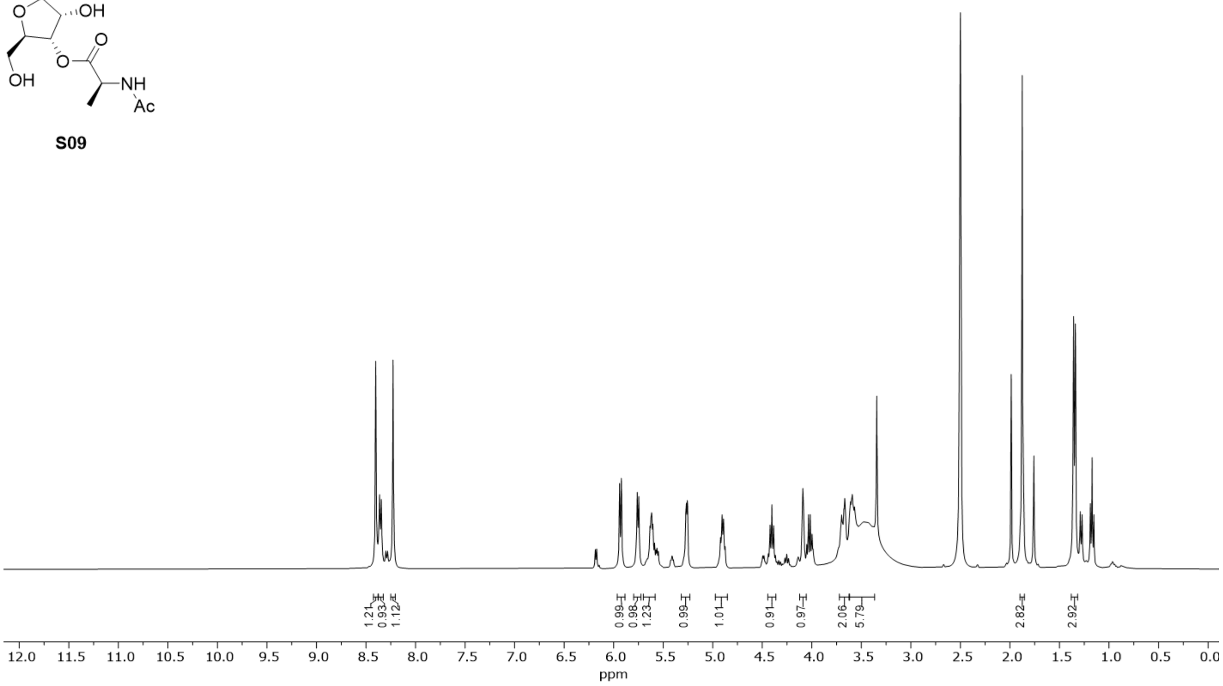

13C-NMR (DMSO)

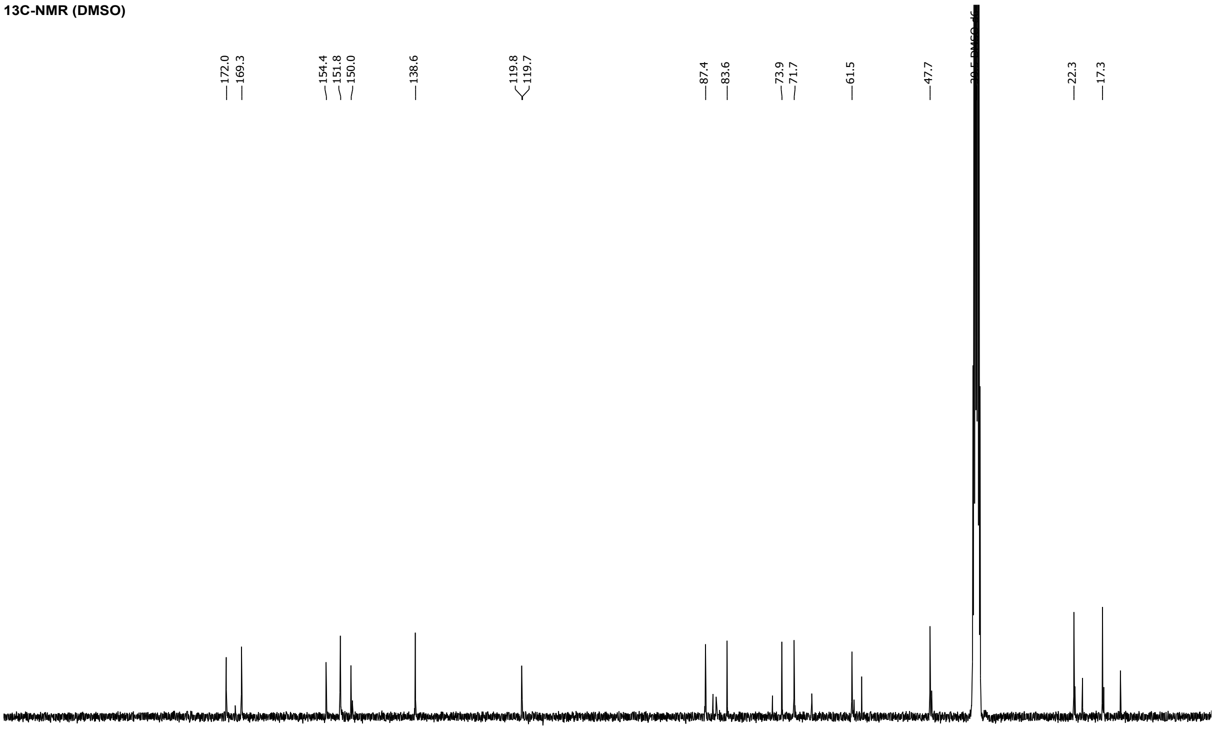

S11

1H-NMR (CDCl3)

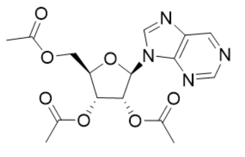

S11

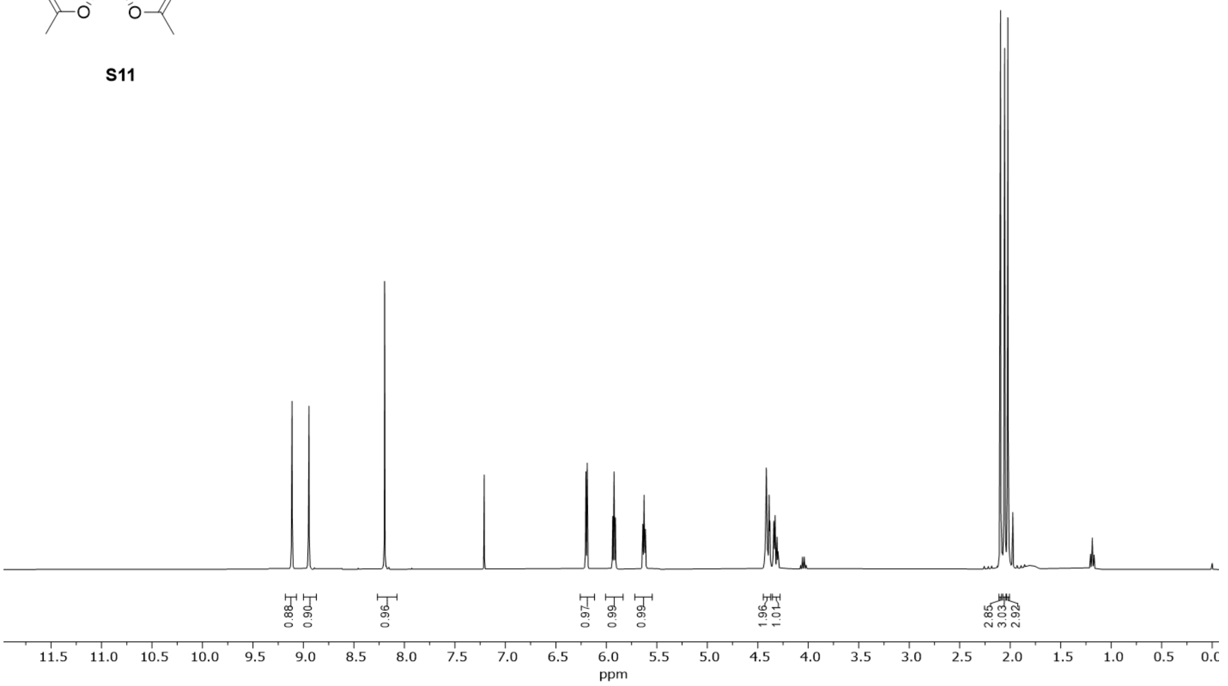

13C-NMR (CDCl3)

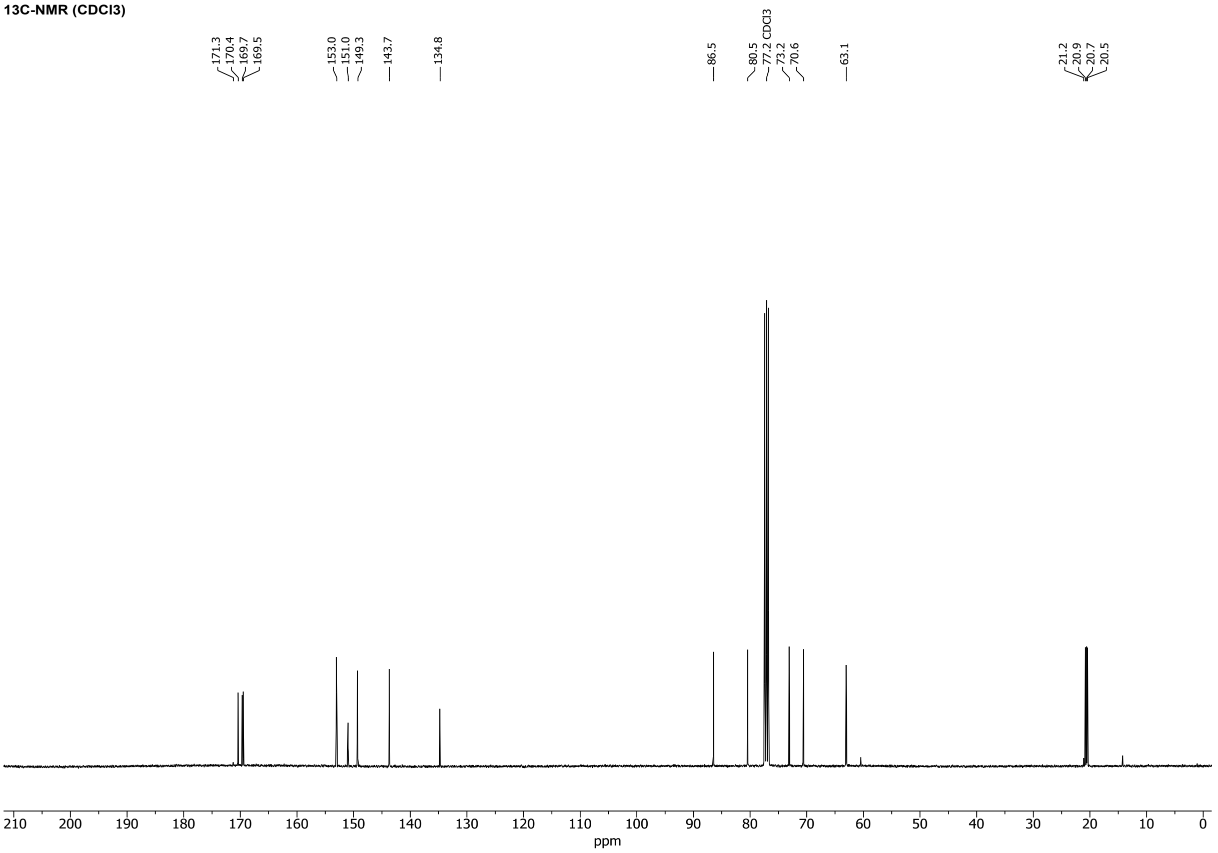

S12

1H-NMR (DMSO)

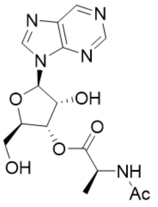

S12

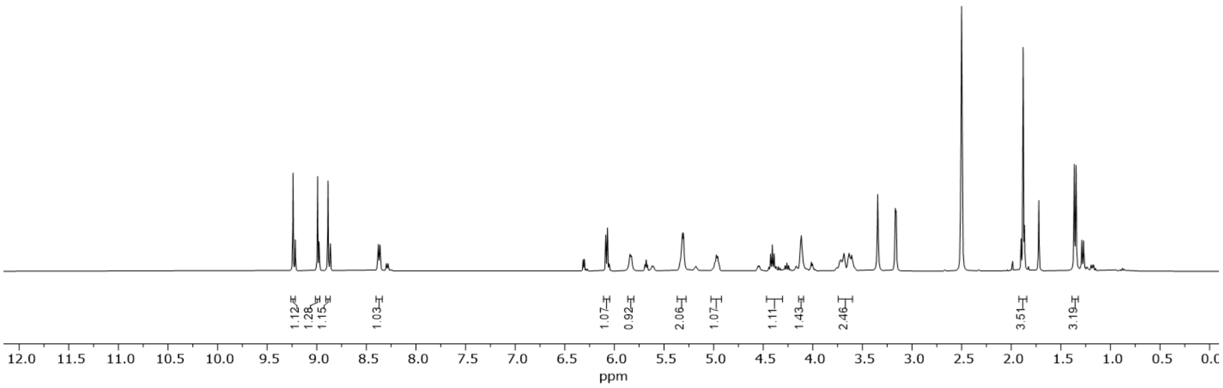

13C-NMR (DMSO)

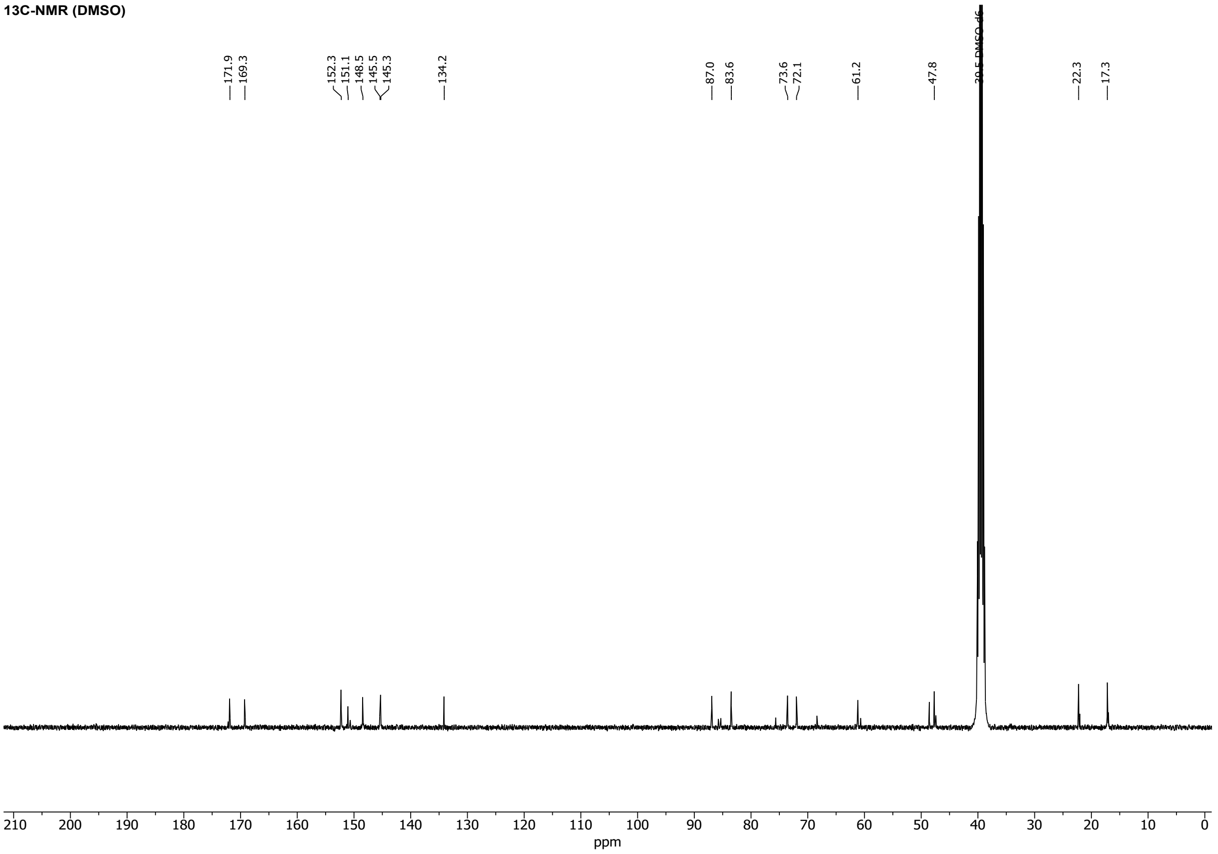

DFT Calculations

DFT calculations regarding the stability of 2'- and 3'-acylated adenosines in the 2'-3' acyl migration were performed to receive the optimized single point energies for both regio isomers utilizing ORCA 6.0.0.[4–12]

All structures were optimized applying the following command line:

! B3LYP[13] def2-SVP[14] def2/J D3BJ TIGHTSCF CPCM(THF) Opt Freq

For all calculations regarding the optimized structures the input as well as the corresponding output files are attached as additional files to the supplementary information. For all calculation no imaginary frequencies were obtained.

All visualizations of calculated structures were obtained utilizing CYLview20.

(2*R*,3*R*,4*R*,5*R*)-2-(6-amino-9*H*-purin-9-yl)-4-hydroxy-5-(hydroxymethyl)tetrahydrofuran-3-yl acetate (**5a**)

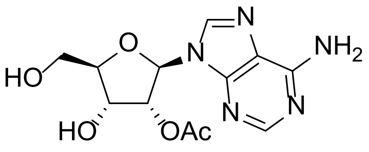

5a

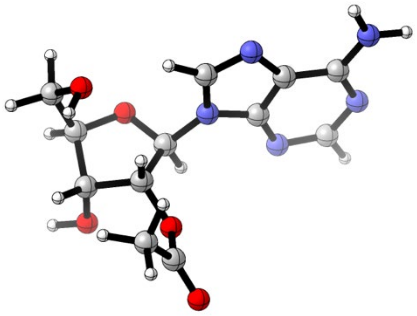

Cartesian coordinates (Angstroem)

N	-1.006706	11.413716	7.581210
C	-0.124761	10.533358	8.073557
N	0.950464	10.017939	7.472555
C	1.102025	10.487190	6.228973
C	0.266869	11.408246	5.579085
C	-0.843361	11.879439	6.325700
N	2.087780	10.187640	5.309622
C	1.808812	10.935649	4.176093
N	0.730054	11.672928	4.301951
N	-1.727656	12.763508	5.829381
C	4.718114	7.769272	4.481414
C	3.224068	8.091314	4.568833
C	3.207727	9.298578	5.518394
O	4.417363	9.981567	5.283033
C	5.317080	9.188621	4.486471
O	2.414986	7.066460	5.112998

O	5.061321	7.033875	5.633236
C	5.508290	9.831823	3.115847
O	4.324509	9.872585	2.332167
C	1.823757	6.113381	4.349635
C	1.960935	6.215693	2.850625
O	1.205399	5.245091	4.913134
H	2.463014	10.880162	3.304538
H	-1.613170	13.135240	4.895548
H	-2.497803	13.081623	6.404985
H	4.977450	7.223481	3.556142
H	2.867884	8.409847	3.583166
H	3.131183	8.947754	6.560372
H	6.297222	9.168417	4.992884
H	6.015662	6.873100	5.617834
H	6.324580	9.310538	2.585300
H	5.826824	10.873842	3.279363
H	4.279381	9.078382	1.781744
H	1.498534	7.145310	2.482604
H	3.017451	6.223048	2.543256
H	1.453476	5.355802	2.398369
H	-0.313558	10.195228	9.100142

E [Hartree] –1114.873344955552

(2*R*,3*S*,4*R*,5*R*)-5-(6-amino-9*H*-purin-9-yl)-4-hydroxy-2-(hydroxymethyl)tetrahydrofuran-3-yl acetate (**6a**)

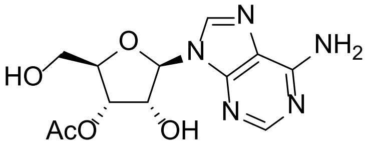

6a

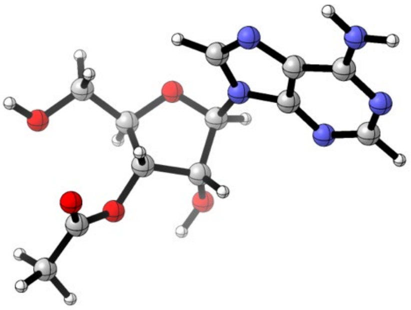

Cartesian coordinates (Angstroem)

N	-1.855781	9.241311	4.593083
C	-0.819684	8.942683	3.797603
N	-0.827620	8.247789	2.657120
C	-2.060293	7.834798	2.334111
C	-3.237372	8.066819	3.062311
C	-3.091760	8.817835	4.257595
N	-2.454041	7.095671	1.238957
C	-3.821196	6.924617	1.355231
N	-4.322717	7.490035	2.430551
N	-4.131436	9.117819	5.056312
C	-1.563362	4.257091	0.266285
C	-0.682536	5.448032	0.651124
C	-1.565731	6.614468	0.181287
O	-2.346158	6.133822	-0.887141
C	-2.162463	4.723905	-1.064260
O	0.498841	5.479681	-0.114216

O	-0.780497	3.081481	0.116274
C	-3.478002	4.076921	-1.455876
O	-3.181627	2.741569	-1.817172
C	-1.301682	1.901417	0.540938
C	-0.438612	0.754359	0.102308
O	-2.328995	1.818680	1.167787
H	-4.381615	6.378837	0.598678
H	-5.065252	8.805810	4.823772
H	-3.977296	9.653213	5.902112
H	-2.349310	4.089670	1.015268
H	-0.478044	5.502422	1.733693
H	-0.933583	7.456284	-0.140866
H	-1.423342	4.541839	-1.864481
H	0.888592	4.591728	-0.087514
H	-3.920609	4.646331	-2.296029
H	-4.182372	4.128589	-0.604777
H	-4.000929	2.229098	-1.800992
H	0.624638	0.983377	0.262687
H	-0.727084	-0.158887	0.636721
H	-0.590520	0.608842	-0.980110
H	0.157109	9.317554	4.128906

E [Hartree]	–1114.879362883667

(2*R*,3*R*,4*R*,5*R*)-2-(6-Amino-9*H*-purin-9-yl)-4-hydroxy-5-(hydroxymethyl)tetrahydrofuran-3-yl (*tert*-butoxycarbonyl)-L-alaninate (**S13**)

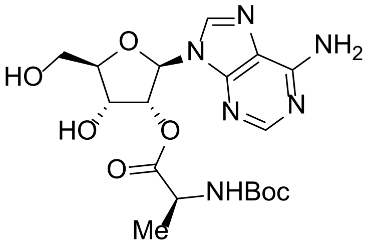

S13

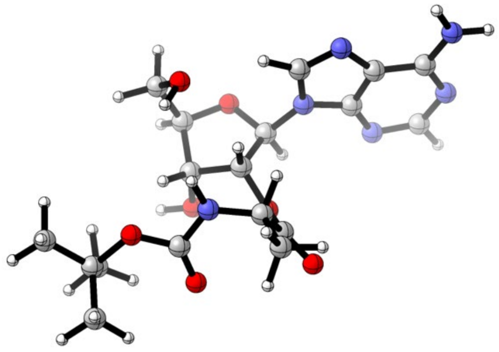

Cartesian coordinates (Angstroem)

N	-1.108537	11.286411	7.034597
C	-0.250919	10.434200	7.611484
N	0.899629	9.964726	7.126714
C	1.163009	10.452987	5.910193
C	0.369989	11.348922	5.181588
C	-0.829959	11.770342	5.807781
N	2.248257	10.197266	5.100139
C	2.059882	10.943290	3.950397
N	0.950571	11.642383	3.963302
N	-1.688558	12.626021	5.224849
C	4.978314	7.838652	4.456592
C	3.480665	8.144870	4.424158
C	3.358834	9.323642	5.402427
O	4.573324	10.027411	5.298915
C	5.560678	9.259780	4.584480

O	2.645267	7.089800	4.861040
O	5.239013	7.035362	5.584424
C	5.894223	9.942598	3.262510
O	4.808745	9.967191	2.345944
C	1.767409	6.415568	4.086307
C	1.800915	6.617064	2.561241
O	0.951215	5.716658	4.628798
C	0.798125	5.691907	1.873925
N	3.146864	6.492014	2.028367
H	2.801966	10.917984	3.150891
H	-1.494800	13.003188	4.306471
H	-2.531835	12.902294	5.712818
H	5.320175	7.356606	3.527222
H	3.218991	8.493118	3.421911
H	3.186471	8.946987	6.423764
H	6.478383	9.226284	5.195878
H	6.189824	6.857632	5.616813
H	6.780427	9.454153	2.820416
H	6.161182	10.988584	3.482695
H	4.856677	9.182910	1.781741
H	1.496464	7.663819	2.388743
H	1.061756	4.641054	2.060532
H	0.812098	5.880379	0.790354
H	-0.217845	5.868696	2.253024

C	3.958088	5.453976	2.373866
H	3.417882	7.073987	1.243411
O	3.667517	4.635795	3.232116
O	5.106319	5.503819	1.680312
C	6.202535	4.550310	1.922866
C	6.743042	4.743293	3.340929
H	7.155997	5.757243	3.459928
H	7.554599	4.022481	3.522626
H	5.958318	4.588273	4.092184
C	7.244527	4.961407	0.885510
H	7.536963	6.012790	1.028993
H	6.845100	4.842591	-0.133221
H	8.140894	4.331642	0.986856
C	5.723552	3.120418	1.669005
H	5.000016	2.800376	2.428202
H	6.588212	2.439658	1.690753
H	5.254047	3.049027	0.675495
H	-0.535399	10.078829	8.609746

E [Hartree]	–1554.846035159974

(2*R*,3*S*,4*R*,5*R*)-5-(6-amino-9*H*-purin-9-yl)-4-hydroxy-2-(hydroxymethyl)tetrahydrofuran-3-yl (*tert*-butoxycarbonyl)-L-alaninate (**12a**)

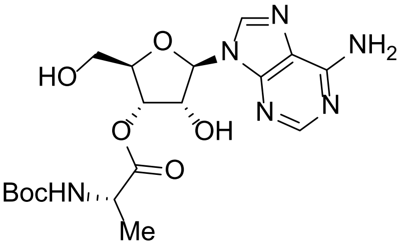

12a

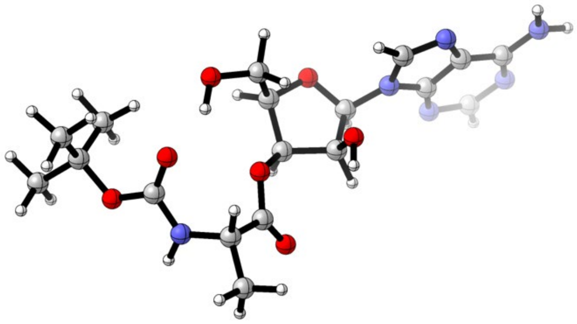

Cartesian coordinates (Angstroem)

C	-0.861787	-1.428967	1.143935
N	2.122331	0.227622	2.450432
C	-2.062668	0.132898	2.854085
O	-1.829890	-2.358913	1.625426
O	0.475263	-1.857542	3.149339
C	1.079918	-0.172651	1.542147
C	-1.271635	0.001525	1.542930
C	0.535598	-1.591341	1.769883
O	-0.026290	0.701800	1.675383
O	-3.447524	-0.045081	2.698860
C	3.446065	0.424389	2.117327
C	4.070843	0.794927	3.313934
N	3.160546	0.834346	4.352167
C	2.021191	0.490050	3.803776
N	4.029019	0.294060	0.920915
C	5.334137	0.567434	0.972585

N	6.063739	0.929496	2.035498
C	5.461636	1.055290	3.235393
N	6.190825	1.418047	4.305851
C	-1.736995	-3.636327	1.216220
O	-0.827026	-4.055530	0.541802
C	-2.955475	-4.443329	1.674066
N	-4.092742	-4.055088	0.854666
O	-5.783750	-2.781881	0.200598
C	-6.656894	-1.594551	0.183709
C	-7.561759	-1.573166	1.416021
C	-5.790605	-0.340056	0.067777
C	-7.481191	-1.804599	-1.084561
C	-4.897435	-3.006515	1.175620
O	-4.815442	-2.385757	2.229153
C	-2.701862	-5.941053	1.597658
H	-0.778854	-1.525901	0.051213
H	-1.897099	1.166622	3.199592
H	-1.632508	-0.544071	3.612092
H	0.304704	-2.800380	3.281175
H	1.519210	-0.108090	0.532206
H	-1.859320	0.478776	0.741396
H	1.145247	-2.335780	1.237182
H	-3.670250	-0.988145	2.577512
H	1.064021	0.415534	4.315215

H	5.882949	0.485417	0.026099
H	7.182224	1.596715	4.202451
H	5.754694	1.520578	5.212800
H	-3.183854	-4.131983	2.703124
H	-4.164985	-4.436762	-0.083862
H	-8.313222	-0.777521	1.297201
H	-6.987617	-1.387018	2.330998
H	-8.091612	-2.533492	1.515624
H	-6.440363	0.543648	-0.025916
H	-5.158765	-0.398064	-0.832576
H	-5.142994	-0.204383	0.943219
H	-8.167148	-0.956788	-1.229400
H	-8.075527	-2.728211	-1.011123
H	-6.823717	-1.878247	-1.964117
H	-2.466492	-6.244012	0.565883
H	-3.593840	-6.487085	1.936584
H	-1.850534	-6.218903	2.235945

E [Hartree]	–1554.855441294987

(2*R*,3*R*,4*R*,5*R*)-2-(6-Amino-9*H*-purin-9-yl)-4-hydroxy-5-(hydroxymethyl)tetrahydrofuran-3-yl L-alaninate (**16a**)

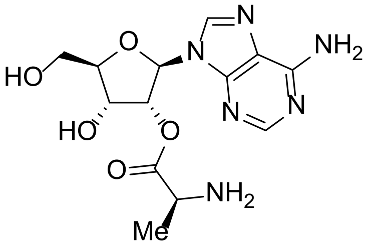

16a

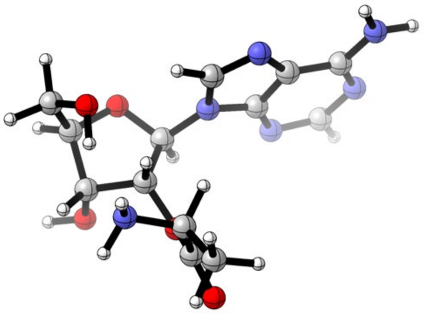

Cartesian coordinates (Angstroem)

N	-1.006164	11.303418	7.552441
C	-0.056855	10.530117	8.096003
N	1.049158	10.052790	7.523011
C	1.155393	10.439819	6.247308
C	0.251143	11.245153	5.542668
C	-0.886055	11.685370	6.265334
N	2.151756	10.146485	5.341963
C	1.805493	10.782199	4.162842
N	0.678286	11.447052	4.245154
N	-1.837415	12.459942	5.713969
C	4.855151	7.772328	4.659181
C	3.368304	8.088004	4.680116
C	3.316567	9.324751	5.578054
O	4.486818	10.037141	5.223966
C	5.399069	9.196709	4.479720
O	2.545143	7.024976	5.108227

O	5.199873	7.201901	5.901993
C	5.518975	9.643965	3.020493
O	4.342654	9.513088	2.244344
C	1.812066	6.304329	4.228244
C	1.678252	6.788568	2.769005
O	1.239975	5.326357	4.639911
C	0.624807	5.952182	2.045936
N	2.980111	6.822999	2.073270
H	-0.213194	10.256266	9.146877
H	2.450335	10.705725	3.285691
H	-1.756604	12.767023	4.753520
H	-2.626576	12.761796	6.272246
H	5.122600	7.113938	3.815415
H	3.081613	8.418017	3.685389
H	3.301466	9.054347	6.645873
H	6.395221	9.284713	4.944489
H	6.154601	7.043242	5.908152
H	6.364304	9.087123	2.571872
H	5.786669	10.712308	3.014320
H	4.117987	8.566402	2.114023
H	1.315048	7.830874	2.828312
H	0.932598	4.896513	2.000192
H	0.499518	6.330719	1.019644
H	-0.347210	6.004212	2.557863

H	3.414887	5.897730	2.102846
H	2.799292	7.001846	1.082994

E [Hartree]	–1209.422497607656

(2*R*,3*S*,4*R*,5*R*)-5-(6-Amino-9*H*-purin-9-yl)-4-hydroxy-2-(hydroxymethyl)tetrahydrofuran-3-yl L-alaninate (**17a**)

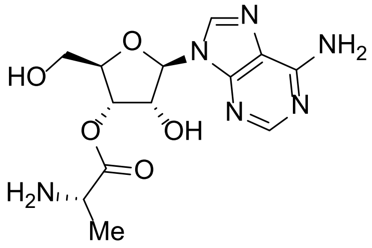

17a

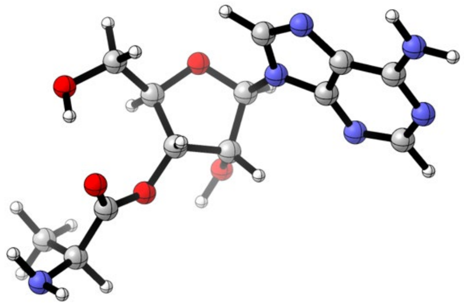

Cartesian coordinates (Angstroem)

N	-1.006164	11.303418	7.552441
C	-0.056855	10.530117	8.096003
N	1.049158	10.052790	7.523011
C	1.155393	10.439819	6.247308
C	0.251143	11.245153	5.542668
C	-0.886055	11.685370	6.265334
N	2.151756	10.146485	5.341963
C	1.805493	10.782199	4.162842
N	0.678286	11.447052	4.245154
N	-1.837415	12.459942	5.713969
C	4.855151	7.772328	4.659181
C	3.368304	8.088004	4.680116
C	3.316567	9.324751	5.578054
O	4.486818	10.037141	5.223966
C	5.399069	9.196709	4.479720
O	2.545143	7.024976	5.108227

O	5.199873	7.201901	5.901993
C	5.518975	9.643965	3.020493
O	4.342654	9.513088	2.244344
C	1.812066	6.304329	4.228244
C	1.678252	6.788568	2.769005
O	1.239975	5.326357	4.639911
C	0.624807	5.952182	2.045936
N	2.980111	6.822999	2.073270
H	-0.213194	10.256266	9.146877
H	2.450335	10.705725	3.285691
H	-1.756604	12.767023	4.753520
H	-2.626576	12.761796	6.272246
H	5.122600	7.113938	3.815415
H	3.081613	8.418017	3.685389
H	3.301466	9.054347	6.645873
H	6.395221	9.284713	4.944489
H	6.154601	7.043242	5.908152
H	6.364304	9.087123	2.571872
H	5.786669	10.712308	3.014320
H	4.117987	8.566402	2.114023
H	1.315048	7.830874	2.828312
H	0.932598	4.896513	2.000192
H	0.499518	6.330719	1.019644
H	-0.347210	6.004212	2.557863

H	3.414887	5.897730	2.102846
H	2.799292	7.001846	1.082994

E [Hartree]	–1209.422493691136

Supplemental Bibliography

[1] K. Sanada, A. Washio, H. Ishikawa, Y. Yoshida, T. Mino, M. Sakamoto, *Angew. Chem. Int. Ed.* **2022**, *61*, e202201268.

[2] S. G. Patching, S. A. Baldwin, A. D. Baldwin, J. D. Young, M. P. Gallagher, P. J. F. Henderson, R. B. Herbert, *Org. Biomol. Chem.* **2005**, *3*, 462–470.

[3] Z.-D. Shi, B.-H. Yang, Y.-L. Wu, *Tetrahedron* **2002**, *58*, 3287–3296.

[4] F. Neese, *J. Comput. Chem.* **2003**, *24*, 1740–1747.

[5] F. Neese, F. Wennmohs, A. Hansen, U. Becker, *Chem. Phys.* **2009**, *356*, 98–109.

[6] D. Bykov, T. Petrenko, R. Izsák, S. Kossmann, U. Becker, E. Valeev, F. Neese, *Mol. Phys.* **2015**, *113*, 1961–1977.

[7] M. Garcia-Ratés, F. Neese, *J. Comput. Chem.* **2019**, *40*, 1816–1828.

[8] M. Garcia-Ratés, F. Neese, *J. Comput. Chem.* **2020**, *41*, 922–939.

[9] B. Helmich-Paris, B. de Souza, F. Neese, R. Izsák, *J. Chem. Phys.* **2021**, *155*, 104109.

[10] F. Neese, *J. Comput. Chem.* **2023**, *44*, 381–396.

[11] S. Grimme, J. Antony, S. Ehrlich, H. Krieg, *J. Chem. Phys.* **2010**, *132*, 154104.

[12] S. Grimme, S. Ehrlich, L. Goerigk, *J. Comput. Chem.* **2011**, *32*, 1456–1465.

[13] A. D. Becke, *J. Chem. Phys.* **1993**, *98*, 1372–1377.

[14] F. Weigend, R. Ahlrichs, *Phys. Chem. Chem. Phys.* **2005**, *7*, 3297–3305.
